# Estrogen deprivation induces hepatic inflammation, Indoleamine-2,3-dioxygenase 1, tryptophan catabolism, and plasma cholesterol

**DOI:** 10.1038/s41598-026-48938-w

**Published:** 2026-04-24

**Authors:** Prarthana Guha, Ashcharya Rishi, Avisankar Chini, Nagashree Bhat, Pavan K. Gondrala, Blake Brady, Hamid R. Baniasadi, Linda I. Perrotti, Subhrangsu S. Mandal

**Affiliations:** 1 Gene Regulation and Epigenetics Research Laboratory, Department of Chemistry and Biochemistry, The University of Texas at Arlington, Arlington, TX 76019 USA; 2 Department of Psychology, The University of Texas at Arlington, Arlington, TX 76019 USA; 3 Department of Biochemistry, University of Texas Southwestern Medical Center at Dallas, Dallas, 75390 TX USA

**Keywords:** Biochemistry, Diseases, Endocrinology, Medical research, Physiology

## Abstract

Inflammation is a central mediator linking metabolic dysfunction to severe human disease. Imbalance or loss of ovarian hormone (such as in post-menopausal women) contributes to increased risk of cardiovascular diseases, obesity and others. Here, using ovariectomized (OVX) Long-Evans rats as model for estrogen deprivation, we demonstrate that estrogen deprivation induces hepatic inflammation, activates tryptophan catabolism, systemic inflammation and disrupted cholesterol homeostasis. OVX animals gained more weight and developed an atherogenic plasma profile with increased LDL and total cholesterol and reduced HDL levels compared to intact female animals, which was reversed by estradiol (E2) administration. Ovariectomy results in elevation of hepatic pro-inflammation cytokine (e.g. TNFα, IL6), tryptophan catabolic enzymes (e.g. IDO1, and TDO2) and reduced reverse cholesterol associated gene SR-BI expression and E2- administration also suppressed the ovariectomy-induced hepatic inflammation resulting in reduction of TNFα, IL6, IDO1 and TDO2 while elevation of SR-BI expression. Plasma kynurenine, nitric oxide and lactate were elevated upon ovariectomy suggesting increased system Trp-catabolism, inflammation, each was reversed by estrogen. Targeted LC-MS metabolomics analysis revealed enhanced Trp-to-kynurenine flux, elevated lactate, accumulation of citrate/isocitrate/aconitate, and a reduced α-ketoglutarate/aconitate ratio (~ 0.6) restored by estradiol (~ 3.6). Together, our studies suggest a a link between estrogen signaling and hepatic immune–metabolism via regulation of Trp-catabolism, this open up potential novel signaling pathways for treating cardiometabolic disease and hormonal disorders.

**Supplementary Information:**

The online version contains supplementary material available at 10.1038/s41598-026-48938-w.

## Introduction

Inflammation is a fundamental biological process that protects tissues from injury and infection by orchestrating immune, vascular, and metabolic responses^1–8^. Under physiological conditions, this process is transient and resolves once the initiating insult is eliminated. However, when inflammation becomes chronic or dysregulated, it promotes tissue injury, fibrosis, and metabolic dysfunction, thereby contributing to a spectrum of diseases including cardiovascular disease (CVD), metabolic syndrome, and fatty liver disease^9–11^. In particular, hepatic inflammation has emerged as a critical mediator linking metabolic imbalance and cardiovascular risk^11–15^. Epidemiological studies have consistently shown a striking sexual dimorphism in cardiovascular health. Premenopausal women exhibit a markedly lower incidence of CVD compared with age-matched men, but this protection diminishes rapidly following menopause. The transition to menopause is accompanied by a steep decline in circulating ovarian hormones, especially estradiol (E2), which profoundly alter lipid metabolism, vascular function, and inflammatory responses. Menopausal estrogen loss has been associated with low ERα mediated dysregulation of low-density lipoprotein receptor (LDLR) and suppression of proprotein convertase subtilisin/kexin type 9 (PCSK9), eventually leading to higher levels of LDL in blood^16,17^.

The loss of estrogen’s regulatory influence on hepatic and systemic metabolism is recognized as a major factor contributing to the surge in cardiovascular morbidity and mortality among postmenopausal women^18–20^. The liver plays a central role in lipid and glucose metabolism and homeostasis^10,11,21–29^. For example, liver assembles very low-density lipoprotein (VLDL) containing apolipoprotein B100 (apoB100) which delivers cholesterol to peripheral tissue and is eventually converted into LDL which is taken up by peripheral tissue via LDLR which may lead to several different outcomes including storage of cholesteryl esters^10,11,21–29^. To remove excess cholesterol, Scavenger Receptor Class B Type I (SR-BI) on the liver selectively mediates uptake of cholesteryl esters from High‑Density Lipoprotein (HDL), thus moving cholesterol from peripheral tissues back to the liver. Meanwhile, in macrophages and other cells, ACAT1 (acyl-CoA: cholesterol acyltransferase-1) esterifies free cholesterol for storage as cholesteryl esters in lipid droplets, a step that helps prevent cytotoxic free-cholesterol accumulation but also influences cholesterol efflux and foam-cell formation, modulating atherosclerosis development^28,30–36^. These lipid transport and homeostasis are controlled by ovarian hormones.

Hepatic inflammation disrupts these processes, impairing cholesterol and lipoprotein metabolism and transport and promoting the release of pro-inflammatory cytokines that exacerbate vascular injury^17,37–41^. Estrogen exerts multifaceted protective effects on the liver through both genomic and non-genomic mechanisms^17,41^. It regulates hepatic lipid oxidation, modulates mitochondrial function, and suppresses oxidative stress and inflammatory signaling via estrogen receptor-dependent pathways. Consequently, estrogen deficiency induces hepatic steatosis, inflammatory activation, and dyslipidemia—hallmarks of metabolic dysfunction that directly increase CVD risk. The molecular mechanisms that regulate this process under hormonal deprivation remain poorly understood.

Inflammation signaling may follow diverse signaling pathways that regulate production of cytokines and inflammatory response. Tryptophan (Trp) catabolism is recognized as a major contributor in inflammatory response^42–51^. Tryptophan may be metabolized through several distinct biochemical pathways, each leading to different physiological outcomes. One major route is the kynurenine pathway, which produces metabolites involved in immune regulation and neuroactive signaling. Another important pathway is the serotonin synthesis route, through which tryptophan is converted into serotonin, a key neurotransmitter that influences mood and cognition^44,46,48^. Additionally, serotonin pathway can lead to the synthesis of melatonin, a hormone that regulates circadian rhythms, or converted into niacin, an essential component of NAD⁺ and NADP⁺. The balance among these pathways is tightly regulated and can be influenced by nutritional status, inflammation, and stress.

Under inflammation, the tryptophan catabolic enzyme, indoleamine 2,3-dioxygenase 1 (IDO1), is induced and that catalyzes the breakdown to Trp to N-formyl kynurenine, which is converted into a more stable metabolite, Kynurenine (KYN), and other downstream bioactive catabolites with profound immunomodulatory effects^49–51^. Thus, IDO1 effectively controls tryptophan catabolism and inflammation and immune response. Under inflammatory conditions, IDO1 is strongly induced by interferon-γ, tumor necrosis factor-α, and lipopolysaccharide stimulation^49–51^. Its activation serves as a negative-feedback mechanism to dampen excessive immune activation and promote tolerance through several classical routes: (1) depletion of local tryptophan pools, which suppresses T-cell proliferation and effector function; (2) generation of kynurenine and downstream metabolites that activate the aryl hydrocarbon receptor (AhR) to induce regulatory pathways; and (3) modulation of redox balance through reactive oxygen species (ROS) generation and antioxidant responses. In parallel, the liver-expressed enzyme tryptophan 2,3-dioxygenase (TDO2) catalyzes the same initial step of tryptophan catabolism under homeostatic conditions and is primarily regulated by glucocorticoids and tryptophan levels^43,51^. Together, IDO1 and TDO2 maintain systemic tryptophan flux and immune-metabolic balance, yet during inflammation or hormonal imbalance, aberrant activation of these enzymes can disrupt hepatic redox and energy homeostasis.

In a recent study, we discovered that SR-BI expression and cholesterol uptakes are downregulated in macrophages under inflammation and this is regulated via IDO1^52^. The inhibition of IDO1 rescues the inflammation associated down regulation of cholesterol uptakes and SR-BI expression in macrophages under inflammation. These observations suggest that IDO1 is potential regulator of inflammation, SR-BI regulation and cholesterol homeostasis.

As SR-BI is a well-known player in reverse cholesterol transport, hepatic cholesterol uptake and is modulated by ovarian hormone estradiol, along with being regulated via IDO1 under inflammation, in the present study, we aim to elucidate the relationship between ovarian hormone deprivation, hepatic inflammation, IDO1, Trp-catabolism and cholesterol homeostasis. Using an estrogen-deficient (ovariectomized) rat model, we investigate the effects of estradiol loss on liver inflammation, oxidative stress, and trp-metabolism. This work seeks to provide mechanistic insight into the increased cardiovascular vulnerability observed in hormonal imbalance and in postmenopausal women and to inform the development of targeted, sex-specific therapeutic strategies.

## Materials and methods

### Animal experiments and estrogen treatment in ovariectomized rats

Experimentally naive, 8–12-week-old female Long-Evans rats, both intact and ovariectomized (OVX), were obtained from Charles River Laboratories (Houston, TX). Aseptic techniques were used for all surgical procedures. Surgical tools were sterilized with chlorhexidine solution and by rinsing in 70% ethanol. Surgical sites were shaved and prepared with Betadine prior to incision. Animals were anesthetized with 3–5% isoflurane or a Ketamine/Xylazine solution. Depth of anesthesia was monitored throughout the surgical procedure and flow rate of isoflurane adjusted as appropriate. If animals appear to have pain or post-surgical complications analgesics will be administered as recommended by the veterinarian. Animals were triple-housed with same-sex cage mates in a temperature/humidity-controlled environment under a 12 h reversed light/dark cycle with lights on at 7 p.m. and off at 7 a.m. All rats had free access to food and water throughout the study and were maintained and cared for in accordance with the NIH Guide for the Care and Use of Laboratory Animals. All animal procedures took place at the University of Texas at Arlington and were approved by the University’s IACUC. All methods are reported in accordance with ARRIVE guidelines.

OVX rats were randomly assigned to one of two treatment groups: an estradiol-treated group (OVX + E2; *n* = 6) and a vehicle control group (OVX + V; *n*= 6)^53^. To confirm the success of ovariectomy and assess normal estrous cycling in intact females, vaginal lavage followed by cytology was performed. All animals were allowed a four-day acclimation period before any treatment commenced. Following acclimation, OVX rats received subcutaneous injections of estradiol benzoate (EB) every fourth day to mimic natural fluctuations in estrogen levels. The OVX + E2 group received 2 µg of estradiol benzoate dissolved in 0.1 mL of peanut oil, while the OVX + V group received an equivalent volume of peanut oil alone (0.1 mL per 100 g of body weight). This dosing regimen has been previously used^53–55^. Injections were administered consistently between 8:30 and 9:00 a.m. Body weight was recorded daily for 22 consecutive days beginning on the first day of the experimental period. Measurements were taken at the same time each day under consistent conditions using a calibrated digital scale, with animals weighed prior to feeding to minimize variability. After completing 10 estradiol injection cycles and following a three-day washout period after the final injection (total 43 days), the animals were sacrificed via decapitation. Blood, brain, and body tissues were collected immediately, flash-frozen in liquid nitrogen, and stored at − 80 °C for later analysis.

### HDL, LDL and total cholesterol measurement assay

Plasma samples were analyzed for cholesterol concentrations in High-Density Lipoprotein (HDL) and Low-Density (LDL)/Very-Low-Density (VLDL) Lipoproteins using a EnzyChrom^™^ HDL and LDL/VLDL Assay Kit (EHDL-100)^56^. Briefly, 20 µL plasma was transferred into a 1.5-mL centrifuge tube, followed by addition of 20 µL Precipitation Reagent. Upon mixing and centrifugation for 5 min at 9,500 RPM, 24 µL supernatant was carefully transferred into a clean tube. 96 µL assay buffer was added the tube was labelled as “HDL”. All remaining supernatant was removed and 40 µL PBS was used to resuspend the pellet. 24 µL of this mixture was transferred into another clean tube and 96 µL assay buffer was added. This tube was labelled as “LDL/VLDL”. In a third tube, 12 µL plasma sample was mixed with 108 µL assay buffer and labelled as “Total”. To prepare the cholesterol standard, 12 µL 300 mg/dL cholesterol was mixed with 108 µL assay buffer and the tube was labelled as “Standard”. 50 µL Assay Buffer (“Blank”), 50 µL Standard, 50 µL “Total”, 50µL “HDL” and 50 µL “LDL/VLDL” were transferred into wells of a clear bottom 96-well plate. For each reaction well, 50 µL Assay Buffer, 18 µL NAD Solution and 1 µL Enzyme Mix were mixed. 60 µL of the Working Reagent was transferred to each reaction well. The absorbance of the formed NADH was measured at 340 nm, which is directly proportionate to the cholesterol concentration in the sample. Cholesterol concentrations in the Total, HDL and (LDL/VLDL) fractions are calculated as follows,

$$\:\left[Total\right]\:=\:OD_{TOTAL}\:-\:OD_{BLANK}\:/\:OD_{STANDARD}\:-\:OD_{BLANK}\:\times\:300\:(mg/dL)$$

$$\:\left[HDL\right]\:=\:OD_{HDL}\:-\:OD_{BLANK}\:/\:OD_{STANDARD}\:-\:OD_{BLANK}\:\times\:300\:(mg/dL)$$

$$\:[LDL/VLDL]\:=\:OD_{LDL/VLDL}\:-\:OD_{BLANK}\:/\:OD_{STANDARD}\:-\:OD_{BLANK}\:\times\:300\:(mg/dL)$$

Plasma samples were run in duplicate according to the standard procedure.

### Tissue processing for RNA and protein extraction

The liver tissue was homogenized using a tissue homogenizer in ice-cold, sterile 1X PBS, 3000–5,000 rpm for 30–60 s on ice, with 3–4 short bursts. No visible tissue chunk should remain. After homogenization, the mixture was centrifuged at 1500 rpm for 10 min at 4 °C, separating the tissue, which was subsequently utilized for both RNA and protein extraction. For RNA extraction, the resultant pellet from the centrifugation was resuspended in 500 µL of TRIzol reagent (Invitrogen) and processed further for subsequent experiments. For protein extraction, the previously obtained homogenate was resuspended in 300 µL of RIPA buffer (ThermoFisher) supplemented with 6 µL of 50X protease inhibitor (ThermoFisher). This mixture was sonicated under controlled conditions and was centrifuged. The clarified supernatant, containing the proteins, was carefully harvested, and stored at − 80 °C for downstream analyses.

### RNA extraction, cDNA synthesis, and RT-qPCR

Total RNA was extracted from liver tissue homogenized in TRIzol™ Reagent (Invitrogen), following the manufacturer’s protocol. Briefly, 100 µL of chloroform was added to each lysate, mixed thoroughly, and incubated on ice for 15 min^57^. Phase separation was achieved by centrifugation at 12,000 rpm for 15 min at 4 °C. The upper aqueous phase was carefully collected, mixed with an equal volume of isopropanol, incubated at room temperature for 10 min, and centrifuged at 11,000 rpm for 10 min at 4 °C. The resulting RNA pellet was washed with 70% ethanol (ice-cold), air-dried, and dissolved in 50 µL of DEPC-treated, RNase-free water (Sigma). RNA concentration and purity were assessed using a Nanodrop spectrophotometer.

cDNA was synthesized in a two-step reverse transcription process. In the first step (RT-1), 1 µg of total RNA was mixed with 0.6 µL of oligo(dT)₁₅ primer (500 µg/mL, Promega) and RNase-free water to a final volume of 12 µL. This mixture was incubated at 70 °C for 15 min to denature secondary RNA structures. In the second step (RT-2), a 13 µL master mix was prepared containing 5 µL of 5× M-MLV RT buffer, 2 µL of DTT (10 mM), 0.25 µL of dNTP mix (40 mM, Promega), 0.25 µL of RNase inhibitor (40 U/µL, Promega), and 0.5 µL of M-MLV Reverse Transcriptase (200 U/µL, Promega), with the remaining volume adjusted using nuclease-free water. The RT2 mix was added to the RT1 mixture (final volume: 25 µL), and the reaction was carried out in a thermal cycler: 90 min at 37 °C, followed by 5 min at 95 °C, and held at 4 °C. The resulting cDNA was diluted to a final volume of 100 µL with nuclease-free water for downstream applications.

RT-qPCR was performed using the CFX96 Real-Time PCR Detection System (Bio-Rad) with iTaq™ Universal SYBR^®^ Green Supermix (Bio-Rad). Each 10 µL reaction contained 2 µL of diluted cDNA, 1 µL of forward and reverse primers (final concentration 0.5 µM each) (see Table 1 for list of primers, r and h represent rat and human, respectively), 3 µL of SYBR Green Supermix, and 4 µL of nuclease-free water. Thermal cycling conditions were as follows: initial denaturation at 95 °C for 2 min, followed by 39 cycles of 95 °C for 5 s and 58 °C for 30 s for annealing and extension. Fluorescence thresholds (RFU) were determined using Bio-Rad CFX96 software, and gene expression levels were normalized to β-Actin as an internal control using the 2^(−ΔCt)^ method. Each qPCR reaction was performed in technical triplicates, and the entire procedure was repeated three times to ensure reproducibility.

**Table 1:** Primer sequences Forward (5′−3′) Reverse (5′−3′).

Gene	Forward (5’→3’)	Reverse (5’→3’)
rGAPDH	TCCCAGAAGACTGTGGATGG	AGCATTGGGGGTAGGAACAC
rIL6	AGTGGCTAAGGACCAAGACC	TAGCACACTAGGTTTGCCGAG
rTNFα	ACAGCAATGGTCGGGACATA	CTGAGAGACCTGACTTGGCA
rTDO2	TGTGATGCGCCTATGTTCGT	CCGCTGTGAATGGTACCGATG
rIDO1	GCTCCTAGACCAGCAGGATG	GGGCCTGTTTACGTACTGGA
rSR-BI	CAAGAAGCCAAGCTGTAGGG	GTTGTCCGCTGAGAGAGTCC
rERα	ACCGGTTCATCATGTCCATA	TAAAGCTGTCTCCGCTCGCT
hβ-actin	CTCTTCCAGCCTTCCTTCCT	AGCACTGTGTTGGCGTACAG
hIL-1β	AAGGCGGCCAGGATATAACT	CCCTAGGGATTGAGTCCACA
hIL6	GAAAGCAGCAAAGAGGCACT	TTTCACCAGGCAAGTCTCCT
hIDO1	TCAGTGCCTCCAGTTCCTTT	CCTGAGGAGCTACCATCTGC
hSR-BI	GGCTGAGCAAGGTTGACTTC	AGAACTCCAGCGAGGACTCA
hERα	AGCACCCTGAAGTCTCTGGA	GATGTGGGAGAGGATGAGGA

### Whole protein extraction and western blotting

Western blotting was conducted as previously described, with minor modifications^57^. Briefly, cell lysates were sonicated on ice using a cycle of 10 s on and 45 s off at 30% output to ensure efficient disruption. The lysates were then centrifuged at 13,000 rpm for 10 min at 4 °C to pellet debris. Protein concentrations in the resulting supernatants were determined using the BCA Protein Assay Kit (Pierce). For SDS-PAGE, 40 µg of total protein was loaded per lane and separated on polyacrylamide gels, followed by transfer onto nitrocellulose membranes. Membranes were blocked for 1 h in 5% non-fat dry milk prepared in 1× Tris-buffered saline containing 0.1% Tween-20 (TBST). After blocking, membranes were rinsed with TBST and incubated overnight at 4 °C with primary antibodies specific for IL6 (#ab9324, Abcam 1:1000 dil.), IDO1 (#13268-1-AP, Proteintech, 1:2000 dil.), TDO2 (#15880-1-AP, Proteintech, 1:1000 dil), TNFα (#sc-52746 Santa Cruz, 1:1000), SR-BI (#NB400-101, Novus Biologicals, 1:1000 dil), ERα (#sc-8005, Santa Cruz, 1:2000), β-actin (#A2066, Sigma, 1:3000), and Lamin A (#ab26300, abcam, 1:2000). Following incubation, membranes were washed three times with TBST (5 min each) and incubated for 2 h at room temperature with alkaline phosphatase (AP)-conjugated secondary antibodies: goat anti-mouse (ab97020, Abcam 1:5000 dil.) or goat anti-rabbit (#ab6722, Abcam 1:3000 dil.), depending on the host species of the primary antibodies. After a final series of three washes, protein bands were visualized using BCIP/NBT substrate solution (Promega). Band intensities were quantified using ImageJ software and plotted after normalizing with loading control (β-actin or Lamin A) for data interpretation.

### LC-MS for metabolomic analysis

Whole blood was collected in an EDTA anti-coagulant solution and immediately centrifuged to separate the plasma. Sera from the OVX and OVX + E2 animals were subjected to LC-MS analysis. Metabolites were extracted using a previously described method^58,59^. Briefly, 980 µL of a chilled (− 80 °C) extraction solvent (80% methanol, 20% water) was added to 20 µL of sera (in a microcentrifuge tube), vortexed, incubated at − 80 °C for 2 h, and centrifuged (14,000 RPM, 10 min, 4 °C) to precipitate any proteins present. The supernatants were filtered (0.2- µm filter), transferred to glass HPLC vials, and kept at − 80 °C prior to performing a targeted metabolomic analysis consisting of 203 metabolite references at the UT Southwestern Medical Center Metabolomics Core Facility (Sciex QTRAP 6500 + mass spectrometer). The LC-MS results from the experimental sample analysis were correlated for the the levels of all targeted metabolites and a collective heatmap was generated. Affected pathway analysis was also achieved using this data. The experiments were performed in three parallel replicates (*n* = 3). Raw metabolomic data is provided in the supplementary information S1.

### Nitric oxide assay

Nitric oxide (NO) levels were estimated indirectly by measuring nitrite (NO₂⁻) concentrations using the Griess reagent assay^52,57^. Plasma was isolated from whole blood and immediately transferred to clean Eppendorf tubes for analysis. Since nitric oxide is unstable and rapidly oxidizes to form nitrite and nitrate, this assay quantifies nitrite as an indicator of NO production. To prepare the Griess reagent, equal volumes of two solutions were mixed: (1) sulfanilamide (0.1 g dissolved in 100 mL of 5% phosphoric acid) and (2) N-(1-naphthyl)ethylenediamine dihydrochloride (NED) solution. For each sample, 100 µL of plasma was added to a 96-well plate, followed by 100 µL of the freshly prepared reagent mixture. Absorbance was measured immediately at 540 nm using a microplate reader. A standard curve was generated using known concentrations of sodium nitrite, and all measurements were normalized using deionized water as a blank control.

### Kynurenine assay

Plasma samples were analyzed for kynurenine content using a colorimetric method based on previously described protocol^52,60^. A portion of each plasma sample was mixed with 30% trichloroacetic acid at a 4:3 ratio and incubated at 45 °C for 30 min to facilitate protein precipitation and hydrolysis. Following incubation, the samples were centrifuged at 10,000 rpm for 5 min at room temperature. From the resulting supernatant, 200 µL was transferred to a 96-well plate. Each well received 100 µL of Ehrlich’s reagent, prepared by mixing p-dimethylaminobenzaldehyde with 95% ethanol and glacial acetic acid in a 1:1 ratio. Samples were analyzed in triplicate. After a 10-minute incubation at 37 °C, absorbance was measured at 492 nm using a microplate reader. A standard curve was generated using known concentrations of L-kynurenine (Sigma), and results were normalized against blank prior to data analysis.

### Lactate detection assay

Lactate levels in plasma samples were measured using a previously described method^61^. Before adding 5 µL of plasma in a 96 well plate, a reaction mixture containing 5 U/mL of Lactate dehydrogenase (LDH-A) enzyme dissolved in 0.1 M phosphate buffer (pH 6.5), 200 µM NAD + and 50 µM Hydrazine Hydrate was prepared. From a total reaction volume of 500 µL, 200 µL was dispensed in each well and absorbance was obtained at 340 nm. Briefly, this assay measures the production of NADH as a reaction of LDH-A that converts the lactate to pyruvate. Different concentrations of Lactate (Stock: 100 mM in water) standards were used with each set of reactions. The experiments were repeated thrice to achieve highest statistical significance.

### THP1 differentiation and treatment with LPS and estradiol

The human monocytic cell line THP-1 (ATCC) was cultured in complete RPMI-1640 medium at 37 °C in a humidified incubator with 5% CO₂, as previously described (Chini et al., 2025)^52^. For macrophage differentiation, 25 nM phorbol 12-myristate 13-acetate (PMA; DMSO stock) was used. Differentiated cells (THP1-MΦ) were given 24 h of charcoal-stripped RPMI followed by 1 nM Estradiol (Sigma E2758) for 6 h and stimulated with 1 µg/mL lipopolysaccharide (LPS; Sigma) for either 4 h for RNA analysis, 18 h for protein analysis and kyn assay, or 36 h for NO assay and cells and culture supernatants were subsequently collected.

### Kupffer cell isolation and treatment with estradiol

Kupffer cells (KCs) were isolated as described previously^55^. Livers were harvested from Long Evans wild-type (WT) rats immediately after perfusion and transferred into pre-warmed ex vivo digestion medium consisting of RPMI supplemented with 1% FBS, 2 mM CaCl_2_, 0.8 mg/mL collagenase, and a DNase inhibitor (100 U/mL). Liver tissues were incubated at 37 °C for 30 min and intermittently vortexed at 2000 rpm for 10 min to facilitate digestion. The digested tissue was gently homogenized 5–6 times and filtered through a 40 μm cell strainer into a 50 mL conical tube. The filtrate was centrifuged at 5000 rpm for 3–4 min. The aqueous phase was collected and transferred to a new tube, while pellets containing dead hepatocytes and debris were discarded. The suspension was then centrifuged at 10,000 rpm for 5 min to pellet liver non-parenchymal cells (LNPCs). The supernatant was discarded, and red blood cells were lysed by resuspending the pellet in 3 mL ACK lysis buffer and incubating for 30 s at room temperature. Osmolarity was restored by adding 20 mL plain RPMI, followed by centrifugation at 10,000 rpm for 5 min. The resulting cell pellet was resuspended in 10 mL ice-cold complete RPMI. Cell viability was determined by mixing an aliquot of the suspension 1:2 with trypan blue and counting viable cells using a hemocytometer. Complete culture medium consisted of RPMI supplemented with 10% FBS and 100 U/mL penicillin/streptomycin. LNPCs were resuspended at a density of 1–3 × 10⁷ cells/mL, and 1 × 10⁷ cells were plated per well in 6-well plates. Cells were incubated for 2 h at 37 °C in a humidified 5% CO₂ incubator to allow Kupffer cell adherence. Non-adherent cells and debris were removed by gently washing with cold PBS. To maintain Kupffer cell differentiation, recombinant macrophage colony-stimulating factor (M-CSF, 50 ng/mL) was added to the culture medium.

To further characterize Kupffer cells, cell identity was confirmed by CD14 immunofluorescence staining (Fig5A). Cells were seeded onto sterile poly-D-lysine–coated coverslips (A3890401, Thermo Fisher Scientific) and allowed to adhere for 4 h. Cells were then fixed with 4% paraformaldehyde (PFA) for 10 min at room temperature, followed by three washes with 1× PBST (5 min each). Cells were permeabilized with 0.1% Triton X-100 in 1× PBST for 15 min at room temperature, followed by blocking with 3% bovine plasma albumin (BSA) in 1× PBST for 1 h to reduce nonspecific binding. Coverslips were incubated with a mouse anti-human CD14 primary antibody (#14–0149-82, Invitrogen) for 4 h at room temperature. After three washes with PBST, cells were incubated with FITC-conjugated goat anti-mouse secondary antibody (#6785, Abcam) for 1 h at room temperature in the dark. Following secondary antibody incubation, nuclei were stained with DAPI (1 µg/mL) for 15 min. Coverslips were washed three additional times with PBST and mounted onto glass slides using mounting medium. Fluorescence images were acquired using a Nikon ECLIPSE TE2000-U fluorescence microscope for analysis. For inflammatory stimulation, KCs cells were treated with E2 for 6 h followed by stimulation with LPS for 4 h for RNA isolation and RT-qPCR. Kynurenine levels were measured in the cell culture supernatant (6 h of E2, 18 h LPS).

**Fig. 1:**
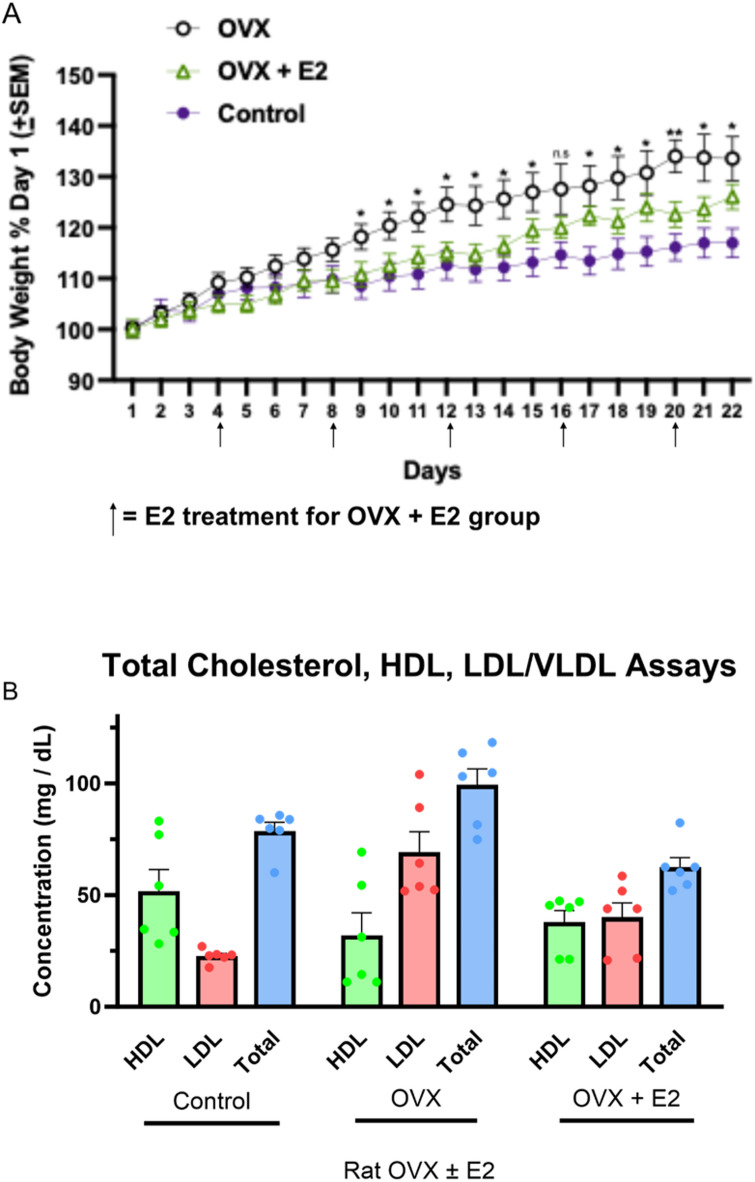
Estrogen deprivation increases body weight and plasma cholesterol levels in female rats. Female Long-Evans rats (intact, OVX, and OVX + E2, *n* = 6) were acclimated for four days. OVX + E2 rats received subcutaneous injections of 2 µg estradiol in 0.1 mL peanut oil every fourth day for 10 cycles; OVX rats received vehicle only. Body weight was monitored throughout the treatment period before sacrifice (panel A). Plasma HDL, LDL/VLDL, and total cholesterol were quantified using the EnzyChrom™ HDL and LDL/VLDL Assay Kit (EHDL-100). Plasma fractions were separated with precipitation reagent, and NADH absorbance was measured at 340 nm to calculate cholesterol concentrations (panel B). (*n* = 3, *p* ≤ 0.001).

**Fig. 2:**
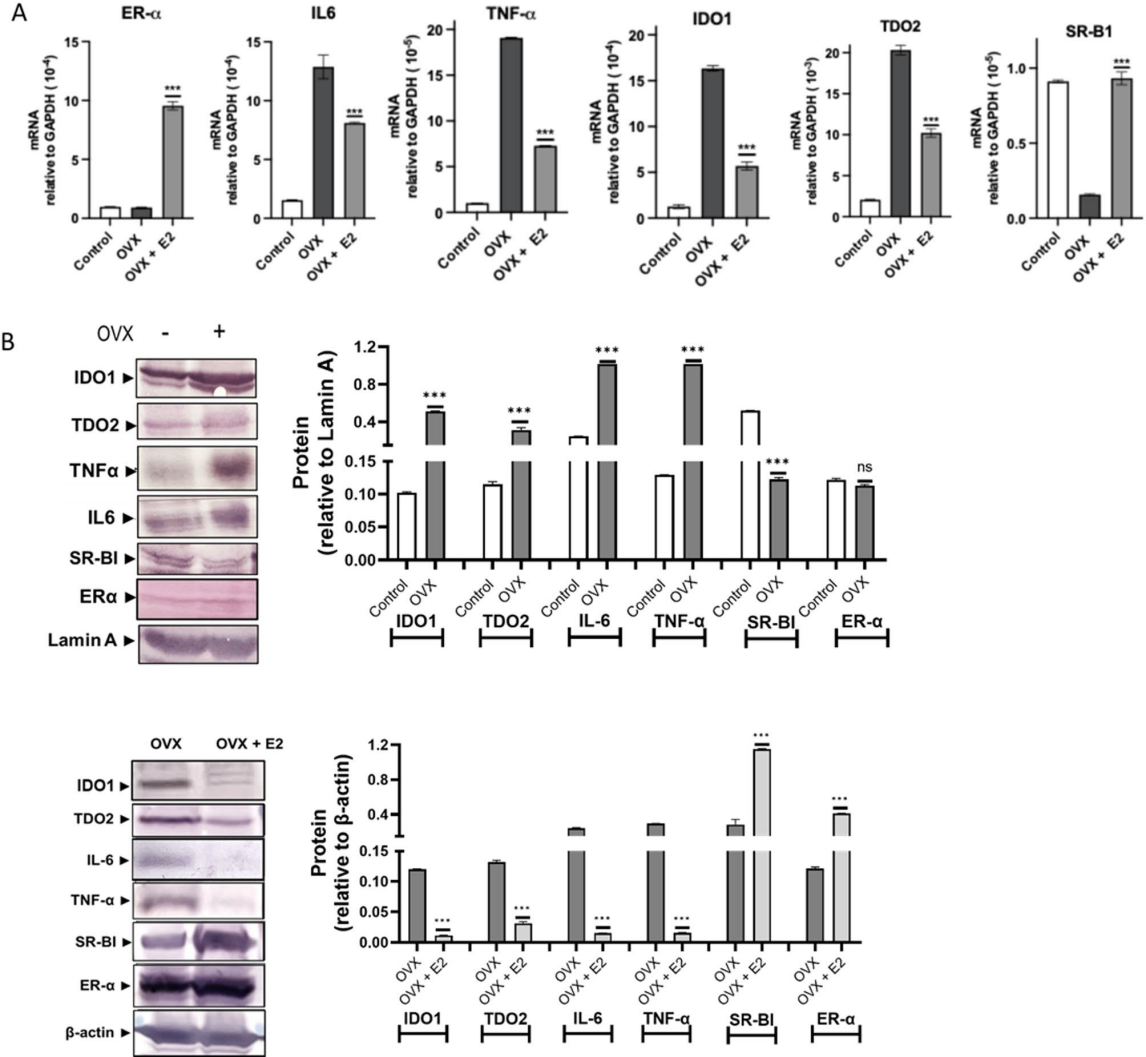
Ovariectomy (OVX) induced hepatic inflammation (in female rats) are rescued upon estrogen supplementation. Intact, OVX and OVX + E2 rat livers were homogenized and used for RNA and protein analyses. Total liver RNA was analyzed by RT-qPCR using primers against ERα, IL6, TNFα, IDO1, TDO2, SR-BI and the housekeeping gene, GAPDH (panel A). Protein (cell lysate) levels of ERα, IL6, TNFα, IDO1, TDO2, SR-BI and the housekeeping gene, β-actin were analyzed (panel B, quantifications are in right). (*n* = 3, *p* ≤ 0.001).

**Fig. 3:**
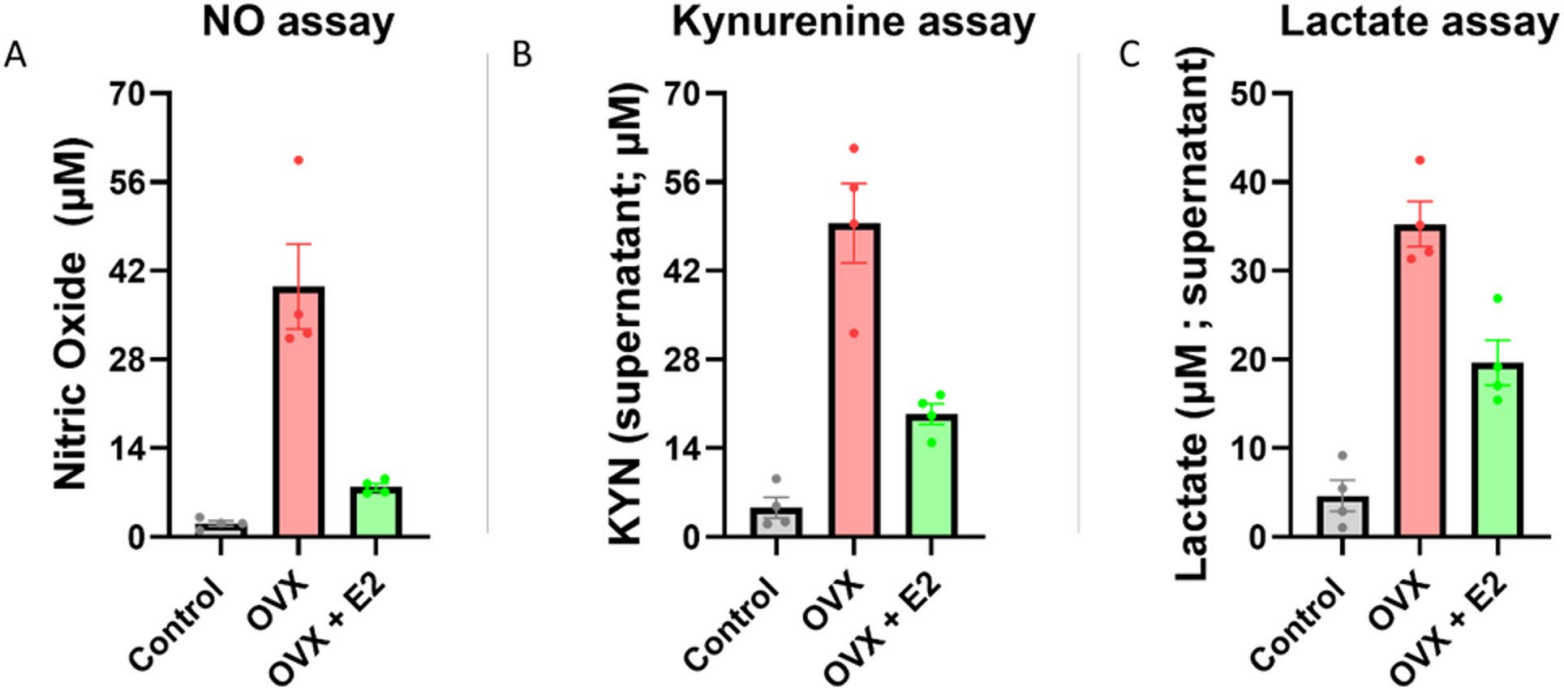
Estrogen supplementation reduces nitric oxide, kynurenine and lactate levels in OVX rats. OVX and OVX + E2 rat sera were analyzed for nitric oxide, kynurenine and lactate levels. Briefly, nitric oxide (NO) production was quantified indirectly by measuring nitrite (NO₂⁻) using the Griess reagent assay. Equal volumes of sulfanilamide (0.1 g in 100 mL of 5% phosphoric acid) and N-(1-naphthyl)ethylenediamine dihydrochloride were mixed, and 100 µL of plasma was reacted with 100 µL reagent in a 96-well plate. Absorbance was read at 540 nm and compared with a sodium nitrite standard curve (panel A). Additionally, kynurenine levels were measured colorimetrically by reacting trichloroacetic acid–treated plasma supernatants with Ehrlich’s reagent (p-dimethylaminobenzaldehyde in ethanol and acetic acid, 1:1). Absorbance was recorded at 492 nm after 10 min at 37 °C using L-kynurenine standards (panel B). Lactate concentration was determined by monitoring NADH formation at 340 nm in reactions containing plasma, 5 U/mL LDH-A, 200 µM NAD⁺, and 50 µM hydrazine hydrate in phosphate buffer (pH 6.5) (panel C). All assays were performed in triplicate, and results were normalized to corresponding blank and standard controls.

**Fig. 4:**
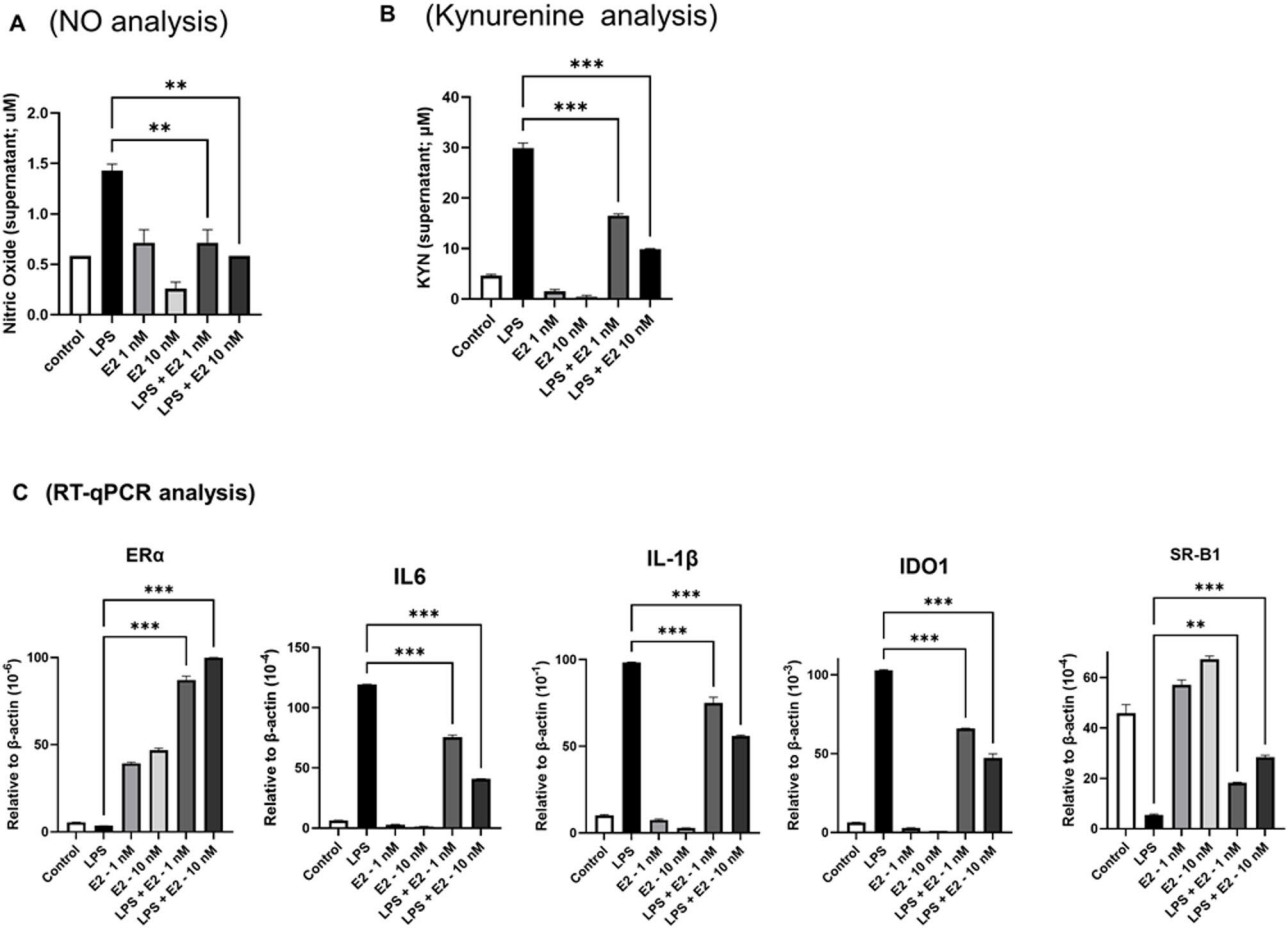
Estrogen supplementation reduces LPS induced inflammation, Trp catabolism and restores SR-BI expression in THP-1 derived macrophages. THP-1 monocyte cells were differentiated into macrophages using PMA, followed by incubation in charcoal stripped RPMI (24 h) and treatment with E2 (6 h) and LPS (4 h for RNA analysis, 18 h for kyn analysis, and 36 h for NO levels). Supernatant was measured colorimetrically for NO level using Griess assay (panel A), and Kyn level (panel B). RNA was analyzed by RT-qPCR using primers against ERα, IL6, IL1β, IDO1, SR-BI, and the housekeeping gene, β-actin (panel C).

**Fig. 5:**
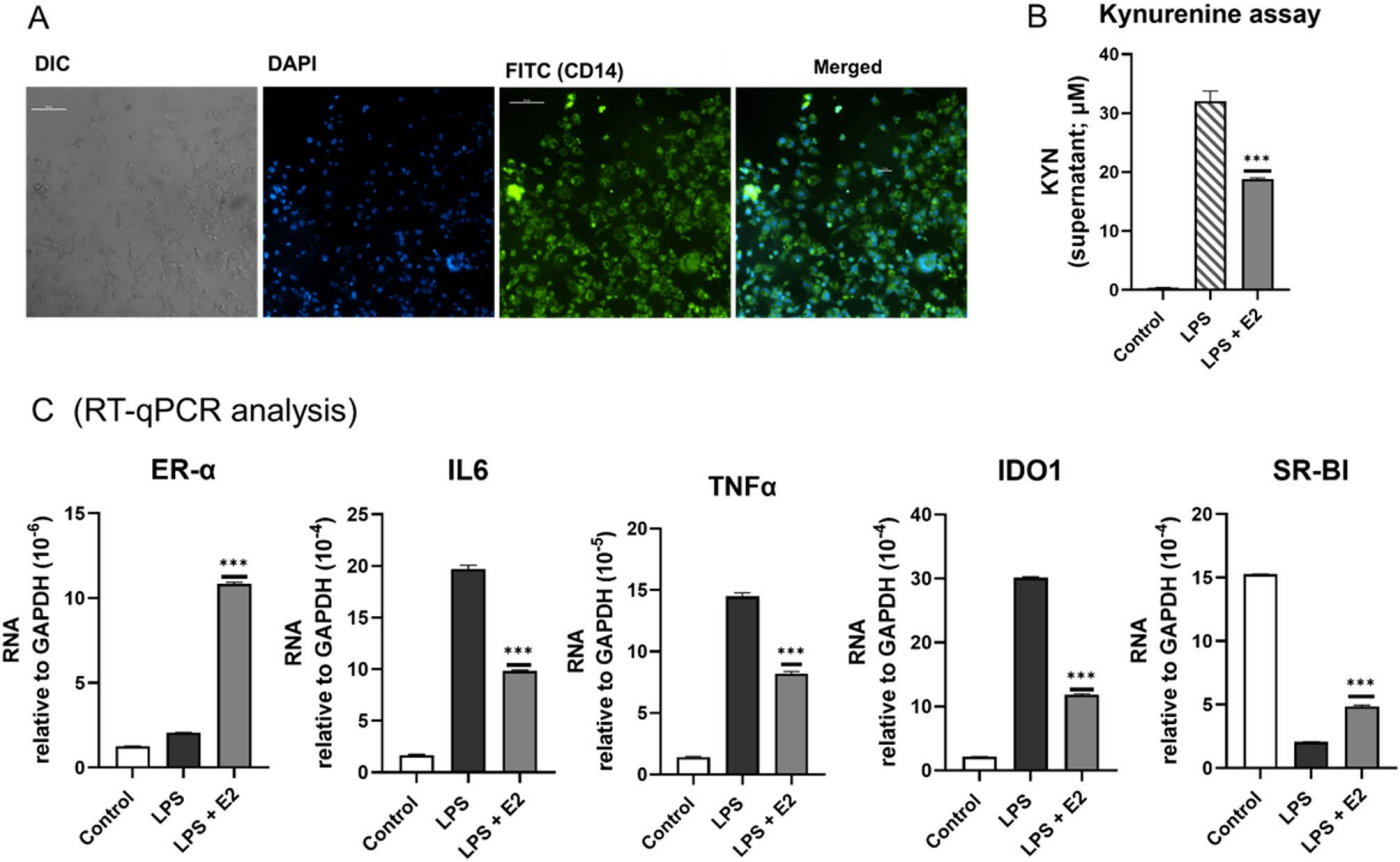
E2 Modulates LPS-Induced Cytokine, IDO1, and SR-BI Expression in Rat Primary Kupffer Cells. Primary Kupffer cells from rat liver were isolated using ex vivo digestion method, followed by LNPC isolation and differentiation. The population was confirmed by CD14 staining (panel A). Once confirmed KCs were treated with E2 (6 h) and followed by LPS (18 h), kynurenine level was measured in cell supernatant (panel B). For the RNA analysis, KCs were also treated E2 (6 h) and followed by LPS (4 h). RNA was analyzed by RT-qPCR using primers against IL6, TNFα, IDO1 and SR-BI, and the housekeeping gene, GAPDH (panel C). (*n* = 3, *p* ≤ 0.001).

### Statistical analysis

Unless otherwise mentioned, all experiments were conducted in triplicate, with a minimum of three independent samples per condition (*n* = 3). The data has been presented as mean values with standard error of the mean (SEM). Statistical analyses were performed using one-way ANOVA with Bonferroni’s post-hoc correction (GraphPad Prism). *P* ≤ 0.05 was deemed to indicate statistical significance. Body weight data statistical analysis was performed using a two-way analysis of variance (ANOVA) with treatment group and time as factors. When a significant interaction or main effect was detected, post hoc comparisons were conducted using Dunnett’s multiple comparisons test to compare each time point of the OVX group to the corresponding intact control group. A p-value ≤ 0.05 was considered statistically significant.

## Results

### Ovariectomy resulted in increased body weight, elevated plasma cholesterol level, and this is reversed by estradiol (EB) in female rats

To understand the role of hormones in lipid metabolism and homeostasis, we have used ovariectomized Long-Evan’s rats devoid of internal sources of ovarian hormone and monitored the overall body weight and plasma lipoproteins (LDL and HDL) and total cholesterol levels. To further understand the impacts of estrogen, we also administered estradiol (E2)-benzoate subcutaneously, every 4 days intervals to OVX rats followed by analysis of overall body weight^62,63^. Notably, all the animals (intact, OVX, and OVX+E2) maintained under similar diet and other conditions. Our analysis showed that both control and OVX female rats gained more weight with age, however, the OVX females have gained significantly higher body weight compared to intact female rats (Fig. 1A). A statistically significant increase in body weight was observed in OVX rats beginning on day 9 compared to intact control animals (*p* < 0.05) (Fig. 1A). Interestingly, treatment with estradiol (OVX+E2) resulted in reduced body weight in compared to OVX animals, though the OVX + E2 group of animal has higher body weight than the intact females. These observations suggest that ovarian hormones play critical roles in regulation of overall body weight in animals, and its reduction leads to decreased metabolism resulting in increased body weight in OVX animals. This is further evidence by the reduction in body weight upon administration of external EB. The higher body weight of the OVX+E2 group of animals compared to intact female, is likely to other factors that may control the metabolism and body homeostasis.

Importantly, body weight is linked to plasma lipid and cholesterol levels and that are associated with the increased risk of CVD^64,65^. To understand further the impact of estrogen on circulating plasma lipids levels, we measured the LDL, HDL and total cholesterol levels in intact, OVX, and OVX+E2 group of animals. Briefly, OVX animals were treated E2-benzoate for 18 days (EB-treatment were performed every 4 days) and then sacrificed. Plasma from animal blood was collected and analyzed for the LDL, HDL, and total cholesterol levels using commercial kits. This analysis revealed that indeed the plasma level of LDL was significantly increased in OVX animals (74 mg/dL) compared to the intact (22 mg/dL) animals and this level was reduced upon treatment with EB (OVX+E2, 38 mg/dL) (Fig. 1B). In contrast, HDL level was significantly reduced in OVX animals (25.5 mg/dL) compared to intact female (50.mg/dL) and E2 administration nominally rescued the HDL level. Total cholesterol level followed the similar pattern of LDL level, increased upon ovariectomy which is reversed by EB-treatment. Taken together observation suggests that ovarian hormone estrogen controls the circulating LDL, HDL, and cholesterol level significantly (Fig. 1B). Increase in LDL and total cholesterol levels and concomitant reduction in HDL levels upon ovariectomy indicate potential increased risk of cardiovascular diseases in females upon estrogen deprivation (such as in postmenopausal conditions and other hormonal imbalance cases).

### Estrogen treatment rescues ovariectomy-induced hepatic inflammation as well as systemic inflammation

Loss of estrogen has been linked to activation of inflammatory pathways, specifically in postmenopausal women where this is prominent with increased emergence of liver disorders and cardiovascular diseases^66–70^. Since hepatic inflammation is a phenomenon widely cited as risk factor for several steatotic liver diseases, dismantled cholesterol homeostasis and enhanced cardiovascular risk, it becomes imperative to understand the role of estrogen loss in regulation of hepatic inflammation. In a recent study, we discovered that human HDLR-SR-BI, a key player in reverse cholesterol transport, is downregulated in macrophages under inflammatory stress resulting in reduced cholesterol uptake by macrophages under inflammation^52^. Additionally, we demonstrated that a tryptophan catabolic enzyme, Indoleamine-2,3-dioxygenase 1 (IDO1), controls the inflammation-induced SR-BI downregulation and reduced cholesterol uptakes by the macrophages. SR-BI is well known to be expressed in liver and controls the selective uptake of HDL-cholesterol ester during reverse cholesterol transport. In a previous study from our laboratory and others demonstrated that SR-BI expression is transcriptionally regulated via estrogen and estrogen deprivation down-regulates SR-BI expression^41^. In the current study, we have employed OVX rats to define the physiological and molecular consequences of estrogen deficiency.

To further elucidate the impact of estrogen deficiency on SR-BI expression and potential link to inflammation response, we examined the level of inflammatory response and SR-BI expression in the absence and presence of estrogen using ovariectomized female rats. Briefly, we have isolated the total RNA and proteins from the control (intact female), OVX and OVX+E2 groups of animals and analyzed by RT-qPCR and Western blotting for expression of various cytokines, SR-BI and ERα. RT-qPCR as well as western blot analysis demonstrated that OVX animal liver has higher level of pro-inflammatory cytokines, IL6 and TNFα, both at the RNA and protein level in comparison to the control female rats, and the level of OVX-induced elevation of IL6 and TNFα was reduced upon treatment with EB [Figures 2A (RT-qPCR analysis) and 2B (western blots, quantification in respective right panels, supplementary figure S1)]. The OVX-associated increase in IL6 and TNFα suggest increased hepatic inflammation in OVX animals due to internal estrogen deprivation, and this is rescued upon estrogen supplementation. The expression of estrogen receptor alpha (ERα), a nuclear receptor that is a well-known estrogen-regulated gene^71,72^, was also significantly elevated upon treatment with EB in the OVX animal liver, as previously observed in other studies^73–75^. To understand further the potential roles of IDO1 and tryptophan catabolism in E2-defficiency induced hepatic inflammation and cholesterol homeostasis, we analyzed the expression of Trp-catabolizing enzymes IDO1 and TDO2, hepatic reverse cholesterol associated gene SR-BI expression in the control, OVX and OVX+E2 animal livers. Interestingly, expressions of IDO1 and TDO2 are elevated in the OVX rat livers in comparison to the intact animals, which were rescued by E2 treatment (Figs. 2A-B). SR-BI, which is a well-known gene regulated via estrogen, is downregulated in the OVX liver, and this was rescued by EB-administration (Figs. 2A – B). The observation suggests that IDO1 as well as TDO2, are upregulated under estrogen deficiency, indicating their potential association with estrogen-deficiency mediated hepatic inflammation, SR-BI expression and cholesterol homeostasis and they were remitted by EB administration. Notably, the apparent discrepancy in expression levels for different proteins such as TNFα and IL6 in the two western blots in panel 2B, are potentially linked to different blots performed using different liver tissue samples at a different time.

As IDO1 and TDO2 are the key enzymes associated with Trp-catabolism, we examined the levels of IDO1-associated Trp-catabolite Kynurenine (KYN) in the intact, OVX and OVX+E2 groups of animal plasma. Interestingly, this analysis indeed demonstrated that there is a significant increase in the KYN level in the OVX animal plasma (49 µM) in comparison the intact female rats (5.6 µM) and that was reversed by EB (Fig. 3A). The elevation in plasma KYN level in OVX animal suggested that there is increased level of systemic inflammation stress upon estrogen deprivation and that were rescued by estrogen supplementation. The rise in KYN level in KYN level is at least in part contributed by the rise in IDO1 and TDO2 in livers and linked to hepatic inflammation.

In addition to the plasma KYN level, we also analyzed the plasma level of nitric oxide (NO), a biomarker for inflammation. Notably, NO is a signaling molecule produced by nitric oxide synthases (NOS), including endothelial (eNOS), neuronal (nNOS), and inducible (iNOS) nitric oxide synthases^76–91^. During inflammation, iNOS is upregulated by cytokines and microbial products, leading to high NO production that helps kill pathogens and regulate immune responses^84–86,89–91^. However, excessive NO can react with reactive oxygen species to form toxic compounds like peroxynitrite, causing tissue damage and chronic inflammation. For example, to mitigate NAFLD or NASH progression, liver eNOS-derived NO from sinusoidal endothelial cells maintains microcirculatory perfusion, mitochondrial health, and reduces inflammation^91–93^. By contrast, iNOS is induced in Kupffer cells and infiltrating macrophages, inducing inflammatory signaling which can accelerate steatohepatitis and fibrogenesis^94,95^. Thus, NO activity is context-dependent and may act as a double-edged sword in terms of inflammation. Briefly, we analyzed the plasma NO level in intact, OVX and OVX+E2 group of animals using Griess assay (Fig. 3B). NO levels were normalized using a standard curve. This analysis demonstrated that there is significant elevation in NO level in the OVX animal plasma in comparison to the intact animals and that were reversed by EB-treatment. These observations further suggest that there is increased systemic inflammation upon estrogen-deprivation in OVX animals as evident by the reversal of NO level in the OVX+E2 animal groups.

Similar to NO level, lactate production is another marker for inflammation. Notably, lactate is produced from pyruvate via lactate dehydrogenase pertaining to a metabolic shift in immune and stromal cells^96–98^. Elevated lactate production and accumulation play a significant role in chronic and hepatic inflammation^96,99^. For instance, in NAFLD and liver fibrosis, increased glycolysis and lactate levels fibrogenesis and inflammatory signaling via receptors like GPR81 and transporters such as MCT4, thus, confirming the role of lactate in chronic liver diseases^100,101^. Therefore, we quantified the lactate levels in plasma of the intact, OVX and OVX + E2 rats. In agreement with KYN and NO level plasma lactate is significantly elevated in the OVX animals compared to control and this was reversed by E2 administration, further indicating systemic inflammation induced by estrogen-deprivation and its mitigation in presence of estrogen (Figs. 3C).

Taken together, our results demonstrated increased hepatic and systemic inflammation, along with elevated Trp-catabolism under estrogen deficiency, which may have potential implications in altered plasma cholesterol contributing to dyslipidemia. Hence, Estrogen plays a critical role in maintaining hepatic health suppressing inflammation and cholesterol lipid transport, metabolism and homeostasis.

### Estradiol (E2) treatment suppressed the LPS-induced inflammation, IDO1 expression, and Trp-catabolism in macrophages

Our results (Figs. 1, 2 and 3) demonstrated that estrogen-deprivation results in elevation of inflammation and Trp-catabolism in female rats and this is suppressed by estradiol (E2), suggesting a key role of estrogen in regulating inflammation under physiological conditions. To understand further the mechanism of inflammation signaling, we investigate the role of estrogen in regulation of inflammatory response in macrophages – THP1 (monocytes) derived macrophages (THP1-Mφ) and also in primary macrophages - liver resident macrophage, Kupffer cells (KCs). THP1 cells (human monocytes) were differentiated into macrophages (THP1-Mφ) using PMA as described previously^52^. THP1-Mφ cells were grown in charcoal-stripped media and then treated with varying concentrations of estradiol (E2) for 6 h and then treated with LPS for additional 4 h. RNA from the control and E2/LPS treated cells were analyzed by RT-qPCR for the expression of various cytokine (IL6, IL-1β), IDO1, and SR-BI expression. Cell supernatants were analyzed for the expression kynurenine (KYN) and nitric oxide (NO) level. These analyses demonstrated that LPS-treatment resulted in elevation of NO as well as Kyn levels in THP1-Mφ, and this was suppressed by E2-treatment in a dose-dependent manner (Figs. 4A and B). Notably, E2-treatment (in the absence of LPS) also quenched the basal level of NO and Kyn level in comparison to the control cells (Figs. 4A and B). LPS-induced elevation in NO and Kyn levels suggested the increased inflammatory response and elevated Trp-catabolism in macrophages, and these were suppressed by E2. RT-qPCR analysis also demonstrated that LPS-treatment induced expression of inflammatory cytokines (IL6, IL-1β) and IDO1 significantly, while SR-BI expression was down-regulated, and all these were reversed by E2-treatment (Fig. 4C). ERα was also increased by treatment with E2 (both in the absence and presence of LPS), indicating effective estrogen treatment response in macrophages. (Fig. 4C). These observations further suggested a crucial role of E2 in regulation of inflammatory response, Trp-catabolism and SR-BI regulation in macrophages.

To further understand the role of E2 in regulation of hepatic inflammations, we isolated the liver resident primary macrophages, Kupffer cells (KCs) from liver from wild type Long-Evans rats, treated with LPS, in the absence and presence of E2 followed by analysis of inflammatory response, Trp-catabolism, and SR-BI expression. KCs were isolated from rat livers using a previously reported procedure^102^ and confirmed by immunostaining CD14, a surface marker found on monocytes and macrophages. Strong CD14 staining was observed in most of the adherent cells, indicating successful enrichment of macrophages. DAPI staining confirmed intact nuclei and normal cell morphology. Together, these results suggest that the isolated and cultured adherent LNPC population mainly consisted of Kupffer cells (Fig. 5A). These primary KCs were cultured in flasks in charcoal stripped plasma containing media, treated with E2 (1 nM 6 h) followed by stimulation with LPS (1 µg/mL, 4 h). RNA was analyzed by qPCR for the expression of various cytokines, Trp-catabolic enzymes, SR-BI, and ERα. Cell culture supernatants from the control and LPS/E2-treated cells were analyzed for the secreted Kyn level. Interestingly, LPS treatment significantly induced the Kyn levels in cell supernatants compared to the untreated control (Fig. 5B). E2-treatment significantly suppressed the LPS-induced elevation of Kyn level (Fig. 5B). RT-qPCR analysis of the mRNAs showed that that LPS-treatment resulted in significant induction of pro-inflammatory cytokines (IL6, TNFα) along with IDO1and TDO2, while the SR-BI expression was downregulated (Fig. 5C) and these were reversed by E2-treatment. These observations further demonstrate that E2 plays critical roles in suppressing inflammatory response and Trp-catabolism in primary Kupffer cells and hence potentially associated with the regulation of hepatic inflammation via regulation of Kupffer cell activation.

### Estrogen-deprivation reprograms Trp-catabolism and glucose metabolism leading to inflammatory response (a mass spectrometry based metabolomic analysis)

**Fig. 6:**
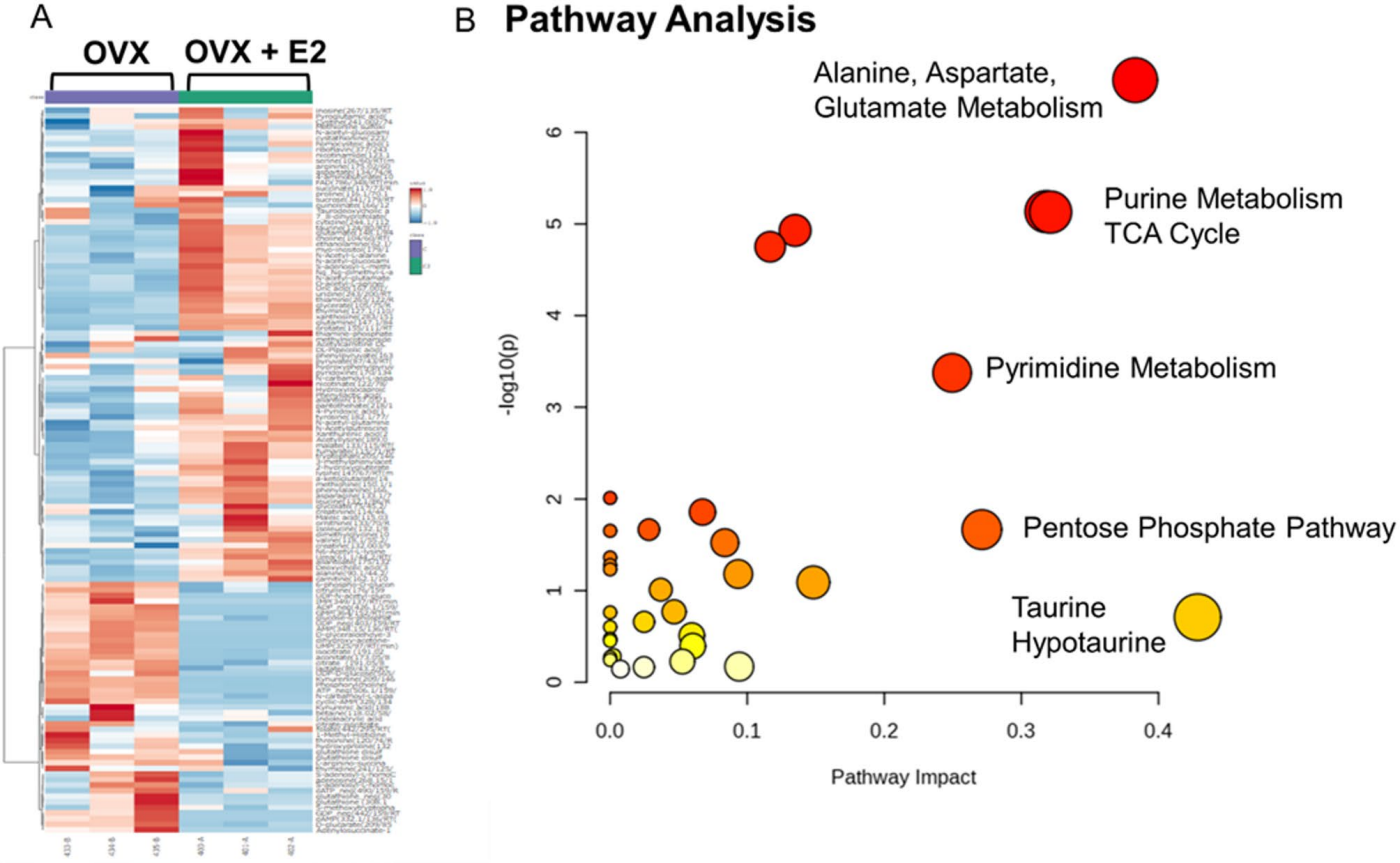
LC-MS analysis of metabolites in OVX and OVX + E2 rat sera. For targeted metabolomic analysis, whole blood sera from the OVX and OVX + E2 animals were subjected to LC-MS. Briefly, 980 µL of a chilled extraction solvent (80% methanol, 20% water) was added to 20 µL of sera and incubated at − 80 °C for 2 h. Upon centrifugation, the supernatants were filtered and a targeted metabolomic analysis consisting of 203 metabolite references was performed using Sciex QTRAP 6500 + mass spectrometer. The LC-MS results are presented in a collective heatmap (panel A). Affected pathway analysis was also achieved using this data (panel B). (*n* = 3, *p* ≤ 0.001).

**Fig. 7:**
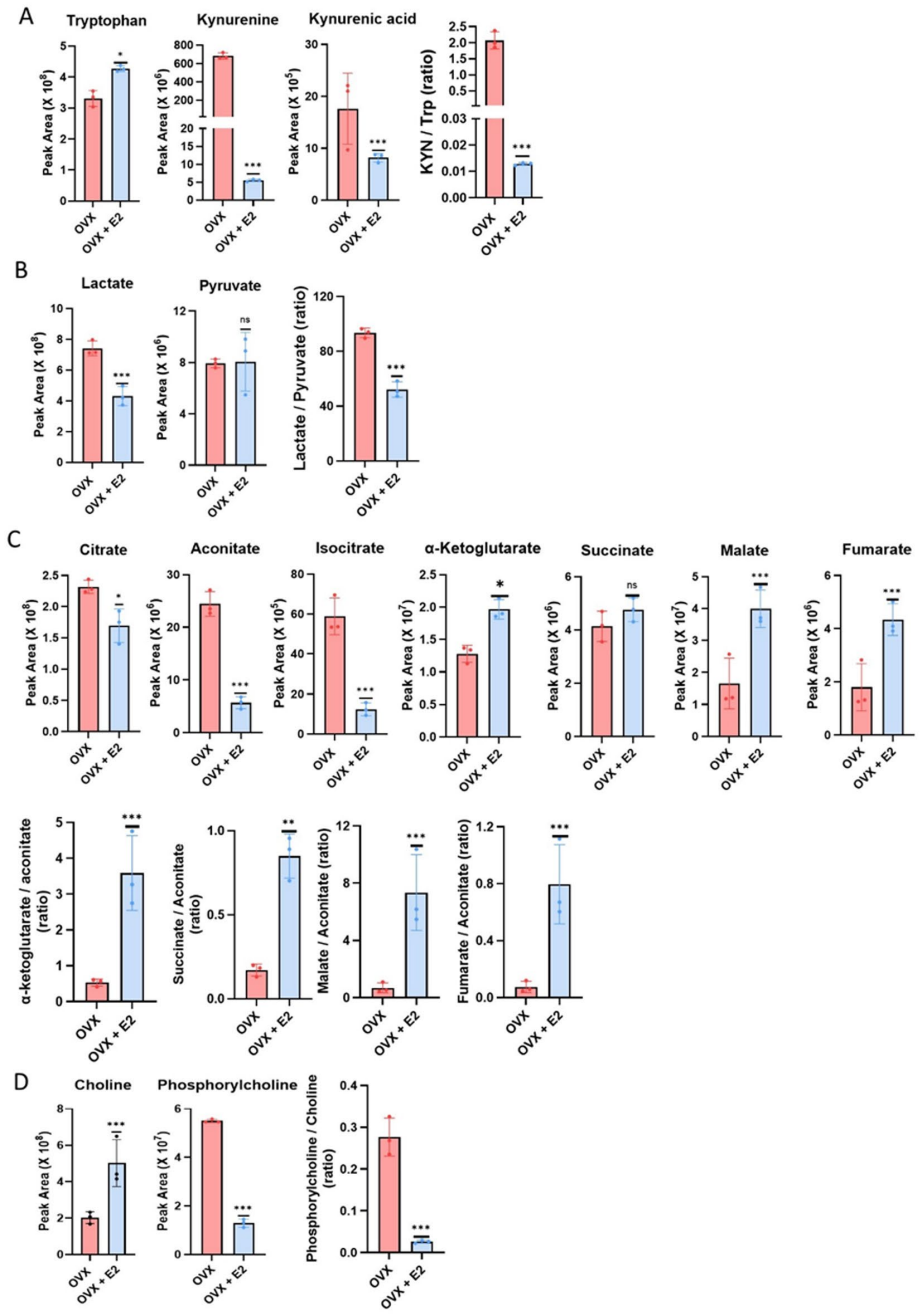
Changes in metabolite (selected) levels in OVX and E2-treated OVX rat blood. To obtain the comparison between certain metabolite levels in OVX and OVX + E2 samples, the normalized peak area is plotted. Panel A represents the tryptophan metabolites along with KYN/Trp ratio. Panel B represents the effect on lactate levels. Panel C is the TCA cycle metabolite and the diverse effects on TCA cycle metabolites represented by selected metabolite ratios. The choline pathway is presented in panel D. (*n* = 3, *p* ≤ 0.001).

To further understand the role of estrogen in regulation of inflammation and Trp-catabolism, we performed targeted metabolomic analysis in plasma samples of OVX rats in the absence and presence of estradiol (E2)-benzoate. Briefly, the Trp-associated metabolites as well other metabolites were extracted from OVX and OVX+E2 animal plasma using 80% methanol (20% water) and subjected to a targeted metabolomic analysis by LC-MS (UT Southwestern medical center metabolomics core facility). The peak area representing each metabolite was compared for OVX and OVX+E2 plasma were used for comparison. A heat map showing the level of changes of different metabolites and pathway affected in OVX and OVX+E2 samples are shown in Figs. 6A and B (also see supplemental information S2). Interestingly, along with various other metabolites the levels of tryptophan catabolite, plasma levels of Kynurenine and Kynurenic acids levels are significantly reduced in EB-treated OVX animals in comparison to the OVX animals. The Trp/KYN ratio is significantly increased in OVX+E2 in comparison to the OVX control animal. These observations demonstrated that there are increased Trp-catabolism in OVX animals, and this is reduced upon treatment with EB (Fig. 7A). Metabolomic analysis also showed that the level of lactate as well as the lactate to pyruvate ratio are significantly high (2.5-fold) in the OVX plasma compared to the EB-treated OVX rats (Fig. 7B). Increased lactate production is a hallmark of inflammation, suggesting increased systemic inflammation induced upon estrogen deprivation and this inflammation level is alleviated under estrogen supplementation.

Along with lactate, the level of TCA cycle activity is known to be reduced/bypassed under inflammation stress. Interestingly, our metabolomic analysis revealed that TCA cycle associated metabolites such as citrate, isocitrate and aconitate levels are significantly high in the OVX animal plasma compared to OVX+E2 plasma (Figs. 7C). Interestingly the level of downstream TCA cycle metabolites such as alpha-ketoglutarate (α-KG) and malate were detected, and their levels remained mostly unaffected under OVX and OVX+E2 treatment conditions. Interestingly, ratio of alpha-KG/aconitate is in the 0.6s in the OVX animal and that is increased to 3.6s under estrogen treatment (Fig. 7C), suggesting the reduced conversion of aconitate to alpha-KG under OVX condition (Figs. 7C). Notably, conversion of aconitate to itaconate via aconitate decarboxylase (ACOD1) that activates downstream KEAP1/NRF2 signaling and inflammatory response pathway are well known under inflammatory conditions^103,104^. Thus, the low ratio of alpha-KG/aconitate is in the 0.6 under OVX condition is likely suggesting the conversion of aconitate into other pathway and bypassing the TCA cycle. This is further reflected in the low malate/aconitate ratio in OVX rats and OVX + E2 (Figs. 7C). These observations suggest the critical roles of estrogen in normal metabolism function for glucose metabolism and energy homeostasis and that estrogen-deprivation induced systemic inflammation which is associated with increased glycolysis, increased lactate production and reduced TCA cycle activity and these effects were revered upon estrogen supplementation.

Along this line our analysis also demonstrated the increase in phosphorylcholine on OVX plasma compared OVX+E2 treated animal (Fig. 7D). Notably, phosphorylcholine-containing phospholipids are recognized as pro-inflammatory intermediates that can activate pattern recognition receptors such as Toll-like receptor 2 (TLR2) and TLR4, leading to cytokine release and complement activation^105–108^. Elevated phosphorylcholine levels are also indicative of membrane lipid remodeling and oxidative phospholipid turnover, processes commonly associated with hepatic inflammation and endothelial dysfunction under estrogen-deficient conditions.

Pathway analysis demonstrated that OVX associated highly affected pathways include alanine, aspartate and glutamate metabolism, TCA-cycle, purine metabolism, taurine and hypotaurine metabolism and pentose phosphate pathway (Table 2). Importantly, disruptions in alanine, aspartate, and glutamate metabolism reflect altered amino acid transamination and nitrogen balance, which are closely linked to hepatic energy production and inflammatory signaling^109–111^. Elevated glutamate levels can enhance oxidative stress and promote the release of pro-inflammatory cytokines, while disruptions in alanine and aspartate flux indicate impaired gluconeogenesis and mitochondrial dysfunction during inflammation. Additionally, alterations in the TCA cycle signify mitochondrial stress and a shift toward incomplete oxidative metabolism. Accumulation of intermediates such as succinate and fumarate can act as metabolic signals that stabilize hypoxia-inducible factor 1α (HIF-1α) and amplify inflammatory cytokine production, thus coupling mitochondrial dysfunction to inflammation^112,113^. Moreover, enhanced purine turnover is often a hallmark of cellular stress and inflammation, leading to increased production of uric acid and reactive oxygen species through xanthine oxidase activity^114–116^. These metabolites contribute to oxidative stress, endothelial injury, and activation of inflammatory cascades within hepatic and vascular tissues. Further associating systemic and liver inflammation, observed dysregulation of taurine and hypotaurine, the sulfur-containing amino acids with potent antioxidants and cytoprotective properties, reduce the liver’s capacity to buffer oxidative stress, leading to lipid peroxidation, mitochondrial injury, and heightened inflammatory signaling^117–119^. In addition, altered PPP activity reflects disrupted cellular redox homeostasis, as this pathway generates NADPH for antioxidant defense and lipid biosynthesis^120,121^. Insufficient NADPH production under oxidative stress limits glutathione regeneration, thereby exacerbating ROS accumulation and perpetuating inflammation in hepatic tissue. Thus, our metabolomic analysis demonstrates that estrogen plays acritical role in regulation of inflammation and its reduction resulting in systemic inflammation.

**Table 2:**
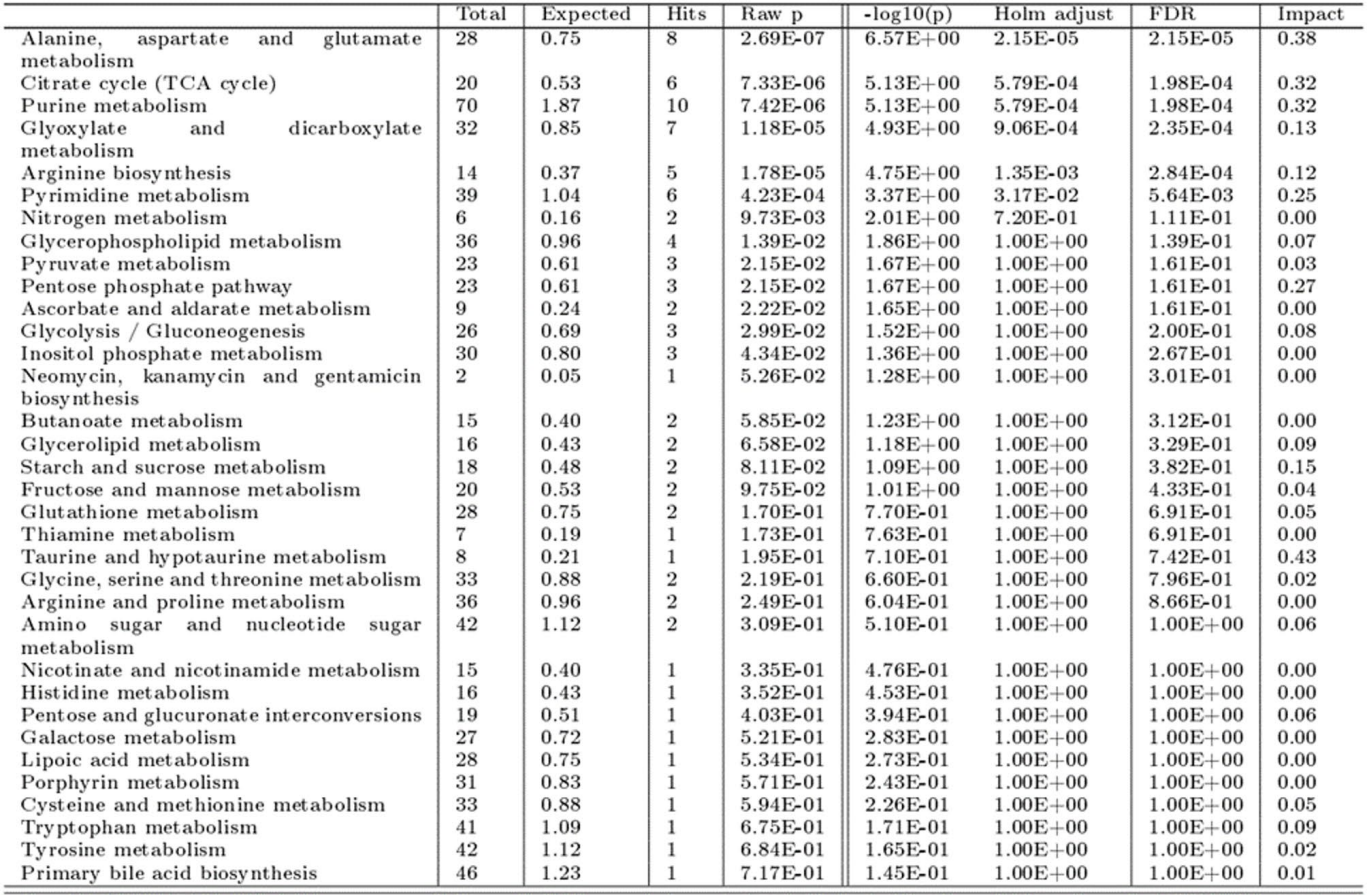
Results from pathway analysis.

## Discussion

Inflammation is central to immune response and unresolved inflammation is the root cause of many chronic and metabolic diseases including cardiovascular disease, obesity, diabetes, and cancer. Liver is an important organ and plays pivotal roles in metabolism, vascular, and immunological functions^9,10,12^. It regulates carbohydrates, protein and fat metabolism. Liver regulates lipid uptake, storage, release and homeostasis. For example, VLDL and HDL are assembled in the endoplasmic reticulum of liver, and their level is tightly regulated by LDLR and SR-BI mediated uptake, along with apolipoproteins such as apoB100 which, together, orchestrate lipid trafficking between peripheral tissues and the liver to control lipid and cholesterol homeostasis^9,122–124^. Hepatic malfunction induces metabolic stress and dyslipidemia by altering lipoprotein secretion, increasing de novo lipogenesis, and impairing β-oxidation contributing to increased risk of cardiovascular disease. Examples of hepatic malfunction associated diseases include NAFLD, steatosis, nonalcoholic steatohepatitis (NASH), and hepatic fibrosis. Studies suggest that there is close correlation between hepatic diseases and cardiovascular risk^10–12^. For example, the symptoms associated with NAFLD are also associated with atherosclerosis.

Liver’s health and functions are closely influenced by sex hormones. Estrogen deficiency increases vulnerability to metabolic dysfunction–associated steatotic liver disease (MASLD) as shown in both clinical and animal studies where loss of estrogen signaling enhances hepatic lipid accumulation, inflammation, and insulin resistance^7,9,12–15,17,19^. Mechanistically, reduced ERα activity in hepatocytes diminishes fatty acid oxidation and antioxidant defense, predisposing the liver to steatosis and inflammatory injury.

Additionally, pre-menopausal women are at lower risk of cardiovascular disease compared to their age matched men and this risk increases^125–130^. Across longitudinal cohorts, the menopause transition is accompanied by rises in total cholesterol, LDL, and by adverse shifts in HDL quantity and function—changes that amplify atherogenic and hepatic inflammatory signaling^125–130^. Estrogen deficiency increases vulnerability to metabolic dysfunction–associated steatotic liver disease (MASLD), with longer durations of postmenopausal hypoestrogenism linked to greater fibrosis risk; mechanistic studies highlight hepatic estrogen signaling as a regulator of lipid handling and inflammatory tone. These hepatic changes intersect with systemic risk: postmenopausal women exhibit higher rates of dyslipidemia, insulin resistance, and subclinical atherosclerosis, contributing to cardiovascular disease burden. The detailed signaling mechanism and molecular fingerprint of the hormonal imbalance, hepatic function and the dyslipidemia and their implications in cardiovascular diseases is poorly understood. In this article, using ovariectomized rats as animal models of estrogen-deprivation, we investigated the significance of ovarian hormones in regulation of hepatic inflammation, tryptophan catabolism, and cholesterol homeostasis.

Our studies demonstrated that OVX animals gained more body weight in comparison to the intact controls and displayed atherogenic lipoprotein profiles, LDL and total cholesterol increased, HDL declined—despite identical diet and housing. Estrogen supplementation attenuated weight gain and improved the lipid profile, indicating hormone sensitivity of hepatic lipid handling, transport, and cholesterol homeostasis. Although circulating estradiol levels were not directly quantified in the present study, it is well established that ovariectomy in rats markedly reduces plasma 17β-estradiol concentrations, typically from approximately 35–40 pg/mL in intact females to low or near-undetectable levels (< 10–20 pg/mL). Estradiol replacement restores circulating levels to physiological or supraphysiological ranges depending on dose and delivery method, as demonstrated in multiple OVX rodent models. Thus, the metabolic and inflammatory alterations observed in our OVX animals are consistent with an estrogen-deficient state that is reversible upon EB supplementation^131–133^.

Converging hepatic readouts revealed that OVX livers upregulated IL-6 and TNF-α at both transcript and protein levels, a signature of inflammatory activation that was rescued by EB. Along with inflammatory cytokines, the reverse cholesterol transport associated with high density lipoprotein receptor SR-BI expression was downregulated in OVX animal liver and this was reversed by EB. Along with SR-BI, EB-treatment also induced the estrogen-receptors overexpression suggesting upregulation of hepatic estrogen-signaling. Notably, previous studies from our laboratory and others demonstrated that estradiol plays key roles in regulation of SR-BI expression in hepatocytes and modulate cholesterol uptakes and homeostasis, in vitro and in vivo^41^. Decline in ovarian hormone (such as in post-menopausal women) has been linked with decreased plasma HDL levels and increased cholesterol and hence increased risk of CVD^68,69^. Thus, our observation with elevated plasma cholesterol and reduced SR-BI expression in OVX animals and their reversal by EB support the crucial roles of ovarian hormones in regulation of hepatic SR-BI and cholesterol homeostasis.

In a recent study, we discovered that SR-BI expression and cholesterol uptakes are reduced in macrophages under inflammation and this is regulated via tryptophan catabolizing enzyme IDO1^52^. Inflammation induces the IDO1 expression and its catabolites Kynurenine level in macrophages. Our previous studies also demonstrated that knockdown or inhibition of IDO1 rescued the inflammation induced downregulation of SR-BI and cholesterol uptakes in macrophages, suggesting crucial roles of IDO1-KYN signaling the SR-BI regulation and cholesterol homeostasis. Based on these foundations, we examined the expression of Trp-catabolizing enzymes IDO1 in the OVX animal liver and also analyzed the level of KYN level in the animal plasma. Interestingly, our studies revealed that both IDO1 is induced in the OVX animal liver and EB reversed it. Further, the plasma Kyn level is elevated in the OVX animals, and this was reversed by E2. Additionally, TDO2, another Trp-catabolizing enzyme which are known to be expressed in liver, is also upregulated in OVX animal liver and reversed by EB. The increase in both IDO1 and TDO2 in liver and KYN in plasma suggest a correlation between hepatic IDO1 expression and Trp-catabolism under hormone deficiency associated in inflammation in OVX animals. The increased hepatic IDO1, plasma KYN level, plasma cholesterol level and reduced SR-BI expression in OVX animals further suggest an inverse correlation between hepatic IDO1 and SR-BI expression and their potential impacts in regulation of plasma cholesterol and lipid levels. Along with Trp-catabolite, we also measured plasma nitric oxide (NO) and lactate levels in the control, OVX and OVX+E2-treated animals. NO is well known biomarker for inflammation. Additionally, increased lactate is another indication of inflammatory stress. Interestingly, our results demonstrated that along elevation of KYN level, the levels of NO and lactate are also elevated in the OVX plasma, and these are reduced by EB-treatment. These observations further demonstrated that ovariectomy not only exerted hepatic inflammation but also contributed to elevation in systemic inflammation further implicating the critical roles of ovarian hormones in management of hepatic as well as systemic inflammatory stress.

Inflammation and immune response majorly contributed via macrophage activation. In general, upon inflammation, macrophages are activated into pro-inflammatory macrophages, which secrete various cytokines and thereby support the immune response, clear infections, and facilitate healing. Macrophages crosstalk with surrounding tissues, resulting in remodeling the tissue microenvironment and immuno-metabolic reprogramming, and ultimately leading to immune response. To further understand the potential role of estrogen in regulating inflammatory stress, we stimulated two different types of macrophages (THP1-derived macrophages and liver resident primary Kupffer cells) with LPS in the presence and absence of estradiol and then analyzed the cytokine expression, IDO1, and Trp-catabolism. Along with systemic inflammation, our results also indicated hepatic inflammation under ovariectomy and its regulation by estradiol. Therefore, in addition to liver tissue analysis and THP-1 macrophages, we examined the inflammatory response in primary Kupffer cells isolated from rat livers. Our results demonstrated that LPS- exposure induced the inflammatory response, including cytokine expression (IL6, IL-1β, TNFα) in macrophages, along with Trp-catabolic enzymes IDO1 and associated catabolites Kyn levels in both THP1 and primary KCs. The expression of SR-BI was downregulated under inflammation. Furthermore, these effects were reversed upon estradiol treatment. These analyses demonstrated a key role of estradiol in regulating inflammatory response and Trp-catabolism in macrophages. These in vitro results are consistent with our in vivo observations. We hypothesize that macrophage activation across different tissue environments (including the liver) is closely associated with the inflammatory response, metabolic reprogramming, and lipid homeostasis, and that these are modulated by ovarian hormones in females. Notably, in a recent study, we demonstrated that IDO1 and trp-catabolism are closely associated with SR-BI expression and cholesterol uptake by the macrophages under inflammation (both using LPS and IFNγ as stimulants)^52^. Thus, observations further support our finding that inflammation reprogrammed Trp-catabolism and potentially other metabolic pathways in vivo.

To further understand the more detailed picture of inflammation stress and functions of ovarian hormones at the systemic level, we performed a targeted metabolomic analysis in OVX and OVX+E2 animal plasma. This analysis resulted in the detection of a wide range of metabolites that are affected under OVX-induced inflammation. The top hits include Trp-catabolism – the OVX animals plasma showed high level of KYN and that was reduced by EB-treatment, the KYN/Trp ratio was significantly high in the OVX animal compared to the OVX+E2 treated group. Kynurenic acid, a downstream metabolite in the kynurenine pathway, is also elevated in the OVX animal compared to OVX+E2 animals. These observations suggested that ovarian hormone derivation induced inflammation is associated with elevated Trp-catabolism and this is potentially mediated by IDO1 and TDO2. Notably, Trp-catabolism is well recognized to be linked to inflammation, immune response and inflammatory diseases^44–46^. For example, IDO1 mediated kynurenine signaling activates NF-κB in endothelial cells, promoting adhesion molecule expression (VCAM-1, ICAM-1) and monocyte recruitment, associating Trp catabolism directly to endothelial dysfunction and atherogenesis as confirmed in both murine and human studies where elevated kynurenine levels correlate with carotid intima–media thickness and endothelial oxidative stress^51,134^. Inhibition of IDO1 has been shown to restore endothelial function and reduce vascular inflammation, underscoring the causal link between Trp catabolism and atherosclerotic progression^51,135^.

Similar to colorimetric measurement data, the metabolomic analysis also resulted in identification of elevated lactate levels in OVX animals’ plasma in comparison to the OVX+E2 group. The elevation in plasma lactate and the increased lactate/pyruvate ratio in OVX mice suggest a metabolic shift toward aerobic glycolysis, commonly observed in activated macrophages and inflamed hepatocytes. This “Warburg-like” metabolic shift prioritizes rapid ATP generation and biosynthetic precursor production over oxidative phosphorylation^96–99,136^. Beyond being a metabolic byproduct, lactate serves as an immunometabolite that shapes inflammatory responses, intracellular lactate accumulation promotes protein lactylation and modulation of downstream gene expression and immune function. Elevated lactate in OVX plasma therefore reflects systemic immune activation and mitochondrial stress, while E2-mediated reductions in lactate indicate restoration of oxidative metabolism and attenuation of inflammatory glycolysis.

Metabolomic profiles showing elevated citrate, isocitrate, and aconitate with relatively lower level of α-ketoglutarate and malate suggest a potential block in the tricarboxylic acid (TCA) cycle distal to aconitate, a metabolic hallmark of inflammatory macrophages. Accumulated citrate can be exported to the cytosol and produce acetyl-CoA for fatty acid synthesis and prostaglandin and nitric oxide production and modulation of inflammatory mediators^103,137^. Concurrently, increased aconitate serves as a substrate for aconitate decarboxylase 1 (ACOD1), producing itaconate—an immunoregulatory metabolite that inhibits succinate dehydrogenase, stabilizes Nrf2 via KEAP1 alkylation, and suppresses excessive IL-1β and type I interferon responses and constrain inflammation^103,104^. Notably, our recent studies demonstrated that ACOD1 expression is also upregulated in macrophages under inflammation^57^. This study shows the estrogen-mediated restoration of the TCA cycle and metabolites reflects the critical roles of ovarian hormones in regulating oxidative stress, thus balancing inflammatory and anti-inflammatory metabolic outputs.

Phosphorylcholine (PC) was elevated in OVX plasma, reflecting enhanced phospholipid turnover and oxidative remodelling under inflammatory stress. Oxidized PC epitopes on damaged membranes act as potent danger-associated molecular patterns (DAMPs), engaging pattern recognition receptors such as Toll-like receptors 2 and 4 (TLR2/4) and scavenger receptors including CD36 and SR-A1^105–108^. These lipid-associated signals also activate hepatic innate immune activation and Kupffer cell infiltration. In estrogen-deficient states, increased oxidative lipid remodelling coincides with impaired antioxidant defenses, driving chronic inflammation and atherogenic lipid profiles. E2 supplementation restores membrane lipid integrity by enhancing expression of antioxidant enzymes and reducing oxidative phospholipid generation, thereby limiting inflammatory phospholipid signalling and complement activation.

Disruptions in alanine, aspartate, and glutamate metabolism highlight altered anaplerotic flux and glutaminolysis during inflammation^109–111^. Glutamine-derived α-ketoglutarate supports mitochondrial respiration. A reduction in α-ketoglutarate, as reflected by lower α-ketoglutarate/aconitate ratios, skews macrophages toward a proinflammatory phenotype. Aspartate and alanine serve as nitrogen donors for nucleotide and amino acid biosynthesis, and their altered ratios signal enhanced cellular proliferation and cytokine synthesis in immune cells. In parallel, perturbations in purine metabolism suggest increased nucleotide turnover and reliance on salvage pathways to sustain transcriptional demand. Estrogen’s antioxidant and metabolic-stabilizing effects likely mitigate these disruptions by preserving mitochondrial integrity and maintaining glutamine flux into the TCA cycle.

Alterations in taurine and hypotaurine metabolism indicate diminished antioxidant buffering and impaired cellular osmoregulation^117,118^. Taurine is a sulfur-containing amino acid that conjugates bile acids and scavenges hypochlorous acid, thereby protecting cells from oxidative injury. Recent studies have shown that taurine depletion enhances NLRP3 inflammasome activation, promoting IL-1β release and hepatic macrophage recruitment^138^. Hypotaurine, a taurine precursor, participates in the detoxification of reactive oxygen species and the regulation of mitochondrial respiration^118^. Reduced taurine/hypotaurine turnover in OVX plasma therefore signifies increased oxidative burden and a heightened proinflammatory state. Estradiol supports taurine biosynthesis and mitochondrial redox homeostasis, so E2 restoration likely reestablishes antioxidant capacity and suppresses inflammasome-driven inflammation.

**Fig. 8:**
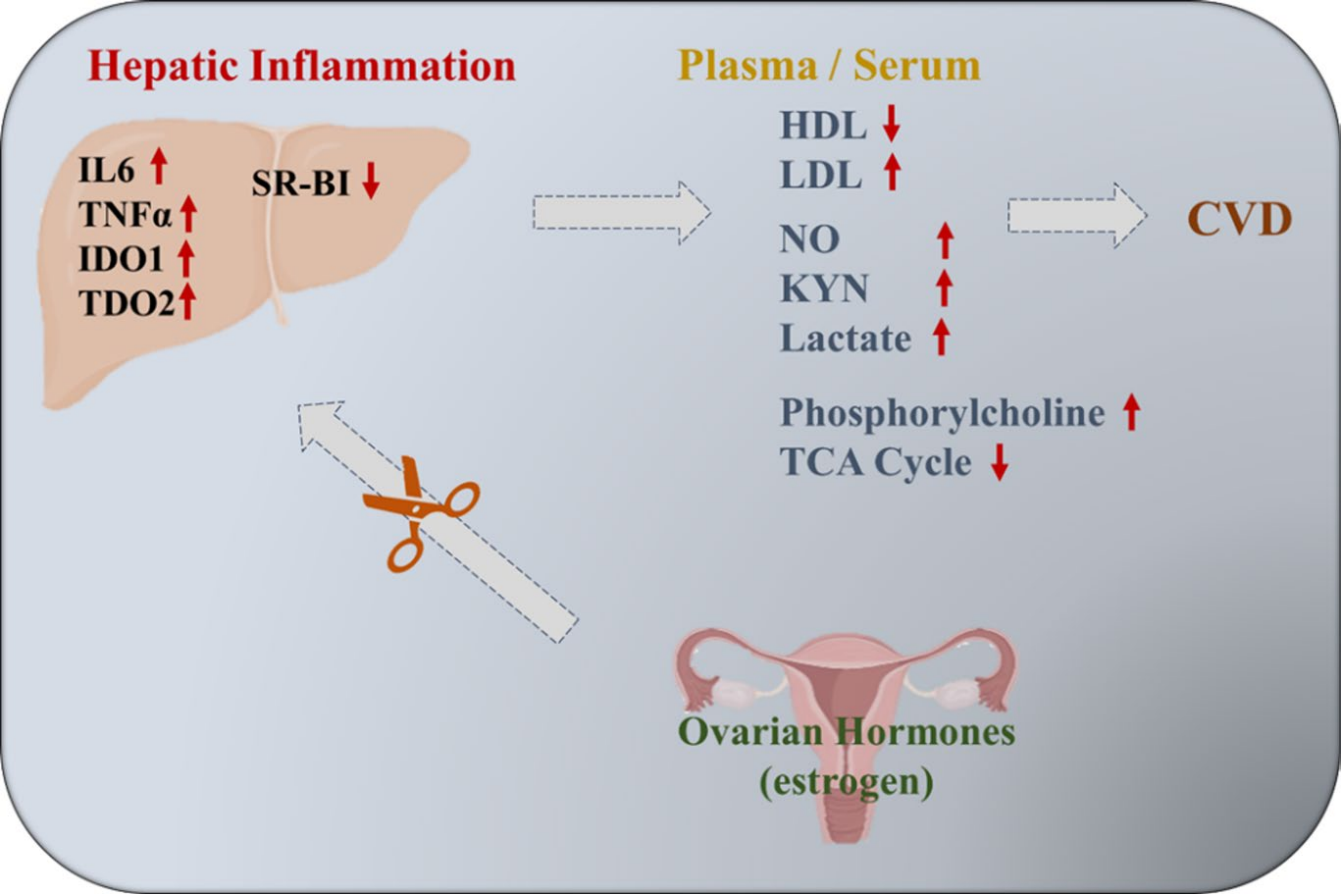
Estradiol deficiency regulates hepatic inflammation and blood metabolite levels. The model shows the molecular and physiological consequences of estrogen loss in ovariectomy (OVX) condition and demonstrates its effect on cholesterol regulation, tryptophan metabolism and hepatic inflammatory markers.

Collectively, these metabolic alterations converge into a unified model of estrogen-deficiency–driven hepatic inflammation characterized by enhanced IDO1/TDO2–KYN signaling, glycolytic reprogramming, phospholipid oxidation, TCA-cycle rewiring with itaconate accumulation, amino acid and purine pathway disruption, and redox imbalance through impaired PPP and taurine metabolism. Each pathway contributes to a self-reinforcing inflammatory loop wherein immune activation, oxidative stress, and metabolic strain perpetuate hepatic injury and systemic inflammation. Estradiol exerts a broad counter-regulatory influence—repressing IDO1 and iNOS induction, restoring mitochondrial oxidative metabolism, enhancing NADPH-dependent antioxidant capacity, and stabilizing membrane lipid homeostasis. A model showing the integrated network of hepatic–systemic inflammatory metabolism under estrogen deficiency, illustrating key nodes (IDO1/TDO2, and SR-BI) is depicted in Fig. 8. Though further research is essential to fully unfold the metabolic reprogramming networks and therapeutic leverage at specific nodes of this network, our studies open up new signaling pathways associated with immuno-metabolism. In conclusion, estrogen deprivation activates a liver-centered immune-metabolic program characterized by cytokine induction, IDO1/TDO2-driven tryptophan catabolism, repression of SR-BI, and broad reorganization of central redox pathways, culminating in dyslipidemia and systemic inflammatory stress. Estradiol restores balance across these nodes. Our studies open up new avenues for the treatment of cardiometabolic diseases and hormone disorders.

## Supplementary Information

Below is the link to the electronic supplementary material.

Supplementary Material 1

## Data Availability

The data including the raw data is available upon a reasonable request to the corresponding author – Subhrangsu S. Mandal (email: smandal@uta.edu).
