# Supplementary material for: Estrogen deprivation induces hepatic inflammation, Indoleamine-2,3-dioxygenase 1, tryptophan catabolism, and plasma cholesterol
Source: Sci Rep. 2026 Apr 24;16:19050. doi: 10.1038/s41598-026-48938-w (PMC13279942; doi:10.1038/s41598-026-48938-w)
Supplement: Supplementary file 1 — Supplementary Material 1 [file 41598_2026_48938_MOESM1_ESM.pdf]

Supplementary figures Guha et al.

Estrogen deprivation induces hepatic inflammation, Indoleamine-2,3-dioxygenase 1, tryptophan catabolism, and plasma cholesterol

Prarthana Guha[1,a], Ashcharya Rishi[1,a], Avisankar Chini[1,a], Nagashree Bhat[1], Pavan K. Gondrala[1], Blake Brady[2], Hamid R Baniasadi[3] Linda I Perrotti[2], and Subhrangsu S. Mandal[1]*

[1]Gene Regulation and Epigenetics Research Laboratory, Department of Chemistry and Biochemistry, The University of Texas at Arlington, Arlington, Texas 76019.; [2]Department of Psychology, The University of Texas at Arlington, Arlington, Texas 76019; [3]Department of Biochemistry, University of Texas Southwestern Medical Center at Dallas, Texas 75390.

Supplementary Figure S1 (Original western blot images used for the figure 2B)

Figure 2B (Top panel)

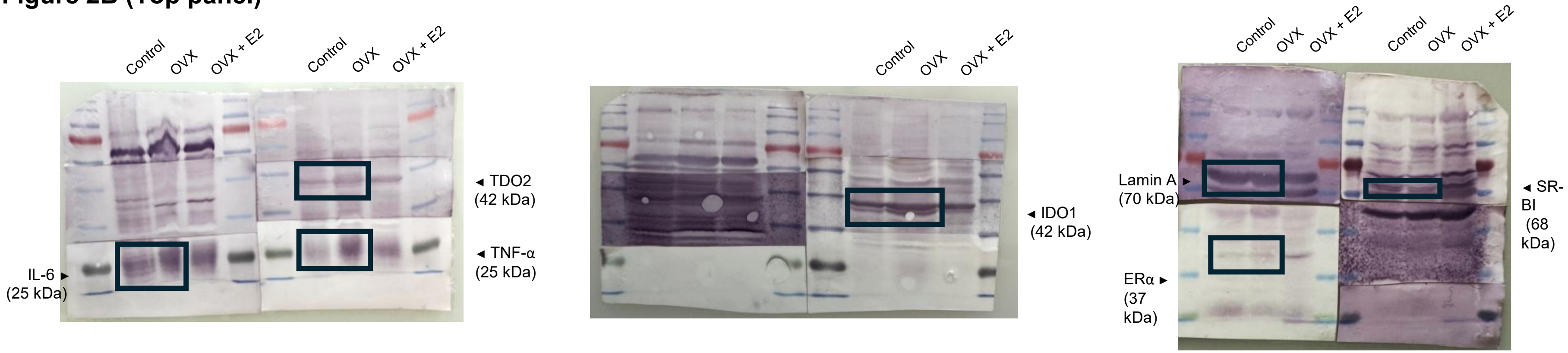

Figure 2B (Bottom panel)

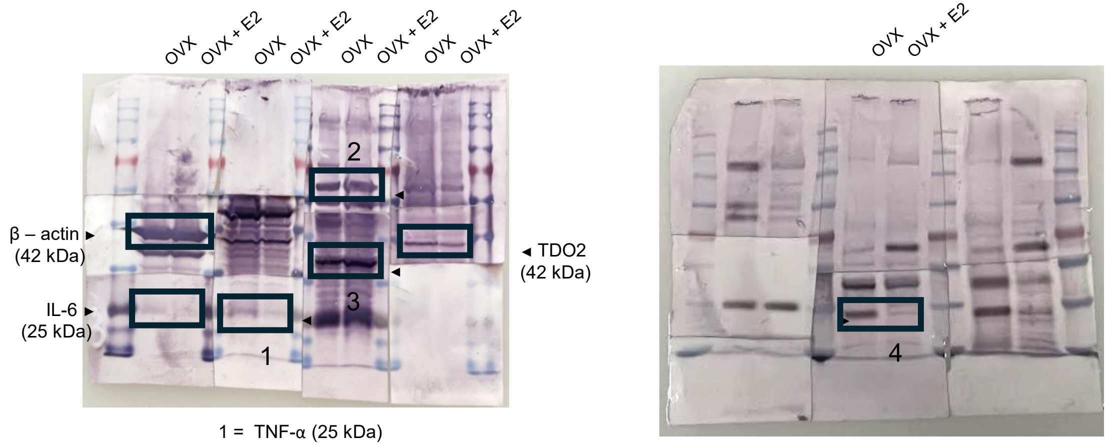

1 = TNF-α (25 kDa)
2 = SR-BI (68 kDa)
3 = ERα (37 kDa)

4 = IDO1 (42 kDa)

Supplemental Figure S2, Guha et al

Figure S2: Metabolomics analysis Raw data

	Sample Name	Rack Code	Rack Position	Plate Code	Plate Position	Vial Positi
1	Blank-1	1.5mL Standa	1	1.5mL Standa	1	1
2	HB-8-0.5uM-start- run-1	1.5mL Standa	1	1.5mL Standa	1	2
3	HB-8-0.5uM-start- run-2	1.5mL Standa	1	1.5mL Standa	1	2
4	Blank-2	1.5mL Standa	1	1.5mL Standa	1	1
5	QC-star-run-1	1.5mL Standa	1	1.5mL Standa	1	4
6	QC-star-run-2	1.5mL Standa	1	1.5mL Standa	1	4
7	Blank-3	1.5mL Standa	1	1.5mL Standa	1	1
8	433-B	1.5mL Standa	1	1.5mL Standa	1	5
9	435-B	1.5mL Standa	1	1.5mL Standa	1	6
10	402-A	1.5mL Standa	1	1.5mL Standa	1	7
11	403-A	1.5mL Standa	1	1.5mL Standa	1	8
12	434-B	1.5mL Standa	1	1.5mL Standa	1	9
13	401-A	1.5mL Standa	1	1.5mL Standa	1	10
14	Blank-4	1.5mL Standa	1	1.5mL Standa	1	1
15	QC-end-run-1	1.5mL Standa	1	1.5mL Standa	1	4
16	QC-end-run-2	1.5mL Standa	1	1.5mL Standa	1	4
17	Blank-5	1.5mL Standa	1	1.5mL Standa	1	1
18	HB-8-0.5uM-end- run-1	1.5mL Standa	1	1.5mL Standa	1	2
19	HB-8-0.5uM-end- run-2	1.5mL Standa	1	1.5mL Standa	1	2
20	Blank-6	1.5mL Standa	1	1.5mL Standa	1	1

on	Data File	Inj.Volume (µl)
	24-M093-pos	5.000
	24-M093-pos	5.000
	24-M093-pos	5.000
	24-M093-pos	5.000
	24-M093-pos	5.000
	24-M093-pos	5.000
	24-M093-pos	5.000
	24-M093-pos	5.000
	24-M093-pos	5.000
	24-M093-pos	5.000
	24-M093-pos	5.000
	24-M093-pos	5.000
	24-M093-pos	5.000
	24-M093-pos	5.000
	24-M093-pos	5.000
	24-M093-pos	5.000
	24-M093-pos	5.000
	24-M093-pos	5.000
	24-M093-pos	5.000
	24-M093-pos	5.000

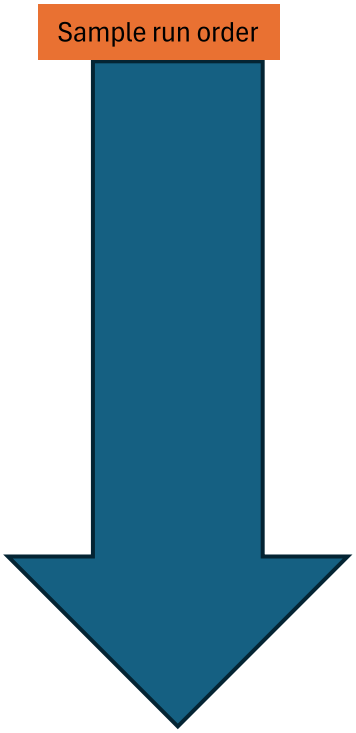

Original Filename	)93\2024_09_03\Data	)93\2024_09_03\Data
Sample Name	401-A	402-A
	RT(min)	RT(min)
Acq. Method Name	C-7-12-24-column-D	C-7-12-24-column-D
Urea(61.1/44.2/RT(min):5.12)	5.1	5.1
ethanolamine(62.1/44.2/RT(min):12.53)	12.7	12.6
Imidazole(69/42.24/RT(min):3.61)	N/A	N/A
glycine(76.1/30.5/RT(min):8.53)	N/A	N/A
alanine(90.1/44.2/RT(min):7.79)	7.6	7.6
choline(104/60/RT(min):12.15	12.0	11.8
4-aminobutyrate(104.01/69/RT(min):8.27)	8.1	8.1
dimethylglycine(104.02/58/RT(min):5.92)	5.8	5.8
serine(106/60/RT(min):8.48)	8.3	8.3
cytosine(112.1/95/RT(min):5.47)	N/A	N/A
creatinine(114/44.2/RT(min):4.53)	4.5	4.5
proline(116.1/70.1/RT(min):6.38)	6.3	6.3
indole(118/91/RT(min):3.58)	N/A	N/A
betaine(118.02/58/RT(min):5.33)	5.3	5.3
valine(118.1/55.2/RT(min):6.08)	6.0	6.0
threonine(120/74/RT(min):7.42)	7.3	7.3
homoserine(120.15/44.2/RT(min):8.02)	N/A	N/A
purine(121/94/RT(min):3.62)	N/A	N/A
cysteine(122.1/59.1/RT(min):8.90)	N/A	N/A
creatine(132.003/90/RT(min):7.78)	7.6	7.6
nicotinamide(123.1/80/RT(min):3.55)	3.8	3.8
Imidazoleacetic acid(127.002/81/RT(min):5.54)	N/A	N/A
thymine(127.1/110/RT(min):3.37)	3.8	3.8
DL-Pipecolic acid(130/84/RT(min):6.05)	6.0	6.0
N-Acetylputrescine(131.001/114/RT(min):11.70)	11.7	11.7
hydroxyproline(132.004/68.2/RT(min):7.66)	7.5	7.5
leucine(132.1/86/RT(min):5.07)	5.0	5.0
ornithine(133/70/RT(min):12.81)	12.9	12.8
asparagine(133.1/74/RT(min):8.18)	8.0	7.9
aspartate(134/74/RT(min):8.58)	8.2	8.2
adenine(136/119/RT(min):4.39)	N/A	N/A
Methylcysteine(136.02/119.02/RT(min):5.49)	N/A	N/A
homocysteine(136.12/90.1/RT(min):8.97)	N/A	N/A
methylnicotinamide(137.001/94/RT(min):14.07)	14.6	14.5
histidinol(142.1/95/RT(min):10.63)	N/A	N/A
lysine(147/67/RT(min):14.08)	14.0	14.0
glutamine(147.1/84.1/RT(min):8.05)	7.9	7.9
O-acetyl-L-serine(148/106/RT(min):4.87)	4.3	4.4
glutamate(148.1/84.1/RT(min):8.21)	7.9	7.8
methionine(150.1/133/RT(min):5.46)	5.4	5.4

guanine(152.2/110/RT(min):6.33)	N/A	N/A
histidine(156.1/110.1/RT(min):8.90)	N/A	N/A
carnitine(162.1/103/RT(min):6.65)	6.5	6.5
Methionine sulfoxide(166/74/RT(min):7.00)	6.7	6.8
phenylalanine(166.1/103/RT(min):4.63)	4.7	4.7
Pyridoxamine(169/134/RT(min):6.93)	N/A	N/A
pyridoxine(170/134/RT(min):3.65)	3.8	3.8
1-Methyl-Histidine(170.1/124/RT(min):7.52)	6.8	6.7
N-acetyl-L-ornithine(175/115.1/RT(min):8.08)	N/A	N/A
arginine(175.02/60/RT(min):14.59)	14.6	14.6
citrulline(176/159/RT(min):8.66)	8.5	8.5
N-carbamoyl-L-aspartate(177.05/74/RT(min):10.28)	9.7	9.8
glucosamine(180/162/RT(min):7.52)	N/A	N/A
tyrosine(182.1/77/RT(min):6.64)	6.6	6.6
Phosphorylcholine(184.001/125/RT(min):8.38)	8.2	8.2
3-phospho-serine(186/88/RT(min):10.14)	N/A	N/A
N6-Acetyl-L-lysine(189.001/84.2/RT(min):7.97)	7.8	7.8
Acetyllysine(189.002/84/RT(min):7.97)	7.8	7.8
N-acetyl-glutamine(189.1/130/RT(min):4.96)	4.4	4.4
N-acetyl-glutamate (190.1/84.1/RT(min):7.89)	8.0	7.9
Ng_Ng-dimethyl-L-arginine(203/70/RT(min):12.15)	12.1	12.1
Acetylcarnitine DL(204/85/RT(min):5.17)	5.1	5.1
tryptophan(205/146/RT(min):5.74)	5.6	5.6
Kynurenine(209/146/RT(min):5.17)	5.1	5.1
N-acetyl-glucosamine(222/138/RT(min):5.74)	5.8	5.8
Flavone(223/121/RT(min):2.42)	N/A	N/A
cystathionine(223/134/RT(min):9.64)	9.4	9.3
5-methoxytryptophan(235/176/RT(min):5.21)	5.3	5.3
Cystine(241.002/74/RT(min):9.08)	8.6	8.6
cytidine(244.1/112/RT(min):5.95)	5.9	5.9
biotin(245.1/227/RT(min):3.79)	N/A	N/A
deoxyadenosine(252/136/RT(min):3.73)	N/A	N/A
D-glucosamine-6-phosphate(260/126/RT(min):*9.99)	N/A	N/A
thiamine(265/122/RT(min):12.39)	12.3	12.2
S-ribosyl-L-homocysteine_pos(268/88/RT(min):4.15)	N/A	N/A
deoxyguanosine(268.1/152/RT(min):5.60)	N/A	N/A
adenosine(268.15/136.1/RT(min):4.20)	4.2	4.2
1-Methyladenosine(281.8/150/RT(min):2.80)	N/A	N/A
guanosine(284.1/135/RT(min):6.55)	N/A	N/A
L-arginino-succinate(291/70/RT(min):9.96)	9.6	9.6
S-methyl-5-thioadenosine(298/136/RT(min):2.55)	N/A	N/A
7-methylguanosine(298.002/166/RT(min):6.95)	N/A	N/A
dCMP(308/112/RT(min):8.67)	N/A	N/A
glutathione (308.1/162/RT(min):7.8)	7.6	7.6

dTMP(323/81/RT(min):6.99)	N/A	N/A
CMP(324/112/RT(min):9.44)	N/A	N/A
UMP(325/97/RT(min):8.91)	N/A	N/A
dAMP(332.1/136/RT(min):6.78)	N/A	N/A
Nicotinamide ribotide(335/123/RT(min):8.71)	N/A	N/A
aminoimidazole carboxamide ribonucleotide(339/110/RT(min):8.74)	N/A	N/A
thiamine-phosphate(345.2/122/RT(min):7.62)	7.4	7.4
dGMP(348.1/135/RT(min):9.31)	N/A	N/A
AMP(348.15/136/RT(min):7.87)	7.4	7.4
IMP(349/137/RT(min):9.31)	N/A	N/A
S-adenosyl-L-methioninamine(355/250/RT(min):9.1)	N/A	N/A
GMP(364/152/RT(min):10.18)	9.7	9.7
xanthosine-5-phosphate(365/97/RT(min):11.18)	N/A	N/A
riboflavin(377/243/RT(min):3.94)	4.0	4.0
S-adenosyl-L-homoCysteine_pos(385.1/136/RT(min):6.97)	6.9	6.9
S-adenosyl-L-methionine(399.1/250/RT(min):9.1)	9.0	9.0
folate(442/295/RT(min):10.47)	9.8	9.9
7_8-dihydrofolate(444.2/178/RT(min):9.86)	9.4	9.3
Diiodothyronine(525.5/352.8/RT(min):2.40)	N/A	N/A
glutathione disulfide_pos(613/231/RT(min):10.30)	10.4	10.4
NAD+_pos(664.1/428/RT(min):7.88)	N/A	N/A
NADH(666.1/514/RT(min):7.21)	N/A	N/A
NADP+_pos(744.2/136/RT(min):10.56)	N/A	N/A
NADPH(746.15/729/RT(min):10.90)	N/A	N/A
coenzyme A_pos(768/261/RT(min):8.00)	N/A	N/A
FAD(786/348/RT(min):6.25)	6.0	6.1
acetyl-CoA_pos(810/303/RT(min):7.39)	N/A	N/A
sarcosine(90.04/44.1/RT(min):*7.21)	N/A	N/A
13C-Proline1(122.00/75.00/RT(min):6.38)	6.3	6.3
13C-Alanine-1(93.94/47.00/RT(min):7.79)	7.6	7.6
13C-Serine-1(110.05/63.00/RT(min):8.48)	8.3	8.3
13C-Valine-1(123.93/77.10/RT(min):6.08)	6.0	6.0
13C-Glycine-1(78.91/32.00/RT(min):8.53)	8.3	8.3
13C-Cystine-1(248.89/155.90/RT(min):9.08)	8.6	8.6
13C-Glutamic acid-1(154.05/89.00/RT(min):8.22)	7.8	7.8
13C-Histidine-1(164.98/117.90/RT(min):8.98)	N/A	N/A
13C-Leucine-1(138.99/92.00/RT(min):5.07)	5.0	5.0
13C-Methionine-1(155.90/138.00/RT(min):5.46)	5.4	5.4
13C-Phenylalanine-1(176.01/128.90/RT(min):4.60)	4.7	4.7
13C-Tyrosine(192.01/174.00/RT(min):6.64)	6.6	6.6
13C valine-1(124.00/59.00/RT(min):6.08)	N/A	N/A
13C tyrosine(192.00/83.00/RT(min):6.64)	6.6	6.6
13 C phenylalanine(176.00/111.00/RT(min):4.60)	4.7	4.7
13C lysine(155.00/90.10/RT(min):14.08)	14.1	14.0

Adenylosuccinate-1(464/252/RT(min): 11.58)	N/A	N/A
Isoleucine(132.1/86/RT(min):5.33)	5.2	5.2
13C-IsoLeucine-1(138.99/92.00/RT(min):5.33)	5.2	5.2
	0.0	0.0
	0.0	0.0
Original Filename	)93\2024_09_03\Data	)93\2024_09_03\Data
Sample Name	401-A	402-A
	RT(min)	RT(min)
Acq. Method Name	C-7-12-24-column-D	C-7-12-24-column-D
glycolate(75/45.2/RT(min):2.90)	3.0	3.0
pyruvate(87/43/RT(min):3.55)	3.8	3.8
lactate(89/43.2/RT(min):4.09)	3.9	3.9
glycerate(105/75/RT(min):5.81)	5.7	5.7
uracil(111.05/42.1/RT(min):4.00)	N/A	N/A
fumarate(115/71/RT(min):9.91)	9.3	9.3
Maleic acid(115.03/71.03/RT(min):4.45)	4.3	4.2
2-keto-isovalerate(115.05/71.05/RT(min):2.6)	N/A	N/A
succinate(117/73/RT(min):9.10)	8.5	8.5
Methylmalonic acid(117.002/73.1/RT(min):8.35)	N/A	N/A
nicotinate(122/78/RT(min):3.60)	3.8	3.8
taurine(124/80/RT(min):7.72)	7.6	7.6
Pyroglutamic acid(128/82.1/RT(min):4.60)	4.1	4.2
Citraconic acid(129.003/85.1/RT(min):4.35)	N/A	N/A
N-Acetyl-L-alanine(130/88/RT(min):3.62)	3.8	3.8
Hydroxyisocaproic acid(131.006/85.1/RT(min):2.30)	2.2	2.3
malate(133/115/RT(min):9.78)	9.3	9.3
hypoxanthine(135/92/RT(min):4.92)	N/A	N/A
anthranilate/p-aminobenzoate(136/92/RT(min):4.8)	N/A	N/A
p-hydroxybenzoate(137/93/RT(min):5.07)	N/A	N/A
Carbamoyl phosphate(140/79/RT(min):9.47)	N/A	N/A
a-ketoglutarate(145/101/RT(min):9.42)	8.8	8.9
Phenylpropiolic acid(145.004/101/RT(min):2.34)	N/A	N/A
2-Hydroxy-2-methylbutanedioic acid(147.001/85.1/RT(min):)	N/A	N/A
3-methylphenylacetic acid(149.002/105/RT(min):2.34*)	2.2	2.2
xanthine (151/108/RT(min):5.73)	N/A	N/A
Hydroxyphenylacetic acid(151.004/107/RT(min):2.60)	N/A	N/A
2_3-dihydroxybenzoic acid(153/109/RT(min):3.62)	N/A	N/A
orotate(155/111/RT(min):4.71)	4.2	4.3
dihydroorotate(157/113/RT(min):5.20)	N/A	N/A
allantoin(157.05/114/RT(min):7.19)	7.1	7.1
Aminoadipic acid(160.001/116/RT(min):8.42)	N/A	N/A
Indole-3-carboxylic acid(160.002/116/RT(min):4.24)	N/A	N/A

phenylpyruvate(163/91/RT(min):2.38)	2.2	2.2
Phenyllactic acid(165.006/103.1/RT(min):2.30)	2.3	2.3
quinolinate(166/122/RT(min):9.13)	8.6	8.6
phosphoenolpyruvate(167/79/RT(min):11.22)	N/A	N/A
Uric acid(167.001/124/RT(min):6.71)	6.8	6.8
dihydroxy-acetone-phosphate(169/79/RT(min):9.03)	N/A	N/A
D-glyceraldehdye-3-phosphate(169.05/97/RT(min):9.03)	N/A	N/A
sn-glycerol-3-phosphate(171/79/RT(min):8.41)	N/A	N/A
shikimate(173/93/RT(min):7.70)	N/A	N/A
aconitate(173.05/85/RT(min):11.22)	10.5	10.6
allantoate(175/132/RT(min):8.35)	8.0	8.0
Ascorbic acid(175.001/87/RT(min):4.30)	N/A	N/A
2-Isopropylmalic acid(175.002/115/RT(min):6.14)	N/A	N/A
N-carbamoyl-L-aspartate_neg(175.03/132/RT(min):10.28)	9.7	9.7
glucono-D-lactone(177/129/RT(min):5.29)	N/A	N/A
myo-inositol(179/161/RT(min):9.34)	9.2	9.2
hydroxyphenylpyruvate(179.05/107/RT(min):3.59)	3.8	3.8
homocysteic acid(182/80/RT(min):9.63)	9.2	9.2
4-Pyridoxic acid(182.003/138/RT(min):2.26)	2.2	2.2
3-phosphoglycerate(185/97/RT(min):10.87)	N/A	N/A
Indoleacrylic acid(186/142.03/RT(min):3.60)	3.8	3.8
Kynurenic acid(188/144/RT(min):2.32)	2.3	2.3
citrate-isocitrate(191/111/RT(min):12)	11.4	11.4
isocitrate (191.02/117/RT(min):12.12)	11.6	11.5
citrate (191.05/87/RT(min):12.12)	11.4	11.4
D-gluconate(195/129/RT(min):7.59)	N/A	N/A
Xanthurenic acid(204.001/160/RT(min):5.65)	5.6	5.6
lipoate(205/171/RT(min):2.22)	N/A	N/A
D-glucarate(209/85/RT(min):10.20)	9.9	9.9
deoxyribose-phosphate(213/79/RT(min):8.45)	N/A	N/A
pantothenate(218/146/RT(min):3.59)	3.8	3.8
deoxyuridine(227/184/RT(min):3.82)	N/A	N/A
ribose-phosphate(229/79/RT(min):9.50)	N/A	N/A
thymidine(241/125/RT(min):3.46)	3.8	3.8
uridine(243/200/RT(min):4.70)	4.7	4.7
deoxyinosine(251/135/RT(min):4.55)	N/A	N/A
hexose-phosphate(259/79/RT(min):9.7*)	N/A	N/A
glucose-1-phosphate(259.01/241/RT(min):9.62)	N/A	N/A
glucose-6-phosphate(259.02/199/RT(min):10.38)	9.9	9.9
fructose-6-phosphate(259.03/169/RT(min):9.72)	N/A	N/A
inosine(267/135/RT(min):5.41)	5.3	5.3
6-phospho-D-gluconate(275/97/RT(min):11.53)	10.6	10.6
xanthosine(283/151/RT(min):6.41)	6.5	6.5
D-sedoheptulose-1-7-phosphate(289/97/RT(min):*9.81)	N/A	N/A

N-acetyl-glucosamine-1-phosphate(300/79/RT(min):8.6)	8.2	8.2
glutathione_neg(306/143/RT(min):7.9)	7.6	7.6
dUMP_neg(307/195/RT(min):7.78)	N/A	N/A
dTMP_neg(321/195/RT(min):6.99)	N/A	N/A
cyclic-AMP(328/134.05/RT(min):4.45)	N/A	N/A
fructose-1_6-bisphosphate(339/97/RT(min):12.10)	N/A	N/A
sucrose(341/179/RT(min):*8.09)	8.0	8.0
trehalose(341/179/RT(min):8.82)	N/A	N/A
S-adenosyl-L-homocysteine_neg(383.1/134/RT(min):6.97)	6.9	6.9
dCDP_neg(386/159/RT(min):10.01)	N/A	N/A
Deoxycholic acid(391.202/345.2/RT(min):2.11)	2.1	2.1
dTDP_neg(401/159/RT(min):8.87)	N/A	N/A
CDP_neg(402/384/RT(min):10.80)	N/A	N/A
UDP_neg(403/159/RT(min):10.38)	N/A	N/A
Thiamine pyrophosphate(423.1/302/RT(min):8.54)	N/A	N/A
ADP_neg(426.1/159/RT(min):9.38)	N/A	N/A
dGDP_neg(426.12/159/RT(min):10.67)	N/A	N/A
IDP_neg(427/159/RT(min):10.65)	N/A	N/A
GDP_neg(442/159/RT(min):11.48)	N/A	N/A
CDP-ethanolamine(445/273/RT(min):9.43)	N/A	N/A
dCTP_neg(466/159/RT(min):11.23)	N/A	N/A
dUTP_neg(467/159/RT(min):10.76)	N/A	N/A
dTTP_neg(481/159/RT(min):10.24)	N/A	N/A
CTP_neg(482/384/RT(min):12.02)	N/A	N/A
UTP_neg(483/159/RT(min):11.60)	N/A	N/A
dATP_neg(490/159/RT(min):9.95)	9.6	N/A
Taurodeoxycholic acid(498.2/124/RT(min):2.0)	2.0	2.0
ATP_neg(506.1/159/RT(min):10.72)	N/A	N/A
dGTP(506.12/159/RT(min):12.41)	N/A	N/A
GTP_neg(522/424/RT(min):12.59)	N/A	N/A
UDP-D-glucose(565/323/RT(min):10.05)	N/A	N/A
UDP-D-glucuronate(579/403/RT(min):12.37)	N/A	N/A
ADP-D-glucose(588/346/RT(min):8.70)	N/A	N/A
guanosine 5-diphosphate_3-diphosphate(602/504/RT(min):)	N/A	N/A
UDP-N-acetyl-glucosamine(606/385/RT(min):9.04)	N/A	N/A
glutathione disulfide_neg(611/306/RT(min):10.3*)	10.3	10.4
cyclic bis(3->5) dimeric GMP(689/344/RT(min):10.78**)	N/A	N/A
coenzyme A_neg(766/408/RT(min):)	N/A	N/A
acetyl-CoA_neg(808/408/RT(min):7.39)	N/A	N/A
2-hydroxygluterate(147.1/128.7/RT(min):9.18)	8.6	8.6
13C-Serine(107.9/77.0/RT(min):8.49)	8.3	8.3
13C-Methionine(153.9/48.1/RT(min):5.46)	5.3	5.3
13C-taurine(126/80/RT(min):7.73)	N/A	N/A
13C-Xanthine(153/43/RT(min):5.73)	N/A	N/A

)93\2024_09_03\Data	093\2024_09_03\Dat	93\2024_09_03\Data	093\2024_09_03\Dat	093\2024_09_03\Dat
403-A	433-B	434-B	435-B	Blank-3
RT(min)	RT(min)	RT(min)	RT(min)	RT(min)
C-7-12-24-column-D	C-7-12-24-column-D	C-7-12-24-column-D	C-7-12-24-column-D	C-7-12-24-column-D
5.1	5.1	5.1	5.1	N/A
12.6	12.6	12.6	12.7	N/A
N/A	N/A	N/A	N/A	N/A
N/A	N/A	N/A	N/A	N/A
7.6	7.6	7.6	7.6	N/A
11.8	12.1	12.1	12.1	N/A
8.1	8.1	8.1	8.1	N/A
5.8	5.8	5.8	5.8	N/A
8.3	8.3	8.3	8.3	N/A
N/A	N/A	N/A	N/A	N/A
4.5	4.5	4.5	4.5	N/A
6.3	6.3	6.3	6.3	N/A
N/A	N/A	N/A	N/A	N/A
5.3	5.3	5.3	5.3	N/A
6.0	6.0	6.0	6.0	N/A
7.3	7.2	7.3	7.3	N/A
N/A	N/A	N/A	N/A	N/A
N/A	N/A	N/A	N/A	N/A
N/A	N/A	N/A	N/A	N/A
7.6	7.6	7.6	7.6	N/A
3.8	3.8	3.8	3.8	N/A
N/A	N/A	N/A	N/A	N/A
3.8	3.8	3.8	3.8	N/A
6.0	6.0	6.0	6.0	N/A
11.7	11.8	11.8	11.7	N/A
7.5	7.5	7.5	7.5	N/A
5.0	5.0	5.0	5.0	N/A
12.9	12.8	12.8	12.9	12.9
7.9	7.9	8.0	8.0	N/A
8.2	8.2	8.2	8.2	N/A
N/A	N/A	N/A	N/A	N/A
N/A	N/A	N/A	N/A	N/A
N/A	N/A	N/A	N/A	N/A
14.6	14.6	14.6	14.6	N/A
N/A	N/A	N/A	N/A	N/A
14.0	14.0	14.0	14.0	N/A
7.9	7.8	7.9	7.9	N/A
4.4	4.5	4.4	4.5	N/A
7.9	7.8	7.8	7.8	N/A
5.4	5.4	5.4	5.4	N/A

N/A	N/A	N/A	N/A	N/A
N/A	N/A	N/A	N/A	N/A
6.5	6.5	6.5	6.5	N/A
6.8	6.7	6.7	6.8	N/A
4.6	4.6	4.7	4.7	N/A
N/A	N/A	N/A	N/A	N/A
3.8	3.8	3.8	3.8	N/A
6.7	6.9	6.9	6.8	N/A
N/A	N/A	N/A	N/A	N/A
14.6	14.6	14.6	14.6	14.6
8.5	8.5	8.5	8.5	N/A
9.8	9.7	9.7	9.8	N/A
N/A	N/A	N/A	N/A	N/A
6.6	6.6	6.6	6.6	N/A
8.2	8.2	8.2	8.2	N/A
N/A	N/A	N/A	N/A	N/A
7.8	7.7	7.7	7.7	N/A
7.8	7.7	7.8	7.7	N/A
4.4	4.4	4.4	4.4	N/A
7.9	8.0	8.0	8.0	N/A
12.1	12.0	12.0	12.1	N/A
5.1	5.1	5.1	5.1	N/A
5.6	5.6	5.6	5.6	N/A
5.1	5.1	5.1	5.1	N/A
5.8	5.8	5.8	5.7	N/A
N/A	N/A	N/A	N/A	N/A
9.3	9.3	9.3	9.4	N/A
5.3	5.2	5.3	5.3	N/A
8.6	8.6	8.6	8.6	N/A
5.9	5.8	5.9	5.9	N/A
N/A	N/A	N/A	N/A	N/A
N/A	N/A	N/A	N/A	N/A
N/A	N/A	N/A	N/A	N/A
12.2	12.3	12.3	12.3	N/A
N/A	N/A	N/A	N/A	N/A
N/A	N/A	N/A	N/A	N/A
4.2	4.2	4.2	4.2	N/A
N/A	N/A	N/A	N/A	N/A
N/A	N/A	N/A	N/A	N/A
9.6	9.6	9.6	9.6	N/A
N/A	N/A	N/A	N/A	N/A
N/A	N/A	N/A	N/A	N/A
N/A	N/A	N/A	N/A	N/A
7.5	7.5	7.5	7.6	N/A

N/A	N/A	N/A	N/A	N/A
N/A	N/A	N/A	N/A	N/A
N/A	8.4	8.4	8.4	N/A
N/A	6.7	6.7	6.7	N/A
N/A	N/A	N/A	N/A	N/A
N/A	N/A	N/A	N/A	N/A
7.4	7.4	7.4	7.4	N/A
N/A	N/A	N/A	N/A	N/A
7.4	7.4	7.4	7.4	N/A
N/A	8.8	8.8	8.8	N/A
N/A	N/A	N/A	N/A	N/A
9.8	9.7	9.7	9.8	N/A
N/A	N/A	N/A	N/A	N/A
4.0	3.9	3.9	3.9	N/A
6.9	6.9	6.9	6.9	N/A
9.0	8.9	8.9	8.9	N/A
9.9	9.8	9.9	9.9	N/A
9.3	9.3	9.3	9.4	N/A
N/A	N/A	N/A	N/A	N/A
10.4	10.3	10.3	10.4	N/A
N/A	N/A	N/A	N/A	N/A
N/A	N/A	N/A	N/A	N/A
N/A	N/A	N/A	N/A	N/A
N/A	N/A	N/A	N/A	N/A
N/A	N/A	N/A	N/A	N/A
6.1	6.0	6.0	6.0	N/A
N/A	N/A	N/A	N/A	N/A
N/A	N/A	N/A	N/A	N/A
6.3	6.3	6.3	6.3	N/A
7.6	7.6	7.6	7.6	N/A
8.3	8.3	8.3	8.3	N/A
6.0	6.0	6.0	6.0	N/A
8.3	8.3	8.4	8.4	N/A
8.6	8.6	8.6	8.6	N/A
7.8	7.8	7.8	7.8	7.9
N/A	N/A	N/A	N/A	N/A
5.0	5.0	5.0	5.0	5.1
5.4	5.4	5.4	5.4	N/A
4.6	4.7	4.7	4.7	N/A
6.6	6.6	6.6	6.6	N/A
N/A	N/A	N/A	N/A	N/A
6.6	6.6	6.6	6.6	N/A
4.6	4.7	4.7	4.7	N/A
14.0	14.0	14.0	14.0	N/A

N/A	11.4	11.4	11.5	N/A
5.2	5.2	5.2	5.2	N/A
5.2	5.2	5.3	5.2	N/A
0.0	0.0	0.0	0.0	0.0
0.0	0.0	0.0	0.0	0.0

)93\2024_09_03\Data	093\2024_09_03\Dat	93\2024_09_03\Data	093\2024_09_03\Dat	093\2024_09_03\Dat
403-A	433-B	434-B	435-B	Blank-3
RT(min)	RT(min)	RT(min)	RT(min)	RT(min)
C-7-12-24-column-D	C-7-12-24-column-D	C-7-12-24-column-D	C-7-12-24-column-D	C-7-12-24-column-D
3.0	3.0	3.0	3.1	N/A
3.8	3.8	3.8	3.8	N/A
3.8	3.9	3.9	3.9	N/A
5.7	5.6	5.6	5.7	N/A
N/A	N/A	N/A	N/A	N/A
9.3	9.3	9.2	9.2	N/A
4.2	4.2	4.3	4.3	N/A
N/A	N/A	N/A	N/A	N/A
8.5	8.5	8.5	8.5	N/A
N/A	N/A	N/A	N/A	N/A
3.8	3.8	3.8	3.8	N/A
7.6	7.6	7.6	7.6	N/A
4.1	4.2	4.2	4.2	N/A
N/A	N/A	N/A	N/A	N/A
3.8	3.8	3.8	3.8	N/A
2.2	2.3	2.3	2.3	N/A
9.3	9.3	9.2	9.2	N/A
N/A	N/A	N/A	N/A	N/A
N/A	N/A	N/A	N/A	N/A
N/A	N/A	N/A	N/A	N/A
N/A	N/A	N/A	N/A	N/A
8.8	8.8	8.8	8.8	N/A
N/A	N/A	N/A	N/A	N/A
N/A	N/A	N/A	N/A	N/A
2.2	2.2	2.2	2.2	N/A
N/A	N/A	N/A	N/A	N/A
N/A	N/A	N/A	N/A	N/A
N/A	N/A	N/A	N/A	N/A
4.2	4.3	4.3	4.3	N/A
N/A	N/A	N/A	N/A	N/A
7.1	7.0	7.0	7.0	N/A
N/A	N/A	N/A	N/A	N/A
N/A	N/A	N/A	N/A	N/A

2.2	2.3	2.3	2.3	N/A
2.3	2.3	2.3	2.3	N/A
8.6	8.5	8.5	8.5	N/A
N/A	N/A	N/A	N/A	N/A
6.8	6.7	6.7	6.7	N/A
N/A	8.6	8.5	8.5	N/A
N/A	8.6	8.5	8.5	N/A
N/A	N/A	N/A	N/A	N/A
N/A	N/A	N/A	N/A	N/A
10.6	10.5	10.5	10.5	N/A
8.0	8.0	8.0	8.0	N/A
N/A	N/A	N/A	N/A	N/A
N/A	N/A	N/A	N/A	N/A
9.7	9.7	9.7	9.7	N/A
N/A	N/A	N/A	N/A	N/A
9.2	9.2	9.2	9.2	N/A
3.8	3.8	3.8	3.8	N/A
9.2	9.2	9.2	9.2	N/A
2.2	2.2	2.2	2.2	N/A
N/A	10.8	10.8	10.7	N/A
3.8	3.8	3.8	3.8	N/A
2.3	2.3	2.3	2.3	N/A
11.4	11.2	11.2	11.1	11.4
11.5	11.5	11.5	11.5	11.6
11.4	11.2	11.2	11.2	11.4
N/A	N/A	N/A	N/A	N/A
5.6	5.6	5.6	5.6	N/A
N/A	N/A	N/A	N/A	N/A
9.9	9.9	9.9	9.9	N/A
N/A	N/A	N/A	N/A	N/A
3.8	3.8	3.8	3.8	N/A
N/A	N/A	N/A	N/A	N/A
N/A	N/A	N/A	N/A	N/A
3.8	3.8	3.8	3.8	N/A
4.7	4.7	4.7	4.7	N/A
N/A	N/A	N/A	N/A	N/A
N/A	N/A	N/A	N/A	N/A
N/A	N/A	N/A	N/A	N/A
9.9	9.9	9.9	9.9	N/A
N/A	N/A	N/A	N/A	N/A
5.2	5.3	5.3	5.3	N/A
10.6	10.7	10.7	10.7	N/A
6.5	6.4	6.5	6.5	N/A
N/A	N/A	N/A	N/A	N/A

8.2	8.2	8.2	8.2	N/A
7.6	7.6	7.6	7.6	N/A
N/A	N/A	N/A	N/A	N/A
N/A	N/A	N/A	N/A	N/A
N/A	4.0	4.0	4.1	N/A
N/A	N/A	N/A	N/A	N/A
8.0	8.0	8.0	8.0	N/A
N/A	N/A	N/A	N/A	N/A
6.9	6.9	6.9	6.9	N/A
N/A	N/A	N/A	N/A	N/A
2.1	2.1	2.1	2.1	N/A
N/A	N/A	N/A	N/A	N/A
N/A	N/A	N/A	N/A	N/A
N/A	9.8	9.8	9.8	N/A
N/A	N/A	N/A	N/A	N/A
N/A	8.8	8.8	8.8	N/A
N/A	N/A	N/A	N/A	N/A
N/A	N/A	N/A	N/A	N/A
N/A	10.9	10.9	10.9	N/A
N/A	N/A	N/A	N/A	N/A
N/A	N/A	N/A	N/A	N/A
N/A	N/A	N/A	N/A	N/A
N/A	N/A	N/A	N/A	N/A
N/A	N/A	N/A	N/A	N/A
N/A	N/A	N/A	N/A	N/A
N/A	N/A	9.5	9.7	N/A
2.0	2.0	2.0	2.0	N/A
N/A	10.0	10.0	10.0	N/A
N/A	N/A	N/A	N/A	11.4
N/A	N/A	N/A	N/A	N/A
N/A	9.6	9.6	9.6	N/A
N/A	N/A	N/A	N/A	N/A
N/A	N/A	N/A	N/A	N/A
N/A	N/A	N/A	N/A	N/A
N/A	8.6	8.6	8.6	N/A
10.4	10.4	10.3	10.3	N/A
N/A	N/A	N/A	N/A	N/A
N/A	N/A	N/A	N/A	N/A
N/A	N/A	N/A	N/A	N/A
8.6	8.6	8.6	8.6	N/A
8.3	8.3	8.3	8.3	N/A
5.3	5.3	5.3	5.3	N/A
N/A	N/A	N/A	N/A	N/A
N/A	N/A	N/A	N/A	N/A

093\2024_09_03\Dat	093\2024_09_03\Dat	93\2024_09_03\Data	93\2024_09_03\Data
QC-star-run-1	QC-star-run-2	QC-end-run-1	QC-end-run-2
RT(min)	RT(min)	RT(min)	RT(min)
C-7-12-24-column-D	C-7-12-24-column-D	C-7-12-24-column-D	C-7-12-24-column-D
5.1	5.1	5.1	5.1
12.6	12.6	12.6	12.7
N/A	N/A	N/A	N/A
N/A	N/A	N/A	N/A
7.6	7.6	7.6	7.6
11.9	11.9	11.9	11.9
8.1	8.1	8.1	8.1
5.8	5.8	5.8	5.8
8.3	8.3	8.3	8.3
N/A	N/A	N/A	N/A
4.5	4.5	4.5	4.5
6.3	6.3	6.3	6.3
N/A	N/A	N/A	N/A
5.3	5.3	5.3	5.3
6.0	6.0	6.0	6.0
7.3	7.3	7.3	7.3
N/A	N/A	N/A	N/A
N/A	N/A	N/A	N/A
N/A	N/A	N/A	N/A
7.6	7.6	7.6	7.6
3.8	3.8	3.8	3.8
N/A	N/A	N/A	N/A
3.8	3.8	3.8	3.8
6.0	6.0	6.0	6.0
11.7	11.7	11.7	11.7
7.5	7.5	7.5	7.5
5.0	5.0	5.0	5.0
12.8	12.8	12.9	12.9
7.9	7.9	7.9	8.0
8.2	8.2	8.2	8.2
N/A	N/A	N/A	N/A
N/A	N/A	N/A	N/A
N/A	N/A	N/A	N/A
14.5	14.5	14.6	14.6
N/A	N/A	N/A	N/A
14.0	14.0	14.0	14.0
7.8	7.8	7.8	7.9
4.4	4.4	4.4	4.4
7.8	7.8	7.8	7.8
5.4	5.4	5.4	5.4

N/A	N/A	N/A	N/A
N/A	N/A	N/A	N/A
6.5	6.5	6.5	6.5
6.8	6.7	6.8	6.8
4.7	4.6	4.7	4.6
N/A	N/A	N/A	N/A
3.8	3.8	3.8	3.8
6.8	6.8	6.8	6.8
N/A	N/A	N/A	N/A
14.6	14.6	14.6	14.6
8.5	8.5	8.5	8.5
9.7	9.7	9.7	9.7
N/A	N/A	N/A	N/A
6.6	6.6	6.6	6.6
8.2	8.2	8.2	8.2
N/A	N/A	N/A	N/A
7.7	7.7	7.7	7.8
7.7	7.7	7.7	7.8
4.4	4.4	4.4	4.4
8.0	8.0	8.0	8.0
12.0	12.0	12.1	12.1
5.1	5.1	5.1	5.1
5.6	5.6	5.6	5.6
5.1	5.1	5.1	5.1
5.8	5.8	5.8	5.8
N/A	N/A	N/A	N/A
9.3	9.3	9.3	9.4
5.3	5.3	5.3	5.3
8.6	8.6	8.6	8.6
5.9	5.9	5.9	5.9
N/A	N/A	N/A	N/A
N/A	N/A	N/A	N/A
N/A	N/A	N/A	N/A
12.3	12.3	12.3	12.3
N/A	N/A	N/A	N/A
N/A	N/A	N/A	N/A
4.2	4.2	4.2	4.2
N/A	N/A	N/A	N/A
N/A	N/A	N/A	N/A
9.6	9.6	9.6	9.6
N/A	N/A	N/A	N/A
N/A	N/A	N/A	N/A
N/A	N/A	N/A	N/A
7.5	7.5	7.5	7.6

N/A	N/A	N/A	N/A
N/A	N/A	N/A	N/A
8.4	8.4	8.4	8.4
6.7	6.7	6.7	6.7
N/A	N/A	N/A	N/A
N/A	N/A	N/A	N/A
7.4	7.4	7.4	7.4
N/A	N/A	N/A	N/A
7.4	7.4	7.4	7.4
8.8	8.8	8.8	8.8
N/A	N/A	N/A	N/A
9.7	9.7	9.8	9.8
N/A	N/A	N/A	N/A
4.0	4.0	4.0	4.0
6.9	6.9	6.9	6.9
8.9	8.9	8.9	8.9
9.9	9.9	9.9	9.9
9.3	9.3	9.4	9.3
N/A	N/A	N/A	N/A
10.3	10.3	10.4	10.4
N/A	N/A	N/A	N/A
N/A	N/A	N/A	N/A
N/A	N/A	N/A	N/A
N/A	N/A	N/A	N/A
N/A	N/A	N/A	N/A
6.0	6.0	6.0	6.0
N/A	N/A	N/A	N/A
N/A	N/A	N/A	N/A
6.3	6.3	6.3	6.3
7.6	7.6	7.6	7.6
8.3	8.3	8.3	8.3
6.0	6.0	6.0	6.0
8.3	8.4	8.4	8.4
8.6	8.6	8.6	8.6
7.8	7.8	7.8	7.8
N/A	N/A	N/A	N/A
5.0	5.0	5.0	5.0
5.4	5.4	5.4	5.4
4.7	4.6	4.7	4.7
6.6	6.6	6.6	6.6
N/A	N/A	N/A	N/A
6.6	6.6	6.6	6.6
4.7	4.7	4.7	4.7
14.0	14.0	14.0	14.0

11.4	11.4	11.5	11.5
5.2	5.2	5.2	5.2
5.2	5.2	5.2	5.2
0.0	0.0	0.0	0.0
0.0	0.0	0.0	0.0
093\2024_09_03\Dat	093\2024_09_03\Dat	93\2024_09_03\Data	93\2024_09_03\Data
QC-star-run-1	QC-star-run-2	QC-end-run-1	QC-end-run-2
RT(min)	RT(min)	RT(min)	RT(min)
C-7-12-24-column-D	C-7-12-24-column-D	C-7-12-24-column-D	C-7-12-24-column-D
3.0	3.1	3.1	3.0
3.8	3.8	3.8	3.8
3.9	3.9	3.9	3.9
5.7	5.7	5.7	5.7
N/A	N/A	N/A	N/A
9.3	9.3	9.3	9.3
4.3	4.3	4.3	4.2
N/A	N/A	N/A	N/A
8.5	8.5	8.5	8.5
N/A	N/A	N/A	N/A
3.8	3.8	3.8	3.8
7.6	7.6	7.6	7.6
4.2	4.2	4.2	4.2
N/A	N/A	N/A	N/A
3.8	3.8	3.8	3.8
2.3	2.3	2.3	2.3
9.3	9.3	9.3	9.3
N/A	N/A	N/A	N/A
N/A	N/A	N/A	N/A
N/A	N/A	N/A	N/A
N/A	N/A	N/A	N/A
8.8	8.8	8.8	8.8
N/A	N/A	N/A	N/A
N/A	N/A	N/A	N/A
2.2	2.2	2.2	2.2
N/A	N/A	N/A	N/A
N/A	N/A	N/A	N/A
N/A	N/A	N/A	N/A
4.3	4.3	4.3	4.3
N/A	N/A	N/A	N/A
7.1	7.1	7.1	7.1
N/A	N/A	N/A	N/A
N/A	N/A	N/A	N/A

2.2	2.2	2.2	2.2
2.3	2.3	2.3	2.3
8.6	8.6	8.5	8.6
N/A	N/A	N/A	N/A
6.8	6.8	6.8	6.8
8.6	8.6	8.6	8.6
8.6	8.6	8.5	8.6
N/A	N/A	N/A	N/A
N/A	N/A	N/A	N/A
10.5	10.5	10.5	10.5
8.0	8.0	8.0	8.0
N/A	N/A	N/A	N/A
N/A	N/A	N/A	N/A
9.7	9.7	9.7	9.8
N/A	N/A	N/A	N/A
9.2	9.2	9.2	9.2
3.8	3.8	3.8	3.8
9.2	9.2	9.2	9.2
2.2	2.2	2.2	2.2
10.7	10.7	10.7	10.7
3.8	3.8	3.8	3.8
2.3	2.3	2.3	2.3
11.3	11.3	11.2	11.3
11.5	11.5	11.5	11.5
11.3	11.3	11.2	11.3
N/A	N/A	N/A	N/A
5.6	5.6	5.6	5.6
N/A	N/A	N/A	N/A
9.9	9.9	9.9	9.9
N/A	N/A	N/A	N/A
3.8	3.8	3.8	3.8
N/A	N/A	N/A	N/A
N/A	N/A	N/A	N/A
3.8	3.8	3.8	3.8
4.7	4.7	4.7	4.7
N/A	N/A	N/A	N/A
N/A	N/A	N/A	N/A
N/A	N/A	N/A	N/A
9.9	9.9	9.9	9.9
N/A	N/A	N/A	N/A
5.3	5.3	5.3	5.3
10.6	10.6	10.6	10.6
6.5	6.5	6.5	6.5
N/A	N/A	N/A	N/A

8.2	8.2	8.2	8.2
7.6	7.6	7.6	7.6
N/A	N/A	N/A	N/A
N/A	N/A	N/A	N/A
4.0	4.0	4.0	4.0
N/A	N/A	N/A	N/A
8.0	8.0	8.0	8.0
N/A	N/A	N/A	N/A
6.9	6.9	6.9	6.9
N/A	N/A	N/A	N/A
2.1	2.1	2.1	2.1
N/A	N/A	N/A	N/A
N/A	N/A	N/A	N/A
9.8	9.8	9.8	9.8
N/A	N/A	N/A	N/A
8.8	8.8	8.8	8.8
N/A	N/A	N/A	N/A
N/A	N/A	N/A	N/A
10.9	10.9	10.9	11.0
N/A	N/A	N/A	N/A
N/A	N/A	N/A	N/A
N/A	N/A	N/A	N/A
N/A	N/A	N/A	N/A
N/A	N/A	N/A	N/A
N/A	N/A	N/A	N/A
N/A	9.4	N/A	N/A
2.0	2.0	2.0	2.0
10.0	10.1	10.0	10.0
11.3	11.2	11.2	11.2
N/A	N/A	N/A	N/A
9.6	9.6	9.5	9.6
N/A	N/A	N/A	N/A
N/A	N/A	N/A	N/A
N/A	N/A	N/A	N/A
8.6	8.6	8.6	8.6
10.4	10.4	10.3	10.3
N/A	N/A	N/A	N/A
N/A	N/A	N/A	N/A
N/A	N/A	N/A	N/A
8.6	8.6	8.6	8.6
8.3	8.3	8.3	8.3
5.3	5.3	5.3	5.3
N/A	N/A	N/A	N/A
N/A	N/A	N/A	N/A

:s\24-M093\2024_09_03\Data\24-M	:s\24-M093\2024_09_03\Data\24-M	s\24-M093\2024_09_03\Data\24-M0
HB-8-0.5uM-start- run-1	HB-8-0.5uM-start- run-2	HB-8-0.5uM-end- run-1
RT(min)	RT(min)	RT(min)
c-pHILIC-7-12-24-column-D-pos.da	c-pHILIC-7-12-24-column-D-pos.da	c-pHILIC-7-12-24-column-D-pos.da
5.1	5.1	5.1
12.7	12.7	12.7
3.8	3.8	3.8
N/A	N/A	N/A
7.6	7.6	7.6
12.2	12.2	12.2
N/A	N/A	N/A
5.8	5.8	5.8
8.3	8.3	8.3
5.4	5.4	5.4
4.5	4.5	4.5
6.3	6.3	6.3
3.8	3.8	3.8
5.3	5.3	5.3
5.9	5.9	5.9
7.2	7.2	7.2
7.8	7.8	7.8
3.8	3.8	3.8
N/A	N/A	N/A
7.6	7.6	7.6
3.8	3.8	3.8
N/A	N/A	N/A
3.8	3.8	3.8
N/A	N/A	N/A
11.8	11.8	11.8
7.5	7.5	7.5
5.0	5.0	5.0
12.9	12.9	12.9
7.9	7.9	7.9
8.1	8.1	8.1
N/A	N/A	N/A
N/A	N/A	N/A
8.7	8.6	8.7
N/A	N/A	N/A
N/A	N/A	N/A
14.1	14.1	14.1
7.8	7.8	7.8
4.3	4.3	4.3
7.7	7.7	7.7
5.5	5.5	5.5

N/A	N/A	N/A
8.5	8.5	8.2
6.5	6.5	6.5
6.4	6.4	6.4
4.6	4.6	4.6
N/A	N/A	N/A
3.8	3.8	3.8
7.1	7.1	6.9
7.9	7.9	7.9
14.7	14.7	14.7
8.5	8.4	8.5
9.7	9.6	9.7
N/A	N/A	N/A
6.5	6.4	6.5
8.2	8.2	8.2
9.8	9.7	9.8
7.7	7.7	7.7
7.7	7.7	7.7
N/A	N/A	N/A
7.7	7.7	7.7
N/A	N/A	N/A
N/A	N/A	N/A
5.6	5.6	5.6
5.1	5.1	N/A
5.7	5.6	5.6
2.3	2.3	2.2
N/A	N/A	N/A
5.1	5.1	5.1
8.6	8.6	8.6
5.9	5.9	5.9
3.8	3.8	3.8
3.8	3.8	3.8
9.5	9.4	9.5
12.4	12.4	12.4
N/A	N/A	N/A
5.5	5.5	5.5
4.2	4.2	4.2
N/A	N/A	N/A
6.4	6.4	6.4
9.6	9.5	9.6
N/A	N/A	N/A
6.9	6.9	6.9
8.1	8.1	8.2
N/A	N/A	N/A

N/A	N/A	N/A
N/A	N/A	N/A
8.3	8.3	8.3
N/A	N/A	N/A
8.3	8.3	8.3
N/A	N/A	N/A
7.4	7.4	7.4
8.8	8.8	8.8
7.3	7.3	7.3
8.7	8.7	8.8
N/A	N/A	N/A
9.7	9.7	9.7
N/A	N/A	N/A
3.9	3.9	3.9
6.8	6.8	6.8
9.0	9.0	9.0
9.8	9.8	9.8
N/A	N/A	N/A
N/A	N/A	N/A
10.3	10.3	10.3
7.5	7.5	7.5
N/A	N/A	N/A
N/A	N/A	N/A
N/A	N/A	N/A
N/A	N/A	N/A
N/A	N/A	N/A
N/A	N/A	N/A
N/A	N/A	N/A
6.2	6.2	6.3
7.6	7.6	7.6
8.3	8.3	8.3
5.9	5.9	5.9
8.3	8.3	8.4
8.6	8.6	8.6
7.7	7.7	7.7
N/A	N/A	N/A
5.0	5.0	5.0
5.3	5.3	5.3
4.6	4.6	4.6
6.5	6.5	6.5
N/A	N/A	N/A
6.5	6.5	6.5
4.6	4.6	4.6
14.1	14.1	14.1

11.4	11.4	11.4
5.0	5.0	5.0
5.2	5.2	5.2
0.0	0.0	0.0
0.0	0.0	0.0
:s\24-M093\2024_09_03\Data\24-M	:s\24-M093\2024_09_03\Data\24-M	s\24-M093\2024_09_03\Data\24-M(
HB-8-0.5uM-start- run-1	HB-8-0.5uM-start- run-2	HB-8-0.5uM-end- run-1
RT(min)	RT(min)	RT(min)
c-pHILIC-7-12-24-column-D-neg.da	c-pHILIC-7-12-24-column-D-neg.da	c-pHILIC-7-12-24-column-D-neg.da
3.0	3.1	3.1
3.7	3.7	3.8
3.8	3.8	3.8
N/A	N/A	N/A
4.1	4.1	4.1
9.2	9.3	9.3
N/A	N/A	N/A
N/A	N/A	N/A
8.4	8.4	8.4
N/A	N/A	N/A
3.8	3.8	3.8
7.5	7.5	7.5
N/A	N/A	N/A
N/A	N/A	N/A
N/A	N/A	N/A
2.3	2.2	2.3
9.3	9.3	9.3
N/A	N/A	N/A
N/A	N/A	N/A
N/A	N/A	N/A
N/A	N/A	N/A
N/A	N/A	N/A
N/A	N/A	N/A
N/A	N/A	N/A
N/A	N/A	N/A
5.1	5.1	5.1
N/A	N/A	N/A
N/A	N/A	N/A
4.1	4.1	4.1
4.8	4.7	4.8
7.1	7.0	7.1
N/A	N/A	N/A
3.8	3.8	3.8

2.2	2.2	2.3
2.3	2.3	2.3
8.5	8.5	8.5
10.6	10.6	10.6
N/A	N/A	N/A
N/A	N/A	N/A
N/A	N/A	N/A
7.9	7.9	7.9
N/A	N/A	N/A
10.5	10.5	10.4
7.9	7.9	7.9
N/A	N/A	N/A
N/A	N/A	N/A
9.7	9.7	9.7
N/A	N/A	N/A
9.2	9.2	9.2
N/A	N/A	N/A
9.1	9.1	9.1
2.2	2.2	2.2
N/A	N/A	N/A
3.7	3.7	3.8
2.3	2.3	2.3
11.4	11.4	11.4
11.5	11.5	11.5
11.4	11.4	11.4
7.1	7.1	7.1
N/A	N/A	N/A
2.2	2.2	2.2
N/A	N/A	N/A
7.9	7.9	7.9
3.7	3.7	3.7
N/A	N/A	N/A
N/A	N/A	N/A
3.8	3.8	3.8
N/A	4.6	4.7
4.5	4.5	4.5
N/A	N/A	N/A
N/A	N/A	N/A
9.9	9.9	9.9
N/A	N/A	N/A
5.3	5.3	5.3
N/A	10.5	N/A
6.4	6.4	6.4
N/A	N/A	N/A

8.2	8.2	8.2
N/A	N/A	N/A
N/A	N/A	N/A
N/A	N/A	N/A
N/A	N/A	N/A
N/A	11.4	11.4
8.0	8.0	7.9
8.6	8.7	8.6
6.8	6.8	6.8
N/A	N/A	N/A
2.1	2.1	2.1
8.4	8.4	8.4
N/A	N/A	N/A
N/A	N/A	N/A
8.2	8.2	8.2
8.9	8.9	8.8
N/A	N/A	N/A
N/A	N/A	N/A
11.0	10.9	10.9
N/A	N/A	N/A
10.8	10.8	10.7
10.8	10.7	10.8
9.7	9.7	9.7
11.4	11.4	11.3
11.0	11.0	11.0
9.5	9.5	9.5
2.0	2.0	2.0
10.2	10.2	10.2
11.3	11.3	11.3
N/A	N/A	N/A
9.5	9.5	9.5
N/A	N/A	N/A
N/A	N/A	N/A
N/A	N/A	N/A
8.5	8.5	8.5
10.3	10.3	10.3
N/A	N/A	N/A
7.8	7.9	7.8
N/A	N/A	N/A
8.5	8.5	8.5
8.3	8.3	8.3
5.3	5.3	5.3
7.5	7.5	7.5
5.1	5.1	5.1

s\24-M093\2024_09_03\Data\24-M0	0	Original Filename
HB-8-0.5uM-end- run-2	0	Sample Name
RT(min)	0	
c-pHILIC-7-12-24-column-D-pos.da	0	Acq. Method Name
5.1	0	Urea(61.1/44.2/RT(min):5.12)
12.7	0	ethanolamine(62.1/44.2/RT(min):12.53)
3.8	0	Imidazole(69/42.24/RT(min):3.61)
N/A	0	glycine(76.1/30.5/RT(min):8.53)
7.6	0	alanine(90.1/44.2/RT(min):7.79)
12.2	0	choline(104/60/RT(min):12.15
N/A	0	4-aminobutyrate(104.01/69/RT(min):8.27)
5.8	0	dimethylglycine(104.02/58/RT(min):5.92)
8.3	0	serine(106/60/RT(min):8.48)
5.4	0	cytosine(112.1/95/RT(min):5.47)
4.5	0	creatinine(114/44.2/RT(min):4.53)
6.3	0	proline(116.1/70.1/RT(min):6.38)
3.8	0	indole(118/91/RT(min):3.58)
5.3	0	betaine(118.02/58/RT(min):5.33)
5.9	0	valine(118.1/55.2/RT(min):6.08)
7.2	0	threonine(120/74/RT(min):7.42)
7.8	0	homoserine(120.15/44.2/RT(min):8.02)
3.8	0	purine(121/94/RT(min):3.62)
N/A	0	cysteine(122.1/59.1/RT(min):8.90)
7.6	0	creatine(132.003/90/RT(min):7.78)
3.8	0	nicotinamide(123.1/80/RT(min):3.55)
N/A	0	Imidazoleacetic acid(127.002/81/RT(min):5.54)
3.7	0	thymine(127.1/110/RT(min):3.37)
N/A	0	DL-Pipecolic acid(130/84/RT(min):6.05)
11.8	0	N-Acetylputrescine(131.001/114/RT(min):11.70)
7.5	0	hydroxyproline(132.004/68.2/RT(min):7.66)
5.0	0	leucine(132.1/86/RT(min):5.07)
12.9	0	ornithine(133/70/RT(min):12.81)
7.9	0	asparagine(133.1/74/RT(min):8.18)
8.1	0	aspartate(134/74/RT(min):8.58)
N/A	0	adenine(136/119/RT(min):4.39)
N/A	0	Methylcysteine(136.02/119.02/RT(min):5.49)
8.7	0	homocysteine(136.12/90.1/RT(min):8.97)
N/A	0	methylnicotinamide(137.001/94/RT(min):14.07)
N/A	0	histidinol(142.1/95/RT(min):10.63)
14.1	0	lysine(147/67/RT(min):14.08)
7.8	0	glutamine(147.1/84.1/RT(min):8.05)
4.3	0	O-acetyl-L-serine(148/106/RT(min):4.87)
7.7	0	glutamate(148.1/84.1/RT(min):8.21)
5.5	0	methionine(150.1/133/RT(min):5.46)

N/A	0	guanine(152.2/110/RT(min):6.33)
8.3	0	histidine(156.1/110.1/RT(min):8.90)
6.5	0	carnitine(162.1/103/RT(min):6.65)
6.4	0	Methionine sulfoxide(166/74/RT(min):7.00)
4.6	0	phenylalanine(166.1/103/RT(min):4.63)
N/A	0	Pyridoxamine(169/134/RT(min):6.93)
3.8	0	pyridoxine(170/134/RT(min):3.65)
6.9	0	1-Methyl-Histidine(170.1/124/RT(min):7.52)
7.9	0	N-acetyl-L-ornithine(175/115.1/RT(min):8.08)
14.7	0	arginine(175.02/60/RT(min):14.59)
8.5	0	citrulline(176/159/RT(min):8.66)
9.7	0	N-carbamoyl-L-aspartate(177.05/74/RT(min):10.28)
N/A	0	glucosamine(180/162/RT(min):7.52)
6.4	0	tyrosine(182.1/77/RT(min):6.64)
8.2	0	Phosphorylcholine(184.001/125/RT(min):8.38)
9.8	0	3-phospho-serine(186/88/RT(min):10.14)
7.7	0	N6-Acetyl-L-lysine(189.001/84.2/RT(min):7.97)
7.7	0	Acetyllysine(189.002/84/RT(min):7.97)
N/A	0	N-acetyl-glutamine(189.1/130/RT(min):4.96)
7.7	0	N-acetyl-glutamate (190.1/84.1/RT(min):7.89)
N/A	0	Ng_Ng-dimethyl-L-arginine(203/70/RT(min):12.15)
N/A	0	Acetylcarnitine DL(204/85/RT(min):5.17)
5.6	0	tryptophan(205/146/RT(min):5.74)
5.1	0	Kynurenine(209/146/RT(min):5.17)
5.6	0	N-acetyl-glucosamine(222/138/RT(min):5.74)
2.2	0	Flavone(223/121/RT(min):2.42)
N/A	0	cystathionine(223/134/RT(min):9.64)
5.1	0	5-methoxytryptophan(235/176/RT(min):5.21)
8.6	0	Cystine(241.002/74/RT(min):9.08)
5.8	0	cytidine(244.1/112/RT(min):5.95)
3.7	0	biotin(245.1/227/RT(min):3.79)
3.8	0	deoxyadenosine(252/136/RT(min):3.73)
9.5	0	D-glucosamine-6-phosphate(260/126/RT(min):*9.99)
12.4	0	thiamine(265/122/RT(min):12.39)
N/A	0	S-ribosyl-L-homocysteine_pos(268/88/RT(min):4.15)
5.4	0	deoxyguanosine(268.1/152/RT(min):5.60)
4.2	0	adenosine(268.15/136.1/RT(min):4.20)
N/A	0	1-Methyladenosine(281.8/150/RT(min):2.80)
6.4	0	guanosine(284.1/135/RT(min):6.55)
9.6	0	L-arginino-succinate(291/70/RT(min):9.96)
N/A	0	S-methyl-5-thioadenosine(298/136/RT(min):2.55)
6.9	0	7-methylguanosine(298.002/166/RT(min):6.95)
8.2	0	dCMP(308/112/RT(min):8.67)
N/A	0	glutathione (308.1/162/RT(min):7.8)

N/A	0	dTMP(323/81/RT(min):6.99)
N/A	0	CMP(324/112/RT(min):9.44)
8.3	0	UMP(325/97/RT(min):8.91)
6.3	0	dAMP(332.1/136/RT(min):6.78)
8.3	0	Nicotinamide ribotide(335/123/RT(min):8.71)
N/A	0	aminoimidazole carboxamide ribonucleotide(339/110/RT(
7.4	0	thiamine-phosphate(345.2/122/RT(min):7.62)
8.8	0	dGMP(348.1/135/RT(min):9.31)
7.3	0	AMP(348.15/136/RT(min):7.87)
8.8	0	IMP(349/137/RT(min):9.31)
N/A	0	S-adenosyl-L-methioninamine(355/250/RT(min):9.1)
9.7	0	GMP(364/152/RT(min):10.18)
N/A	0	xanthosine-5-phosphate(365/97/RT(min):11.18)
3.9	0	riboflavin(377/243/RT(min):3.94)
6.8	0	S-adenosyl-L-homoCysteine_pos(385.1/136/RT(min):6.97
9.0	0	S-adenosyl-L-methionine(399.1/250/RT(min):9.1)
9.8	0	folate(442/295/RT(min):10.47)
N/A	0	7_8-dihydrofolate(444.2/178/RT(min):9.86)
N/A	0	Diiodothyronine(525.5/352.8/RT(min):2.40)
10.3	0	glutathione disulfide_pos(613/231/RT(min):10.30)
7.5	0	NAD+_pos(664.1/428/RT(min):7.88)
N/A	0	NADH(666.1/514/RT(min):7.21)
N/A	0	NADP+_pos(744.2/136/RT(min):10.56)
N/A	0	NADPH(746.15/729/RT(min):10.90)
N/A	0	coenzyme A_pos(768/261/RT(min):8.00)
N/A	0	FAD(786/348/RT(min):6.25)
N/A	0	acetyl-CoA_pos(810/303/RT(min):7.39)
N/A	0	sarcosine(90.04/44.1/RT(min):*7.21)
6.2	0	13C-Proline1(122.00/75.00/RT(min):6.38)
7.6	0	13C-Alanine-1(93.94/47.00/RT(min):7.79)
8.3	0	13C-Serine-1(110.05/63.00/RT(min):8.48)
5.9	0	13C-Valine-1(123.93/77.10/RT(min):6.08)
8.3	0	13C-Glycine-1(78.91/32.00/RT(min):8.53)
8.6	0	13C-Cystine-1(248.89/155.90/RT(min):9.08)
7.7	0	13C-Glutamic acid-1(154.05/89.00/RT(min):8.22)
N/A	0	13C-Histidine-1(164.98/117.90/RT(min):8.98)
5.0	0	13C-Leucine-1(138.99/92.00/RT(min):5.07)
5.3	0	13C-Methionine-1(155.90/138.00/RT(min):5.46)
4.6	0	13C-Phenylalanine-1(176.01/128.90/RT(min):4.60)
6.5	0	13C-Tyrosine(192.01/174.00/RT(min):6.64)
N/A	0	13C valine-1(124.00/59.00/RT(min):6.08)
6.4	0	13C tyrosine(192.00/83.00/RT(min):6.64)
4.6	0	13 C phenylalanine(176.00/111.00/RT(min):4.60)
14.1	0	13C lysine(155.00/90.10/RT(min):14.08)

11.4	0	Adenylosuccinate-1(464/252/RT(min): 11.58)	
5.0	0	Isoleucine(132.1/86/RT(min):5.33)	
5.2	0	13C-IsoLeucine-1(138.99/92.00/RT(min):5.33)	
0.0	#		0.0
0.0	#		0.0
s\24-M093\2024_09_03\Data\24-MC	0	Original Filename	
HB-8-0.5uM-end- run-2	0	Sample Name	
RT(min)	0		
c-pHILIC-7-12-24-column-D-neg.da	0	Acq. Method Name	
3.1	0	glycolate(75/45.2/RT(min):2.90)	
3.7	0	pyruvate(87/43/RT(min):3.55)	
3.8	0	lactate(89/43.2/RT(min):4.09)	
N/A	0	glycerate(105/75/RT(min):5.81)	
4.1	0	uracil(111.05/42.1/RT(min):4.00)	
9.3	0	fumarate(115/71/RT(min):9.91)	
N/A	0	Maleic acid(115.03/71.03/RT(min):4.45)	
N/A	0	2-keto-isovalerate(115.05/71.05/RT(min):2.6)	
8.4	0	succinate(117/73/RT(min):9.10)	
N/A	0	Methylmalonic acid(117.002/73.1/RT(min):8.35)	
3.7	0	nicotinate(122/78/RT(min):3.60)	
7.5	0	taurine(124/80/RT(min):7.72)	
N/A	0	Pyroglutamic acid(128/82.1/RT(min):4.60)	
N/A	0	Citraconic acid(129.003/85.1/RT(min):4.35)	
N/A	0	N-Acetyl-L-alanine(130/88/RT(min):3.62)	
2.3	0	Hydroxyisocaproic acid(131.006/85.1/RT(min):2.30)	
9.3	0	malate(133/115/RT(min):9.78)	
N/A	0	hypoxanthine(135/92/RT(min):4.92)	
N/A	0	anthranilate/p-aminobenzoate(136/92/RT(min):4.8)	
N/A	0	p-hydroxybenzoate(137/93/RT(min):5.07)	
N/A	0	Carbamoyl phosphate(140/79/RT(min):9.47)	
N/A	0	a-ketoglutarate(145/101/RT(min):9.42)	
N/A	0	Phenylpropiolic acid(145.004/101/RT(min):2.34)	
N/A	0	2-Hydroxy-2-methylbutanedioic acid(147.001/85.1/RT(mi	
N/A	0	3-methylphenylacetic acid(149.002/105/RT(min):2.34*)	
5.1	0	xanthine (151/108/RT(min):5.73)	
N/A	0	Hydroxyphenylacetic acid(151.004/107/RT(min):2.60)	
N/A	0	2_3-dihydroxybenzoic acid(153/109/RT(min):3.62)	
4.1	0	orotate(155/111/RT(min):4.71)	
4.8	0	dihydroorotate(157/113/RT(min):5.20)	
7.1	0	allantoin(157.05/114/RT(min):7.19)	
N/A	0	Aminoadipic acid(160.001/116/RT(min):8.42)	
3.8	0	Indole-3-carboxylic acid(160.002/116/RT(min):4.24)	

2.2	0	phenylpyruvate(163/91/RT(min):2.38)
2.3	0	Phenyllactic acid(165.006/103.1/RT(min):2.30)
8.5	0	quinolinate(166/122/RT(min):9.13)
10.6	0	phosphoenolpyruvate(167/79/RT(min):11.22)
N/A	0	Uric acid(167.001/124/RT(min):6.71)
N/A	0	dihydroxy-acetone-phosphate(169/79/RT(min):9.03)
N/A	0	D-glyceraldehdye-3-phosphate(169.05/97/RT(min):9.03)
7.9	0	sn-glycerol-3-phosphate(171/79/RT(min):8.41)
N/A	0	shikimate(173/93/RT(min):7.70)
10.5	0	aconitate(173.05/85/RT(min):11.22)
7.9	0	allantoate(175/132/RT(min):8.35)
N/A	0	Ascorbic acid(175.001/87/RT(min):4.30)
N/A	0	2-Isopropylmalic acid(175.002/115/RT(min):6.14)
9.7	0	N-carbamoyl-L-aspartate_neg(175.03/132/RT(min):10.28
N/A	0	glucono-D-lactone(177/129/RT(min):5.29)
9.2	0	myo-inositol(179/161/RT(min):9.34)
N/A	0	hydroxyphenylpyruvate(179.05/107/RT(min):3.59)
9.1	0	homocysteic acid(182/80/RT(min):9.63)
2.2	0	4-Pyridoxic acid(182.003/138/RT(min):2.26)
N/A	0	3-phosphoglycerate(185/97/RT(min):10.87)
3.7	0	Indoleacrylic acid(186/142.03/RT(min):3.60)
2.3	0	Kynurenic acid(188/144/RT(min):2.32)
11.4	0	citrate-isocitrate(191/111/RT(min):12)
11.5	0	isocitrate (191.02/117/RT(min):12.12)
11.4	0	citrate (191.05/87/RT(min):12.12)
7.2	0	D-gluconate(195/129/RT(min):7.59)
N/A	0	Xanthurenic acid(204.001/160/RT(min):5.65)
2.2	0	lipoate(205/171/RT(min):2.22)
N/A	0	D-glucarate(209/85/RT(min):10.20)
7.9	0	deoxyribose-phosphate(213/79/RT(min):8.45)
3.7	0	pantothenate(218/146/RT(min):3.59)
N/A	0	deoxyuridine(227/184/RT(min):3.82)
N/A	0	ribose-phosphate(229/79/RT(min):9.50)
3.7	0	thymidine(241/125/RT(min):3.46)
4.6	0	uridine(243/200/RT(min):4.70)
4.5	0	deoxyinosine(251/135/RT(min):4.55)
N/A	0	hexose-phosphate(259/79/RT(min):9.7*)
N/A	0	glucose-1-phosphate(259.01/241/RT(min):9.62)
9.9	0	glucose-6-phosphate(259.02/199/RT(min):10.38)
N/A	0	fructose-6-phosphate(259.03/169/RT(min):9.72)
5.3	0	inosine(267/135/RT(min):5.41)
N/A	0	6-phospho-D-gluconate(275/97/RT(min):11.53)
6.5	0	xanthosine(283/151/RT(min):6.41)
N/A	0	D-sedoheptulose-1-7-phosphate(289/97/RT(min):*9.81)

8.2	0	N-acetyl-glucosamine-1-phosphate(300/79/RT(min):8.6)
N/A	0	glutathione_neg(306/143/RT(min):7.9)
N/A	0	dUMP_neg(307/195/RT(min):7.78)
N/A	0	dTMP_neg(321/195/RT(min):6.99)
N/A	0	cyclic-AMP(328/134.05/RT(min):4.45)
11.4	0	fructose-1_6-bisphosphate(339/97/RT(min):12.10)
8.0	0	sucrose(341/179/RT(min):*8.09)
8.6	0	trehalose(341/179/RT(min):8.82)
6.8	0	S-adenosyl-L-homocysteine_neg(383.1/134/RT(min):6.97
N/A	0	dCDP_neg(386/159/RT(min):10.01)
2.1	0	Deoxycholic acid(391.202/345.2/RT(min):2.11)
8.4	0	dTDP_neg(401/159/RT(min):8.87)
N/A	0	CDP_neg(402/384/RT(min):10.80)
N/A	0	UDP_neg(403/159/RT(min):10.38)
8.2	0	Thiamine pyrophosphate(423.1/302/RT(min):8.54)
8.9	0	ADP_neg(426.1/159/RT(min):9.38)
N/A	0	dGDP_neg(426.12/159/RT(min):10.67)
N/A	0	IDP_neg(427/159/RT(min):10.65)
11.0	0	GDP_neg(442/159/RT(min):11.48)
N/A	0	CDP-ethanolamine(445/273/RT(min):9.43)
10.8	0	dCTP_neg(466/159/RT(min):11.23)
10.8	0	dUTP_neg(467/159/RT(min):10.76)
9.7	0	dTTP_neg(481/159/RT(min):10.24)
11.3	0	CTP_neg(482/384/RT(min):12.02)
11.0	0	UTP_neg(483/159/RT(min):11.60)
9.6	0	dATP_neg(490/159/RT(min):9.95)
2.0	0	Taurodeoxycholic acid(498.2/124/RT(min):2.0)
10.2	0	ATP_neg(506.1/159/RT(min):10.72)
11.3	0	dGTP(506.12/159/RT(min):12.41)
N/A	0	GTP_neg(522/424/RT(min):12.59)
9.5	0	UDP-D-glucose(565/323/RT(min):10.05)
N/A	0	UDP-D-glucuronate(579/403/RT(min):12.37)
N/A	0	ADP-D-glucose(588/346/RT(min):8.70)
N/A	0	guanosine 5-diphosphate_3-diphosphate(602/504/RT(mi
8.5	0	UDP-N-acetyl-glucosamine(606/385/RT(min):9.04)
10.3	0	glutathione disulfide_neg(611/306/RT(min):10.3*)
N/A	0	cyclic bis(3->5) dimeric GMP(689/344/RT(min):10.78**)
7.9	0	coenzyme A_neg(766/408/RT(min):)
N/A	0	acetyl-CoA_neg(808/408/RT(min):7.39)
8.5	0	2-hydroxygluterate(147.1/128.7/RT(min):9.18)
8.3	0	13C-Serine(107.9/77.0/RT(min):8.49)
5.3	0	13C-Methionine(153.9/48.1/RT(min):5.46)
7.5	0	13C-taurine(126/80/RT(min):7.73)
5.1	0	13C-Xanthine(153/43/RT(min):5.73)

s\24-M093\2024_09_03\Data\24-M0	s\24-M093\2024_09_03\Data\24-M0	s\24-M093\2024_09_03\Data\24-M0
401-A	402-A	403-A
Peak Height	Peak Height	Peak Height
ic-pHILIC-7-12-24-column-D-pos.da	ric-pHILIC-7-12-24-column-D-pos.da	ric-pHILIC-7-12-24-column-D-pos.dar
1.24E+07	1.37E+07	1.23E+07
4.40E+05	4.47E+05	6.70E+05
N/A	N/A	N/A
N/A	N/A	N/A
6.89E+06	7.19E+06	6.97E+06
1.58E+07	2.05E+07	2.99E+07
3.34E+04	1.86E+04	1.36E+05
9.18E+06	1.03E+07	9.24E+06
4.78E+06	5.79E+06	7.12E+06
N/A	N/A	N/A
1.37E+07	1.45E+07	1.51E+07
5.69E+07	5.83E+07	5.92E+07
N/A	N/A	N/A
6.82E+07	6.88E+07	6.85E+07
1.10E+06	1.26E+06	1.05E+06
1.32E+07	1.57E+07	1.63E+07
N/A	N/A	N/A
N/A	N/A	N/A
N/A	N/A	N/A
6.65E+07	6.88E+07	6.93E+07
7.65E+05	1.14E+06	1.78E+06
N/A	N/A	N/A
7.66E+04	8.88E+04	1.05E+05
6.89E+06	5.86E+06	5.70E+06
1.48E+04	1.68E+04	1.79E+04
1.60E+06	2.39E+06	2.71E+06
4.11E+07	4.03E+07	4.17E+07
1.85E+06	1.57E+06	1.39E+06
8.84E+05	8.31E+05	9.12E+05
3.48E+05	3.16E+05	9.34E+05
N/A	N/A	N/A
N/A	N/A	N/A
N/A	N/A	N/A
3.43E+05	5.84E+05	2.42E+05
N/A	N/A	N/A
1.15E+06	1.09E+06	1.25E+06
4.18E+07	4.27E+07	4.76E+07
3.90E+04	4.81E+04	4.67E+04
4.51E+06	4.77E+06	7.24E+06
8.10E+06	8.83E+06	8.47E+06

N/A	N/A	N/A
N/A	N/A	N/A
4.52E+07	5.29E+07	4.94E+07
3.17E+06	2.48E+06	3.36E+06
1.51E+07	1.51E+07	1.65E+07
N/A	N/A	N/A
8.73E+04	1.50E+05	4.42E+04
1.94E+06	2.61E+06	2.91E+06
N/A	N/A	N/A
9.57E+06	1.06E+07	1.17E+07
4.36E+07	4.42E+07	4.58E+07
3.45E+03	5.71E+03	4.56E+03
N/A	N/A	N/A
1.98E+06	2.29E+06	2.27E+06
3.92E+05	4.96E+05	5.19E+05
N/A	N/A	N/A
2.37E+05	3.01E+05	2.57E+05
2.05E+05	2.48E+05	2.18E+05
3.52E+05	4.50E+05	3.72E+05
3.32E+05	3.67E+05	4.81E+05
1.37E+06	1.72E+06	1.85E+06
2.48E+07	3.16E+07	2.60E+07
2.98E+07	2.86E+07	3.14E+07
5.38E+05	5.73E+05	5.86E+05
5.24E+04	5.08E+04	7.63E+04
N/A	N/A	N/A
1.00E+05	1.61E+05	2.56E+05
1.86E+04	2.59E+04	2.95E+04
4.86E+05	5.28E+05	6.06E+05
5.54E+06	7.56E+06	9.57E+06
N/A	N/A	N/A
N/A	N/A	N/A
N/A	N/A	N/A
6.70E+05	6.54E+05	8.09E+05
N/A	N/A	N/A
N/A	N/A	N/A
1.29E+05	2.59E+05	5.99E+04
N/A	N/A	N/A
N/A	N/A	N/A
2.88E+05	4.07E+05	6.89E+05
N/A	N/A	N/A
N/A	N/A	N/A
N/A	N/A	N/A
2.59E+05	3.23E+05	3.76E+05

N/A	N/A	N/A
N/A	N/A	N/A
N/A	N/A	N/A
N/A	N/A	N/A
N/A	N/A	N/A
N/A	N/A	N/A
4.12E+05	7.51E+05	5.13E+05
N/A	N/A	N/A
2.95E+04	2.26E+04	4.22E+04
N/A	N/A	N/A
N/A	N/A	N/A
1.43E+03	2.41E+03	9.70E+03
N/A	N/A	N/A
8.69E+04	1.07E+05	2.10E+05
3.28E+05	4.22E+05	3.01E+05
6.98E+05	9.25E+05	1.36E+06
6.39E+03	2.34E+04	9.42E+03
1.44E+04	1.75E+04	2.24E+04
N/A	N/A	N/A
2.02E+04	3.73E+04	2.07E+05
N/A	N/A	N/A
N/A	N/A	N/A
N/A	N/A	N/A
N/A	N/A	N/A
N/A	N/A	N/A
1.52E+04	8.57E+03	2.67E+04
N/A	N/A	N/A
N/A	N/A	N/A
6.50E+05	6.40E+05	5.91E+05
1.76E+04	1.35E+04	1.46E+04
3.74E+04	3.15E+04	2.52E+04
2.77E+05	2.76E+05	2.37E+05
1.82E+03	1.51E+03	1.98E+03
6.82E+04	7.33E+04	6.37E+04
6.88E+04	5.91E+04	5.55E+04
N/A	N/A	N/A
4.15E+05	3.87E+05	3.88E+05
2.36E+05	1.92E+05	1.77E+05
1.42E+06	1.35E+06	1.19E+06
7.66E+05	6.32E+05	6.08E+05
N/A	N/A	N/A
5.39E+04	4.19E+04	4.72E+04
2.16E+05	1.94E+05	1.57E+05
4.22E+04	4.05E+04	3.97E+04

N/A	N/A	N/A
1.67E+07	1.56E+07	1.31E+07
3.31E+05	2.42E+05	2.15E+05
0.0	0.0	0.0
0.0	0.0	0.0
s\24-M093\2024_09_03\Data\24-M0	s\24-M093\2024_09_03\Data\24-M0	s\24-M093\2024_09_03\Data\24-M0
401-A	402-A	403-A
Peak Height	Peak Height	Peak Height
ic-pHILIC-7-12-24-column-D-neg.dar	ic-pHILIC-7-12-24-column-D-neg.dar	ic-pHILIC-7-12-24-column-D-neg.dar
1.46E+05	1.28E+05	1.40E+05
1.13E+06	1.40E+06	7.91E+05
4.65E+07	4.80E+07	4.41E+07
5.20E+04	7.30E+04	8.20E+04
N/A	N/A	N/A
3.44E+05	2.85E+05	2.68E+05
4.13E+04	3.62E+04	2.83E+04
N/A	N/A	N/A
2.56E+05	2.94E+05	3.05E+05
N/A	N/A	N/A
1.13E+05	6.97E+05	2.77E+05
2.55E+07	2.62E+07	3.66E+07
1.29E+04	1.87E+04	2.01E+04
N/A	N/A	N/A
1.19E+05	1.28E+05	1.61E+05
8.30E+04	1.15E+05	1.03E+05
2.98E+06	2.60E+06	2.53E+06
N/A	N/A	N/A
N/A	N/A	N/A
N/A	N/A	N/A
N/A	N/A	N/A
1.81E+06	1.59E+06	1.61E+06
N/A	N/A	N/A
N/A	N/A	N/A
8.92E+04	7.84E+04	1.01E+05
N/A	N/A	N/A
N/A	N/A	N/A
N/A	N/A	N/A
4.05E+05	4.69E+05	4.83E+05
N/A	N/A	N/A
2.06E+06	3.21E+06	3.26E+06
N/A	N/A	N/A
N/A	N/A	N/A

2.97E+04	2.87E+04	1.77E+04
1.72E+04	3.34E+04	3.25E+04
3.67E+05	3.63E+05	5.20E+05
N/A	N/A	N/A
3.17E+07	3.58E+07	3.77E+07
N/A	N/A	N/A
N/A	N/A	N/A
N/A	N/A	N/A
N/A	N/A	N/A
3.16E+05	4.04E+05	3.23E+05
3.14E+04	4.16E+04	2.87E+04
N/A	N/A	N/A
N/A	N/A	N/A
1.19E+05	1.65E+05	1.44E+05
N/A	N/A	N/A
7.99E+05	6.15E+05	1.39E+06
1.72E+05	2.60E+05	1.17E+05
4.42E+03	1.01E+04	1.55E+04
3.41E+05	5.95E+05	5.82E+05
N/A	N/A	N/A
1.52E+06	1.44E+06	1.60E+06
5.73E+04	6.84E+04	6.44E+04
1.08E+07	1.32E+07	1.21E+07
5.03E+04	9.42E+04	6.78E+04
3.48E+06	4.24E+06	4.00E+06
N/A	N/A	N/A
3.48E+05	3.83E+05	3.78E+05
N/A	N/A	N/A
1.89E+04	5.20E+04	3.77E+04
N/A	N/A	N/A
7.24E+05	9.90E+05	1.00E+06
N/A	N/A	N/A
N/A	N/A	N/A
1.99E+04	9.91E+03	3.41E+04
1.07E+04	1.34E+04	1.78E+04
N/A	N/A	N/A
N/A	N/A	N/A
N/A	N/A	N/A
1.11E+04	2.63E+04	2.94E+04
N/A	N/A	N/A
8.99E+03	1.75E+04	2.31E+04
1.42E+04	1.21E+04	1.34E+04
1.58E+06	1.81E+06	1.86E+06
N/A	N/A	N/A

6.68E+04	1.04E+05	1.96E+05
5.04E+04	6.79E+04	9.08E+04
N/A	N/A	N/A
N/A	N/A	N/A
N/A	N/A	N/A
N/A	N/A	N/A
5.19E+04	2.20E+05	8.54E+05
N/A	N/A	N/A
2.11E+05	2.65E+05	2.02E+05
N/A	N/A	N/A
2.11E+04	2.78E+04	2.15E+04
N/A	N/A	N/A
N/A	N/A	N/A
N/A	N/A	N/A
N/A	N/A	N/A
N/A	N/A	N/A
N/A	N/A	N/A
N/A	N/A	N/A
N/A	N/A	N/A
N/A	N/A	N/A
N/A	N/A	N/A
N/A	N/A	N/A
N/A	N/A	N/A
N/A	N/A	N/A
N/A	N/A	N/A
3.33E+02	N/A	N/A
5.61E+04	5.93E+04	1.14E+05
N/A	N/A	N/A
N/A	N/A	N/A
N/A	N/A	N/A
N/A	N/A	N/A
N/A	N/A	N/A
N/A	N/A	N/A
N/A	N/A	N/A
N/A	N/A	N/A
2.38E+04	4.54E+04	2.37E+05
N/A	N/A	N/A
N/A	N/A	N/A
N/A	N/A	N/A
2.60E+05	2.27E+05	3.09E+05
1.45E+04	1.09E+04	9.79E+03
2.16E+04	1.84E+04	1.73E+04
N/A	N/A	N/A
N/A	N/A	N/A

ts\24-M093\2024_09_03\Data\24-M(	s\24-M093\2024_09_03\Data\24-M0	ts\24-M093\2024_09_03\Data\24-M(
433-B	434-B	435-B
Peak Height	Peak Height	Peak Height
ic-pHILIC-7-12-24-column-D-pos.da	ric-pHILIC-7-12-24-column-D-pos.da	ric-pHILIC-7-12-24-column-D-pos.dar
7.55E+06	7.95E+06	8.33E+06
1.85E+05	1.41E+05	1.49E+05
N/A	N/A	N/A
N/A	N/A	N/A
5.68E+06	5.56E+06	5.92E+06
1.25E+07	9.63E+06	1.07E+07
1.49E+04	1.55E+04	3.35E+04
7.71E+06	7.85E+06	6.18E+06
4.38E+06	4.39E+06	5.01E+06
N/A	N/A	N/A
1.27E+07	1.20E+07	1.23E+07
5.32E+07	5.33E+07	5.61E+07
N/A	N/A	N/A
6.79E+07	6.90E+07	6.77E+07
8.97E+05	8.91E+05	9.04E+05
1.77E+07	1.52E+07	1.64E+07
N/A	N/A	N/A
N/A	N/A	N/A
N/A	N/A	N/A
6.82E+07	6.84E+07	6.55E+07
3.98E+05	7.11E+05	8.55E+05
N/A	N/A	N/A
5.88E+04	6.21E+04	7.53E+04
3.84E+06	4.93E+06	4.65E+06
1.26E+04	1.65E+04	1.71E+04
3.14E+06	2.14E+06	2.68E+06
2.43E+07	2.63E+07	2.94E+07
1.29E+06	1.19E+06	1.32E+06
4.26E+05	3.36E+05	4.37E+05
2.07E+05	2.16E+05	2.59E+05
N/A	N/A	N/A
N/A	N/A	N/A
N/A	N/A	N/A
3.62E+05	4.99E+05	7.88E+05
N/A	N/A	N/A
8.45E+05	8.00E+05	1.03E+06
1.89E+07	2.02E+07	2.19E+07
2.95E+04	3.92E+04	3.53E+04
1.63E+06	1.60E+06	1.89E+06
6.43E+06	5.77E+06	6.85E+06

N/A	N/A	N/A
N/A	N/A	N/A
3.89E+07	4.14E+07	4.13E+07
2.43E+06	3.11E+06	3.46E+06
1.15E+07	1.13E+07	1.14E+07
N/A	N/A	N/A
8.75E+04	4.76E+04	1.01E+05
2.63E+06	2.09E+06	2.26E+06
N/A	N/A	N/A
9.82E+06	9.63E+06	1.11E+07
4.60E+07	5.06E+07	4.86E+07
7.10E+03	7.36E+03	7.93E+03
N/A	N/A	N/A
1.81E+06	1.58E+06	1.80E+06
2.05E+06	2.03E+06	2.29E+06
N/A	N/A	N/A
9.64E+04	1.17E+05	1.43E+05
7.74E+04	1.11E+05	1.22E+05
2.20E+05	3.33E+05	3.60E+05
2.06E+05	2.32E+05	1.83E+05
1.10E+06	1.25E+06	1.16E+06
1.81E+07	2.96E+07	2.33E+07
2.16E+07	2.46E+07	2.58E+07
5.79E+07	5.10E+07	5.19E+07
2.93E+04	2.19E+04	2.67E+04
N/A	N/A	N/A
1.30E+05	1.13E+05	1.25E+05
1.61E+04	2.59E+04	3.63E+04
2.83E+05	4.96E+05	4.63E+05
6.22E+06	4.81E+06	3.64E+06
N/A	N/A	N/A
N/A	N/A	N/A
N/A	N/A	N/A
3.22E+05	3.61E+05	4.57E+05
N/A	N/A	N/A
N/A	N/A	N/A
5.80E+04	5.34E+05	9.15E+05
N/A	N/A	N/A
N/A	N/A	N/A
5.31E+05	5.41E+05	6.42E+05
N/A	N/A	N/A
N/A	N/A	N/A
N/A	N/A	N/A
3.95E+05	8.86E+05	1.83E+06

N/A	N/A	N/A
N/A	N/A	N/A
6.17E+04	1.06E+05	7.72E+04
5.21E+04	7.01E+04	1.09E+05
N/A	N/A	N/A
N/A	N/A	N/A
4.60E+05	5.49E+05	6.02E+05
N/A	N/A	N/A
2.15E+07	4.19E+07	3.95E+07
5.09E+05	1.19E+06	3.41E+05
N/A	N/A	N/A
1.61E+06	2.09E+06	2.35E+06
N/A	N/A	N/A
1.11E+05	1.28E+05	1.03E+05
3.26E+05	5.28E+05	6.43E+05
1.98E+05	2.79E+05	2.80E+05
2.06E+04	1.03E+04	1.27E+04
1.96E+04	8.58E+03	7.47E+03
N/A	N/A	N/A
2.90E+05	2.67E+05	2.32E+05
N/A	N/A	N/A
N/A	N/A	N/A
N/A	N/A	N/A
N/A	N/A	N/A
N/A	N/A	N/A
1.74E+04	1.67E+04	1.44E+04
N/A	N/A	N/A
N/A	N/A	N/A
6.69E+05	7.52E+05	5.64E+05
1.18E+04	1.13E+04	1.47E+04
2.85E+04	2.94E+04	3.01E+04
2.54E+05	2.52E+05	1.89E+05
1.45E+03	1.40E+03	9.91E+02
6.90E+04	6.37E+04	5.91E+04
3.00E+04	2.88E+04	2.92E+04
N/A	N/A	N/A
2.26E+05	2.51E+05	2.45E+05
1.77E+05	1.58E+05	1.58E+05
1.02E+06	1.02E+06	9.24E+05
3.86E+05	4.24E+05	5.05E+05
N/A	N/A	N/A
2.52E+04	3.00E+04	2.86E+04
1.39E+05	1.55E+05	1.45E+05
4.10E+04	3.63E+04	4.27E+04

1.99E+04	2.60E+04	6.69E+04
1.15E+07	1.01E+07	9.84E+06
2.22E+05	1.95E+05	1.86E+05
0.0	0.0	0.0
0.0	0.0	0.0
ts\24-M093\2024_09_03\Data\24-M(	s\24-M093\2024_09_03\Data\24-M0	ts\24-M093\2024_09_03\Data\24-M(
433-B	434-B	435-B
Peak Height	Peak Height	Peak Height
ic-pHILIC-7-12-24-column-D-neg.da	ric-pHILIC-7-12-24-column-D-neg.da	ric-pHILIC-7-12-24-column-D-neg.dar
1.37E+05	1.37E+05	1.28E+05
8.85E+05	9.74E+05	1.02E+06
4.82E+07	4.80E+07	4.85E+07
1.88E+04	2.23E+04	2.87E+04
N/A	N/A	N/A
1.02E+05	1.12E+05	2.31E+05
2.91E+04	2.64E+04	3.11E+04
N/A	N/A	N/A
2.82E+05	2.52E+05	3.19E+05
N/A	N/A	N/A
1.49E+05	1.44E+05	2.31E+05
1.50E+07	1.76E+07	1.51E+07
1.81E+04	2.02E+04	1.59E+04
N/A	N/A	N/A
9.35E+04	9.22E+04	1.00E+05
7.24E+04	7.29E+04	9.09E+04
9.86E+05	9.74E+05	2.03E+06
N/A	N/A	N/A
N/A	N/A	N/A
N/A	N/A	N/A
N/A	N/A	N/A
1.29E+06	1.05E+06	1.12E+06
N/A	N/A	N/A
N/A	N/A	N/A
5.25E+04	6.76E+04	5.79E+04
N/A	N/A	N/A
N/A	N/A	N/A
N/A	N/A	N/A
2.20E+05	3.25E+05	2.20E+05
N/A	N/A	N/A
9.98E+05	1.54E+06	2.23E+06
N/A	N/A	N/A
N/A	N/A	N/A

2.77E+04	2.04E+04	1.98E+04
9.95E+03	1.18E+04	2.07E+04
4.17E+05	3.90E+05	5.02E+05
N/A	N/A	N/A
2.47E+07	2.71E+07	2.34E+07
2.77E+05	4.15E+05	3.66E+05
6.14E+05	9.40E+05	6.68E+05
N/A	N/A	N/A
N/A	N/A	N/A
1.65E+06	1.82E+06	1.45E+06
1.54E+04	1.91E+04	2.58E+04
N/A	N/A	N/A
N/A	N/A	N/A
1.08E+05	1.18E+05	1.35E+05
N/A	N/A	N/A
2.88E+05	2.77E+05	3.07E+05
1.98E+05	8.96E+04	1.25E+05
5.38E+03	4.73E+03	5.56E+03
3.54E+05	2.84E+05	3.07E+05
2.29E+05	3.31E+05	1.69E+05
9.03E+05	2.56E+06	1.15E+06
7.43E+04	2.04E+05	9.65E+04
1.62E+07	1.58E+07	1.64E+07
4.15E+05	5.19E+05	4.20E+05
4.98E+06	5.44E+06	5.39E+06
N/A	N/A	N/A
2.89E+05	3.16E+05	3.20E+05
N/A	N/A	N/A
1.79E+05	1.91E+05	2.55E+05
N/A	N/A	N/A
6.11E+05	5.62E+05	6.39E+05
N/A	N/A	N/A
N/A	N/A	N/A
4.30E+04	2.61E+04	2.24E+04
2.29E+03	2.43E+03	2.56E+03
N/A	N/A	N/A
N/A	N/A	N/A
N/A	N/A	N/A
6.43E+04	8.78E+04	9.51E+04
N/A	N/A	N/A
6.19E+03	1.73E+04	1.64E+04
1.23E+04	1.89E+04	1.33E+04
8.88E+04	1.20E+05	1.12E+05
N/A	N/A	N/A

6.28E+04	7.33E+04	7.69E+04
1.56E+05	2.54E+05	5.33E+05
N/A	N/A	N/A
N/A	N/A	N/A
3.17E+04	1.77E+04	1.70E+04
N/A	N/A	N/A
8.19E+04	5.23E+04	5.73E+05
N/A	N/A	N/A
2.26E+05	3.58E+05	3.41E+05
N/A	N/A	N/A
1.60E+04	1.01E+04	1.37E+04
N/A	N/A	N/A
N/A	N/A	N/A
9.45E+03	1.64E+04	1.62E+04
N/A	N/A	N/A
1.48E+05	2.01E+05	2.40E+05
N/A	N/A	N/A
N/A	N/A	N/A
1.83E+03	1.94E+03	4.16E+03
N/A	N/A	N/A
N/A	N/A	N/A
N/A	N/A	N/A
N/A	N/A	N/A
N/A	N/A	N/A
N/A	N/A	N/A
N/A	6.67E+02	1.00E+03
9.56E+04	2.69E+04	2.41E+04
1.19E+04	9.11E+03	1.05E+04
N/A	N/A	N/A
N/A	N/A	N/A
5.60E+04	1.95E+04	6.14E+04
N/A	N/A	N/A
N/A	N/A	N/A
N/A	N/A	N/A
4.06E+04	7.57E+04	4.24E+04
2.66E+05	1.55E+05	2.03E+05
N/A	N/A	N/A
N/A	N/A	N/A
N/A	N/A	N/A
1.19E+05	1.41E+05	1.83E+05
7.26E+03	9.85E+03	9.56E+03
1.38E+04	1.68E+04	1.49E+04
N/A	N/A	N/A
N/A	N/A	N/A

ts\24-M093\2024_09_03\Data\24-M(	ts\24-M093\2024_09_03\Data\24-M(	ts\24-M093\2024_09_03\Data\24-M(
Blank-3	QC-star-run-1	QC-star-run-2
Peak Height	Peak Height	Peak Height
ic-pHILIC-7-12-24-column-D-pos.dar	ic-pHILIC-7-12-24-column-D-pos.dar	ic-pHILIC-7-12-24-column-D-pos.dar
N/A	1.35E+07	1.36E+07
N/A	4.10E+05	3.70E+05
N/A	N/A	N/A
N/A	N/A	N/A
N/A	7.25E+06	7.16E+06
N/A	1.78E+07	1.62E+07
N/A	4.37E+04	4.72E+04
N/A	1.01E+07	9.11E+06
N/A	6.25E+06	5.78E+06
N/A	N/A	N/A
N/A	1.59E+07	1.43E+07
N/A	5.89E+07	5.93E+07
N/A	N/A	N/A
N/A	6.83E+07	6.75E+07
N/A	1.27E+06	1.13E+06
N/A	1.73E+07	1.78E+07
N/A	N/A	N/A
N/A	N/A	N/A
N/A	N/A	N/A
N/A	6.95E+07	6.92E+07
N/A	1.24E+06	1.14E+06
N/A	N/A	N/A
N/A	9.99E+04	1.09E+05
N/A	6.39E+06	5.84E+06
N/A	1.96E+04	2.06E+04
N/A	3.03E+06	2.80E+06
N/A	3.95E+07	3.88E+07
6.46E+03	1.66E+06	1.66E+06
N/A	6.28E+05	6.39E+05
N/A	4.65E+05	4.34E+05
N/A	N/A	N/A
N/A	N/A	N/A
N/A	N/A	N/A
N/A	6.10E+05	5.36E+05
N/A	N/A	N/A
N/A	1.16E+06	1.13E+06
N/A	2.84E+07	2.92E+07
N/A	4.88E+04	4.20E+04
N/A	3.11E+06	3.01E+06
N/A	8.62E+06	8.09E+06

N/A	N/A	N/A
N/A	N/A	N/A
N/A	4.75E+07	4.94E+07
N/A	3.11E+06	3.37E+06
N/A	1.53E+07	1.43E+07
N/A	N/A	N/A
N/A	9.32E+04	9.34E+04
N/A	2.56E+06	2.87E+06
N/A	N/A	N/A
2.35E+04	1.21E+07	1.19E+07
N/A	5.04E+07	4.97E+07
N/A	5.91E+03	8.74E+03
N/A	N/A	N/A
N/A	2.30E+06	2.07E+06
N/A	1.65E+06	1.59E+06
N/A	N/A	N/A
N/A	1.72E+05	1.82E+05
N/A	1.40E+05	1.50E+05
N/A	4.54E+05	4.27E+05
N/A	2.75E+05	2.87E+05
N/A	1.81E+06	1.44E+06
N/A	2.67E+07	2.87E+07
N/A	3.05E+07	2.81E+07
N/A	8.95E+06	8.43E+06
N/A	4.22E+04	5.37E+04
N/A	N/A	N/A
N/A	1.59E+05	1.62E+05
N/A	2.73E+04	2.67E+04
N/A	5.53E+05	4.98E+05
N/A	7.44E+06	6.79E+06
N/A	N/A	N/A
N/A	N/A	N/A
N/A	N/A	N/A
N/A	6.41E+05	5.35E+05
N/A	N/A	N/A
N/A	N/A	N/A
N/A	5.08E+05	4.66E+05
N/A	N/A	N/A
N/A	N/A	N/A
N/A	7.67E+05	7.81E+05
N/A	N/A	N/A
N/A	N/A	N/A
N/A	N/A	N/A
N/A	1.04E+06	1.00E+06

N/A	N/A	N/A
N/A	N/A	N/A
N/A	5.28E+04	6.21E+04
N/A	5.25E+04	5.68E+04
N/A	N/A	N/A
N/A	N/A	N/A
N/A	7.27E+05	7.16E+05
N/A	N/A	N/A
N/A	3.23E+07	2.93E+07
N/A	4.18E+05	4.51E+05
N/A	N/A	N/A
N/A	1.34E+06	1.29E+06
N/A	N/A	N/A
N/A	1.63E+05	1.47E+05
N/A	4.09E+05	4.54E+05
N/A	7.65E+05	7.15E+05
N/A	2.49E+04	1.62E+04
N/A	1.89E+04	1.99E+04
N/A	N/A	N/A
N/A	2.12E+05	2.10E+05
N/A	N/A	N/A
N/A	N/A	N/A
N/A	N/A	N/A
N/A	N/A	N/A
N/A	N/A	N/A
N/A	1.85E+04	1.53E+04
N/A	N/A	N/A
N/A	N/A	N/A
N/A	7.49E+05	7.07E+05
N/A	1.55E+04	1.42E+04
N/A	3.63E+04	2.99E+04
N/A	2.92E+05	2.82E+05
N/A	1.55E+03	1.67E+03
N/A	7.30E+04	7.56E+04
1.33E+02	4.66E+04	4.39E+04
N/A	N/A	N/A
1.33E+02	3.65E+05	4.14E+05
N/A	2.17E+05	1.95E+05
N/A	1.41E+06	1.31E+06
N/A	6.11E+05	4.97E+05
N/A	N/A	N/A
N/A	4.51E+04	3.63E+04
N/A	2.06E+05	2.07E+05
N/A	4.60E+04	4.32E+04

N/A	2.32E+04	2.78E+04
N/A	1.40E+07	1.44E+07
N/A	2.65E+05	2.59E+05
0.0	0.0	0.0
0.0	0.0	0.0
ts\24-M093\2024_09_03\Data\24-M(	ts\24-M093\2024_09_03\Data\24-M(	ts\24-M093\2024_09_03\Data\24-M(
Blank-3	QC-star-run-1	QC-star-run-2
Peak Height	Peak Height	Peak Height
ic-pHILIC-7-12-24-column-D-neg.dar	ic-pHILIC-7-12-24-column-D-neg.dar	ic-pHILIC-7-12-24-column-D-neg.dar
N/A	1.23E+05	1.31E+05
N/A	9.16E+05	9.90E+05
N/A	4.80E+07	4.74E+07
N/A	4.53E+04	4.34E+04
N/A	N/A	N/A
N/A	1.83E+05	2.02E+05
N/A	6.14E+04	5.98E+04
N/A	N/A	N/A
N/A	4.59E+05	4.65E+05
N/A	N/A	N/A
N/A	3.04E+05	2.70E+05
N/A	2.29E+07	2.25E+07
N/A	1.90E+04	1.82E+04
N/A	N/A	N/A
N/A	1.11E+05	1.10E+05
N/A	9.39E+04	8.21E+04
N/A	1.72E+06	1.91E+06
N/A	N/A	N/A
N/A	N/A	N/A
N/A	N/A	N/A
N/A	N/A	N/A
N/A	1.54E+06	1.61E+06
N/A	N/A	N/A
N/A	N/A	N/A
N/A	6.72E+04	7.62E+04
N/A	N/A	N/A
N/A	N/A	N/A
N/A	N/A	N/A
N/A	3.75E+05	3.65E+05
N/A	N/A	N/A
N/A	2.34E+06	2.51E+06
N/A	N/A	N/A
N/A	N/A	N/A

N/A	2.22E+04	1.99E+04
N/A	1.61E+04	1.94E+04
N/A	4.37E+05	4.14E+05
N/A	N/A	N/A
N/A	3.00E+07	2.95E+07
N/A	2.21E+05	1.79E+05
N/A	4.80E+05	4.09E+05
N/A	N/A	N/A
N/A	N/A	N/A
N/A	9.22E+05	1.01E+06
N/A	1.72E+04	2.11E+04
N/A	N/A	N/A
N/A	N/A	N/A
N/A	1.39E+05	1.63E+05
N/A	N/A	N/A
N/A	4.50E+05	4.52E+05
N/A	1.68E+05	1.67E+05
N/A	4.80E+03	6.57E+03
N/A	4.13E+05	4.41E+05
N/A	1.62E+05	1.70E+05
N/A	1.69E+06	1.69E+06
N/A	7.54E+04	7.64E+04
1.31E+06	1.20E+07	1.38E+07
3.36E+03	2.49E+05	2.60E+05
4.30E+05	4.04E+06	4.37E+06
N/A	N/A	N/A
N/A	3.73E+05	3.53E+05
N/A	N/A	N/A
N/A	1.34E+05	1.32E+05
N/A	N/A	N/A
N/A	6.99E+05	7.11E+05
N/A	N/A	N/A
N/A	N/A	N/A
N/A	1.16E+04	7.54E+03
N/A	7.77E+03	7.79E+03
N/A	N/A	N/A
N/A	N/A	N/A
N/A	N/A	N/A
N/A	6.49E+04	5.71E+04
N/A	N/A	N/A
N/A	1.71E+04	1.56E+04
N/A	1.62E+04	1.14E+04
N/A	8.84E+05	8.74E+05
N/A	N/A	N/A

N/A	1.23E+05	9.70E+04
N/A	2.12E+05	2.04E+05
N/A	N/A	N/A
N/A	N/A	N/A
N/A	1.35E+04	1.22E+04
N/A	N/A	N/A
N/A	2.50E+05	2.59E+05
N/A	N/A	N/A
N/A	2.71E+05	2.54E+05
N/A	N/A	N/A
N/A	1.95E+04	1.84E+04
N/A	N/A	N/A
N/A	N/A	N/A
N/A	7.12E+03	7.50E+03
N/A	N/A	N/A
N/A	1.10E+05	9.92E+04
N/A	N/A	N/A
N/A	N/A	N/A
N/A	1.03E+03	1.32E+03
N/A	N/A	N/A
N/A	N/A	N/A
N/A	N/A	N/A
N/A	N/A	N/A
N/A	N/A	N/A
N/A	N/A	N/A
N/A	N/A	3.33E+02
N/A	7.33E+04	6.44E+04
N/A	5.83E+03	4.92E+03
2.94E+02	2.20E+02	2.95E+02
N/A	N/A	N/A
N/A	2.20E+04	2.18E+04
N/A	N/A	N/A
N/A	N/A	N/A
N/A	N/A	N/A
N/A	1.96E+04	2.08E+04
N/A	1.76E+05	1.53E+05
N/A	N/A	N/A
N/A	N/A	N/A
N/A	N/A	N/A
N/A	2.55E+05	2.64E+05
N/A	9.26E+03	9.16E+03
N/A	1.67E+04	1.69E+04
N/A	N/A	N/A
N/A	N/A	N/A

s\24-M093\2024_09_03\Data\24-M0	s\24-M093\2024_09_03\Data\24-M0	ts\24-M093\2024_09_03\Data\24-M(
QC-end-run-1	QC-end-run-2	HB-8-0.5uM-start- run-1
Peak Height	Peak Height	Peak Height
ic-pHILIC-7-12-24-column-D-pos.da	ric-pHILIC-7-12-24-column-D-pos.da	ric-pHILIC-7-12-24-column-D-pos.dar
1.18E+07	1.13E+07	2.74E+05
3.30E+05	3.52E+05	6.92E+04
N/A	N/A	1.26E+06
N/A	N/A	N/A
6.18E+06	6.15E+06	2.58E+05
1.57E+07	1.58E+07	5.75E+06
4.26E+04	4.57E+04	N/A
8.85E+06	9.23E+06	3.52E+06
5.40E+06	5.34E+06	1.16E+05
N/A	N/A	3.61E+06
1.32E+07	1.46E+07	2.96E+06
5.67E+07	5.70E+07	2.18E+06
N/A	N/A	9.40E+04
6.71E+07	6.80E+07	1.53E+07
1.12E+06	1.17E+06	3.76E+04
1.74E+07	1.64E+07	2.25E+05
N/A	N/A	9.88E+04
N/A	N/A	1.09E+07
N/A	N/A	N/A
6.77E+07	6.77E+07	8.09E+06
1.14E+06	1.14E+06	2.19E+06
N/A	N/A	N/A
8.42E+04	9.80E+04	9.39E+05
5.94E+06	6.03E+06	N/A
1.83E+04	1.99E+04	1.93E+06
2.55E+06	2.58E+06	8.04E+05
3.97E+07	3.86E+07	1.53E+06
1.44E+06	1.46E+06	1.69E+05
5.04E+05	5.55E+05	6.54E+04
4.05E+05	3.94E+05	2.78E+04
N/A	N/A	N/A
N/A	N/A	N/A
N/A	N/A	1.79E+04
5.26E+05	5.09E+05	N/A
N/A	N/A	N/A
1.03E+06	1.12E+06	6.98E+03
2.70E+07	2.78E+07	9.04E+05
4.33E+04	4.46E+04	2.11E+05
2.92E+06	2.97E+06	2.54E+05
7.75E+06	7.83E+06	2.04E+05

N/A	N/A	N/A
N/A	N/A	2.63E+05
4.99E+07	4.95E+07	7.87E+06
2.93E+06	3.14E+06	2.17E+06
1.39E+07	1.37E+07	6.00E+05
N/A	N/A	N/A
8.47E+04	8.58E+04	1.28E+07
2.65E+06	2.34E+06	8.97E+05
N/A	N/A	4.11E+06
1.14E+07	1.06E+07	4.22E+05
4.85E+07	4.93E+07	4.66E+06
6.32E+03	7.41E+03	1.67E+05
N/A	N/A	N/A
2.13E+06	2.18E+06	1.15E+05
1.36E+06	1.44E+06	1.03E+06
N/A	N/A	2.16E+04
1.76E+05	1.74E+05	6.65E+06
1.41E+05	1.53E+05	6.18E+06
3.75E+05	4.18E+05	N/A
2.40E+05	2.52E+05	5.43E+05
1.47E+06	1.47E+06	N/A
2.87E+07	2.91E+07	N/A
2.91E+07	2.87E+07	9.57E+05
9.09E+06	8.44E+06	1.14E+04
3.51E+04	4.20E+04	3.64E+05
N/A	N/A	4.96E+04
1.61E+05	1.50E+05	N/A
2.33E+04	3.30E+04	2.48E+06
5.38E+05	5.13E+05	3.20E+03
6.77E+06	6.96E+06	7.55E+06
N/A	N/A	5.40E+06
N/A	N/A	5.46E+07
N/A	N/A	3.67E+04
6.03E+05	5.85E+05	2.22E+06
N/A	N/A	N/A
N/A	N/A	2.11E+04
4.76E+05	4.65E+05	3.89E+07
N/A	N/A	N/A
N/A	N/A	3.87E+05
6.09E+05	6.93E+05	1.42E+06
N/A	N/A	N/A
N/A	N/A	2.57E+06
N/A	N/A	1.62E+05
8.70E+05	8.16E+05	N/A

N/A	N/A	N/A
N/A	N/A	N/A
5.87E+04	5.30E+04	6.78E+04
5.59E+04	5.43E+04	N/A
N/A	N/A	1.95E+06
N/A	N/A	N/A
6.42E+05	5.90E+05	3.00E+04
N/A	N/A	2.78E+04
2.88E+07	2.85E+07	7.59E+06
4.43E+05	3.96E+05	1.85E+05
N/A	N/A	N/A
1.34E+06	1.26E+06	5.76E+05
N/A	N/A	N/A
1.54E+05	1.46E+05	3.35E+05
4.32E+05	3.97E+05	8.17E+06
6.74E+05	6.90E+05	1.58E+06
2.35E+04	1.64E+04	1.54E+06
1.76E+04	1.58E+04	N/A
N/A	N/A	N/A
2.06E+05	1.78E+05	1.38E+05
N/A	N/A	1.08E+06
N/A	N/A	N/A
N/A	N/A	N/A
N/A	N/A	N/A
N/A	N/A	N/A
2.78E+04	2.02E+04	N/A
N/A	N/A	N/A
N/A	N/A	N/A
6.41E+05	6.94E+05	2.80E+06
1.23E+04	1.43E+04	8.63E+04
3.00E+04	3.30E+04	8.23E+04
2.60E+05	2.65E+05	8.55E+05
1.59E+03	9.23E+02	3.97E+03
7.17E+04	6.27E+04	3.61E+05
3.89E+04	4.26E+04	1.63E+05
N/A	N/A	N/A
3.71E+05	3.54E+05	1.45E+06
1.93E+05	1.99E+05	7.44E+05
1.31E+06	1.37E+06	2.12E+06
5.09E+05	5.30E+05	8.06E+05
N/A	N/A	N/A
3.92E+04	3.84E+04	5.28E+04
1.93E+05	1.93E+05	2.90E+05
4.91E+04	4.19E+04	1.10E+05

2.32E+04	2.52E+04	3.65E+06
1.29E+07	1.41E+07	1.24E+06
2.37E+05	2.45E+05	1.24E+06
0.0	0.0	0.0
0.0	0.0	0.0
s\24-M093\2024_09_03\Data\24-M0	s\24-M093\2024_09_03\Data\24-M0	ts\24-M093\2024_09_03\Data\24-M(
QC-end-run-1	QC-end-run-2	HB-8-0.5uM-start- run-1
Peak Height	Peak Height	Peak Height
ic-pHILIC-7-12-24-column-D-neg.da	ric-pHILIC-7-12-24-column-D-neg.da	ric-pHILIC-7-12-24-column-D-neg.dar
1.39E+05	1.37E+05	1.04E+05
9.62E+05	9.70E+05	6.22E+03
4.94E+07	4.91E+07	3.11E+05
4.71E+04	4.09E+04	N/A
N/A	N/A	6.78E+05
2.08E+05	2.01E+05	3.59E+04
6.08E+04	5.70E+04	N/A
N/A	N/A	N/A
4.60E+05	4.64E+05	6.82E+04
N/A	N/A	N/A
3.48E+05	2.81E+05	1.82E+05
2.20E+07	2.43E+07	2.23E+05
2.04E+04	1.80E+04	N/A
N/A	N/A	N/A
1.28E+05	1.26E+05	N/A
9.48E+04	8.76E+04	2.60E+05
1.84E+06	1.89E+06	2.96E+05
N/A	N/A	N/A
N/A	N/A	N/A
N/A	N/A	N/A
N/A	N/A	N/A
1.36E+06	1.60E+06	N/A
N/A	N/A	N/A
N/A	N/A	N/A
7.74E+04	7.11E+04	N/A
N/A	N/A	5.85E+05
N/A	N/A	N/A
N/A	N/A	N/A
4.06E+05	3.85E+05	3.00E+06
N/A	N/A	6.78E+05
2.38E+06	2.45E+06	1.62E+04
N/A	N/A	N/A
N/A	N/A	4.37E+06

2.44E+04	2.50E+04	6.94E+03
2.32E+04	2.09E+04	1.81E+05
4.34E+05	4.54E+05	6.46E+04
N/A	N/A	2.41E+05
2.90E+07	2.96E+07	N/A
1.88E+05	2.37E+05	N/A
3.80E+05	4.48E+05	N/A
N/A	N/A	1.35E+05
N/A	N/A	N/A
1.13E+06	1.07E+06	9.15E+05
2.00E+04	1.68E+04	2.96E+04
N/A	N/A	N/A
N/A	N/A	N/A
1.35E+05	1.35E+05	3.10E+06
N/A	N/A	N/A
4.20E+05	4.71E+05	2.59E+04
1.77E+05	1.70E+05	N/A
6.55E+03	7.17E+03	5.77E+05
4.10E+05	4.61E+05	1.33E+07
1.64E+05	1.67E+05	N/A
1.80E+06	1.77E+06	1.95E+06
8.07E+04	8.02E+04	1.66E+06
1.35E+07	1.29E+07	3.68E+06
2.79E+05	2.90E+05	2.88E+05
4.65E+06	4.51E+06	9.03E+05
N/A	N/A	1.53E+05
3.66E+05	3.85E+05	N/A
N/A	N/A	2.77E+04
1.28E+05	1.38E+05	N/A
N/A	N/A	2.47E+04
7.75E+05	7.62E+05	1.34E+06
N/A	N/A	N/A
N/A	N/A	N/A
1.33E+04	2.46E+04	7.62E+04
6.67E+03	8.28E+03	N/A
N/A	N/A	7.80E+05
N/A	N/A	N/A
N/A	N/A	N/A
5.82E+04	5.13E+04	1.65E+04
N/A	N/A	N/A
1.67E+04	1.76E+04	1.45E+04
1.11E+04	1.32E+04	N/A
8.20E+05	9.55E+05	5.79E+04
N/A	N/A	N/A

1.14E+05	1.14E+05	4.75E+05
2.28E+05	1.88E+05	N/A
N/A	N/A	N/A
N/A	N/A	N/A
1.49E+04	1.53E+04	N/A
N/A	N/A	N/A
2.92E+05	2.91E+05	2.56E+05
N/A	N/A	2.25E+05
2.75E+05	2.61E+05	9.04E+05
N/A	N/A	N/A
2.09E+04	2.30E+04	1.69E+05
N/A	N/A	3.39E+05
N/A	N/A	N/A
6.26E+03	6.84E+03	N/A
N/A	N/A	2.03E+04
1.07E+05	1.08E+05	1.08E+05
N/A	N/A	N/A
N/A	N/A	N/A
9.76E+02	8.83E+02	1.03E+04
N/A	N/A	N/A
N/A	N/A	3.71E+04
N/A	N/A	4.81E+03
N/A	N/A	3.52E+04
N/A	N/A	2.46E+04
N/A	N/A	5.61E+04
N/A	N/A	2.27E+04
6.28E+04	6.24E+04	3.59E+05
5.27E+03	4.90E+03	4.47E+04
2.88E+02	2.23E+02	2.45E+04
N/A	N/A	N/A
2.30E+04	2.27E+04	2.76E+05
N/A	N/A	N/A
N/A	N/A	N/A
N/A	N/A	N/A
2.15E+04	2.33E+04	2.82E+03
1.70E+05	1.71E+05	1.81E+05
N/A	N/A	N/A
N/A	N/A	4.28E+04
N/A	N/A	N/A
2.58E+05	2.77E+05	1.62E+05
7.77E+03	9.28E+03	7.98E+03
2.03E+04	1.88E+04	1.42E+04
N/A	N/A	2.93E+05
N/A	N/A	8.41E+04

ts\24-M093\2024_09_03\Data\24-M(	s\24-M093\2024_09_03\Data\24-M0	s\24-M093\2024_09_03\Data\24-M0	0
HB-8-0.5uM-start- run-2	HB-8-0.5uM-end- run-1	HB-8-0.5uM-end- run-2	0
Peak Height	Peak Height	Peak Height	0
ic-pHILIC-7-12-24-column-D-pos.dar	ic-pHILIC-7-12-24-column-D-pos.dar	ic-pHILIC-7-12-24-column-D-pos.dar	0
3.20E+05	2.46E+05	2.09E+05	0
8.14E+04	6.19E+04	6.52E+04	0
1.26E+06	1.01E+06	9.88E+05	0
N/A	N/A	N/A	0
3.19E+05	2.82E+05	2.56E+05	0
6.09E+06	5.54E+06	4.91E+06	0
N/A	N/A	N/A	0
4.33E+06	3.61E+06	3.50E+06	0
1.26E+05	9.83E+04	1.00E+05	0
3.58E+06	3.42E+06	3.42E+06	0
3.29E+06	3.36E+06	3.04E+06	0
2.33E+06	2.03E+06	1.92E+06	0
6.90E+04	6.81E+04	9.34E+04	0
1.48E+07	1.60E+07	1.50E+07	0
3.22E+04	3.20E+04	3.24E+04	0
2.74E+05	2.22E+05	2.10E+05	0
1.08E+05	8.67E+04	8.60E+04	0
9.94E+06	1.18E+07	8.28E+06	0
N/A	N/A	N/A	0
8.20E+06	8.45E+06	8.33E+06	0
2.24E+06	2.17E+06	2.02E+06	0
N/A	N/A	N/A	0
8.87E+05	8.98E+05	8.40E+05	0
N/A	N/A	N/A	0
3.11E+06	2.78E+06	2.74E+06	0
9.39E+05	8.05E+05	7.85E+05	0
1.57E+06	1.32E+06	1.43E+06	0
6.18E+04	4.86E+04	5.04E+04	0
8.04E+04	6.99E+04	6.71E+04	0
3.43E+04	2.88E+04	2.92E+04	0
N/A	N/A	N/A	0
N/A	N/A	N/A	0
1.77E+04	1.54E+04	1.64E+04	0
N/A	N/A	N/A	0
N/A	N/A	N/A	0
5.02E+03	4.36E+03	5.43E+03	0
8.95E+05	8.49E+05	8.79E+05	0
2.34E+05	2.19E+05	1.74E+05	0
2.84E+05	2.58E+05	2.64E+05	0
2.24E+05	1.88E+05	1.93E+05	0

N/A	N/A	N/A	0
2.96E+05	3.46E+05	3.44E+05	0
8.44E+06	7.86E+06	7.78E+06	0
2.46E+06	2.15E+06	2.10E+06	0
6.21E+05	5.27E+05	5.30E+05	0
N/A	N/A	N/A	0
1.30E+07	1.25E+07	1.27E+07	0
1.02E+06	1.06E+06	1.13E+06	0
4.52E+06	4.27E+06	3.81E+06	0
3.09E+05	2.20E+05	3.06E+05	0
4.80E+06	4.50E+06	4.35E+06	0
1.95E+05	1.70E+05	1.51E+05	0
N/A	N/A	N/A	0
1.40E+05	1.11E+05	1.10E+05	0
1.36E+06	1.20E+06	1.18E+06	0
2.41E+04	1.91E+04	2.06E+04	0
7.15E+06	6.84E+06	6.92E+06	0
6.19E+06	6.58E+06	6.54E+06	0
N/A	N/A	N/A	0
5.33E+05	5.53E+05	5.75E+05	0
N/A	N/A	N/A	0
N/A	N/A	N/A	0
1.18E+06	1.03E+06	1.06E+06	0
1.31E+04	N/A	1.14E+04	0
4.26E+05	4.38E+05	3.83E+05	0
5.25E+04	3.85E+04	4.95E+04	0
N/A	N/A	N/A	0
2.85E+06	2.36E+06	2.61E+06	0
5.10E+03	4.04E+03	4.30E+03	0
8.62E+06	7.83E+06	8.30E+06	0
6.29E+06	6.26E+06	6.28E+06	0
5.71E+07	6.11E+07	5.59E+07	0
3.25E+04	2.57E+04	2.41E+04	0
2.31E+06	2.15E+06	2.17E+06	0
N/A	N/A	N/A	0
2.80E+04	2.70E+04	2.66E+04	0
3.78E+07	3.67E+07	3.86E+07	0
N/A	N/A	N/A	0
4.57E+05	4.44E+05	4.50E+05	0
2.24E+06	1.92E+06	2.02E+06	0
N/A	N/A	N/A	0
2.70E+06	2.41E+06	2.62E+06	0
1.90E+05	1.94E+05	1.83E+05	0
N/A	N/A	N/A	0

N/A	N/A	N/A	0
N/A	N/A	N/A	0
9.04E+04	7.93E+04	7.60E+04	0
N/A	N/A	1.23E+04	0
2.18E+06	2.16E+06	2.09E+06	0
N/A	N/A	N/A	0
3.46E+04	3.31E+04	3.37E+04	0
4.04E+04	3.37E+04	3.03E+04	0
8.29E+06	8.10E+06	8.23E+06	0
2.15E+05	1.94E+05	1.88E+05	0
N/A	N/A	N/A	0
6.74E+05	5.92E+05	6.06E+05	0
N/A	N/A	N/A	0
4.53E+05	3.92E+05	4.17E+05	0
8.65E+06	8.90E+06	8.23E+06	0
1.71E+06	1.71E+06	1.61E+06	0
1.68E+06	2.18E+06	2.02E+06	0
N/A	N/A	N/A	0
N/A	N/A	N/A	0
1.78E+05	1.77E+05	1.85E+05	0
1.37E+06	1.26E+06	1.39E+06	0
N/A	N/A	N/A	0
N/A	N/A	N/A	0
N/A	N/A	N/A	0
N/A	N/A	N/A	0
N/A	N/A	N/A	0
N/A	N/A	N/A	0
N/A	N/A	N/A	0
3.70E+06	3.21E+06	3.25E+06	0
8.52E+04	7.16E+04	7.24E+04	0
8.86E+04	7.43E+04	7.12E+04	0
9.35E+05	7.25E+05	7.36E+05	0
3.09E+03	2.64E+03	3.46E+03	0
4.12E+05	3.68E+05	3.86E+05	0
2.10E+05	1.70E+05	1.74E+05	0
N/A	N/A	N/A	0
1.37E+06	1.19E+06	1.29E+06	0
8.17E+05	7.26E+05	7.01E+05	0
2.61E+06	2.18E+06	2.24E+06	0
8.83E+05	7.58E+05	7.43E+05	0
N/A	N/A	N/A	0
6.41E+04	5.76E+04	5.74E+04	0
3.63E+05	2.99E+05	3.25E+05	0
5.67E+04	3.84E+04	4.21E+04	0

4.01E+06	3.87E+06	3.55E+06	0
1.19E+06	1.18E+06	1.05E+06	0
1.37E+06	1.07E+06	1.14E+06	0
0.0	0.0	0.0	#
0.0	0.0	0.0	#
ts\24-M093\2024_09_03\Data\24-M(	s\24-M093\2024_09_03\Data\24-M0	s\24-M093\2024_09_03\Data\24-M0	0
HB-8-0.5uM-start- run-2	HB-8-0.5uM-end- run-1	HB-8-0.5uM-end- run-2	0
Peak Height	Peak Height	Peak Height	0
ic-pHILIC-7-12-24-column-D-neg.dar	ic-pHILIC-7-12-24-column-D-neg.dar	ic-pHILIC-7-12-24-column-D-neg.dar	0
1.19E+05	1.31E+05	1.24E+05	0
7.69E+03	6.46E+03	8.47E+03	0
3.25E+05	3.99E+05	3.64E+05	0
N/A	N/A	N/A	0
7.16E+05	7.02E+05	6.68E+05	0
4.22E+04	4.63E+04	4.47E+04	0
N/A	N/A	N/A	0
N/A	N/A	N/A	0
8.47E+04	7.41E+04	7.25E+04	0
N/A	N/A	N/A	0
2.01E+05	2.59E+05	2.19E+05	0
2.13E+05	2.53E+05	2.46E+05	0
N/A	N/A	N/A	0
N/A	N/A	N/A	0
N/A	N/A	N/A	0
2.79E+05	2.87E+05	2.96E+05	0
3.45E+05	3.40E+05	3.39E+05	0
N/A	N/A	N/A	0
N/A	N/A	N/A	0
N/A	N/A	N/A	0
N/A	N/A	N/A	0
N/A	N/A	N/A	0
N/A	N/A	N/A	0
N/A	N/A	N/A	0
N/A	N/A	N/A	0
6.55E+05	6.84E+05	6.36E+05	0
N/A	N/A	N/A	0
N/A	N/A	N/A	0
2.92E+06	2.81E+06	2.62E+06	0
6.11E+05	5.80E+05	6.44E+05	0
1.39E+04	1.58E+04	1.58E+04	0
N/A	N/A	N/A	0
4.75E+06	5.02E+06	4.39E+06	0

8.01E+03	7.32E+03	8.21E+03	0
1.71E+05	1.86E+05	1.69E+05	0
6.71E+04	6.72E+04	6.28E+04	0
2.29E+05	2.96E+05	2.42E+05	0
N/A	N/A	N/A	0
N/A	N/A	N/A	0
N/A	N/A	N/A	0
1.58E+05	1.75E+05	1.68E+05	0
N/A	N/A	N/A	0
9.64E+05	1.00E+06	9.96E+05	0
3.40E+04	3.56E+04	3.42E+04	0
N/A	N/A	N/A	0
N/A	N/A	N/A	0
3.51E+06	4.28E+06	3.98E+06	0
N/A	N/A	N/A	0
3.23E+04	3.24E+04	3.00E+04	0
N/A	N/A	N/A	0
6.99E+05	7.26E+05	7.15E+05	0
1.54E+07	1.45E+07	1.48E+07	0
N/A	N/A	N/A	0
2.13E+06	2.27E+06	2.33E+06	0
1.59E+06	1.93E+06	1.80E+06	0
3.52E+06	4.11E+06	3.95E+06	0
3.20E+05	3.21E+05	3.42E+05	0
9.05E+05	1.18E+06	1.06E+06	0
1.57E+05	1.62E+05	1.62E+05	0
N/A	N/A	N/A	0
3.08E+04	3.45E+04	3.34E+04	0
N/A	N/A	N/A	0
3.27E+04	2.95E+04	3.09E+04	0
1.31E+06	1.52E+06	1.38E+06	0
N/A	N/A	N/A	0
N/A	N/A	N/A	0
1.09E+05	1.33E+05	1.08E+05	0
7.19E+03	6.19E+03	7.67E+03	0
7.83E+05	7.87E+05	7.81E+05	0
N/A	N/A	N/A	0
N/A	N/A	N/A	0
2.06E+04	1.95E+04	2.18E+04	0
N/A	N/A	N/A	0
1.79E+04	1.97E+04	1.73E+04	0
2.61E+02	N/A	N/A	0
5.63E+04	6.39E+04	6.26E+04	0
N/A	N/A	N/A	0

5.31E+05	5.98E+05	5.52E+05	0
N/A	N/A	N/A	0
N/A	N/A	N/A	0
N/A	N/A	N/A	0
N/A	N/A	N/A	0
4.77E+04	4.41E+04	4.58E+04	0
2.34E+05	2.49E+05	2.57E+05	0
2.21E+05	2.39E+05	2.12E+05	0
8.83E+05	1.09E+06	1.01E+06	0
N/A	N/A	N/A	0
1.96E+05	1.97E+05	1.96E+05	0
3.86E+05	4.55E+05	3.88E+05	0
N/A	N/A	N/A	0
N/A	N/A	N/A	0
1.98E+04	1.83E+04	2.08E+04	0
1.12E+05	1.27E+05	1.22E+05	0
N/A	N/A	N/A	0
N/A	N/A	N/A	0
1.19E+04	1.02E+04	1.07E+04	0
N/A	N/A	N/A	0
3.38E+04	3.60E+04	3.13E+04	0
5.54E+03	3.63E+03	3.87E+03	0
3.87E+04	3.96E+04	3.60E+04	0
2.75E+04	3.29E+04	3.27E+04	0
6.34E+04	7.43E+04	6.84E+04	0
2.50E+04	2.73E+04	2.70E+04	0
3.11E+05	3.19E+05	3.72E+05	0
4.91E+04	5.02E+04	4.96E+04	0
2.62E+04	3.30E+04	3.67E+04	0
N/A	N/A	N/A	0
2.87E+05	3.21E+05	2.80E+05	0
N/A	N/A	N/A	0
N/A	N/A	N/A	0
N/A	N/A	N/A	0
2.64E+03	3.16E+03	2.79E+03	0
1.96E+05	2.05E+05	2.17E+05	0
N/A	N/A	N/A	0
4.21E+04	4.89E+04	3.74E+04	0
N/A	N/A	N/A	0
1.74E+05	1.83E+05	1.63E+05	0
7.37E+03	7.56E+03	7.70E+03	0
1.61E+04	1.65E+04	1.93E+04	0
3.18E+05	3.32E+05	3.25E+05	0
8.87E+04	9.48E+04	9.62E+04	0

Original Filename	M093\2024_09_03\Data\2
Sample Name	401-A
	Peak Area
Acq. Method Name	LIC-7-12-24-column-D-p
Urea(61.1/44.2/RT(min):5.12)	1.52E+08
ethanolamine(62.1/44.2/RT(min):12.53)	8.08E+06
Imidazole(69/42.24/RT(min):3.61)	N/A
glycine(76.1/30.5/RT(min):8.53)	N/A
alanine(90.1/44.2/RT(min):7.79)	8.83E+07
choline(104/60/RT(min):12.15	4.16E+08
4-aminobutyrate(104.01/69/RT(min):8.27)	4.31E+05
dimethylglycine(104.02/58/RT(min):5.92)	1.19E+08
serine(106/60/RT(min):8.48)	5.13E+07
cytosine(112.1/95/RT(min):5.47)	N/A
creatinine(114/44.2/RT(min):4.53)	1.61E+08
proline(116.1/70.1/RT(min):6.38)	1.08E+09
indole(118/91/RT(min):3.58)	N/A
betaine(118.02/58/RT(min):5.33)	1.15E+09
valine(118.1/55.2/RT(min):6.08)	1.69E+07
threonine(120/74/RT(min):7.42)	1.41E+08
homoserine(120.15/44.2/RT(min):8.02)	N/A
purine(121/94/RT(min):3.62)	N/A
cysteine(122.1/59.1/RT(min):8.90)	N/A
creatine(132.003/90/RT(min):7.78)	1.06E+09
nicotinamide(123.1/80/RT(min):3.55)	5.84E+06
Imidazoleacetic acid(127.002/81/RT(min):5.54)	N/A
thymine(127.1/110/RT(min):3.37)	2.54E+05
DL-Pipecolic acid(130/84/RT(min):6.05)	5.51E+07
N-Acetylputrescine(131.001/114/RT(min):11.70)	2.56E+05
hydroxyproline(132.004/68.2/RT(min):7.66)	1.51E+07
leucine(132.1/86/RT(min):5.07)	5.69E+08
ornithine(133/70/RT(min):12.81)	4.22E+07
asparagine(133.1/74/RT(min):8.18)	9.85E+06
aspartate(134/74/RT(min):8.58)	4.49E+06
adenine(136/119/RT(min):4.39)	N/A
Methylcysteine(136.02/119.02/RT(min):5.49)	N/A
homocysteine(136.12/90.1/RT(min):8.97)	N/A
methylnicotinamide(137.001/94/RT(min):14.07)	7.25E+06
histidinol(142.1/95/RT(min):10.63)	N/A
lysine(147/67/RT(min):14.08)	1.91E+07
glutamine(147.1/84.1/RT(min):8.05)	5.44E+08
O-acetyl-L-serine(148/106/RT(min):4.87)	8.80E+05
glutamate(148.1/84.1/RT(min):8.21)	6.61E+07
methionine(150.1/133/RT(min):5.46)	1.17E+08

guanine(152.2/110/RT(min):6.33)	N/A
histidine(156.1/110.1/RT(min):8.90)	N/A
carnitine(162.1/103/RT(min):6.65)	6.46E+08
Methionine sulfoxide(166/74/RT(min):7.00)	3.53E+07
phenylalanine(166.1/103/RT(min):4.63)	1.98E+08
Pyridoxamine(169/134/RT(min):6.93)	N/A
pyridoxine(170/134/RT(min):3.65)	6.95E+05
1-Methyl-Histidine(170.1/124/RT(min):7.52)	8.37E+07
N-acetyl-L-ornithine(175/115.1/RT(min):8.08)	N/A
arginine(175.02/60/RT(min):14.59)	2.13E+08
citrulline(176/159/RT(min):8.66)	5.58E+08
N-carbamoyl-L-aspartate(177.05/74/RT(min):10.28)	3.89E+04
glucosamine(180/162/RT(min):7.52)	N/A
tyrosine(182.1/77/RT(min):6.64)	2.71E+07
Phosphorylcholine(184.001/125/RT(min):8.38)	1.11E+07
3-phospho-serine(186/88/RT(min):10.14)	N/A
N6-Acetyl-L-lysine(189.001/84.2/RT(min):7.97)	2.69E+06
Acetyllysine(189.002/84/RT(min):7.97)	2.09E+06
N-acetyl-glutamine(189.1/130/RT(min):4.96)	7.62E+06
N-acetyl-glutamate (190.1/84.1/RT(min):7.89)	3.71E+06
Ng_Ng-dimethyl-L-arginine(203/70/RT(min):12.15)	2.34E+07
Acetylcarnitine DL(204/85/RT(min):5.17)	4.00E+08
tryptophan(205/146/RT(min):5.74)	4.19E+08
Kynurenine(209/146/RT(min):5.17)	5.14E+06
N-acetyl-glucosamine(222/138/RT(min):5.74)	1.53E+06
Flavone(223/121/RT(min):2.42)	N/A
cystathionine(223/134/RT(min):9.64)	1.35E+06
5-methoxytryptophan(235/176/RT(min):5.21)	2.15E+05
Cystine(241.002/74/RT(min):9.08)	5.71E+06
cytidine(244.1/112/RT(min):5.95)	5.97E+07
biotin(245.1/227/RT(min):3.79)	N/A
deoxyadenosine(252/136/RT(min):3.73)	N/A
D-glucosamine-6-phosphate(260/126/RT(min):*9.99)	N/A
thiamine(265/122/RT(min):12.39)	1.93E+07
S-ribosyl-L-homocysteine_pos(268/88/RT(min):4.15)	N/A
deoxyguanosine(268.1/152/RT(min):5.60)	N/A
adenosine(268.15/136.1/RT(min):4.20)	1.30E+06
1-Methyladenosine(281.8/150/RT(min):2.80)	N/A
guanosine(284.1/135/RT(min):6.55)	N/A
L-arginino-succinate(291/70/RT(min):9.96)	3.03E+06
S-methyl-5-thioadenosine(298/136/RT(min):2.55)	N/A
7-methylguanosine(298.002/166/RT(min):6.95)	N/A
dCMP(308/112/RT(min):8.67)	N/A
glutathione (308.1/162/RT(min):7.8)	2.89E+06

dTMP(323/81/RT(min):6.99)	N/A
CMP(324/112/RT(min):9.44)	N/A
UMP(325/97/RT(min):8.91)	N/A
dAMP(332.1/136/RT(min):6.78)	N/A
Nicotinamide ribotide(335/123/RT(min):8.71)	N/A
aminoimidazole carboxamide ribonucleotide(339/110/RT(min):8.74)	N/A
thiamine-phosphate(345.2/122/RT(min):7.62)	5.91E+06
dGMP(348.1/135/RT(min):9.31)	N/A
AMP(348.15/136/RT(min):7.87)	3.63E+05
IMP(349/137/RT(min):9.31)	N/A
S-adenosyl-L-methioninamine(355/250/RT(min):9.1)	N/A
GMP(364/152/RT(min):10.18)	1.83E+04
xanthosine-5-phosphate(365/97/RT(min):11.18)	N/A
riboflavin(377/243/RT(min):3.94)	8.60E+05
S-adenosyl-L-homoCysteine_pos(385.1/136/RT(min):6.97)	3.39E+06
S-adenosyl-L-methionine(399.1/250/RT(min):9.1)	1.15E+07
folate(442/295/RT(min):10.47)	7.61E+04
7_8-dihydrofolate(444.2/178/RT(min):9.86)	1.40E+05
Diiodothyronine(525.5/352.8/RT(min):2.40)	N/A
glutathione disulfide_pos(613/231/RT(min):10.30)	2.15E+05
NAD+_pos(664.1/428/RT(min):7.88)	N/A
NADH(666.1/514/RT(min):7.21)	N/A
NADP+_pos(744.2/136/RT(min):10.56)	N/A
NADPH(746.15/729/RT(min):10.90)	N/A
coenzyme A_pos(768/261/RT(min):8.00)	N/A
FAD(786/348/RT(min):6.25)	1.63E+05
acetyl-CoA_pos(810/303/RT(min):7.39)	N/A
sarcosine(90.04/44.1/RT(min):*7.21)	N/A
13C-Proline1(122.00/75.00/RT(min):6.38)	1.14E+07
13C-Alanine-1(93.94/47.00/RT(min):7.79)	1.91E+05
13C-Serine-1(110.05/63.00/RT(min):8.48)	3.74E+05
13C-Valine-1(123.93/77.10/RT(min):6.08)	4.07E+06
13C-Glycine-1(78.91/32.00/RT(min):8.53)	1.58E+04
13C-Cystine-1(248.89/155.90/RT(min):9.08)	7.95E+05
13C-Glutamic acid-1(154.05/89.00/RT(min):8.22)	9.70E+05
13C-Histidine-1(164.98/117.90/RT(min):8.98)	N/A
13C-Leucine-1(138.99/92.00/RT(min):5.07)	5.56E+06
13C-Methionine-1(155.90/138.00/RT(min):5.46)	3.03E+06
13C-Phenylalanine-1(176.01/128.90/RT(min):4.60)	1.96E+07
13C-Tyrosine(192.01/174.00/RT(min):6.64)	9.88E+06
13C valine-1(124.00/59.00/RT(min):6.08)	N/A
13C tyrosine(192.00/83.00/RT(min):6.64)	7.30E+05
13 C phenylalanine(176.00/111.00/RT(min):4.60)	2.89E+06
13C lysine(155.00/90.10/RT(min):14.08)	7.13E+05

Adenylosuccinate-1(464/252/RT(min): 11.58)		N/A
Isoleucine(132.1/86/RT(min):5.33)		1.84E+08
13C-IsoLeucine-1(138.99/92.00/RT(min):5.33)		3.82E+06
	0.0	0.0
	0.0	0.0
Original Filename		M093\2024_09_03\Data\2
Sample Name		401-A
		Peak Area
Acq. Method Name		LIC-7-12-24-column-D-n
glycolate(75/45.2/RT(min):2.90)		5.16E+06
pyruvate(87/43/RT(min):3.55)		8.90E+06
lactate(89/43.2/RT(min):4.09)		4.21E+08
glycerate(105/75/RT(min):5.81)		1.47E+06
uracil(111.05/42.1/RT(min):4.00)		N/A
fumarate(115/71/RT(min):9.91)		5.02E+06
Maleic acid(115.03/71.03/RT(min):4.45)		3.25E+05
2-keto-isovalerate(115.05/71.05/RT(min):2.6)		N/A
succinate(117/73/RT(min):9.10)		4.30E+06
Methylmalonic acid(117.002/73.1/RT(min):8.35)		N/A
nicotinate(122/78/RT(min):3.60)		6.75E+05
taurine(124/80/RT(min):7.72)		2.85E+08
Pyroglutamic acid(128/82.1/RT(min):4.60)		2.80E+05
Citraconic acid(129.003/85.1/RT(min):4.35)		N/A
N-Acetyl-L-alanine(130/88/RT(min):3.62)		7.19E+05
Hydroxyisocaproic acid(131.006/85.1/RT(min):2.30)		5.03E+05
malate(133/115/RT(min):9.78)		4.67E+07
hypoxanthine(135/92/RT(min):4.92)		N/A
anthranilate/p-aminobenzoate(136/92/RT(min):4.8)		N/A
p-hydroxybenzoate(137/93/RT(min):5.07)		N/A
Carbamoyl phosphate(140/79/RT(min):9.47)		N/A
a-ketoglutarate(145/101/RT(min):9.42)		2.14E+07
Phenylpropiolic acid(145.004/101/RT(min):2.34)		N/A
2-Hydroxy-2-methylbutanedioic acid(147.001/85.1/RT(min):)		N/A
3-methylphenylacetic acid(149.002/105/RT(min):2.34*)		6.65E+05
xanthine (151/108/RT(min):5.73)		N/A
Hydroxyphenylacetic acid(151.004/107/RT(min):2.60)		N/A
2_3-dihydroxybenzoic acid(153/109/RT(min):3.62)		N/A
orotate(155/111/RT(min):4.71)		7.72E+06
dihydroorotate(157/113/RT(min):5.20)		N/A
allantoin(157.05/114/RT(min):7.19)		1.89E+07
Aminoadipic acid(160.001/116/RT(min):8.42)		N/A
Indole-3-carboxylic acid(160.002/116/RT(min):4.24)		N/A

phenylpyruvate(163/91/RT(min):2.38)	2.71E+05
Phenyllactic acid(165.006/103.1/RT(min):2.30)	1.40E+05
quinolinate(166/122/RT(min):9.13)	4.18E+06
phosphoenolpyruvate(167/79/RT(min):11.22)	N/A
Uric acid(167.001/124/RT(min):6.71)	4.34E+08
dihydroxy-acetone-phosphate(169/79/RT(min):9.03)	N/A
D-glyceraldehdye-3-phosphate(169.05/97/RT(min):9.03)	N/A
sn-glycerol-3-phosphate(171/79/RT(min):8.41)	N/A
shikimate(173/93/RT(min):7.70)	N/A
aconitate(173.05/85/RT(min):11.22)	4.50E+06
allantoate(175/132/RT(min):8.35)	3.36E+05
Ascorbic acid(175.001/87/RT(min):4.30)	N/A
2-Isopropylmalic acid(175.002/115/RT(min):6.14)	N/A
N-carbamoyl-L-aspartate_neg(175.03/132/RT(min):10.28)	1.18E+06
glucono-D-lactone(177/129/RT(min):5.29)	N/A
myo-inositol(179/161/RT(min):9.34)	8.47E+06
hydroxyphenylpyruvate(179.05/107/RT(min):3.59)	1.07E+06
homocysteic acid(182/80/RT(min):9.63)	5.61E+04
4-Pyridoxic acid(182.003/138/RT(min):2.26)	3.35E+06
3-phosphoglycerate(185/97/RT(min):10.87)	N/A
Indoleacrylic acid(186/142.03/RT(min):3.60)	9.25E+06
Kynurenic acid(188/144/RT(min):2.32)	7.22E+05
citrate-isocitrate(191/111/RT(min):12)	4.13E+08
isocitrate (191.02/117/RT(min):12.12)	9.35E+05
citrate (191.05/87/RT(min):12.12)	1.40E+08
D-gluconate(195/129/RT(min):7.59)	N/A
Xanthurenic acid(204.001/160/RT(min):5.65)	4.27E+06
lipoate(205/171/RT(min):2.22)	N/A
D-glucarate(209/85/RT(min):10.20)	2.82E+05
deoxyribose-phosphate(213/79/RT(min):8.45)	N/A
pantothenate(218/146/RT(min):3.59)	4.35E+06
deoxyuridine(227/184/RT(min):3.82)	N/A
ribose-phosphate(229/79/RT(min):9.50)	N/A
thymidine(241/125/RT(min):3.46)	1.20E+05
uridine(243/200/RT(min):4.70)	1.03E+05
deoxyinosine(251/135/RT(min):4.55)	N/A
hexose-phosphate(259/79/RT(min):9.7*)	N/A
glucose-1-phosphate(259.01/241/RT(min):9.62)	N/A
glucose-6-phosphate(259.02/199/RT(min):10.38)	2.03E+05
fructose-6-phosphate(259.03/169/RT(min):9.72)	N/A
inosine(267/135/RT(min):5.41)	1.19E+05
6-phospho-D-gluconate(275/97/RT(min):11.53)	2.29E+05
xanthosine(283/151/RT(min):6.41)	1.85E+07
D-sedoheptulose-1-7-phosphate(289/97/RT(min):*9.81)	N/A

N-acetyl-glucosamine-1-phosphate(300/79/RT(min):8.6)	7.99E+05
glutathione_neg(306/143/RT(min):7.9)	6.17E+05
dUMP_neg(307/195/RT(min):7.78)	N/A
dTMP_neg(321/195/RT(min):6.99)	N/A
cyclic-AMP(328/134.05/RT(min):4.45)	N/A
fructose-1_6-bisphosphate(339/97/RT(min):12.10)	N/A
sucrose(341/179/RT(min):*8.09)	5.55E+05
trehalose(341/179/RT(min):8.82)	N/A
S-adenosyl-L-homocysteine_neg(383.1/134/RT(min):6.97)	1.99E+06
dCDP_neg(386/159/RT(min):10.01)	N/A
Deoxycholic acid(391.202/345.2/RT(min):2.11)	1.87E+05
dTDP_neg(401/159/RT(min):8.87)	N/A
CDP_neg(402/384/RT(min):10.80)	N/A
UDP_neg(403/159/RT(min):10.38)	N/A
Thiamine pyrophosphate(423.1/302/RT(min):8.54)	N/A
ADP_neg(426.1/159/RT(min):9.38)	N/A
dGDP_neg(426.12/159/RT(min):10.67)	N/A
IDP_neg(427/159/RT(min):10.65)	N/A
GDP_neg(442/159/RT(min):11.48)	N/A
CDP-ethanolamine(445/273/RT(min):9.43)	N/A
dCTP_neg(466/159/RT(min):11.23)	N/A
dUTP_neg(467/159/RT(min):10.76)	N/A
dTTP_neg(481/159/RT(min):10.24)	N/A
CTP_neg(482/384/RT(min):12.02)	N/A
UTP_neg(483/159/RT(min):11.60)	N/A
dATP_neg(490/159/RT(min):9.95)	6.47E+02
Taurodeoxycholic acid(498.2/124/RT(min):2.0)	5.58E+05
ATP_neg(506.1/159/RT(min):10.72)	N/A
dGTP(506.12/159/RT(min):12.41)	N/A
GTP_neg(522/424/RT(min):12.59)	N/A
UDP-D-glucose(565/323/RT(min):10.05)	N/A
UDP-D-glucuronate(579/403/RT(min):12.37)	N/A
ADP-D-glucose(588/346/RT(min):8.70)	N/A
guanosine 5-diphosphate_3-diphosphate(602/504/RT(min):)	N/A
UDP-N-acetyl-glucosamine(606/385/RT(min):9.04)	N/A
glutathione disulfide_neg(611/306/RT(min):10.3*)	2.42E+05
cyclic bis(3->5) dimeric GMP(689/344/RT(min):10.78**)	N/A
coenzyme A_neg(766/408/RT(min):)	N/A
acetyl-CoA_neg(808/408/RT(min):7.39)	N/A
2-hydroxygluterate(147.1/128.7/RT(min):9.18)	3.09E+06
13C-Serine(107.9/77.0/RT(min):8.49)	1.30E+05
13C-Methionine(153.9/48.1/RT(min):5.46)	2.60E+05
13C-taurine(126/80/RT(min):7.73)	N/A
13C-Xanthine(153/43/RT(min):5.73)	N/A

M093\2024_09_03\Data\2	M093\2024_09_03\Data\2	M093\2024_09_03\Data\	M093\2024_09_03\Data\2
402-A	403-A	433-B	434-B
Peak Area	Peak Area	Peak Area	Peak Area
LIC-7-12-24-column-D-p	LIC-7-12-24-column-D-p	LIC-7-12-24-column-D-p	LIC-7-12-24-column-D-p
1.60E+08	1.51E+08	9.85E+07	1.03E+08
8.36E+06	1.34E+07	3.40E+06	2.62E+06
N/A	N/A	N/A	N/A
N/A	N/A	N/A	N/A
9.79E+07	9.54E+07	7.46E+07	7.34E+07
4.41E+08	6.50E+08	2.32E+08	1.68E+08
2.48E+05	1.62E+06	1.70E+05	1.97E+05
1.36E+08	1.23E+08	1.05E+08	1.07E+08
6.11E+07	7.60E+07	4.60E+07	4.63E+07
N/A	N/A	N/A	N/A
1.67E+08	1.77E+08	1.56E+08	1.51E+08
1.12E+09	1.19E+09	1.03E+09	1.00E+09
N/A	N/A	N/A	N/A
1.23E+09	1.32E+09	1.16E+09	1.35E+09
1.95E+07	1.63E+07	1.47E+07	1.41E+07
1.60E+08	1.64E+08	2.00E+08	1.67E+08
N/A	N/A	N/A	N/A
N/A	N/A	N/A	N/A
N/A	N/A	N/A	N/A
1.25E+09	1.24E+09	1.08E+09	1.15E+09
8.68E+06	1.29E+07	2.64E+06	4.85E+06
N/A	N/A	N/A	N/A
3.27E+05	3.54E+05	1.86E+05	2.20E+05
5.76E+07	4.78E+07	3.80E+07	5.37E+07
3.20E+05	3.01E+05	2.14E+05	2.54E+05
2.26E+07	2.51E+07	3.11E+07	2.11E+07
5.72E+08	5.86E+08	3.57E+08	3.47E+08
3.55E+07	2.94E+07	2.80E+07	2.45E+07
9.02E+06	1.00E+07	5.21E+06	4.06E+06
3.88E+06	1.11E+07	2.46E+06	2.53E+06
N/A	N/A	N/A	N/A
N/A	N/A	N/A	N/A
N/A	N/A	N/A	N/A
1.21E+07	4.79E+06	6.41E+06	9.47E+06
N/A	N/A	N/A	N/A
1.81E+07	2.09E+07	1.47E+07	1.39E+07
5.52E+08	6.33E+08	2.68E+08	2.94E+08
1.02E+06	1.21E+06	6.85E+05	7.84E+05
6.35E+07	9.97E+07	2.65E+07	2.48E+07
1.19E+08	1.19E+08	8.84E+07	8.11E+07

N/A	N/A	N/A	N/A
N/A	N/A	N/A	N/A
8.46E+08	7.48E+08	5.64E+08	5.71E+08
3.40E+07	4.42E+07	2.57E+07	3.43E+07
2.10E+08	2.24E+08	1.57E+08	1.54E+08
N/A	N/A	N/A	N/A
1.19E+06	3.65E+05	6.35E+05	3.58E+05
1.00E+08	1.03E+08	1.23E+08	1.03E+08
N/A	N/A	N/A	N/A
2.33E+08	2.78E+08	2.08E+08	2.01E+08
5.85E+08	5.95E+08	6.46E+08	6.97E+08
4.87E+04	5.40E+04	8.44E+04	8.13E+04
N/A	N/A	N/A	N/A
3.22E+07	3.21E+07	2.51E+07	2.34E+07
1.32E+07	1.45E+07	5.47E+07	5.47E+07
N/A	N/A	N/A	N/A
3.07E+06	2.63E+06	1.03E+06	1.27E+06
2.44E+06	2.20E+06	9.11E+05	1.08E+06
9.59E+06	8.74E+06	3.89E+06	6.18E+06
4.15E+06	5.37E+06	2.50E+06	2.97E+06
2.74E+07	3.15E+07	1.98E+07	2.07E+07
5.24E+08	4.37E+08	2.96E+08	4.88E+08
4.26E+08	4.38E+08	3.04E+08	3.36E+08
5.56E+06	5.78E+06	7.21E+08	6.68E+08
1.42E+06	2.14E+06	8.48E+05	5.73E+05
N/A	N/A	N/A	N/A
2.35E+06	3.76E+06	1.79E+06	1.53E+06
2.91E+05	3.42E+05	2.31E+05	3.36E+05
6.02E+06	6.80E+06	3.53E+06	5.89E+06
8.39E+07	1.07E+08	7.08E+07	5.66E+07
N/A	N/A	N/A	N/A
N/A	N/A	N/A	N/A
N/A	N/A	N/A	N/A
2.18E+07	2.72E+07	8.51E+06	9.18E+06
N/A	N/A	N/A	N/A
N/A	N/A	N/A	N/A
2.56E+06	6.03E+05	6.36E+05	5.85E+06
N/A	N/A	N/A	N/A
N/A	N/A	N/A	N/A
4.38E+06	7.06E+06	5.64E+06	5.93E+06
N/A	N/A	N/A	N/A
N/A	N/A	N/A	N/A
N/A	N/A	N/A	N/A
3.81E+06	4.50E+06	4.70E+06	1.08E+07

N/A	N/A	N/A	N/A
N/A	N/A	N/A	N/A
N/A	N/A	7.02E+05	1.19E+06
N/A	N/A	5.54E+05	7.04E+05
N/A	N/A	N/A	N/A
N/A	N/A	N/A	N/A
9.77E+06	6.61E+06	6.17E+06	7.23E+06
N/A	N/A	N/A	N/A
3.39E+05	5.23E+05	2.59E+08	5.13E+08
N/A	N/A	6.37E+06	1.47E+07
N/A	N/A	N/A	N/A
2.51E+04	1.06E+05	1.79E+07	2.38E+07
N/A	N/A	N/A	N/A
1.15E+06	2.05E+06	1.15E+06	1.34E+06
4.18E+06	3.27E+06	3.49E+06	5.48E+06
1.37E+07	2.43E+07	1.95E+06	2.77E+06
2.78E+05	7.49E+04	2.56E+05	1.19E+05
1.92E+05	2.29E+05	2.07E+05	1.16E+05
N/A	N/A	N/A	N/A
4.18E+05	2.27E+06	2.95E+06	2.74E+06
N/A	N/A	N/A	N/A
N/A	N/A	N/A	N/A
N/A	N/A	N/A	N/A
N/A	N/A	N/A	N/A
N/A	N/A	N/A	N/A
1.16E+05	3.27E+05	1.47E+05	1.67E+05
N/A	N/A	N/A	N/A
N/A	N/A	N/A	N/A
1.08E+07	1.00E+07	1.18E+07	1.23E+07
1.56E+05	1.65E+05	1.47E+05	1.42E+05
3.36E+05	2.86E+05	3.02E+05	3.05E+05
4.02E+06	3.61E+06	4.01E+06	3.87E+06
1.58E+04	2.10E+04	1.23E+04	1.58E+04
8.65E+05	7.12E+05	8.01E+05	6.72E+05
8.83E+05	7.46E+05	5.82E+05	4.41E+05
N/A	N/A	N/A	N/A
4.84E+06	5.04E+06	3.46E+06	3.69E+06
2.52E+06	2.26E+06	2.24E+06	2.07E+06
1.79E+07	1.60E+07	1.40E+07	1.46E+07
8.93E+06	8.27E+06	6.06E+06	6.24E+06
N/A	N/A	N/A	N/A
6.46E+05	6.55E+05	3.68E+05	4.21E+05
2.52E+06	2.17E+06	1.97E+06	1.99E+06
7.35E+05	6.78E+05	6.71E+05	5.89E+05

N/A	N/A	1.97E+05	2.49E+05
1.75E+08	1.53E+08	1.34E+08	1.24E+08
3.08E+06	2.81E+06	2.78E+06	2.76E+06
0.0	0.0	0.0	0.0
0.0	0.0	0.0	0.0
M093\2024_09_03\Data\2	M093\2024_09_03\Data\2	M093\2024_09_03\Data\	M093\2024_09_03\Data\2
402-A	403-A	433-B	434-B
Peak Area	Peak Area	Peak Area	Peak Area
LIC-7-12-24-column-D-n	LIC-7-12-24-column-D-n	LIC-7-12-24-column-D-n	LIC-7-12-24-column-D-n
4.81E+06	5.14E+06	4.70E+06	4.84E+06
9.82E+06	5.48E+06	7.59E+06	7.95E+06
4.98E+08	3.74E+08	7.15E+08	7.13E+08
2.19E+06	2.37E+06	4.65E+05	6.10E+05
N/A	N/A	N/A	N/A
4.09E+06	3.91E+06	1.32E+06	1.26E+06
2.60E+05	2.21E+05	1.85E+05	1.93E+05
N/A	N/A	N/A	N/A
4.77E+06	5.19E+06	4.16E+06	3.55E+06
N/A	N/A	N/A	N/A
3.98E+06	1.61E+06	8.69E+05	8.48E+05
2.90E+08	3.99E+08	1.53E+08	1.72E+08
4.22E+05	4.61E+05	2.76E+05	3.62E+05
N/A	N/A	N/A	N/A
7.84E+05	9.50E+05	5.95E+05	5.49E+05
7.04E+05	6.36E+05	4.93E+05	4.82E+05
3.71E+07	3.60E+07	1.18E+07	1.22E+07
N/A	N/A	N/A	N/A
N/A	N/A	N/A	N/A
N/A	N/A	N/A	N/A
N/A	N/A	N/A	N/A
1.86E+07	1.90E+07	1.37E+07	1.13E+07
N/A	N/A	N/A	N/A
N/A	N/A	N/A	N/A
5.17E+05	6.26E+05	3.42E+05	4.45E+05
N/A	N/A	N/A	N/A
N/A	N/A	N/A	N/A
N/A	N/A	N/A	N/A
8.28E+06	9.71E+06	3.26E+06	5.23E+06
N/A	N/A	N/A	N/A
3.27E+07	3.16E+07	1.18E+07	1.52E+07
N/A	N/A	N/A	N/A
N/A	N/A	N/A	N/A

2.47E+05	1.52E+05	2.51E+05	1.66E+05
2.46E+05	2.05E+05	7.92E+04	8.48E+04
4.35E+06	6.16E+06	4.69E+06	4.21E+06
N/A	N/A	N/A	N/A
5.10E+08	6.53E+08	3.34E+08	3.75E+08
N/A	N/A	3.33E+06	4.71E+06
N/A	N/A	5.09E+06	7.43E+06
N/A	N/A	N/A	N/A
N/A	N/A	N/A	N/A
6.78E+06	5.83E+06	2.27E+07	2.71E+07
4.59E+05	2.86E+05	1.41E+05	1.86E+05
N/A	N/A	N/A	N/A
N/A	N/A	N/A	N/A
1.78E+06	1.68E+06	1.21E+06	1.30E+06
N/A	N/A	N/A	N/A
6.91E+06	1.50E+07	3.17E+06	3.01E+06
1.65E+06	7.45E+05	1.20E+06	5.71E+05
1.02E+05	1.42E+05	5.64E+04	5.88E+04
5.57E+06	5.84E+06	3.38E+06	2.44E+06
N/A	N/A	2.63E+06	3.98E+06
8.94E+06	9.73E+06	5.99E+06	1.59E+07
8.58E+05	8.75E+05	9.72E+05	2.21E+06
6.17E+08	5.33E+08	6.57E+08	6.87E+08
1.58E+06	1.21E+06	5.34E+06	6.96E+06
1.93E+08	1.75E+08	2.28E+08	2.24E+08
N/A	N/A	N/A	N/A
4.73E+06	4.81E+06	3.71E+06	3.99E+06
N/A	N/A	N/A	N/A
7.88E+05	5.57E+05	2.21E+06	2.30E+06
N/A	N/A	N/A	N/A
6.07E+06	6.01E+06	3.65E+06	3.37E+06
N/A	N/A	N/A	N/A
N/A	N/A	N/A	N/A
7.93E+04	1.97E+05	2.39E+05	1.54E+05
1.26E+05	1.79E+05	1.75E+04	2.27E+04
N/A	N/A	N/A	N/A
N/A	N/A	N/A	N/A
N/A	N/A	N/A	N/A
3.22E+05	4.55E+05	7.65E+05	1.04E+06
N/A	N/A	N/A	N/A
2.32E+05	3.15E+05	8.08E+04	2.09E+05
1.97E+05	1.97E+05	2.78E+05	3.48E+05
2.19E+07	2.28E+07	1.19E+06	1.61E+06
N/A	N/A	N/A	N/A

1.23E+06	2.26E+06	7.79E+05	8.50E+05
9.33E+05	1.17E+06	1.74E+06	2.95E+06
N/A	N/A	N/A	N/A
N/A	N/A	N/A	N/A
N/A	N/A	3.31E+05	2.32E+05
N/A	N/A	N/A	N/A
2.35E+06	8.98E+06	8.98E+05	5.88E+05
N/A	N/A	N/A	N/A
2.30E+06	1.77E+06	2.03E+06	3.34E+06
N/A	N/A	N/A	N/A
2.37E+05	1.87E+05	1.28E+05	1.03E+05
N/A	N/A	N/A	N/A
N/A	N/A	N/A	N/A
N/A	N/A	1.11E+05	2.02E+05
N/A	N/A	N/A	N/A
N/A	N/A	1.87E+06	2.68E+06
N/A	N/A	N/A	N/A
N/A	N/A	N/A	N/A
N/A	N/A	2.33E+04	2.11E+04
N/A	N/A	N/A	N/A
N/A	N/A	N/A	N/A
N/A	N/A	N/A	N/A
N/A	N/A	N/A	N/A
N/A	N/A	N/A	N/A
N/A	N/A	N/A	N/A
N/A	N/A	N/A	9.72E+02
5.75E+05	1.14E+06	9.32E+05	2.86E+05
N/A	N/A	2.27E+05	1.84E+05
N/A	N/A	N/A	N/A
N/A	N/A	N/A	N/A
N/A	N/A	6.01E+05	2.26E+05
N/A	N/A	N/A	N/A
N/A	N/A	N/A	N/A
N/A	N/A	N/A	N/A
N/A	N/A	3.83E+05	6.98E+05
4.46E+05	2.35E+06	2.49E+06	1.53E+06
N/A	N/A	N/A	N/A
N/A	N/A	N/A	N/A
N/A	N/A	N/A	N/A
2.44E+06	3.42E+06	1.52E+06	1.74E+06
1.10E+05	9.20E+04	6.58E+04	8.52E+04
2.48E+05	2.32E+05	2.03E+05	2.01E+05
N/A	N/A	N/A	N/A
N/A	N/A	N/A	N/A

M093\2024_09_03\Data\	M093\2024_09_03\Data\	M093\2024_09_03\Data\	M093\2024_09_03\Data\
435-B	Blank-3	QC-star-run-1	QC-star-run-2
Peak Area	Peak Area	Peak Area	Peak Area
LIC-7-12-24-column-D-p	LIC-7-12-24-column-D-p	LIC-7-12-24-column-D-p	LIC-7-12-24-column-D-p
1.07E+08	N/A	1.61E+08	1.57E+08
2.67E+06	N/A	7.55E+06	6.88E+06
N/A	N/A	N/A	N/A
N/A	N/A	N/A	N/A
7.40E+07	N/A	9.20E+07	9.25E+07
2.08E+08	N/A	4.33E+08	4.06E+08
3.66E+05	N/A	5.41E+05	5.58E+05
9.06E+07	N/A	1.29E+08	1.23E+08
5.37E+07	N/A	6.58E+07	6.20E+07
N/A	N/A	N/A	N/A
1.55E+08	N/A	1.86E+08	1.70E+08
1.12E+09	N/A	1.18E+09	1.13E+09
N/A	N/A	N/A	N/A
1.22E+09	N/A	1.24E+09	1.20E+09
1.47E+07	N/A	1.86E+07	1.79E+07
1.83E+08	N/A	1.86E+08	1.88E+08
N/A	N/A	N/A	N/A
N/A	N/A	N/A	N/A
N/A	N/A	N/A	N/A
1.01E+09	N/A	1.15E+09	1.15E+09
5.79E+06	N/A	8.40E+06	7.82E+06
N/A	N/A	N/A	N/A
2.19E+05	N/A	3.56E+05	3.05E+05
4.39E+07	N/A	6.06E+07	5.66E+07
2.78E+05	N/A	3.66E+05	3.28E+05
2.76E+07	N/A	2.82E+07	2.66E+07
3.97E+08	N/A	5.33E+08	5.36E+08
3.03E+07	3.87E+05	3.50E+07	3.49E+07
5.37E+06	N/A	7.25E+06	7.24E+06
3.02E+06	N/A	5.33E+06	4.93E+06
N/A	N/A	N/A	N/A
N/A	N/A	N/A	N/A
N/A	N/A	N/A	N/A
1.46E+07	N/A	1.15E+07	1.07E+07
N/A	N/A	N/A	N/A
1.80E+07	N/A	2.03E+07	1.90E+07
3.07E+08	N/A	3.98E+08	4.08E+08
6.85E+05	N/A	1.00E+06	1.03E+06
3.17E+07	N/A	4.69E+07	4.55E+07
9.45E+07	N/A	1.15E+08	1.15E+08

N/A	N/A	N/A	N/A
N/A	N/A	N/A	N/A
5.73E+08	N/A	6.97E+08	7.35E+08
3.66E+07	N/A	3.72E+07	3.82E+07
1.60E+08	N/A	2.14E+08	2.03E+08
N/A	N/A	N/A	N/A
7.68E+05	N/A	8.10E+05	7.90E+05
1.05E+08	N/A	1.16E+08	1.22E+08
N/A	N/A	N/A	N/A
2.26E+08	9.10E+05	2.63E+08	2.79E+08
6.48E+08	N/A	6.99E+08	6.77E+08
8.28E+04	N/A	6.98E+04	8.78E+04
N/A	N/A	N/A	N/A
2.57E+07	N/A	3.17E+07	2.91E+07
5.58E+07	N/A	4.36E+07	4.05E+07
N/A	N/A	N/A	N/A
1.53E+06	N/A	1.80E+06	1.83E+06
1.79E+06	N/A	1.51E+06	1.53E+06
7.19E+06	N/A	8.86E+06	8.41E+06
2.28E+06	N/A	3.23E+06	3.16E+06
1.96E+07	N/A	2.92E+07	2.50E+07
3.93E+08	N/A	4.66E+08	4.56E+08
3.55E+08	N/A	4.37E+08	4.13E+08
6.58E+08	N/A	8.82E+07	8.08E+07
7.85E+05	N/A	1.30E+06	1.40E+06
N/A	N/A	N/A	N/A
1.78E+06	N/A	2.23E+06	2.30E+06
4.28E+05	N/A	3.50E+05	2.89E+05
5.75E+06	N/A	6.81E+06	6.12E+06
4.24E+07	N/A	7.89E+07	7.39E+07
N/A	N/A	N/A	N/A
N/A	N/A	N/A	N/A
N/A	N/A	N/A	N/A
1.14E+07	N/A	1.85E+07	1.74E+07
N/A	N/A	N/A	N/A
N/A	N/A	N/A	N/A
1.02E+07	N/A	5.28E+06	5.05E+06
N/A	N/A	N/A	N/A
N/A	N/A	N/A	N/A
6.61E+06	N/A	7.54E+06	7.86E+06
N/A	N/A	N/A	N/A
N/A	N/A	N/A	N/A
N/A	N/A	N/A	N/A
2.16E+07	N/A	1.13E+07	1.12E+07

N/A	N/A	N/A	N/A
N/A	N/A	N/A	N/A
9.48E+05	N/A	6.39E+05	6.87E+05
1.27E+06	N/A	5.98E+05	6.10E+05
N/A	N/A	N/A	N/A
N/A	N/A	N/A	N/A
7.66E+06	N/A	9.37E+06	9.55E+06
N/A	N/A	N/A	N/A
4.71E+08	N/A	3.70E+08	3.36E+08
4.39E+06	N/A	5.30E+06	5.47E+06
N/A	N/A	N/A	N/A
2.61E+07	N/A	1.46E+07	1.37E+07
N/A	N/A	N/A	N/A
1.14E+06	N/A	1.68E+06	1.54E+06
6.26E+06	N/A	4.46E+06	4.84E+06
2.89E+06	N/A	7.69E+06	7.34E+06
1.44E+05	N/A	2.52E+05	1.83E+05
8.62E+04	N/A	2.02E+05	2.15E+05
N/A	N/A	N/A	N/A
2.47E+06	N/A	2.18E+06	2.10E+06
N/A	N/A	N/A	N/A
N/A	N/A	N/A	N/A
N/A	N/A	N/A	N/A
N/A	N/A	N/A	N/A
N/A	N/A	N/A	N/A
1.45E+05	N/A	2.39E+05	1.61E+05
N/A	N/A	N/A	N/A
N/A	N/A	N/A	N/A
1.00E+07	N/A	1.26E+07	1.17E+07
1.66E+05	N/A	1.86E+05	1.64E+05
3.05E+05	N/A	3.65E+05	3.44E+05
3.22E+06	N/A	4.39E+06	4.31E+06
1.16E+04	N/A	1.82E+04	1.61E+04
6.34E+05	N/A	7.84E+05	8.40E+05
5.36E+05	7.01E+02	7.61E+05	6.25E+05
N/A	N/A	N/A	N/A
3.52E+06	7.02E+02	4.90E+06	5.28E+06
2.05E+06	N/A	2.89E+06	2.57E+06
1.31E+07	N/A	1.87E+07	1.76E+07
6.72E+06	N/A	8.15E+06	7.57E+06
N/A	N/A	N/A	N/A
3.82E+05	N/A	5.87E+05	5.16E+05
1.85E+06	N/A	2.66E+06	2.63E+06
6.76E+05	N/A	7.68E+05	7.80E+05

6.04E+05	N/A	2.28E+05	2.58E+05
1.11E+08	N/A	1.59E+08	1.45E+08
2.42E+06	N/A	3.11E+06	2.96E+06
0.0	0.0	0.0	0.0
0.0	0.0	0.0	0.0
M093\2024_09_03\Data\	M093\2024_09_03\Data\	M093\2024_09_03\Data\	M093\2024_09_03\Data\
435-B	Blank-3	QC-star-run-1	QC-star-run-2
Peak Area	Peak Area	Peak Area	Peak Area
LIC-7-12-24-column-D-n	LIC-7-12-24-column-D-n	LIC-7-12-24-column-D-n	LIC-7-12-24-column-D-n
4.47E+06	N/A	4.61E+06	4.58E+06
8.27E+06	N/A	6.85E+06	7.32E+06
7.98E+08	N/A	5.36E+08	5.45E+08
7.58E+05	N/A	1.21E+06	1.21E+06
N/A	N/A	N/A	N/A
2.82E+06	N/A	2.19E+06	2.50E+06
2.17E+05	N/A	4.09E+05	3.93E+05
N/A	N/A	N/A	N/A
4.69E+06	N/A	7.14E+06	7.31E+06
N/A	N/A	N/A	N/A
1.34E+06	N/A	1.73E+06	1.60E+06
1.53E+08	N/A	2.44E+08	2.38E+08
2.73E+05	N/A	3.32E+05	3.07E+05
N/A	N/A	N/A	N/A
6.38E+05	N/A	6.78E+05	6.68E+05
6.19E+05	N/A	6.10E+05	5.44E+05
2.57E+07	N/A	2.15E+07	2.37E+07
N/A	N/A	N/A	N/A
N/A	N/A	N/A	N/A
N/A	N/A	N/A	N/A
N/A	N/A	N/A	N/A
1.34E+07	N/A	1.78E+07	1.85E+07
N/A	N/A	N/A	N/A
N/A	N/A	N/A	N/A
3.94E+05	N/A	4.68E+05	5.25E+05
N/A	N/A	N/A	N/A
N/A	N/A	N/A	N/A
N/A	N/A	N/A	N/A
3.24E+06	N/A	6.17E+06	6.11E+06
N/A	N/A	N/A	N/A
2.07E+07	N/A	2.17E+07	2.19E+07
N/A	N/A	N/A	N/A
N/A	N/A	N/A	N/A

1.59E+05	N/A	2.17E+05	1.78E+05
1.39E+05	N/A	1.08E+05	1.49E+05
5.66E+06	N/A	5.09E+06	4.78E+06
N/A	N/A	N/A	N/A
3.21E+08	N/A	4.29E+08	4.06E+08
3.91E+06	N/A	2.46E+06	1.88E+06
5.66E+06	N/A	3.88E+06	2.96E+06
N/A	N/A	N/A	N/A
N/A	N/A	N/A	N/A
2.35E+07	N/A	1.54E+07	1.56E+07
2.31E+05	N/A	1.63E+05	1.70E+05
N/A	N/A	N/A	N/A
N/A	N/A	N/A	N/A
1.38E+06	N/A	1.54E+06	1.65E+06
N/A	N/A	N/A	N/A
3.45E+06	N/A	4.74E+06	4.75E+06
8.07E+05	N/A	1.02E+06	1.03E+06
6.31E+04	N/A	5.90E+04	6.82E+04
2.99E+06	N/A	3.97E+06	4.15E+06
2.10E+06	N/A	2.43E+06	2.71E+06
7.60E+06	N/A	1.03E+07	1.03E+07
1.10E+06	N/A	1.01E+06	1.02E+06
5.41E+08	3.83E+07	5.46E+08	6.09E+08
5.36E+06	9.49E+04	3.54E+06	3.61E+06
2.44E+08	1.26E+07	1.95E+08	2.06E+08
N/A	N/A	N/A	N/A
4.26E+06	N/A	4.60E+06	4.52E+06
N/A	N/A	N/A	N/A
3.52E+06	N/A	1.81E+06	1.83E+06
N/A	N/A	N/A	N/A
3.83E+06	N/A	4.21E+06	4.35E+06
N/A	N/A	N/A	N/A
N/A	N/A	N/A	N/A
1.42E+05	N/A	8.87E+04	5.60E+04
2.41E+04	N/A	6.54E+04	7.99E+04
N/A	N/A	N/A	N/A
N/A	N/A	N/A	N/A
N/A	N/A	N/A	N/A
1.06E+06	N/A	8.40E+05	7.41E+05
N/A	N/A	N/A	N/A
1.99E+05	N/A	2.12E+05	2.01E+05
3.11E+05	N/A	3.10E+05	2.11E+05
1.58E+06	N/A	1.02E+07	1.04E+07
N/A	N/A	N/A	N/A

9.68E+05	N/A	1.37E+06	1.14E+06
6.22E+06	N/A	2.55E+06	2.44E+06
N/A	N/A	N/A	N/A
N/A	N/A	N/A	N/A
1.95E+05	N/A	1.50E+05	1.58E+05
N/A	N/A	N/A	N/A
6.24E+06	N/A	2.68E+06	2.65E+06
N/A	N/A	N/A	N/A
3.19E+06	N/A	2.48E+06	2.37E+06
N/A	N/A	N/A	N/A
1.18E+05	N/A	1.53E+05	1.63E+05
N/A	N/A	N/A	N/A
N/A	N/A	N/A	N/A
1.80E+05	N/A	7.91E+04	9.03E+04
N/A	N/A	N/A	N/A
3.21E+06	N/A	1.42E+06	1.33E+06
N/A	N/A	N/A	N/A
N/A	N/A	N/A	N/A
4.48E+04	N/A	1.26E+04	1.78E+04
N/A	N/A	N/A	N/A
N/A	N/A	N/A	N/A
N/A	N/A	N/A	N/A
N/A	N/A	N/A	N/A
N/A	N/A	N/A	N/A
N/A	N/A	N/A	N/A
1.30E+03	N/A	N/A	9.71E+02
2.70E+05	N/A	6.85E+05	6.34E+05
2.15E+05	N/A	1.06E+05	1.07E+05
N/A	1.85E+03	1.75E+03	1.69E+03
N/A	N/A	N/A	N/A
6.60E+05	N/A	2.39E+05	2.35E+05
N/A	N/A	N/A	N/A
N/A	N/A	N/A	N/A
N/A	N/A	N/A	N/A
3.93E+05	N/A	2.15E+05	2.03E+05
1.91E+06	N/A	1.64E+06	1.52E+06
N/A	N/A	N/A	N/A
N/A	N/A	N/A	N/A
N/A	N/A	N/A	N/A
2.30E+06	N/A	3.10E+06	3.05E+06
9.30E+04	N/A	7.97E+04	9.01E+04
2.11E+05	N/A	2.37E+05	2.12E+05
N/A	N/A	N/A	N/A
N/A	N/A	N/A	N/A

M093\2024_09_03\Data\2	M093\2024_09_03\Data\2	rojects\24-M093\2024_09_03\Data\24-M093-p
QC-end-run-1	QC-end-run-2	HB-8-0.5uM-start- run-1
Peak Area	Peak Area	Peak Area
LIC-7-12-24-column-D-p	LIC-7-12-24-column-D-p	Zic-pHILIC-7-12-24-column-D-pos.dam
1.36E+08	1.32E+08	2.85E+06
6.20E+06	6.55E+06	1.15E+06
N/A	N/A	8.00E+06
N/A	N/A	N/A
8.14E+07	8.02E+07	3.18E+06
3.75E+08	3.99E+08	9.60E+07
4.70E+05	5.09E+05	N/A
1.13E+08	1.18E+08	5.30E+07
5.66E+07	5.53E+07	1.34E+06
N/A	N/A	3.67E+07
1.57E+08	1.70E+08	3.42E+07
1.08E+09	1.12E+09	2.89E+07
N/A	N/A	4.16E+05
1.16E+09	1.22E+09	2.11E+08
1.71E+07	1.82E+07	5.01E+05
1.82E+08	1.76E+08	3.12E+06
N/A	N/A	1.27E+06
N/A	N/A	2.93E+07
N/A	N/A	N/A
1.13E+09	1.12E+09	1.01E+08
7.89E+06	7.69E+06	1.59E+07
N/A	N/A	N/A
3.33E+05	3.35E+05	2.97E+06
5.76E+07	5.50E+07	N/A
3.04E+05	3.29E+05	3.23E+07
2.40E+07	2.39E+07	9.96E+06
5.28E+08	5.38E+08	1.68E+07
3.15E+07	3.25E+07	4.31E+06
6.03E+06	6.50E+06	8.83E+05
4.65E+06	4.52E+06	6.06E+05
N/A	N/A	N/A
N/A	N/A	N/A
N/A	N/A	2.11E+05
9.90E+06	1.06E+07	N/A
N/A	N/A	N/A
1.82E+07	1.86E+07	1.37E+05
3.69E+08	3.89E+08	1.40E+07
9.15E+05	9.85E+05	4.39E+06
4.30E+07	4.36E+07	5.75E+06
1.05E+08	1.08E+08	3.03E+06

N/A	N/A	N/A
N/A	N/A	2.18E+07
6.69E+08	6.63E+08	1.01E+08
3.46E+07	3.67E+07	3.48E+07
1.91E+08	1.94E+08	8.12E+06
N/A	N/A	N/A
6.69E+05	7.05E+05	8.60E+07
1.22E+08	1.13E+08	6.95E+07
N/A	N/A	5.38E+07
2.50E+08	2.41E+08	6.89E+06
6.69E+08	6.76E+08	5.85E+07
7.40E+04	8.21E+04	2.22E+06
N/A	N/A	N/A
2.90E+07	3.08E+07	1.55E+06
3.79E+07	3.81E+07	3.10E+07
N/A	N/A	4.07E+05
1.72E+06	1.61E+06	1.07E+08
1.47E+06	1.54E+06	9.75E+07
7.95E+06	8.45E+06	N/A
2.90E+06	3.06E+06	9.82E+06
2.64E+07	2.46E+07	N/A
4.91E+08	4.59E+08	N/A
4.00E+08	4.20E+08	1.16E+07
8.46E+07	8.12E+07	9.52E+04
1.16E+06	1.31E+06	8.42E+06
N/A	N/A	3.62E+05
2.34E+06	2.25E+06	N/A
2.97E+05	4.29E+05	2.35E+07
6.27E+06	6.27E+06	5.25E+04
7.49E+07	7.84E+07	9.27E+07
N/A	N/A	3.54E+07
N/A	N/A	2.52E+08
N/A	N/A	6.06E+05
1.70E+07	1.80E+07	5.46E+07
N/A	N/A	N/A
N/A	N/A	2.42E+05
4.86E+06	4.83E+06	4.73E+08
N/A	N/A	N/A
N/A	N/A	4.78E+06
6.88E+06	6.99E+06	1.99E+07
N/A	N/A	N/A
N/A	N/A	3.10E+07
N/A	N/A	2.69E+06
9.81E+06	9.43E+06	N/A

N/A	N/A	N/A
N/A	N/A	N/A
6.60E+05	5.80E+05	1.15E+06
5.87E+05	6.01E+05	N/A
N/A	N/A	2.81E+07
N/A	N/A	N/A
8.25E+06	8.29E+06	6.33E+05
N/A	N/A	3.79E+05
3.35E+08	3.34E+08	1.44E+08
5.13E+06	4.88E+06	2.64E+06
N/A	N/A	N/A
1.45E+07	1.36E+07	7.10E+06
N/A	N/A	N/A
1.51E+06	1.44E+06	3.45E+06
4.41E+06	4.16E+06	1.08E+08
6.97E+06	7.38E+06	2.59E+07
2.36E+05	1.71E+05	1.90E+07
1.94E+05	1.63E+05	N/A
N/A	N/A	N/A
2.20E+06	1.80E+06	1.54E+06
N/A	N/A	1.24E+07
N/A	N/A	N/A
N/A	N/A	N/A
N/A	N/A	N/A
N/A	N/A	N/A
2.78E+05	2.14E+05	N/A
N/A	N/A	N/A
N/A	N/A	N/A
1.09E+07	1.17E+07	3.99E+07
1.46E+05	1.52E+05	1.02E+06
3.15E+05	3.16E+05	9.74E+05
3.99E+06	4.06E+06	1.19E+07
1.48E+04	9.82E+03	3.89E+04
7.87E+05	7.57E+05	4.65E+06
5.83E+05	6.45E+05	3.43E+06
N/A	N/A	N/A
4.81E+06	4.71E+06	1.84E+07
2.44E+06	2.52E+06	8.29E+06
1.77E+07	1.83E+07	3.01E+07
6.99E+06	7.51E+06	1.06E+07
N/A	N/A	N/A
5.17E+05	5.30E+05	7.06E+05
2.55E+06	2.54E+06	4.21E+06
7.56E+05	7.03E+05	1.68E+06

2.47E+05	2.40E+05	3.94E+07
1.38E+08	1.46E+08	1.34E+07
2.77E+06	3.04E+06	1.27E+07
0.0	0.0	0.0
0.0	0.0	0.0
M093\2024_09_03\Data\2	M093\2024_09_03\Data\2	rojects\24-M093\2024_09_03\Data\24-M093-n
QC-end-run-1	QC-end-run-2	HB-8-0.5uM-start- run-1
Peak Area	Peak Area	Peak Area
LIC-7-12-24-column-D-n	LIC-7-12-24-column-D-n	Zic-pHILIC-7-12-24-column-D-neg.dam
5.13E+06	5.08E+06	3.69E+06
7.26E+06	7.29E+06	4.26E+04
5.73E+08	5.62E+08	1.79E+06
1.26E+06	1.12E+06	N/A
N/A	N/A	1.49E+07
2.43E+06	2.47E+06	6.60E+05
4.40E+05	3.96E+05	N/A
N/A	N/A	N/A
7.32E+06	7.33E+06	1.14E+06
N/A	N/A	N/A
1.97E+06	1.65E+06	1.02E+06
2.35E+08	2.41E+08	2.83E+06
3.54E+05	3.39E+05	N/A
N/A	N/A	N/A
7.87E+05	7.78E+05	N/A
6.43E+05	5.92E+05	1.49E+06
2.26E+07	2.36E+07	6.14E+06
N/A	N/A	N/A
N/A	N/A	N/A
N/A	N/A	N/A
N/A	N/A	N/A
1.63E+07	1.81E+07	N/A
N/A	N/A	N/A
N/A	N/A	N/A
5.42E+05	4.83E+05	N/A
N/A	N/A	5.64E+06
N/A	N/A	N/A
N/A	N/A	N/A
6.59E+06	6.39E+06	5.29E+07
N/A	N/A	4.60E+06
2.21E+07	2.33E+07	1.58E+05
N/A	N/A	N/A
N/A	N/A	1.39E+07

2.30E+05	2.04E+05	4.86E+04
1.55E+05	1.43E+05	1.16E+06
5.05E+06	5.10E+06	1.10E+06
N/A	N/A	2.13E+06
4.20E+08	4.17E+08	N/A
2.21E+06	2.72E+06	N/A
3.27E+06	3.79E+06	N/A
N/A	N/A	2.41E+06
N/A	N/A	N/A
1.64E+07	1.63E+07	1.31E+07
1.84E+05	1.22E+05	4.62E+05
N/A	N/A	N/A
N/A	N/A	N/A
1.42E+06	1.52E+06	4.52E+07
N/A	N/A	N/A
4.60E+06	4.93E+06	3.07E+05
1.08E+06	1.08E+06	N/A
6.48E+04	7.49E+04	8.70E+06
4.00E+06	4.25E+06	1.03E+08
2.69E+06	2.47E+06	N/A
1.14E+07	1.09E+07	1.17E+07
1.15E+06	1.10E+06	1.57E+07
5.81E+08	6.59E+08	8.92E+07
3.79E+06	4.09E+06	6.52E+06
2.19E+08	2.11E+08	2.87E+07
N/A	N/A	2.74E+06
4.66E+06	4.71E+06	N/A
N/A	N/A	1.95E+05
1.70E+06	1.76E+06	N/A
N/A	N/A	4.59E+05
4.64E+06	4.65E+06	7.50E+06
N/A	N/A	N/A
N/A	N/A	N/A
8.98E+04	1.39E+05	4.90E+05
6.56E+04	7.54E+04	N/A
N/A	N/A	8.18E+06
N/A	N/A	N/A
N/A	N/A	N/A
7.85E+05	7.27E+05	1.79E+05
N/A	N/A	N/A
2.26E+05	2.15E+05	1.66E+05
2.13E+05	2.61E+05	N/A
9.95E+06	1.09E+07	6.76E+05
N/A	N/A	N/A

1.29E+06	1.35E+06	7.31E+06
2.54E+06	2.31E+06	N/A
N/A	N/A	N/A
N/A	N/A	N/A
1.72E+05	1.66E+05	N/A
N/A	N/A	N/A
2.98E+06	2.92E+06	2.73E+06
N/A	N/A	1.81E+06
2.51E+06	2.39E+06	1.18E+07
N/A	N/A	N/A
1.75E+05	2.03E+05	1.38E+06
N/A	N/A	4.04E+06
N/A	N/A	N/A
6.68E+04	9.30E+04	N/A
N/A	N/A	4.45E+05
1.45E+06	1.42E+06	1.88E+06
N/A	N/A	N/A
N/A	N/A	N/A
1.24E+04	9.12E+03	1.46E+05
N/A	N/A	N/A
N/A	N/A	5.09E+05
N/A	N/A	4.51E+04
N/A	N/A	7.42E+05
N/A	N/A	4.06E+05
N/A	N/A	1.38E+06
N/A	N/A	4.84E+05
6.22E+05	6.32E+05	3.51E+06
1.07E+05	1.01E+05	1.15E+06
1.61E+03	1.44E+03	4.26E+05
N/A	N/A	N/A
2.25E+05	2.37E+05	3.19E+06
N/A	N/A	N/A
N/A	N/A	N/A
N/A	N/A	N/A
2.03E+05	2.13E+05	2.92E+04
1.65E+06	1.58E+06	2.03E+06
N/A	N/A	N/A
N/A	N/A	3.59E+05
N/A	N/A	N/A
2.94E+06	3.24E+06	2.70E+06
7.16E+04	9.46E+04	9.30E+04
2.52E+05	2.26E+05	1.65E+05
N/A	N/A	3.93E+06
N/A	N/A	1.12E+06

rojects\24-M093\2024_09_03\Data\24-M093-p	rojects\24-M093\2024_09_03\Data\24-M093-po
HB-8-0.5uM-start- run-2	HB-8-0.5uM-end- run-1
Peak Area	Peak Area
Zic-pHILIC-7-12-24-column-D-pos.dam	Zic-pHILIC-7-12-24-column-D-pos.dam
3.20E+06	2.56E+06
1.40E+06	1.15E+06
9.18E+06	6.77E+06
N/A	N/A
3.75E+06	3.32E+06
1.02E+08	8.78E+07
N/A	N/A
5.90E+07	5.19E+07
1.44E+06	1.17E+06
3.83E+07	3.58E+07
3.72E+07	3.81E+07
3.30E+07	2.79E+07
3.64E+05	3.28E+05
2.18E+08	1.95E+08
4.37E+05	4.39E+05
3.68E+06	3.08E+06
1.39E+06	1.16E+06
3.48E+07	3.38E+07
N/A	N/A
1.02E+08	1.02E+08
1.57E+07	1.69E+07
N/A	N/A
3.02E+06	2.21E+06
N/A	N/A
5.12E+07	4.55E+07
1.14E+07	9.95E+06
1.91E+07	1.41E+07
1.44E+06	1.26E+06
9.97E+05	9.49E+05
7.33E+05	5.81E+05
N/A	N/A
N/A	N/A
2.61E+05	2.12E+05
N/A	N/A
N/A	N/A
1.01E+05	1.06E+05
1.43E+07	1.37E+07
4.67E+06	4.19E+06
6.39E+06	5.89E+06
2.84E+06	2.40E+06

N/A	N/A
2.09E+07	2.24E+07
1.03E+08	1.16E+08
3.95E+07	3.52E+07
8.32E+06	7.31E+06
N/A	N/A
8.80E+07	8.51E+07
8.32E+07	8.23E+07
6.29E+07	5.79E+07
5.13E+06	4.37E+06
6.17E+07	5.69E+07
2.65E+06	2.38E+06
N/A	N/A
1.83E+06	1.56E+06
3.61E+07	3.37E+07
5.34E+05	4.62E+05
1.19E+08	1.08E+08
1.01E+08	1.02E+08
N/A	N/A
1.11E+07	1.01E+07
N/A	N/A
N/A	N/A
1.39E+07	1.26E+07
9.36E+04	N/A
9.44E+06	9.12E+06
4.90E+05	2.88E+05
N/A	N/A
2.77E+07	2.37E+07
6.52E+04	6.38E+04
1.05E+08	9.67E+07
4.07E+07	4.10E+07
2.50E+08	2.77E+08
5.40E+05	4.67E+05
5.97E+07	5.75E+07
N/A	N/A
2.95E+05	3.29E+05
4.66E+08	4.57E+08
N/A	N/A
5.77E+06	5.73E+06
3.13E+07	2.82E+07
N/A	N/A
3.38E+07	3.23E+07
3.26E+06	3.16E+06
N/A	N/A

N/A	N/A
N/A	N/A
1.37E+06	1.25E+06
N/A	N/A
3.63E+07	3.57E+07
N/A	N/A
7.97E+05	6.39E+05
4.88E+05	4.43E+05
1.71E+08	1.58E+08
2.89E+06	2.73E+06
N/A	N/A
8.21E+06	7.34E+06
N/A	N/A
4.81E+06	4.26E+06
1.19E+08	1.23E+08
2.86E+07	3.08E+07
2.10E+07	2.48E+07
N/A	N/A
N/A	N/A
2.17E+06	2.19E+06
1.49E+07	1.46E+07
N/A	N/A
N/A	N/A
N/A	N/A
N/A	N/A
N/A	N/A
N/A	N/A
N/A	N/A
4.76E+07	4.08E+07
1.01E+06	8.81E+05
1.05E+06	8.33E+05
1.31E+07	1.02E+07
3.51E+04	3.19E+04
5.64E+06	4.85E+06
4.12E+06	3.48E+06
N/A	N/A
1.87E+07	1.58E+07
8.99E+06	7.50E+06
3.41E+07	2.94E+07
1.20E+07	9.81E+06
N/A	N/A
9.03E+05	7.94E+05
4.72E+06	4.16E+06
1.06E+06	7.35E+05

4.12E+07	4.14E+07
1.31E+07	1.23E+07
1.46E+07	1.17E+07
0.0	0.0
0.0	0.0
rojects\24-M093\2024_09_03\Data\24-M093-n	rojects\24-M093\2024_09_03\Data\24-M093-ne
HB-8-0.5uM-start- run-2	HB-8-0.5uM-end- run-1
Peak Area	Peak Area
Zic-pHILIC-7-12-24-column-D-neg.dam	Zic-pHILIC-7-12-24-column-D-neg.dam
4.06E+06	4.65E+06
5.62E+04	4.99E+04
1.91E+06	2.36E+06
N/A	N/A
1.56E+07	1.66E+07
8.28E+05	8.75E+05
N/A	N/A
N/A	N/A
1.32E+06	1.24E+06
N/A	N/A
1.12E+06	1.42E+06
2.94E+06	3.22E+06
N/A	N/A
N/A	N/A
N/A	N/A
1.61E+06	1.68E+06
6.91E+06	7.38E+06
N/A	N/A
N/A	N/A
N/A	N/A
N/A	N/A
N/A	N/A
N/A	N/A
N/A	N/A
N/A	N/A
6.58E+06	7.53E+06
N/A	N/A
N/A	N/A
5.54E+07	5.75E+07
5.18E+06	5.08E+06
1.65E+05	1.79E+05
N/A	N/A
1.55E+07	1.57E+07

6.60E+04	5.98E+04
1.12E+06	1.19E+06
1.19E+06	1.17E+06
2.12E+06	2.54E+06
N/A	N/A
N/A	N/A
N/A	N/A
2.65E+06	2.80E+06
N/A	N/A
1.39E+07	1.57E+07
4.89E+05	5.21E+05
N/A	N/A
N/A	N/A
5.16E+07	6.21E+07
N/A	N/A
3.71E+05	3.60E+05
N/A	N/A
1.00E+07	1.17E+07
1.14E+08	1.16E+08
N/A	N/A
1.24E+07	1.32E+07
1.54E+07	1.80E+07
1.16E+08	1.19E+08
6.81E+06	7.22E+06
3.22E+07	3.65E+07
3.00E+06	3.04E+06
N/A	N/A
2.09E+05	2.35E+05
N/A	N/A
5.43E+05	5.56E+05
7.42E+06	8.61E+06
N/A	N/A
N/A	N/A
6.58E+05	7.77E+05
6.34E+04	5.86E+04
8.26E+06	8.52E+06
N/A	N/A
N/A	N/A
2.25E+05	2.25E+05
N/A	N/A
1.90E+05	2.14E+05
1.56E+03	N/A
6.40E+05	7.42E+05
N/A	N/A

8.14E+06	8.81E+06
N/A	N/A
N/A	N/A
N/A	N/A
N/A	N/A
9.91E+05	1.06E+06
2.58E+06	2.85E+06
1.75E+06	1.93E+06
1.21E+07	1.42E+07
N/A	N/A
1.55E+06	1.60E+06
4.56E+06	5.37E+06
N/A	N/A
N/A	N/A
4.53E+05	5.13E+05
1.93E+06	2.21E+06
N/A	N/A
N/A	N/A
1.81E+05	1.67E+05
N/A	N/A
5.25E+05	5.80E+05
4.62E+04	4.89E+04
7.99E+05	8.30E+05
4.19E+05	5.04E+05
1.54E+06	1.76E+06
5.88E+05	6.35E+05
3.24E+06	3.22E+06
1.19E+06	1.37E+06
4.64E+05	6.24E+05
N/A	N/A
3.24E+06	3.59E+06
N/A	N/A
N/A	N/A
N/A	N/A
3.27E+04	3.63E+04
2.15E+06	2.29E+06
N/A	N/A
3.77E+05	3.32E+05
N/A	N/A
2.97E+06	3.03E+06
8.75E+04	9.11E+04
1.78E+05	1.75E+05
4.25E+06	4.35E+06
1.10E+06	1.20E+06

rojects\24-M093\2024_09_03\Data\24-M093-pos.wiff (sample 19)
HB-8-0.5uM-end- run-2
Peak Area
Zic-pHILIC-7-12-24-column-D-pos.dam
2.19E+06
1.27E+06
6.37E+06
N/A
3.07E+06
8.20E+07
N/A
5.15E+07
1.11E+06
3.57E+07
3.48E+07
2.72E+07
3.62E+05
2.05E+08
4.08E+05
3.01E+06
1.11E+06
3.32E+07
N/A
9.64E+07
1.51E+07
N/A
1.99E+06
N/A
4.53E+07
9.52E+06
1.55E+07
1.33E+06
8.36E+05
5.83E+05
N/A
N/A
2.14E+05
N/A
N/A
9.14E+04
1.37E+07
3.74E+06
5.74E+06
2.91E+06

N/A
2.27E+07
1.06E+08
3.42E+07
7.25E+06
N/A
8.63E+07
8.02E+07
5.26E+07
5.23E+06
5.64E+07
2.18E+06
N/A
1.56E+06
3.47E+07
4.52E+05
1.08E+08
1.02E+08
N/A
1.02E+07
N/A
N/A
1.25E+07
1.26E+05
8.95E+06
4.28E+05
N/A
2.55E+07
6.43E+04
1.00E+08
4.17E+07
2.56E+08
5.19E+05
5.67E+07
N/A
2.99E+05
4.70E+08
N/A
5.68E+06
2.77E+07
N/A
3.27E+07
2.95E+06
N/A

N/A
N/A
1.24E+06
1.34E+05
2.83E+07
N/A
8.07E+05
4.20E+05
1.60E+08
2.73E+06
N/A
7.35E+06
N/A
4.45E+06
1.18E+08
2.98E+07
2.28E+07
N/A
N/A
2.24E+06
1.47E+07
N/A
N/A
N/A
N/A
N/A
N/A
N/A
4.26E+07
8.47E+05
8.22E+05
1.03E+07
3.72E+04
5.12E+06
3.65E+06
N/A
1.65E+07
7.67E+06
3.00E+07
9.95E+06
N/A
7.81E+05
4.20E+06
8.29E+05

4.10E+07
9.26E+06
1.22E+07

0.0
0.0

rojects\24-M093\2024_09_03\Data\24-M093-neg.wiff (sample 19)
HB-8-0.5uM-end- run-2
Peak Area
Zic-pHILIC-7-12-24-column-D-neg.dam
4.56E+06
6.31E+04
2.08E+06
N/A
1.65E+07
7.92E+05
N/A
N/A
1.17E+06
N/A
1.26E+06
3.33E+06
N/A
N/A
N/A
1.77E+06
7.12E+06
N/A
N/A
N/A
N/A
N/A
N/A
N/A
N/A
6.58E+06
N/A
N/A
5.48E+07
4.08E+06
1.76E+05
N/A
1.62E+07

7.38E+04
1.18E+06
1.19E+06
2.40E+06
N/A
N/A
N/A
2.81E+06
N/A
1.38E+07
4.87E+05
N/A
N/A
5.57E+07
N/A
3.39E+05
N/A
1.08E+07
1.22E+08
N/A
1.34E+07
1.82E+07
1.16E+08
7.31E+06
3.53E+07
3.19E+06
N/A
2.34E+05
N/A
5.66E+05
7.76E+06
N/A
N/A
6.75E+05
8.15E+04
8.32E+06
N/A
N/A
2.36E+05
N/A
1.95E+05
N/A
7.33E+05
N/A

8.55E+06
N/A
N/A
N/A
N/A
9.90E+05
2.77E+06
1.81E+06
1.35E+07
N/A
1.62E+06
4.43E+06
N/A
N/A
5.12E+05
2.06E+06
N/A
N/A
1.91E+05
N/A
5.66E+05
5.58E+04
8.33E+05
4.73E+05
1.60E+06
6.15E+05
3.63E+06
1.21E+06
6.05E+05
N/A
3.27E+06
N/A
N/A
N/A
3.02E+04
2.44E+06
N/A
3.29E+05
N/A
2.46E+06
8.85E+04
1.91E+05
4.65E+06
1.20E+06

Sample Name	Polarity
Urea(61.1/44.2/RT(min):5.12)	Positive
ethanolamine(62.1/44.2/RT(min):12.53)	Positive
alanine(90.1/44.2/RT(min):7.79)	Positive
choline(104/60/RT(min):12.15	Positive
4-aminobutyrate(104.01/69/RT(min):8.27)	Positive
dimethylglycine(104.02/58/RT(min):5.92)	Positive
serine(106/60/RT(min):8.48)	Positive
creatinine(114/44.2/RT(min):4.53)	Positive
proline(116.1/70.1/RT(min):6.38)	Positive
betaine(118.02/58/RT(min):5.33)	Positive
valine(118.1/55.2/RT(min):6.08)	Positive
threonine(120/74/RT(min):7.42)	Positive
creatine(132.003/90/RT(min):7.78)	Positive
nicotinamide(123.1/80/RT(min):3.55)	Positive
thymine(127.1/110/RT(min):3.37)	Positive
DL-Pipecolic acid(130/84/RT(min):6.05)	Positive
N-Acetylputrescine(131.001/114/RT(min):11.70)	Positive
hydroxyproline(132.004/68.2/RT(min):7.66)	Positive
leucine(132.1/86/RT(min):5.07)	Positive
ornithine(133/70/RT(min):12.81)	Positive
asparagine(133.1/74/RT(min):8.18)	Positive
aspartate(134/74/RT(min):8.58)	Positive
methylnicotinamide(137.001/94/RT(min):14.07)	Positive
lysine(147/67/RT(min):14.08)	Positive
glutamine(147.1/84.1/RT(min):8.05)	Positive
O-acetyl-L-serine(148/106/RT(min):4.87)	Positive
glutamate(148.1/84.1/RT(min):8.21)	Positive
methionine(150.1/133/RT(min):5.46)	Positive
carnitine(162.1/103/RT(min):6.65)	Positive
Methionine sulfoxide(166/74/RT(min):7.00)	Positive
phenylalanine(166.1/103/RT(min):4.63)	Positive
pyridoxine(170/134/RT(min):3.65)	Positive
1-Methyl-Histidine(170.1/124/RT(min):7.52)	Positive
arginine(175.02/60/RT(min):14.59)	Positive
citrulline(176/159/RT(min):8.66)	Positive
N-carbamoyl-L-aspartate(177.05/74/RT(min):10.28)	Positive
tyrosine(182.1/77/RT(min):6.64)	Positive
Phosphorylcholine(184.001/125/RT(min):8.38)	Positive
N6-Acetyl-L-lysine(189.001/84.2/RT(min):7.97)	Positive
Acetyllysine(189.002/84/RT(min):7.97)	Positive
N-acetyl-glutamine(189.1/130/RT(min):4.96)	Positive
N-acetyl-glutamate (190.1/84.1/RT(min):7.89)	Positive

Ng_Ng-dimethyl-L-arginine(203/70/RT(min):12.15)	Positive
Acetylcarnitine DL(204/85/RT(min):5.17)	Positive
tryptophan(205/146/RT(min):5.74)	Positive
Kynurenine(209/146/RT(min):5.17)	Positive
N-acetyl-glucosamine(222/138/RT(min):5.74)	Positive
cystathionine(223/134/RT(min):9.64)	Positive
5-methoxytryptophan(235/176/RT(min):5.21)	Positive
Cystine(241.002/74/RT(min):9.08)	Positive
cytidine(244.1/112/RT(min):5.95)	Positive
thiamine(265/122/RT(min):12.39)	Positive
adenosine(268.15/136.1/RT(min):4.20)	Positive
L-arginino-succinate(291/70/RT(min):9.96)	Positive
glutathione (308.1/162/RT(min):7.8)	Positive
UMP(325/97/RT(min):8.91)	Positive
dAMP(332.1/136/RT(min):6.78)	Positive
thiamine-phosphate(345.2/122/RT(min):7.62)	Positive
AMP(348.15/136/RT(min):7.87)	Positive
IMP(349/137/RT(min):9.31)	Positive
GMP(364/152/RT(min):10.18)	Positive
riboflavin(377/243/RT(min):3.94)	Positive
S-adenosyl-L-homoCysteine_pos(385.1/136/RT(min):6.97)	Positive
S-adenosyl-L-methionine(399.1/250/RT(min):9.1)	Positive
folate(442/295/RT(min):10.47)	Positive
7_8-dihydrofolate(444.2/178/RT(min):9.86)	Positive
glutathione disulfide_pos(613/231/RT(min):10.30)	Positive
FAD(786/348/RT(min):6.25)	Positive
13C-Proline1(122.00/75.00/RT(min):6.38)	Positive
13C-Alanine-1(93.94/47.00/RT(min):7.79)	Positive
13C-Serine-1(110.05/63.00/RT(min):8.48)	Positive
13C-Valine-1(123.93/77.10/RT(min):6.08)	Positive
13C-Glycine-1(78.91/32.00/RT(min):8.53)	Positive
13C-Cystine-1(248.89/155.90/RT(min):9.08)	Positive
13C-Glutamic acid-1(154.05/89.00/RT(min):8.22)	Positive
13C-Leucine-1(138.99/92.00/RT(min):5.07)	Positive
13C-Methionine-1(155.90/138.00/RT(min):5.46)	Positive
13C-Phenylalanine-1(176.01/128.90/RT(min):4.60)	Positive
13C-Tyrosine(192.01/174.00/RT(min):6.64)	Positive
13C tyrosine(192.00/83.00/RT(min):6.64)	Positive
13 C phenylalanine(176.00/111.00/RT(min):4.60)	Positive
13C lysine(155.00/90.10/RT(min):14.08)	Positive
Adenylosuccinate-1(464/252/RT(min): 11.58)	Positive
Isoleucine(132.1/86/RT(min):5.33)	Positive
13C-IsoLeucine-1(138.99/92.00/RT(min):5.33)	Positive

Sample Name	Polarity
glycolate(75/45.2/RT(min):2.90)	Negative
pyruvate(87/43/RT(min):3.55)	Negative
lactate(89/43.2/RT(min):4.09)	Negative
glycerate(105/75/RT(min):5.81)	Negative
fumarate(115/71/RT(min):9.91)	Negative
Maleic acid(115.03/71.03/RT(min):4.45)	Negative
succinate(117/73/RT(min):9.10)	Negative
nicotinate(122/78/RT(min):3.60)	Negative
taurine(124/80/RT(min):7.72)	Negative
Pyroglutamic acid(128/82.1/RT(min):4.60)	Negative
N-Acetyl-L-alanine(130/88/RT(min):3.62)	Negative
Hydroxyisocaproic acid(131.006/85.1/RT(min):2.30)	Negative
malate(133/115/RT(min):9.78)	Negative
a-ketoglutarate(145/101/RT(min):9.42)	Negative
3-methylphenylacetic acid(149.002/105/RT(min):2.34*)	Negative
orotate(155/111/RT(min):4.71)	Negative
allantoin(157.05/114/RT(min):7.19)	Negative
phenylpyruvate(163/91/RT(min):2.38)	Negative
Phenyllactic acid(165.006/103.1/RT(min):2.30)	Negative
quinolinate(166/122/RT(min):9.13)	Negative
Uric acid(167.001/124/RT(min):6.71)	Negative
dihydroxy-acetone-phosphate(169/79/RT(min):9.03)	Negative
D-glyceraldehdye-3-phosphate(169.05/97/RT(min):9.03)	Negative
aconitate(173.05/85/RT(min):11.22)	Negative
allantoate(175/132/RT(min):8.35)	Negative
N-carbamoyl-L-aspartate_neg(175.03/132/RT(min):10.28)	Negative
myo-inositol(179/161/RT(min):9.34)	Negative
hydroxyphenylpyruvate(179.05/107/RT(min):3.59)	Negative
homocysteic acid(182/80/RT(min):9.63)	Negative
4-Pyridoxic acid(182.003/138/RT(min):2.26)	Negative
Indoleacrylic acid(186/142.03/RT(min):3.60)	Negative
Kynurenic acid(188/144/RT(min):2.32)	Negative
citrate-isocitrate(191/111/RT(min):12)	Negative
isocitrate (191.02/117/RT(min):12.12)	Negative
citrate (191.05/87/RT(min):12.12)	Negative
Xanthurenic acid(204.001/160/RT(min):5.65)	Negative
D-glucarate(209/85/RT(min):10.20)	Negative
pantothenate(218/146/RT(min):3.59)	Negative
thymidine(241/125/RT(min):3.46)	Negative

uridine(243/200/RT(min):4.70)	Negative
glucose-6-phosphate(259.02/199/RT(min):10.38)	Negative
inosine(267/135/RT(min):5.41)	Negative
6-phospho-D-gluconate(275/97/RT(min):11.53)	Negative
xanthosine(283/151/RT(min):6.41)	Negative
N-acetyl-glucosamine-1-phosphate(300/79/RT(min):8.6)	Negative
glutathione_neg(306/143/RT(min):7.9)	Negative
cyclic-AMP(328/134.05/RT(min):4.45)	Negative
sucrose(341/179/RT(min):*8.09)	Negative
S-adenosyl-L-homocysteine_neg(383.1/134/RT(min):6.97)	Negative
Deoxycholic acid(391.202/345.2/RT(min):2.11)	Negative
UDP_neg(403/159/RT(min):10.38)	Negative
ADP_neg(426.1/159/RT(min):9.38)	Negative
GDP_neg(442/159/RT(min):11.48)	Negative
dATP_neg(490/159/RT(min):9.95)	Negative
Taurodeoxycholic acid(498.2/124/RT(min):2.0)	Negative
ATP_neg(506.1/159/RT(min):10.72)	Negative
UDP-D-glucose(565/323/RT(min):10.05)	Negative
UDP-N-acetyl-glucosamine(606/385/RT(min):9.04)	Negative
glutathione disulfide_neg(611/306/RT(min):10.3*)	Negative
2-hydroxygluterate(147.1/128.7/RT(min):9.18)	Negative
13C-Serine(107.9/77.0/RT(min):8.49)	Negative
13C-Methionine(153.9/48.1/RT(min):5.46)	Negative

401-A	402-A	403-A	433-B	434-B
RT(min)	RT(min)	RT(min)	RT(min)	RT(min)
5.1	5.1	5.1	5.1	5.1
12.7	12.6	12.6	12.6	12.6
7.6	7.6	7.6	7.6	7.6
12.0	11.8	11.8	12.1	12.1
8.1	8.1	8.1	8.1	8.1
5.8	5.8	5.8	5.8	5.8
8.3	8.3	8.3	8.3	8.3
4.5	4.5	4.5	4.5	4.5
6.3	6.3	6.3	6.3	6.3
5.3	5.3	5.3	5.3	5.3
6.0	6.0	6.0	6.0	6.0
7.3	7.3	7.3	7.2	7.3
7.6	7.6	7.6	7.6	7.6
3.8	3.8	3.8	3.8	3.8
3.8	3.8	3.8	3.8	3.8
6.0	6.0	6.0	6.0	6.0
11.7	11.7	11.7	11.8	11.8
7.5	7.5	7.5	7.5	7.5
5.0	5.0	5.0	5.0	5.0
12.9	12.8	12.9	12.8	12.8
8.0	7.9	7.9	7.9	8.0
8.2	8.2	8.2	8.2	8.2
14.6	14.5	14.6	14.6	14.6
14.0	14.0	14.0	14.0	14.0
7.9	7.9	7.9	7.8	7.9
4.3	4.4	4.4	4.5	4.4
7.9	7.8	7.9	7.8	7.8
5.4	5.4	5.4	5.4	5.4
6.5	6.5	6.5	6.5	6.5
6.7	6.8	6.8	6.7	6.7
4.7	4.7	4.6	4.6	4.7
3.8	3.8	3.8	3.8	3.8
6.8	6.7	6.7	6.9	6.9
14.6	14.6	14.6	14.6	14.6
8.5	8.5	8.5	8.5	8.5
9.7	9.8	9.8	9.7	9.7
6.6	6.6	6.6	6.6	6.6
8.2	8.2	8.2	8.2	8.2
7.8	7.8	7.8	7.7	7.7
7.8	7.8	7.8	7.7	7.8
4.4	4.4	4.4	4.4	4.4
8.0	7.9	7.9	8.0	8.0

12.1	12.1	12.1	12.0	12.0
5.1	5.1	5.1	5.1	5.1
5.6	5.6	5.6	5.6	5.6
5.1	5.1	5.1	5.1	5.1
5.8	5.8	5.8	5.8	5.8
9.4	9.3	9.3	9.3	9.3
5.3	5.3	5.3	5.2	5.3
8.6	8.6	8.6	8.6	8.6
5.9	5.9	5.9	5.8	5.9
12.3	12.2	12.2	12.3	12.3
4.2	4.2	4.2	4.2	4.2
9.6	9.6	9.6	9.6	9.6
7.6	7.6	7.5	7.5	7.5
N/A	N/A	N/A	8.4	8.4
N/A	N/A	N/A	6.7	6.7
7.4	7.4	7.4	7.4	7.4
7.4	7.4	7.4	7.4	7.4
N/A	N/A	N/A	8.8	8.8
9.7	9.7	9.8	9.7	9.7
4.0	4.0	4.0	3.9	3.9
6.9	6.9	6.9	6.9	6.9
9.0	9.0	9.0	8.9	8.9
9.8	9.9	9.9	9.8	9.9
9.4	9.3	9.3	9.3	9.3
10.4	10.4	10.4	10.3	10.3
6.0	6.1	6.1	6.0	6.0
6.3	6.3	6.3	6.3	6.3
7.6	7.6	7.6	7.6	7.6
8.3	8.3	8.3	8.3	8.3
6.0	6.0	6.0	6.0	6.0
8.3	8.3	8.3	8.3	8.4
8.6	8.6	8.6	8.6	8.6
7.8	7.8	7.8	7.8	7.8
5.0	5.0	5.0	5.0	5.0
5.4	5.4	5.4	5.4	5.4
4.7	4.7	4.6	4.7	4.7
6.6	6.6	6.6	6.6	6.6
6.6	6.6	6.6	6.6	6.6
4.7	4.7	4.6	4.7	4.7
14.1	14.0	14.0	14.0	14.0
N/A	N/A	N/A	11.4	11.4
5.2	5.2	5.2	5.2	5.2
5.2	5.2	5.2	5.2	5.3

0.0	0.0	0.0	0.0	0.0
0.0	0.0	0.0	0.0	0.0

401-A	402-A	403-A	433-B	434-B
RT(min)	RT(min)	RT(min)	RT(min)	RT(min)
3.0	3.0	3.0	3.0	3.0
3.8	3.8	3.8	3.8	3.8
3.9	3.9	3.8	3.9	3.9
5.7	5.7	5.7	5.6	5.6
9.3	9.3	9.3	9.3	9.2
4.3	4.2	4.2	4.2	4.3
8.5	8.5	8.5	8.5	8.5
3.8	3.8	3.8	3.8	3.8
7.6	7.6	7.6	7.6	7.6
4.1	4.2	4.1	4.2	4.2
3.8	3.8	3.8	3.8	3.8
2.2	2.3	2.2	2.3	2.3
9.3	9.3	9.3	9.3	9.2
8.8	8.9	8.8	8.8	8.8
2.2	2.2	2.2	2.2	2.2
4.2	4.3	4.2	4.3	4.3
7.1	7.1	7.1	7.0	7.0
2.2	2.2	2.2	2.3	2.3
2.3	2.3	2.3	2.3	2.3
8.6	8.6	8.6	8.5	8.5
6.8	6.8	6.8	6.7	6.7
N/A	N/A	N/A	8.6	8.5
N/A	N/A	N/A	8.6	8.5
10.5	10.6	10.6	10.5	10.5
8.0	8.0	8.0	8.0	8.0
9.7	9.7	9.7	9.7	9.7
9.2	9.2	9.2	9.2	9.2
3.8	3.8	3.8	3.8	3.8
9.2	9.2	9.2	9.2	9.2
2.2	2.2	2.2	2.2	2.2
3.8	3.8	3.8	3.8	3.8
2.3	2.3	2.3	2.3	2.3
11.4	11.4	11.4	11.2	11.2
11.6	11.5	11.5	11.5	11.5
11.4	11.4	11.4	11.2	11.2
5.6	5.6	5.6	5.6	5.6
9.9	9.9	9.9	9.9	9.9
3.8	3.8	3.8	3.8	3.8
3.8	3.8	3.8	3.8	3.8

4.7	4.7	4.7	4.7	4.7
9.9	9.9	9.9	9.9	9.9
5.3	5.3	5.2	5.3	5.3
10.6	10.6	10.6	10.7	10.7
6.5	6.5	6.5	6.4	6.5
8.2	8.2	8.2	8.2	8.2
7.6	7.6	7.6	7.6	7.6
N/A	N/A	N/A	4.0	4.0
8.0	8.0	8.0	8.0	8.0
6.9	6.9	6.9	6.9	6.9
2.1	2.1	2.1	2.1	2.1
N/A	N/A	N/A	9.8	9.8
N/A	N/A	N/A	8.8	8.8
N/A	N/A	N/A	10.9	10.9
9.6	N/A	N/A	N/A	9.5
2.0	2.0	2.0	2.0	2.0
N/A	N/A	N/A	10.0	10.0
N/A	N/A	N/A	9.6	9.6
N/A	N/A	N/A	8.6	8.6
10.3	10.4	10.4	10.4	10.3
8.6	8.6	8.6	8.6	8.6
8.3	8.3	8.3	8.3	8.3
5.3	5.3	5.3	5.3	5.3

435-B	Blank-3	QC-star-run-1	QC-star-run-2	QC-end-run-1
RT(min)	RT(min)	RT(min)	RT(min)	RT(min)
5.1	N/A	5.1	5.1	5.1
12.7	N/A	12.6	12.6	12.6
7.6	N/A	7.6	7.6	7.6
12.1	N/A	11.9	11.9	11.9
8.1	N/A	8.1	8.1	8.1
5.8	N/A	5.8	5.8	5.8
8.3	N/A	8.3	8.3	8.3
4.5	N/A	4.5	4.5	4.5
6.3	N/A	6.3	6.3	6.3
5.3	N/A	5.3	5.3	5.3
6.0	N/A	6.0	6.0	6.0
7.3	N/A	7.3	7.3	7.3
7.6	N/A	7.6	7.6	7.6
3.8	N/A	3.8	3.8	3.8
3.8	N/A	3.8	3.8	3.8
6.0	N/A	6.0	6.0	6.0
11.7	N/A	11.7	11.7	11.7
7.5	N/A	7.5	7.5	7.5
5.0	N/A	5.0	5.0	5.0
12.9	12.9	12.8	12.8	12.9
8.0	N/A	7.9	7.9	7.9
8.2	N/A	8.2	8.2	8.2
14.6	N/A	14.5	14.5	14.6
14.0	N/A	14.0	14.0	14.0
7.9	N/A	7.8	7.8	7.8
4.5	N/A	4.4	4.4	4.4
7.8	N/A	7.8	7.8	7.8
5.4	N/A	5.4	5.4	5.4
6.5	N/A	6.5	6.5	6.5
6.8	N/A	6.8	6.7	6.8
4.7	N/A	4.7	4.6	4.7
3.8	N/A	3.8	3.8	3.8
6.8	N/A	6.8	6.8	6.8
14.6	14.6	14.6	14.6	14.6
8.5	N/A	8.5	8.5	8.5
9.8	N/A	9.7	9.7	9.7
6.6	N/A	6.6	6.6	6.6
8.2	N/A	8.2	8.2	8.2
7.7	N/A	7.7	7.7	7.7
7.7	N/A	7.7	7.7	7.7
4.4	N/A	4.4	4.4	4.4
8.0	N/A	8.0	8.0	8.0

12.1	N/A	12.0	12.0	12.1
5.1	N/A	5.1	5.1	5.1
5.6	N/A	5.6	5.6	5.6
5.1	N/A	5.1	5.1	5.1
5.7	N/A	5.8	5.8	5.8
9.4	N/A	9.3	9.3	9.3
5.3	N/A	5.3	5.3	5.3
8.6	N/A	8.6	8.6	8.6
5.9	N/A	5.9	5.9	5.9
12.3	N/A	12.3	12.3	12.3
4.2	N/A	4.2	4.2	4.2
9.6	N/A	9.6	9.6	9.6
7.6	N/A	7.5	7.5	7.5
8.4	N/A	8.4	8.4	8.4
6.7	N/A	6.7	6.7	6.7
7.4	N/A	7.4	7.4	7.4
7.4	N/A	7.4	7.4	7.4
8.8	N/A	8.8	8.8	8.8
9.8	N/A	9.7	9.7	9.8
3.9	N/A	4.0	4.0	4.0
6.9	N/A	6.9	6.9	6.9
8.9	N/A	8.9	8.9	8.9
9.9	N/A	9.9	9.9	9.9
9.4	N/A	9.3	9.3	9.4
10.4	N/A	10.3	10.3	10.4
6.0	N/A	6.0	6.0	6.0
6.3	N/A	6.3	6.3	6.3
7.6	N/A	7.6	7.6	7.6
8.3	N/A	8.3	8.3	8.3
6.0	N/A	6.0	6.0	6.0
8.4	N/A	8.3	8.4	8.4
8.6	N/A	8.6	8.6	8.6
7.8	7.9	7.8	7.8	7.8
5.0	5.1	5.0	5.0	5.0
5.4	N/A	5.4	5.4	5.4
4.7	N/A	4.7	4.6	4.7
6.6	N/A	6.6	6.6	6.6
6.6	N/A	6.6	6.6	6.6
4.7	N/A	4.7	4.7	4.7
14.0	N/A	14.0	14.0	14.0
11.5	N/A	11.4	11.4	11.5
5.2	N/A	5.2	5.2	5.2
5.2	N/A	5.2	5.2	5.2

0.0	0.0	0.0	0.0	0.0
0.0	0.0	0.0	0.0	0.0
435-B	Blank-3	QC-star-run-1	QC-star-run-2	QC-end-run-1
RT(min)	RT(min)	RT(min)	RT(min)	RT(min)
3.1	N/A	3.0	3.1	3.1
3.8	N/A	3.8	3.8	3.8
3.9	N/A	3.9	3.9	3.9
5.7	N/A	5.7	5.7	5.7
9.2	N/A	9.3	9.3	9.3
4.3	N/A	4.3	4.3	4.3
8.5	N/A	8.5	8.5	8.5
3.8	N/A	3.8	3.8	3.8
7.6	N/A	7.6	7.6	7.6
4.2	N/A	4.2	4.2	4.2
3.8	N/A	3.8	3.8	3.8
2.3	N/A	2.3	2.3	2.3
9.2	N/A	9.3	9.3	9.3
8.8	N/A	8.8	8.8	8.8
2.2	N/A	2.2	2.2	2.2
4.3	N/A	4.3	4.3	4.3
7.0	N/A	7.1	7.1	7.1
2.3	N/A	2.2	2.2	2.2
2.3	N/A	2.3	2.3	2.3
8.5	N/A	8.6	8.6	8.5
6.7	N/A	6.8	6.8	6.8
8.5	N/A	8.6	8.6	8.6
8.5	N/A	8.6	8.6	8.5
10.5	N/A	10.5	10.5	10.5
8.0	N/A	8.0	8.0	8.0
9.7	N/A	9.7	9.7	9.7
9.2	N/A	9.2	9.2	9.2
3.8	N/A	3.8	3.8	3.8
9.2	N/A	9.2	9.2	9.2
2.2	N/A	2.2	2.2	2.2
3.8	N/A	3.8	3.8	3.8
2.3	N/A	2.3	2.3	2.3
11.1	11.4	11.3	11.3	11.2
11.5	11.6	11.5	11.5	11.5
11.2	11.4	11.3	11.3	11.2
5.6	N/A	5.6	5.6	5.6
9.9	N/A	9.9	9.9	9.9
3.8	N/A	3.8	3.8	3.8
3.8	N/A	3.8	3.8	3.8

4.7	N/A	4.7	4.7	4.7
9.9	N/A	9.9	9.9	9.9
5.3	N/A	5.3	5.3	5.3
10.7	N/A	10.6	10.6	10.6
6.5	N/A	6.5	6.5	6.5
8.2	N/A	8.2	8.2	8.2
7.6	N/A	7.6	7.6	7.6
4.1	N/A	4.0	4.0	4.0
8.0	N/A	8.0	8.0	8.0
6.9	N/A	6.9	6.9	6.9
2.1	N/A	2.1	2.1	2.1
9.8	N/A	9.8	9.8	9.8
8.8	N/A	8.8	8.8	8.8
10.9	N/A	10.9	10.9	10.9
9.7	N/A	N/A	9.4	N/A
2.0	N/A	2.0	2.0	2.0
10.0	N/A	10.0	10.1	10.0
9.6	N/A	9.6	9.6	9.5
8.6	N/A	8.6	8.6	8.6
10.3	N/A	10.4	10.4	10.3
8.6	N/A	8.6	8.6	8.6
8.3	N/A	8.3	8.3	8.3
5.3	N/A	5.3	5.3	5.3

QC-end-run-2	0	Sample Name
RT(min)	0	
5.1	0	Urea(61.1/44.2/RT(min):5.12)
12.7	0	ethanolamine(62.1/44.2/RT(min):12.53)
7.6	0	alanine(90.1/44.2/RT(min):7.79)
11.9	0	choline(104/60/RT(min):12.15
8.1	0	4-aminobutyrate(104.01/69/RT(min):8.27)
5.8	0	dimethylglycine(104.02/58/RT(min):5.92)
8.3	0	serine(106/60/RT(min):8.48)
4.5	0	creatinine(114/44.2/RT(min):4.53)
6.3	0	proline(116.1/70.1/RT(min):6.38)
5.3	0	betaine(118.02/58/RT(min):5.33)
6.0	0	valine(118.1/55.2/RT(min):6.08)
7.3	0	threonine(120/74/RT(min):7.42)
7.6	0	creatine(132.003/90/RT(min):7.78)
3.8	0	nicotinamide(123.1/80/RT(min):3.55)
3.8	0	thymine(127.1/110/RT(min):3.37)
6.0	0	DL-Pipecolic acid(130/84/RT(min):6.05)
11.7	0	N-Acetylputrescine(131.001/114/RT(min):11.70)
7.5	0	hydroxyproline(132.004/68.2/RT(min):7.66)
5.0	0	leucine(132.1/86/RT(min):5.07)
12.9	0	ornithine(133/70/RT(min):12.81)
8.0	0	asparagine(133.1/74/RT(min):8.18)
8.2	0	aspartate(134/74/RT(min):8.58)
14.6	0	methylnicotinamide(137.001/94/RT(min):14.07)
14.0	0	lysine(147/67/RT(min):14.08)
7.9	0	glutamine(147.1/84.1/RT(min):8.05)
4.4	0	O-acetyl-L-serine(148/106/RT(min):4.87)
7.8	0	glutamate(148.1/84.1/RT(min):8.21)
5.4	0	methionine(150.1/133/RT(min):5.46)
6.5	0	carnitine(162.1/103/RT(min):6.65)
6.8	0	Methionine sulfoxide(166/74/RT(min):7.00)
4.6	0	phenylalanine(166.1/103/RT(min):4.63)
3.8	0	pyridoxine(170/134/RT(min):3.65)
6.8	0	1-Methyl-Histidine(170.1/124/RT(min):7.52)
14.6	0	arginine(175.02/60/RT(min):14.59)
8.5	0	citrulline(176/159/RT(min):8.66)
9.7	0	N-carbamoyl-L-aspartate(177.05/74/RT(min):10.28)
6.6	0	tyrosine(182.1/77/RT(min):6.64)
8.2	0	Phosphorylcholine(184.001/125/RT(min):8.38)
7.8	0	N6-Acetyl-L-lysine(189.001/84.2/RT(min):7.97)
7.8	0	Acetyllysine(189.002/84/RT(min):7.97)
4.4	0	N-acetyl-glutamine(189.1/130/RT(min):4.96)
8.0	0	N-acetyl-glutamate (190.1/84.1/RT(min):7.89)

12.1	0	Ng_Ng-dimethyl-L-arginine(203/70/RT(min):12.15)
5.1	0	Acetylcarnitine DL(204/85/RT(min):5.17)
5.6	0	tryptophan(205/146/RT(min):5.74)
5.1	0	Kynurenine(209/146/RT(min):5.17)
5.8	0	N-acetyl-glucosamine(222/138/RT(min):5.74)
9.4	0	cystathionine(223/134/RT(min):9.64)
5.3	0	5-methoxytryptophan(235/176/RT(min):5.21)
8.6	0	Cystine(241.002/74/RT(min):9.08)
5.9	0	cytidine(244.1/112/RT(min):5.95)
12.3	0	thiamine(265/122/RT(min):12.39)
4.2	0	adenosine(268.15/136.1/RT(min):4.20)
9.6	0	L-arginino-succinate(291/70/RT(min):9.96)
7.6	0	glutathione (308.1/162/RT(min):7.8)
8.4	0	UMP(325/97/RT(min):8.91)
6.7	0	dAMP(332.1/136/RT(min):6.78)
7.4	0	thiamine-phosphate(345.2/122/RT(min):7.62)
7.4	0	AMP(348.15/136/RT(min):7.87)
8.8	0	IMP(349/137/RT(min):9.31)
9.8	0	GMP(364/152/RT(min):10.18)
4.0	0	riboflavin(377/243/RT(min):3.94)
6.9	0	S-adenosyl-L-homoCysteine_pos(385.1/136/RT(min):6.97
8.9	0	S-adenosyl-L-methionine(399.1/250/RT(min):9.1)
9.9	0	folate(442/295/RT(min):10.47)
9.3	0	7_8-dihydrofolate(444.2/178/RT(min):9.86)
10.4	0	glutathione disulfide_pos(613/231/RT(min):10.30)
6.0	0	FAD(786/348/RT(min):6.25)
6.3	0	13C-Proline1(122.00/75.00/RT(min):6.38)
7.6	0	13C-Alanine-1(93.94/47.00/RT(min):7.79)
8.3	0	13C-Serine-1(110.05/63.00/RT(min):8.48)
6.0	0	13C-Valine-1(123.93/77.10/RT(min):6.08)
8.4	0	13C-Glycine-1(78.91/32.00/RT(min):8.53)
8.6	0	13C-Cystine-1(248.89/155.90/RT(min):9.08)
7.8	0	13C-Glutamic acid-1(154.05/89.00/RT(min):8.22)
5.0	0	13C-Leucine-1(138.99/92.00/RT(min):5.07)
5.4	0	13C-Methionine-1(155.90/138.00/RT(min):5.46)
4.7	0	13C-Phenylalanine-1(176.01/128.90/RT(min):4.60)
6.6	0	13C-Tyrosine(192.01/174.00/RT(min):6.64)
6.6	0	13C tyrosine(192.00/83.00/RT(min):6.64)
4.7	0	13 C phenylalanine(176.00/111.00/RT(min):4.60)
14.0	0	13C lysine(155.00/90.10/RT(min):14.08)
11.5	0	Adenylosuccinate-1(464/252/RT(min): 11.58)
5.2	0	Isoleucine(132.1/86/RT(min):5.33)
5.2	0	13C-IsoLeucine-1(138.99/92.00/RT(min):5.33)

0.0	#	0.0
0.0	#	0.0

QC-end-run-2	0	Sample Name
RT(min)	0	
3.0	0	glycolate(75/45.2/RT(min):2.90)
3.8	0	pyruvate(87/43/RT(min):3.55)
3.9	0	lactate(89/43.2/RT(min):4.09)
5.7	0	glycerate(105/75/RT(min):5.81)
9.3	0	fumarate(115/71/RT(min):9.91)
4.2	0	Maleic acid(115.03/71.03/RT(min):4.45)
8.5	0	succinate(117/73/RT(min):9.10)
3.8	0	nicotinate(122/78/RT(min):3.60)
7.6	0	taurine(124/80/RT(min):7.72)
4.2	0	Pyroglutamic acid(128/82.1/RT(min):4.60)
3.8	0	N-Acetyl-L-alanine(130/88/RT(min):3.62)
2.3	0	Hydroxyisocaproic acid(131.006/85.1/RT(min):2.30)
9.3	0	malate(133/115/RT(min):9.78)
8.8	0	a-ketoglutarate(145/101/RT(min):9.42)
2.2	0	3-methylphenylacetic acid(149.002/105/RT(min):2.34*)
4.3	0	orotate(155/111/RT(min):4.71)
7.1	0	allantoin(157.05/114/RT(min):7.19)
2.2	0	phenylpyruvate(163/91/RT(min):2.38)
2.3	0	Phenyllactic acid(165.006/103.1/RT(min):2.30)
8.6	0	quinolinate(166/122/RT(min):9.13)
6.8	0	Uric acid(167.001/124/RT(min):6.71)
8.6	0	dihydroxy-acetone-phosphate(169/79/RT(min):9.03)
8.6	0	D-glyceraldehdye-3-phosphate(169.05/97/RT(min):9.03)
10.5	0	aconitate(173.05/85/RT(min):11.22)
8.0	0	allantoate(175/132/RT(min):8.35)
9.8	0	N-carbamoyl-L-aspartate_neg(175.03/132/RT(min):10.28
9.2	0	myo-inositol(179/161/RT(min):9.34)
3.8	0	hydroxyphenylpyruvate(179.05/107/RT(min):3.59)
9.2	0	homocysteic acid(182/80/RT(min):9.63)
2.2	0	4-Pyridoxic acid(182.003/138/RT(min):2.26)
3.8	0	Indoleacrylic acid(186/142.03/RT(min):3.60)
2.3	0	Kynurenic acid(188/144/RT(min):2.32)
11.3	0	citrate-isocitrate(191/111/RT(min):12)
11.5	0	isocitrate (191.02/117/RT(min):12.12)
11.3	0	citrate (191.05/87/RT(min):12.12)
5.6	0	Xanthurenic acid(204.001/160/RT(min):5.65)
9.9	0	D-glucarate(209/85/RT(min):10.20)
3.8	0	pantothenate(218/146/RT(min):3.59)
3.8	0	thymidine(241/125/RT(min):3.46)

4.7	0	uridine(243/200/RT(min):4.70)
9.9	0	glucose-6-phosphate(259.02/199/RT(min):10.38)
5.3	0	inosine(267/135/RT(min):5.41)
10.6	0	6-phospho-D-gluconate(275/97/RT(min):11.53)
6.5	0	xanthosine(283/151/RT(min):6.41)
8.2	0	N-acetyl-glucosamine-1-phosphate(300/79/RT(min):8.6)
7.6	0	glutathione_neg(306/143/RT(min):7.9)
4.0	0	cyclic-AMP(328/134.05/RT(min):4.45)
8.0	0	sucrose(341/179/RT(min):*8.09)
6.9	0	S-adenosyl-L-homocysteine_neg(383.1/134/RT(min):6.97
2.1	0	Deoxycholic acid(391.202/345.2/RT(min):2.11)
9.8	0	UDP_neg(403/159/RT(min):10.38)
8.8	0	ADP_neg(426.1/159/RT(min):9.38)
11.0	0	GDP_neg(442/159/RT(min):11.48)
N/A	0	dATP_neg(490/159/RT(min):9.95)
2.0	0	Taurodeoxycholic acid(498.2/124/RT(min):2.0)
10.0	0	ATP_neg(506.1/159/RT(min):10.72)
9.6	0	UDP-D-glucose(565/323/RT(min):10.05)
8.6	0	UDP-N-acetyl-glucosamine(606/385/RT(min):9.04)
10.3	0	glutathione disulfide_neg(611/306/RT(min):10.3*)
8.6	0	2-hydroxygluterate(147.1/128.7/RT(min):9.18)
8.3	0	13C-Serine(107.9/77.0/RT(min):8.49)
5.3	0	13C-Methionine(153.9/48.1/RT(min):5.46)

401-A	402-A	403-A
Peak Height	Peak Height	Peak Height
1.24E+07	1.37E+07	1.23E+07
4.40E+05	4.47E+05	6.70E+05
6.89E+06	7.19E+06	6.97E+06
1.58E+07	2.05E+07	2.99E+07
3.34E+04	1.86E+04	1.36E+05
9.18E+06	1.03E+07	9.24E+06
4.78E+06	5.79E+06	7.12E+06
1.37E+07	1.45E+07	1.51E+07
5.69E+07	5.83E+07	5.92E+07
6.82E+07	6.88E+07	6.85E+07
1.10E+06	1.26E+06	1.05E+06
1.32E+07	1.57E+07	1.63E+07
6.65E+07	6.88E+07	6.93E+07
7.65E+05	1.14E+06	1.78E+06
7.66E+04	8.88E+04	1.05E+05
6.89E+06	5.86E+06	5.70E+06
1.48E+04	1.68E+04	1.79E+04
1.60E+06	2.39E+06	2.71E+06
4.11E+07	4.03E+07	4.17E+07
1.85E+06	1.57E+06	1.39E+06
8.84E+05	8.31E+05	9.12E+05
3.48E+05	3.16E+05	9.34E+05
3.43E+05	5.84E+05	2.42E+05
1.15E+06	1.09E+06	1.25E+06
4.18E+07	4.27E+07	4.76E+07
3.90E+04	4.81E+04	4.67E+04
4.51E+06	4.77E+06	7.24E+06
8.10E+06	8.83E+06	8.47E+06
4.52E+07	5.29E+07	4.94E+07
3.17E+06	2.48E+06	3.36E+06
1.51E+07	1.51E+07	1.65E+07
8.73E+04	1.50E+05	4.42E+04
1.94E+06	2.61E+06	2.91E+06
9.57E+06	1.06E+07	1.17E+07
4.36E+07	4.42E+07	4.58E+07
3.45E+03	5.71E+03	4.56E+03
1.98E+06	2.29E+06	2.27E+06
3.92E+05	4.96E+05	5.19E+05
2.37E+05	3.01E+05	2.57E+05
2.05E+05	2.48E+05	2.18E+05
3.52E+05	4.50E+05	3.72E+05
3.32E+05	3.67E+05	4.81E+05

1.37E+06	1.72E+06	1.85E+06
2.48E+07	3.16E+07	2.60E+07
2.98E+07	2.86E+07	3.14E+07
5.38E+05	5.73E+05	5.86E+05
5.24E+04	5.08E+04	7.63E+04
1.00E+05	1.61E+05	2.56E+05
1.86E+04	2.59E+04	2.95E+04
4.86E+05	5.28E+05	6.06E+05
5.54E+06	7.56E+06	9.57E+06
6.70E+05	6.54E+05	8.09E+05
1.29E+05	2.59E+05	5.99E+04
2.88E+05	4.07E+05	6.89E+05
2.59E+05	3.23E+05	3.76E+05
N/A	N/A	N/A
N/A	N/A	N/A
4.12E+05	7.51E+05	5.13E+05
2.95E+04	2.26E+04	4.22E+04
N/A	N/A	N/A
1.43E+03	2.41E+03	9.70E+03
8.69E+04	1.07E+05	2.10E+05
3.28E+05	4.22E+05	3.01E+05
6.98E+05	9.25E+05	1.36E+06
6.39E+03	2.34E+04	9.42E+03
1.44E+04	1.75E+04	2.24E+04
2.02E+04	3.73E+04	2.07E+05
1.52E+04	8.57E+03	2.67E+04
6.50E+05	6.40E+05	5.91E+05
1.76E+04	1.35E+04	1.46E+04
3.74E+04	3.15E+04	2.52E+04
2.77E+05	2.76E+05	2.37E+05
1.82E+03	1.51E+03	1.98E+03
6.82E+04	7.33E+04	6.37E+04
6.88E+04	5.91E+04	5.55E+04
4.15E+05	3.87E+05	3.88E+05
2.36E+05	1.92E+05	1.77E+05
1.42E+06	1.35E+06	1.19E+06
7.66E+05	6.32E+05	6.08E+05
5.39E+04	4.19E+04	4.72E+04
2.16E+05	1.94E+05	1.57E+05
4.22E+04	4.05E+04	3.97E+04
N/A	N/A	N/A
1.67E+07	1.56E+07	1.31E+07
3.31E+05	2.42E+05	2.15E+05

0.0	0.0	0.0
0.0	0.0	0.0
401-A	402-A	403-A
Peak Height	Peak Height	Peak Height
1.46E+05	1.28E+05	1.40E+05
1.13E+06	1.40E+06	7.91E+05
4.65E+07	4.80E+07	4.41E+07
5.20E+04	7.30E+04	8.20E+04
3.44E+05	2.85E+05	2.68E+05
4.13E+04	3.62E+04	2.83E+04
2.56E+05	2.94E+05	3.05E+05
1.13E+05	6.97E+05	2.77E+05
2.55E+07	2.62E+07	3.66E+07
1.29E+04	1.87E+04	2.01E+04
1.19E+05	1.28E+05	1.61E+05
8.30E+04	1.15E+05	1.03E+05
2.98E+06	2.60E+06	2.53E+06
1.81E+06	1.59E+06	1.61E+06
8.92E+04	7.84E+04	1.01E+05
4.05E+05	4.69E+05	4.83E+05
2.06E+06	3.21E+06	3.26E+06
2.97E+04	2.87E+04	1.77E+04
1.72E+04	3.34E+04	3.25E+04
3.67E+05	3.63E+05	5.20E+05
3.17E+07	3.58E+07	3.77E+07
N/A	N/A	N/A
N/A	N/A	N/A
3.16E+05	4.04E+05	3.23E+05
3.14E+04	4.16E+04	2.87E+04
1.19E+05	1.65E+05	1.44E+05
7.99E+05	6.15E+05	1.39E+06
1.72E+05	2.60E+05	1.17E+05
4.42E+03	1.01E+04	1.55E+04
3.41E+05	5.95E+05	5.82E+05
1.52E+06	1.44E+06	1.60E+06
5.73E+04	6.84E+04	6.44E+04
1.08E+07	1.32E+07	1.21E+07
5.03E+04	9.42E+04	6.78E+04
3.48E+06	4.24E+06	4.00E+06
3.48E+05	3.83E+05	3.78E+05
1.89E+04	5.20E+04	3.77E+04
7.24E+05	9.90E+05	1.00E+06
1.99E+04	9.91E+03	3.41E+04

1.07E+04	1.34E+04	1.78E+04
1.11E+04	2.63E+04	2.94E+04
8.99E+03	1.75E+04	2.31E+04
1.42E+04	1.21E+04	1.34E+04
1.58E+06	1.81E+06	1.86E+06
6.68E+04	1.04E+05	1.96E+05
5.04E+04	6.79E+04	9.08E+04
N/A	N/A	N/A
5.19E+04	2.20E+05	8.54E+05
2.11E+05	2.65E+05	2.02E+05
2.11E+04	2.78E+04	2.15E+04
N/A	N/A	N/A
N/A	N/A	N/A
N/A	N/A	N/A
3.33E+02	N/A	N/A
5.61E+04	5.93E+04	1.14E+05
N/A	N/A	N/A
N/A	N/A	N/A
N/A	N/A	N/A
2.38E+04	4.54E+04	2.37E+05
2.60E+05	2.27E+05	3.09E+05
1.45E+04	1.09E+04	9.79E+03
2.16E+04	1.84E+04	1.73E+04

433-B	434-B	435-B
Peak Height	Peak Height	Peak Height
7.55E+06	7.95E+06	8.33E+06
1.85E+05	1.41E+05	1.49E+05
5.68E+06	5.56E+06	5.92E+06
1.25E+07	9.63E+06	1.07E+07
1.49E+04	1.55E+04	3.35E+04
7.71E+06	7.85E+06	6.18E+06
4.38E+06	4.39E+06	5.01E+06
1.27E+07	1.20E+07	1.23E+07
5.32E+07	5.33E+07	5.61E+07
6.79E+07	6.90E+07	6.77E+07
8.97E+05	8.91E+05	9.04E+05
1.77E+07	1.52E+07	1.64E+07
6.82E+07	6.84E+07	6.55E+07
3.98E+05	7.11E+05	8.55E+05
5.88E+04	6.21E+04	7.53E+04
3.84E+06	4.93E+06	4.65E+06
1.26E+04	1.65E+04	1.71E+04
3.14E+06	2.14E+06	2.68E+06
2.43E+07	2.63E+07	2.94E+07
1.29E+06	1.19E+06	1.32E+06
4.26E+05	3.36E+05	4.37E+05
2.07E+05	2.16E+05	2.59E+05
3.62E+05	4.99E+05	7.88E+05
8.45E+05	8.00E+05	1.03E+06
1.89E+07	2.02E+07	2.19E+07
2.95E+04	3.92E+04	3.53E+04
1.63E+06	1.60E+06	1.89E+06
6.43E+06	5.77E+06	6.85E+06
3.89E+07	4.14E+07	4.13E+07
2.43E+06	3.11E+06	3.46E+06
1.15E+07	1.13E+07	1.14E+07
8.75E+04	4.76E+04	1.01E+05
2.63E+06	2.09E+06	2.26E+06
9.82E+06	9.63E+06	1.11E+07
4.60E+07	5.06E+07	4.86E+07
7.10E+03	7.36E+03	7.93E+03
1.81E+06	1.58E+06	1.80E+06
2.05E+06	2.03E+06	2.29E+06
9.64E+04	1.17E+05	1.43E+05
7.74E+04	1.11E+05	1.22E+05
2.20E+05	3.33E+05	3.60E+05
2.06E+05	2.32E+05	1.83E+05

1.10E+06	1.25E+06	1.16E+06
1.81E+07	2.96E+07	2.33E+07
2.16E+07	2.46E+07	2.58E+07
5.79E+07	5.10E+07	5.19E+07
2.93E+04	2.19E+04	2.67E+04
1.30E+05	1.13E+05	1.25E+05
1.61E+04	2.59E+04	3.63E+04
2.83E+05	4.96E+05	4.63E+05
6.22E+06	4.81E+06	3.64E+06
3.22E+05	3.61E+05	4.57E+05
5.80E+04	5.34E+05	9.15E+05
5.31E+05	5.41E+05	6.42E+05
3.95E+05	8.86E+05	1.83E+06
6.17E+04	1.06E+05	7.72E+04
5.21E+04	7.01E+04	1.09E+05
4.60E+05	5.49E+05	6.02E+05
2.15E+07	4.19E+07	3.95E+07
5.09E+05	1.19E+06	3.41E+05
1.61E+06	2.09E+06	2.35E+06
1.11E+05	1.28E+05	1.03E+05
3.26E+05	5.28E+05	6.43E+05
1.98E+05	2.79E+05	2.80E+05
2.06E+04	1.03E+04	1.27E+04
1.96E+04	8.58E+03	7.47E+03
2.90E+05	2.67E+05	2.32E+05
1.74E+04	1.67E+04	1.44E+04
6.69E+05	7.52E+05	5.64E+05
1.18E+04	1.13E+04	1.47E+04
2.85E+04	2.94E+04	3.01E+04
2.54E+05	2.52E+05	1.89E+05
1.45E+03	1.40E+03	9.91E+02
6.90E+04	6.37E+04	5.91E+04
3.00E+04	2.88E+04	2.92E+04
2.26E+05	2.51E+05	2.45E+05
1.77E+05	1.58E+05	1.58E+05
1.02E+06	1.02E+06	9.24E+05
3.86E+05	4.24E+05	5.05E+05
2.52E+04	3.00E+04	2.86E+04
1.39E+05	1.55E+05	1.45E+05
4.10E+04	3.63E+04	4.27E+04
1.99E+04	2.60E+04	6.69E+04
1.15E+07	1.01E+07	9.84E+06
2.22E+05	1.95E+05	1.86E+05

0.0	0.0	0.0
0.0	0.0	0.0
433-B	434-B	435-B
Peak Height	Peak Height	Peak Height
1.37E+05	1.37E+05	1.28E+05
8.85E+05	9.74E+05	1.02E+06
4.82E+07	4.80E+07	4.85E+07
1.88E+04	2.23E+04	2.87E+04
1.02E+05	1.12E+05	2.31E+05
2.91E+04	2.64E+04	3.11E+04
2.82E+05	2.52E+05	3.19E+05
1.49E+05	1.44E+05	2.31E+05
1.50E+07	1.76E+07	1.51E+07
1.81E+04	2.02E+04	1.59E+04
9.35E+04	9.22E+04	1.00E+05
7.24E+04	7.29E+04	9.09E+04
9.86E+05	9.74E+05	2.03E+06
1.29E+06	1.05E+06	1.12E+06
5.25E+04	6.76E+04	5.79E+04
2.20E+05	3.25E+05	2.20E+05
9.98E+05	1.54E+06	2.23E+06
2.77E+04	2.04E+04	1.98E+04
9.95E+03	1.18E+04	2.07E+04
4.17E+05	3.90E+05	5.02E+05
2.47E+07	2.71E+07	2.34E+07
2.77E+05	4.15E+05	3.66E+05
6.14E+05	9.40E+05	6.68E+05
1.65E+06	1.82E+06	1.45E+06
1.54E+04	1.91E+04	2.58E+04
1.08E+05	1.18E+05	1.35E+05
2.88E+05	2.77E+05	3.07E+05
1.98E+05	8.96E+04	1.25E+05
5.38E+03	4.73E+03	5.56E+03
3.54E+05	2.84E+05	3.07E+05
9.03E+05	2.56E+06	1.15E+06
7.43E+04	2.04E+05	9.65E+04
1.62E+07	1.58E+07	1.64E+07
4.15E+05	5.19E+05	4.20E+05
4.98E+06	5.44E+06	5.39E+06
2.89E+05	3.16E+05	3.20E+05
1.79E+05	1.91E+05	2.55E+05
6.11E+05	5.62E+05	6.39E+05
4.30E+04	2.61E+04	2.24E+04

2.29E+03	2.43E+03	2.56E+03
6.43E+04	8.78E+04	9.51E+04
6.19E+03	1.73E+04	1.64E+04
1.23E+04	1.89E+04	1.33E+04
8.88E+04	1.20E+05	1.12E+05
6.28E+04	7.33E+04	7.69E+04
1.56E+05	2.54E+05	5.33E+05
3.17E+04	1.77E+04	1.70E+04
8.19E+04	5.23E+04	5.73E+05
2.26E+05	3.58E+05	3.41E+05
1.60E+04	1.01E+04	1.37E+04
9.45E+03	1.64E+04	1.62E+04
1.48E+05	2.01E+05	2.40E+05
1.83E+03	1.94E+03	4.16E+03
N/A	6.67E+02	1.00E+03
9.56E+04	2.69E+04	2.41E+04
1.19E+04	9.11E+03	1.05E+04
5.60E+04	1.95E+04	6.14E+04
4.06E+04	7.57E+04	4.24E+04
2.66E+05	1.55E+05	2.03E+05
1.19E+05	1.41E+05	1.83E+05
7.26E+03	9.85E+03	9.56E+03
1.38E+04	1.68E+04	1.49E+04

Blank-3	QC-star-run-1	QC-star-run-2
Peak Height	Peak Height	Peak Height
N/A	1.35E+07	1.36E+07
N/A	4.10E+05	3.70E+05
N/A	7.25E+06	7.16E+06
N/A	1.78E+07	1.62E+07
N/A	4.37E+04	4.72E+04
N/A	1.01E+07	9.11E+06
N/A	6.25E+06	5.78E+06
N/A	1.59E+07	1.43E+07
N/A	5.89E+07	5.93E+07
N/A	6.83E+07	6.75E+07
N/A	1.27E+06	1.13E+06
N/A	1.73E+07	1.78E+07
N/A	6.95E+07	6.92E+07
N/A	1.24E+06	1.14E+06
N/A	9.99E+04	1.09E+05
N/A	6.39E+06	5.84E+06
N/A	1.96E+04	2.06E+04
N/A	3.03E+06	2.80E+06
N/A	3.95E+07	3.88E+07
6.46E+03	1.66E+06	1.66E+06
N/A	6.28E+05	6.39E+05
N/A	4.65E+05	4.34E+05
N/A	6.10E+05	5.36E+05
N/A	1.16E+06	1.13E+06
N/A	2.84E+07	2.92E+07
N/A	4.88E+04	4.20E+04
N/A	3.11E+06	3.01E+06
N/A	8.62E+06	8.09E+06
N/A	4.75E+07	4.94E+07
N/A	3.11E+06	3.37E+06
N/A	1.53E+07	1.43E+07
N/A	9.32E+04	9.34E+04
N/A	2.56E+06	2.87E+06
2.35E+04	1.21E+07	1.19E+07
N/A	5.04E+07	4.97E+07
N/A	5.91E+03	8.74E+03
N/A	2.30E+06	2.07E+06
N/A	1.65E+06	1.59E+06
N/A	1.72E+05	1.82E+05
N/A	1.40E+05	1.50E+05
N/A	4.54E+05	4.27E+05
N/A	2.75E+05	2.87E+05

N/A	1.81E+06	1.44E+06
N/A	2.67E+07	2.87E+07
N/A	3.05E+07	2.81E+07
N/A	8.95E+06	8.43E+06
N/A	4.22E+04	5.37E+04
N/A	1.59E+05	1.62E+05
N/A	2.73E+04	2.67E+04
N/A	5.53E+05	4.98E+05
N/A	7.44E+06	6.79E+06
N/A	6.41E+05	5.35E+05
N/A	5.08E+05	4.66E+05
N/A	7.67E+05	7.81E+05
N/A	1.04E+06	1.00E+06
N/A	5.28E+04	6.21E+04
N/A	5.25E+04	5.68E+04
N/A	7.27E+05	7.16E+05
N/A	3.23E+07	2.93E+07
N/A	4.18E+05	4.51E+05
N/A	1.34E+06	1.29E+06
N/A	1.63E+05	1.47E+05
N/A	4.09E+05	4.54E+05
N/A	7.65E+05	7.15E+05
N/A	2.49E+04	1.62E+04
N/A	1.89E+04	1.99E+04
N/A	2.12E+05	2.10E+05
N/A	1.85E+04	1.53E+04
N/A	7.49E+05	7.07E+05
N/A	1.55E+04	1.42E+04
N/A	3.63E+04	2.99E+04
N/A	2.92E+05	2.82E+05
N/A	1.55E+03	1.67E+03
N/A	7.30E+04	7.56E+04
1.33E+02	4.66E+04	4.39E+04
1.33E+02	3.65E+05	4.14E+05
N/A	2.17E+05	1.95E+05
N/A	1.41E+06	1.31E+06
N/A	6.11E+05	4.97E+05
N/A	4.51E+04	3.63E+04
N/A	2.06E+05	2.07E+05
N/A	4.60E+04	4.32E+04
N/A	2.32E+04	2.78E+04
N/A	1.40E+07	1.44E+07
N/A	2.65E+05	2.59E+05

0.0	0.0	0.0
0.0	0.0	0.0
Blank-3	QC-star-run-1	QC-star-run-2
Peak Height	Peak Height	Peak Height
N/A	1.23E+05	1.31E+05
N/A	9.16E+05	9.90E+05
N/A	4.80E+07	4.74E+07
N/A	4.53E+04	4.34E+04
N/A	1.83E+05	2.02E+05
N/A	6.14E+04	5.98E+04
N/A	4.59E+05	4.65E+05
N/A	3.04E+05	2.70E+05
N/A	2.29E+07	2.25E+07
N/A	1.90E+04	1.82E+04
N/A	1.11E+05	1.10E+05
N/A	9.39E+04	8.21E+04
N/A	1.72E+06	1.91E+06
N/A	1.54E+06	1.61E+06
N/A	6.72E+04	7.62E+04
N/A	3.75E+05	3.65E+05
N/A	2.34E+06	2.51E+06
N/A	2.22E+04	1.99E+04
N/A	1.61E+04	1.94E+04
N/A	4.37E+05	4.14E+05
N/A	3.00E+07	2.95E+07
N/A	2.21E+05	1.79E+05
N/A	4.80E+05	4.09E+05
N/A	9.22E+05	1.01E+06
N/A	1.72E+04	2.11E+04
N/A	1.39E+05	1.63E+05
N/A	4.50E+05	4.52E+05
N/A	1.68E+05	1.67E+05
N/A	4.80E+03	6.57E+03
N/A	4.13E+05	4.41E+05
N/A	1.69E+06	1.69E+06
N/A	7.54E+04	7.64E+04
1.31E+06	1.20E+07	1.38E+07
3.36E+03	2.49E+05	2.60E+05
4.30E+05	4.04E+06	4.37E+06
N/A	3.73E+05	3.53E+05
N/A	1.34E+05	1.32E+05
N/A	6.99E+05	7.11E+05
N/A	1.16E+04	7.54E+03

N/A	7.77E+03	7.79E+03
N/A	6.49E+04	5.71E+04
N/A	1.71E+04	1.56E+04
N/A	1.62E+04	1.14E+04
N/A	8.84E+05	8.74E+05
N/A	1.23E+05	9.70E+04
N/A	2.12E+05	2.04E+05
N/A	1.35E+04	1.22E+04
N/A	2.50E+05	2.59E+05
N/A	2.71E+05	2.54E+05
N/A	1.95E+04	1.84E+04
N/A	7.12E+03	7.50E+03
N/A	1.10E+05	9.92E+04
N/A	1.03E+03	1.32E+03
N/A	N/A	3.33E+02
N/A	7.33E+04	6.44E+04
N/A	5.83E+03	4.92E+03
N/A	2.20E+04	2.18E+04
N/A	1.96E+04	2.08E+04
N/A	1.76E+05	1.53E+05
N/A	2.55E+05	2.64E+05
N/A	9.26E+03	9.16E+03
N/A	1.67E+04	1.69E+04

QC-end-run-1	QC-end-run-2	0
Peak Height	Peak Height	0
1.18E+07	1.13E+07	0
3.30E+05	3.52E+05	0
6.18E+06	6.15E+06	0
1.57E+07	1.58E+07	0
4.26E+04	4.57E+04	0
8.85E+06	9.23E+06	0
5.40E+06	5.34E+06	0
1.32E+07	1.46E+07	0
5.67E+07	5.70E+07	0
6.71E+07	6.80E+07	0
1.12E+06	1.17E+06	0
1.74E+07	1.64E+07	0
6.77E+07	6.77E+07	0
1.14E+06	1.14E+06	0
8.42E+04	9.80E+04	0
5.94E+06	6.03E+06	0
1.83E+04	1.99E+04	0
2.55E+06	2.58E+06	0
3.97E+07	3.86E+07	0
1.44E+06	1.46E+06	0
5.04E+05	5.55E+05	0
4.05E+05	3.94E+05	0
5.26E+05	5.09E+05	0
1.03E+06	1.12E+06	0
2.70E+07	2.78E+07	0
4.33E+04	4.46E+04	0
2.92E+06	2.97E+06	0
7.75E+06	7.83E+06	0
4.99E+07	4.95E+07	0
2.93E+06	3.14E+06	0
1.39E+07	1.37E+07	0
8.47E+04	8.58E+04	0
2.65E+06	2.34E+06	0
1.14E+07	1.06E+07	0
4.85E+07	4.93E+07	0
6.32E+03	7.41E+03	0
2.13E+06	2.18E+06	0
1.36E+06	1.44E+06	0
1.76E+05	1.74E+05	0
1.41E+05	1.53E+05	0
3.75E+05	4.18E+05	0
2.40E+05	2.52E+05	0

1.47E+06	1.47E+06	0
2.87E+07	2.91E+07	0
2.91E+07	2.87E+07	0
9.09E+06	8.44E+06	0
3.51E+04	4.20E+04	0
1.61E+05	1.50E+05	0
2.33E+04	3.30E+04	0
5.38E+05	5.13E+05	0
6.77E+06	6.96E+06	0
6.03E+05	5.85E+05	0
4.76E+05	4.65E+05	0
6.09E+05	6.93E+05	0
8.70E+05	8.16E+05	0
5.87E+04	5.30E+04	0
5.59E+04	5.43E+04	0
6.42E+05	5.90E+05	0
2.88E+07	2.85E+07	0
4.43E+05	3.96E+05	0
1.34E+06	1.26E+06	0
1.54E+05	1.46E+05	0
4.32E+05	3.97E+05	0
6.74E+05	6.90E+05	0
2.35E+04	1.64E+04	0
1.76E+04	1.58E+04	0
2.06E+05	1.78E+05	0
2.78E+04	2.02E+04	0
6.41E+05	6.94E+05	0
1.23E+04	1.43E+04	0
3.00E+04	3.30E+04	0
2.60E+05	2.65E+05	0
1.59E+03	9.23E+02	0
7.17E+04	6.27E+04	0
3.89E+04	4.26E+04	0
3.71E+05	3.54E+05	0
1.93E+05	1.99E+05	0
1.31E+06	1.37E+06	0
5.09E+05	5.30E+05	0
3.92E+04	3.84E+04	0
1.93E+05	1.93E+05	0
4.91E+04	4.19E+04	0
2.32E+04	2.52E+04	0
1.29E+07	1.41E+07	0
2.37E+05	2.45E+05	0

0.0	0.0	#
0.0	0.0	#
QC-end-run-1	QC-end-run-2	0
Peak Height	Peak Height	0
1.39E+05	1.37E+05	0
9.62E+05	9.70E+05	0
4.94E+07	4.91E+07	0
4.71E+04	4.09E+04	0
2.08E+05	2.01E+05	0
6.08E+04	5.70E+04	0
4.60E+05	4.64E+05	0
3.48E+05	2.81E+05	0
2.20E+07	2.43E+07	0
2.04E+04	1.80E+04	0
1.28E+05	1.26E+05	0
9.48E+04	8.76E+04	0
1.84E+06	1.89E+06	0
1.36E+06	1.60E+06	0
7.74E+04	7.11E+04	0
4.06E+05	3.85E+05	0
2.38E+06	2.45E+06	0
2.44E+04	2.50E+04	0
2.32E+04	2.09E+04	0
4.34E+05	4.54E+05	0
2.90E+07	2.96E+07	0
1.88E+05	2.37E+05	0
3.80E+05	4.48E+05	0
1.13E+06	1.07E+06	0
2.00E+04	1.68E+04	0
1.35E+05	1.35E+05	0
4.20E+05	4.71E+05	0
1.77E+05	1.70E+05	0
6.55E+03	7.17E+03	0
4.10E+05	4.61E+05	0
1.80E+06	1.77E+06	0
8.07E+04	8.02E+04	0
1.35E+07	1.29E+07	0
2.79E+05	2.90E+05	0
4.65E+06	4.51E+06	0
3.66E+05	3.85E+05	0
1.28E+05	1.38E+05	0
7.75E+05	7.62E+05	0
1.33E+04	2.46E+04	0

6.67E+03	8.28E+03	0
5.82E+04	5.13E+04	0
1.67E+04	1.76E+04	0
1.11E+04	1.32E+04	0
8.20E+05	9.55E+05	0
1.14E+05	1.14E+05	0
2.28E+05	1.88E+05	0
1.49E+04	1.53E+04	0
2.92E+05	2.91E+05	0
2.75E+05	2.61E+05	0
2.09E+04	2.30E+04	0
6.26E+03	6.84E+03	0
1.07E+05	1.08E+05	0
9.76E+02	8.83E+02	0
N/A	N/A	0
6.28E+04	6.24E+04	0
5.27E+03	4.90E+03	0
2.30E+04	2.27E+04	0
2.15E+04	2.33E+04	0
1.70E+05	1.71E+05	0
2.58E+05	2.77E+05	0
7.77E+03	9.28E+03	0
2.03E+04	1.88E+04	0

Sample Name	401-A
	Peak Area
Urea(61.1/44.2/RT(min):5.12)	1.52E+08
ethanolamine(62.1/44.2/RT(min):12.53)	8.08E+06
alanine(90.1/44.2/RT(min):7.79)	8.83E+07
choline(104/60/RT(min):12.15	4.16E+08
4-aminobutyrate(104.01/69/RT(min):8.27)	4.31E+05
dimethylglycine(104.02/58/RT(min):5.92)	1.19E+08
serine(106/60/RT(min):8.48)	5.13E+07
creatinine(114/44.2/RT(min):4.53)	1.61E+08
proline(116.1/70.1/RT(min):6.38)	1.08E+09
betaine(118.02/58/RT(min):5.33)	1.15E+09
valine(118.1/55.2/RT(min):6.08)	1.69E+07
threonine(120/74/RT(min):7.42)	1.41E+08
creatine(132.003/90/RT(min):7.78)	1.06E+09
nicotinamide(123.1/80/RT(min):3.55)	5.84E+06
thymine(127.1/110/RT(min):3.37)	2.54E+05
DL-Pipecolic acid(130/84/RT(min):6.05)	5.51E+07
N-Acetylputrescine(131.001/114/RT(min):11.70)	2.56E+05
hydroxyproline(132.004/68.2/RT(min):7.66)	1.51E+07
leucine(132.1/86/RT(min):5.07)	5.69E+08
ornithine(133/70/RT(min):12.81)	4.22E+07
asparagine(133.1/74/RT(min):8.18)	9.85E+06
aspartate(134/74/RT(min):8.58)	4.49E+06
methylnicotinamide(137.001/94/RT(min):14.07)	7.25E+06
lysine(147/67/RT(min):14.08)	1.91E+07
glutamine(147.1/84.1/RT(min):8.05)	5.44E+08
O-acetyl-L-serine(148/106/RT(min):4.87)	8.80E+05
glutamate(148.1/84.1/RT(min):8.21)	6.61E+07
methionine(150.1/133/RT(min):5.46)	1.17E+08
carnitine(162.1/103/RT(min):6.65)	6.46E+08
Methionine sulfoxide(166/74/RT(min):7.00)	3.53E+07
phenylalanine(166.1/103/RT(min):4.63)	1.98E+08
pyridoxine(170/134/RT(min):3.65)	6.95E+05
1-Methyl-Histidine(170.1/124/RT(min):7.52)	8.37E+07
arginine(175.02/60/RT(min):14.59)	2.13E+08
citrulline(176/159/RT(min):8.66)	5.58E+08
N-carbamoyl-L-aspartate(177.05/74/RT(min):10.28)	3.89E+04
tyrosine(182.1/77/RT(min):6.64)	2.71E+07
Phosphorylcholine(184.001/125/RT(min):8.38)	1.11E+07
N6-Acetyl-L-lysine(189.001/84.2/RT(min):7.97)	2.69E+06
Acetyllysine(189.002/84/RT(min):7.97)	2.09E+06
N-acetyl-glutamine(189.1/130/RT(min):4.96)	7.62E+06
N-acetyl-glutamate (190.1/84.1/RT(min):7.89)	3.71E+06

Ng_Ng-dimethyl-L-arginine(203/70/RT(min):12.15)	2.34E+07
Acetylcarnitine DL(204/85/RT(min):5.17)	4.00E+08
tryptophan(205/146/RT(min):5.74)	4.19E+08
Kynurenine(209/146/RT(min):5.17)	5.14E+06
N-acetyl-glucosamine(222/138/RT(min):5.74)	1.53E+06
cystathionine(223/134/RT(min):9.64)	1.35E+06
5-methoxytryptophan(235/176/RT(min):5.21)	2.15E+05
Cystine(241.002/74/RT(min):9.08)	5.71E+06
cytidine(244.1/112/RT(min):5.95)	5.97E+07
thiamine(265/122/RT(min):12.39)	1.93E+07
adenosine(268.15/136.1/RT(min):4.20)	1.30E+06
L-arginino-succinate(291/70/RT(min):9.96)	3.03E+06
glutathione (308.1/162/RT(min):7.8)	2.89E+06
UMP(325/97/RT(min):8.91)	N/A
dAMP(332.1/136/RT(min):6.78)	N/A
thiamine-phosphate(345.2/122/RT(min):7.62)	5.91E+06
AMP(348.15/136/RT(min):7.87)	3.63E+05
IMP(349/137/RT(min):9.31)	N/A
GMP(364/152/RT(min):10.18)	1.83E+04
riboflavin(377/243/RT(min):3.94)	8.60E+05
S-adenosyl-L-homoCysteine_pos(385.1/136/RT(min):6.97)	3.39E+06
S-adenosyl-L-methionine(399.1/250/RT(min):9.1)	1.15E+07
folate(442/295/RT(min):10.47)	7.61E+04
7_8-dihydrofolate(444.2/178/RT(min):9.86)	1.40E+05
glutathione disulfide_pos(613/231/RT(min):10.30)	2.15E+05
FAD(786/348/RT(min):6.25)	1.63E+05
13C-Proline1(122.00/75.00/RT(min):6.38)	1.14E+07
13C-Alanine-1(93.94/47.00/RT(min):7.79)	1.91E+05
13C-Serine-1(110.05/63.00/RT(min):8.48)	3.74E+05
13C-Valine-1(123.93/77.10/RT(min):6.08)	4.07E+06
13C-Glycine-1(78.91/32.00/RT(min):8.53)	1.58E+04
13C-Cystine-1(248.89/155.90/RT(min):9.08)	7.95E+05
13C-Glutamic acid-1(154.05/89.00/RT(min):8.22)	9.70E+05
13C-Leucine-1(138.99/92.00/RT(min):5.07)	5.56E+06
13C-Methionine-1(155.90/138.00/RT(min):5.46)	3.03E+06
13C-Phenylalanine-1(176.01/128.90/RT(min):4.60)	1.96E+07
13C-Tyrosine(192.01/174.00/RT(min):6.64)	9.88E+06
13C tyrosine(192.00/83.00/RT(min):6.64)	7.30E+05
13 C phenylalanine(176.00/111.00/RT(min):4.60)	2.89E+06
13C lysine(155.00/90.10/RT(min):14.08)	7.13E+05
Adenylosuccinate-1(464/252/RT(min): 11.58)	N/A
Isoleucine(132.1/86/RT(min):5.33)	1.84E+08
13C-IsoLeucine-1(138.99/92.00/RT(min):5.33)	3.82E+06

	0.0	0.0
	0.0	0.0

Sample Name	401-A
	Peak Area
glycolate(75/45.2/RT(min):2.90)	5.16E+06
pyruvate(87/43/RT(min):3.55)	8.90E+06
lactate(89/43.2/RT(min):4.09)	4.21E+08
glycerate(105/75/RT(min):5.81)	1.47E+06
fumarate(115/71/RT(min):9.91)	5.02E+06
Maleic acid(115.03/71.03/RT(min):4.45)	3.25E+05
succinate(117/73/RT(min):9.10)	4.30E+06
nicotinate(122/78/RT(min):3.60)	6.75E+05
taurine(124/80/RT(min):7.72)	2.85E+08
Pyroglutamic acid(128/82.1/RT(min):4.60)	2.80E+05
N-Acetyl-L-alanine(130/88/RT(min):3.62)	7.19E+05
Hydroxyisocaproic acid(131.006/85.1/RT(min):2.30)	5.03E+05
malate(133/115/RT(min):9.78)	4.67E+07
a-ketoglutarate(145/101/RT(min):9.42)	2.14E+07
3-methylphenylacetic acid(149.002/105/RT(min):2.34*)	6.65E+05
orotate(155/111/RT(min):4.71)	7.72E+06
allantoin(157.05/114/RT(min):7.19)	1.89E+07
phenylpyruvate(163/91/RT(min):2.38)	2.71E+05
Phenyllactic acid(165.006/103.1/RT(min):2.30)	1.40E+05
quinolinate(166/122/RT(min):9.13)	4.18E+06
Uric acid(167.001/124/RT(min):6.71)	4.34E+08
dihydroxy-acetone-phosphate(169/79/RT(min):9.03)	N/A
D-glyceraldehdye-3-phosphate(169.05/97/RT(min):9.03)	N/A
aconitate(173.05/85/RT(min):11.22)	4.50E+06
allantoate(175/132/RT(min):8.35)	3.36E+05
N-carbamoyl-L-aspartate_neg(175.03/132/RT(min):10.28)	1.18E+06
myo-inositol(179/161/RT(min):9.34)	8.47E+06
hydroxyphenylpyruvate(179.05/107/RT(min):3.59)	1.07E+06
homocysteic acid(182/80/RT(min):9.63)	5.61E+04
4-Pyridoxic acid(182.003/138/RT(min):2.26)	3.35E+06
Indoleacrylic acid(186/142.03/RT(min):3.60)	9.25E+06
Kynurenic acid(188/144/RT(min):2.32)	7.22E+05
citrate-isocitrate(191/111/RT(min):12)	4.13E+08
isocitrate (191.02/117/RT(min):12.12)	9.35E+05
citrate (191.05/87/RT(min):12.12)	1.40E+08
Xanthurenic acid(204.001/160/RT(min):5.65)	4.27E+06
D-glucarate(209/85/RT(min):10.20)	2.82E+05
pantothenate(218/146/RT(min):3.59)	4.35E+06
thymidine(241/125/RT(min):3.46)	1.20E+05

uridine(243/200/RT(min):4.70)	1.03E+05
glucose-6-phosphate(259.02/199/RT(min):10.38)	2.03E+05
inosine(267/135/RT(min):5.41)	1.19E+05
6-phospho-D-gluconate(275/97/RT(min):11.53)	2.29E+05
xanthosine(283/151/RT(min):6.41)	1.85E+07
N-acetyl-glucosamine-1-phosphate(300/79/RT(min):8.6)	7.99E+05
glutathione_neg(306/143/RT(min):7.9)	6.17E+05
cyclic-AMP(328/134.05/RT(min):4.45)	N/A
sucrose(341/179/RT(min):*8.09)	5.55E+05
S-adenosyl-L-homocysteine_neg(383.1/134/RT(min):6.97)	1.99E+06
Deoxycholic acid(391.202/345.2/RT(min):2.11)	1.87E+05
UDP_neg(403/159/RT(min):10.38)	N/A
ADP_neg(426.1/159/RT(min):9.38)	N/A
GDP_neg(442/159/RT(min):11.48)	N/A
dATP_neg(490/159/RT(min):9.95)	6.47E+02
Taurodeoxycholic acid(498.2/124/RT(min):2.0)	5.58E+05
ATP_neg(506.1/159/RT(min):10.72)	N/A
UDP-D-glucose(565/323/RT(min):10.05)	N/A
UDP-N-acetyl-glucosamine(606/385/RT(min):9.04)	N/A
glutathione disulfide_neg(611/306/RT(min):10.3*)	2.42E+05
2-hydroxygluterate(147.1/128.7/RT(min):9.18)	3.09E+06
13C-Serine(107.9/77.0/RT(min):8.49)	1.30E+05
13C-Methionine(153.9/48.1/RT(min):5.46)	2.60E+05

402-A	403-A	433-B	434-B
Peak Area	Peak Area	Peak Area	Peak Area
1.60E+08	1.51E+08	9.85E+07	1.03E+08
8.36E+06	1.34E+07	3.40E+06	2.62E+06
9.79E+07	9.54E+07	7.46E+07	7.34E+07
4.41E+08	6.50E+08	2.32E+08	1.68E+08
2.48E+05	1.62E+06	1.70E+05	1.97E+05
1.36E+08	1.23E+08	1.05E+08	1.07E+08
6.11E+07	7.60E+07	4.60E+07	4.63E+07
1.67E+08	1.77E+08	1.56E+08	1.51E+08
1.12E+09	1.19E+09	1.03E+09	1.00E+09
1.23E+09	1.32E+09	1.16E+09	1.35E+09
1.95E+07	1.63E+07	1.47E+07	1.41E+07
1.60E+08	1.64E+08	2.00E+08	1.67E+08
1.25E+09	1.24E+09	1.08E+09	1.15E+09
8.68E+06	1.29E+07	2.64E+06	4.85E+06
3.27E+05	3.54E+05	1.86E+05	2.20E+05
5.76E+07	4.78E+07	3.80E+07	5.37E+07
3.20E+05	3.01E+05	2.14E+05	2.54E+05
2.26E+07	2.51E+07	3.11E+07	2.11E+07
5.72E+08	5.86E+08	3.57E+08	3.47E+08
3.55E+07	2.94E+07	2.80E+07	2.45E+07
9.02E+06	1.00E+07	5.21E+06	4.06E+06
3.88E+06	1.11E+07	2.46E+06	2.53E+06
1.21E+07	4.79E+06	6.41E+06	9.47E+06
1.81E+07	2.09E+07	1.47E+07	1.39E+07
5.52E+08	6.33E+08	2.68E+08	2.94E+08
1.02E+06	1.21E+06	6.85E+05	7.84E+05
6.35E+07	9.97E+07	2.65E+07	2.48E+07
1.19E+08	1.19E+08	8.84E+07	8.11E+07
8.46E+08	7.48E+08	5.64E+08	5.71E+08
3.40E+07	4.42E+07	2.57E+07	3.43E+07
2.10E+08	2.24E+08	1.57E+08	1.54E+08
1.19E+06	3.65E+05	6.35E+05	3.58E+05
1.00E+08	1.03E+08	1.23E+08	1.03E+08
2.33E+08	2.78E+08	2.08E+08	2.01E+08
5.85E+08	5.95E+08	6.46E+08	6.97E+08
4.87E+04	5.40E+04	8.44E+04	8.13E+04
3.22E+07	3.21E+07	2.51E+07	2.34E+07
1.32E+07	1.45E+07	5.47E+07	5.47E+07
3.07E+06	2.63E+06	1.03E+06	1.27E+06
2.44E+06	2.20E+06	9.11E+05	1.08E+06
9.59E+06	8.74E+06	3.89E+06	6.18E+06
4.15E+06	5.37E+06	2.50E+06	2.97E+06

2.74E+07	3.15E+07	1.98E+07	2.07E+07
5.24E+08	4.37E+08	2.96E+08	4.88E+08
4.26E+08	4.38E+08	3.04E+08	3.36E+08
5.56E+06	5.78E+06	7.21E+08	6.68E+08
1.42E+06	2.14E+06	8.48E+05	5.73E+05
2.35E+06	3.76E+06	1.79E+06	1.53E+06
2.91E+05	3.42E+05	2.31E+05	3.36E+05
6.02E+06	6.80E+06	3.53E+06	5.89E+06
8.39E+07	1.07E+08	7.08E+07	5.66E+07
2.18E+07	2.72E+07	8.51E+06	9.18E+06
2.56E+06	6.03E+05	6.36E+05	5.85E+06
4.38E+06	7.06E+06	5.64E+06	5.93E+06
3.81E+06	4.50E+06	4.70E+06	1.08E+07
N/A	N/A	7.02E+05	1.19E+06
N/A	N/A	5.54E+05	7.04E+05
9.77E+06	6.61E+06	6.17E+06	7.23E+06
3.39E+05	5.23E+05	2.59E+08	5.13E+08
N/A	N/A	6.37E+06	1.47E+07
2.51E+04	1.06E+05	1.79E+07	2.38E+07
1.15E+06	2.05E+06	1.15E+06	1.34E+06
4.18E+06	3.27E+06	3.49E+06	5.48E+06
1.37E+07	2.43E+07	1.95E+06	2.77E+06
2.78E+05	7.49E+04	2.56E+05	1.19E+05
1.92E+05	2.29E+05	2.07E+05	1.16E+05
4.18E+05	2.27E+06	2.95E+06	2.74E+06
1.16E+05	3.27E+05	1.47E+05	1.67E+05
1.08E+07	1.00E+07	1.18E+07	1.23E+07
1.56E+05	1.65E+05	1.47E+05	1.42E+05
3.36E+05	2.86E+05	3.02E+05	3.05E+05
4.02E+06	3.61E+06	4.01E+06	3.87E+06
1.58E+04	2.10E+04	1.23E+04	1.58E+04
8.65E+05	7.12E+05	8.01E+05	6.72E+05
8.83E+05	7.46E+05	5.82E+05	4.41E+05
4.84E+06	5.04E+06	3.46E+06	3.69E+06
2.52E+06	2.26E+06	2.24E+06	2.07E+06
1.79E+07	1.60E+07	1.40E+07	1.46E+07
8.93E+06	8.27E+06	6.06E+06	6.24E+06
6.46E+05	6.55E+05	3.68E+05	4.21E+05
2.52E+06	2.17E+06	1.97E+06	1.99E+06
7.35E+05	6.78E+05	6.71E+05	5.89E+05
N/A	N/A	1.97E+05	2.49E+05
1.75E+08	1.53E+08	1.34E+08	1.24E+08
3.08E+06	2.81E+06	2.78E+06	2.76E+06

0.0	0.0	0.0	0.0
0.0	0.0	0.0	0.0
402-A	403-A	433-B	434-B
Peak Area	Peak Area	Peak Area	Peak Area
4.81E+06	5.14E+06	4.70E+06	4.84E+06
9.82E+06	5.48E+06	7.59E+06	7.95E+06
4.98E+08	3.74E+08	7.15E+08	7.13E+08
2.19E+06	2.37E+06	4.65E+05	6.10E+05
4.09E+06	3.91E+06	1.32E+06	1.26E+06
2.60E+05	2.21E+05	1.85E+05	1.93E+05
4.77E+06	5.19E+06	4.16E+06	3.55E+06
3.98E+06	1.61E+06	8.69E+05	8.48E+05
2.90E+08	3.99E+08	1.53E+08	1.72E+08
4.22E+05	4.61E+05	2.76E+05	3.62E+05
7.84E+05	9.50E+05	5.95E+05	5.49E+05
7.04E+05	6.36E+05	4.93E+05	4.82E+05
3.71E+07	3.60E+07	1.18E+07	1.22E+07
1.86E+07	1.90E+07	1.37E+07	1.13E+07
5.17E+05	6.26E+05	3.42E+05	4.45E+05
8.28E+06	9.71E+06	3.26E+06	5.23E+06
3.27E+07	3.16E+07	1.18E+07	1.52E+07
2.47E+05	1.52E+05	2.51E+05	1.66E+05
2.46E+05	2.05E+05	7.92E+04	8.48E+04
4.35E+06	6.16E+06	4.69E+06	4.21E+06
5.10E+08	6.53E+08	3.34E+08	3.75E+08
N/A	N/A	3.33E+06	4.71E+06
N/A	N/A	5.09E+06	7.43E+06
6.78E+06	5.83E+06	2.27E+07	2.71E+07
4.59E+05	2.86E+05	1.41E+05	1.86E+05
1.78E+06	1.68E+06	1.21E+06	1.30E+06
6.91E+06	1.50E+07	3.17E+06	3.01E+06
1.65E+06	7.45E+05	1.20E+06	5.71E+05
1.02E+05	1.42E+05	5.64E+04	5.88E+04
5.57E+06	5.84E+06	3.38E+06	2.44E+06
8.94E+06	9.73E+06	5.99E+06	1.59E+07
8.58E+05	8.75E+05	9.72E+05	2.21E+06
6.17E+08	5.33E+08	6.57E+08	6.87E+08
1.58E+06	1.21E+06	5.34E+06	6.96E+06
1.93E+08	1.75E+08	2.28E+08	2.24E+08
4.73E+06	4.81E+06	3.71E+06	3.99E+06
7.88E+05	5.57E+05	2.21E+06	2.30E+06
6.07E+06	6.01E+06	3.65E+06	3.37E+06
7.93E+04	1.97E+05	2.39E+05	1.54E+05

1.26E+05	1.79E+05	1.75E+04	2.27E+04
3.22E+05	4.55E+05	7.65E+05	1.04E+06
2.32E+05	3.15E+05	8.08E+04	2.09E+05
1.97E+05	1.97E+05	2.78E+05	3.48E+05
2.19E+07	2.28E+07	1.19E+06	1.61E+06
1.23E+06	2.26E+06	7.79E+05	8.50E+05
9.33E+05	1.17E+06	1.74E+06	2.95E+06
N/A	N/A	3.31E+05	2.32E+05
2.35E+06	8.98E+06	8.98E+05	5.88E+05
2.30E+06	1.77E+06	2.03E+06	3.34E+06
2.37E+05	1.87E+05	1.28E+05	1.03E+05
N/A	N/A	1.11E+05	2.02E+05
N/A	N/A	1.87E+06	2.68E+06
N/A	N/A	2.33E+04	2.11E+04
N/A	N/A	N/A	9.72E+02
5.75E+05	1.14E+06	9.32E+05	2.86E+05
N/A	N/A	2.27E+05	1.84E+05
N/A	N/A	6.01E+05	2.26E+05
N/A	N/A	3.83E+05	6.98E+05
4.46E+05	2.35E+06	2.49E+06	1.53E+06
2.44E+06	3.42E+06	1.52E+06	1.74E+06
1.10E+05	9.20E+04	6.58E+04	8.52E+04
2.48E+05	2.32E+05	2.03E+05	2.01E+05

435-B	Blank-3	QC-star-run-1	QC-star-run-2
Peak Area	Peak Area	Peak Area	Peak Area
1.07E+08	N/A	1.61E+08	1.57E+08
2.67E+06	N/A	7.55E+06	6.88E+06
7.40E+07	N/A	9.20E+07	9.25E+07
2.08E+08	N/A	4.33E+08	4.06E+08
3.66E+05	N/A	5.41E+05	5.58E+05
9.06E+07	N/A	1.29E+08	1.23E+08
5.37E+07	N/A	6.58E+07	6.20E+07
1.55E+08	N/A	1.86E+08	1.70E+08
1.12E+09	N/A	1.18E+09	1.13E+09
1.22E+09	N/A	1.24E+09	1.20E+09
1.47E+07	N/A	1.86E+07	1.79E+07
1.83E+08	N/A	1.86E+08	1.88E+08
1.01E+09	N/A	1.15E+09	1.15E+09
5.79E+06	N/A	8.40E+06	7.82E+06
2.19E+05	N/A	3.56E+05	3.05E+05
4.39E+07	N/A	6.06E+07	5.66E+07
2.78E+05	N/A	3.66E+05	3.28E+05
2.76E+07	N/A	2.82E+07	2.66E+07
3.97E+08	N/A	5.33E+08	5.36E+08
3.03E+07	3.87E+05	3.50E+07	3.49E+07
5.37E+06	N/A	7.25E+06	7.24E+06
3.02E+06	N/A	5.33E+06	4.93E+06
1.46E+07	N/A	1.15E+07	1.07E+07
1.80E+07	N/A	2.03E+07	1.90E+07
3.07E+08	N/A	3.98E+08	4.08E+08
6.85E+05	N/A	1.00E+06	1.03E+06
3.17E+07	N/A	4.69E+07	4.55E+07
9.45E+07	N/A	1.15E+08	1.15E+08
5.73E+08	N/A	6.97E+08	7.35E+08
3.66E+07	N/A	3.72E+07	3.82E+07
1.60E+08	N/A	2.14E+08	2.03E+08
7.68E+05	N/A	8.10E+05	7.90E+05
1.05E+08	N/A	1.16E+08	1.22E+08
2.26E+08	9.10E+05	2.63E+08	2.79E+08
6.48E+08	N/A	6.99E+08	6.77E+08
8.28E+04	N/A	6.98E+04	8.78E+04
2.57E+07	N/A	3.17E+07	2.91E+07
5.58E+07	N/A	4.36E+07	4.05E+07
1.53E+06	N/A	1.80E+06	1.83E+06
1.79E+06	N/A	1.51E+06	1.53E+06
7.19E+06	N/A	8.86E+06	8.41E+06
2.28E+06	N/A	3.23E+06	3.16E+06

1.96E+07	N/A	2.92E+07	2.50E+07
3.93E+08	N/A	4.66E+08	4.56E+08
3.55E+08	N/A	4.37E+08	4.13E+08
6.58E+08	N/A	8.82E+07	8.08E+07
7.85E+05	N/A	1.30E+06	1.40E+06
1.78E+06	N/A	2.23E+06	2.30E+06
4.28E+05	N/A	3.50E+05	2.89E+05
5.75E+06	N/A	6.81E+06	6.12E+06
4.24E+07	N/A	7.89E+07	7.39E+07
1.14E+07	N/A	1.85E+07	1.74E+07
1.02E+07	N/A	5.28E+06	5.05E+06
6.61E+06	N/A	7.54E+06	7.86E+06
2.16E+07	N/A	1.13E+07	1.12E+07
9.48E+05	N/A	6.39E+05	6.87E+05
1.27E+06	N/A	5.98E+05	6.10E+05
7.66E+06	N/A	9.37E+06	9.55E+06
4.71E+08	N/A	3.70E+08	3.36E+08
4.39E+06	N/A	5.30E+06	5.47E+06
2.61E+07	N/A	1.46E+07	1.37E+07
1.14E+06	N/A	1.68E+06	1.54E+06
6.26E+06	N/A	4.46E+06	4.84E+06
2.89E+06	N/A	7.69E+06	7.34E+06
1.44E+05	N/A	2.52E+05	1.83E+05
8.62E+04	N/A	2.02E+05	2.15E+05
2.47E+06	N/A	2.18E+06	2.10E+06
1.45E+05	N/A	2.39E+05	1.61E+05
1.00E+07	N/A	1.26E+07	1.17E+07
1.66E+05	N/A	1.86E+05	1.64E+05
3.05E+05	N/A	3.65E+05	3.44E+05
3.22E+06	N/A	4.39E+06	4.31E+06
1.16E+04	N/A	1.82E+04	1.61E+04
6.34E+05	N/A	7.84E+05	8.40E+05
5.36E+05	7.01E+02	7.61E+05	6.25E+05
3.52E+06	7.02E+02	4.90E+06	5.28E+06
2.05E+06	N/A	2.89E+06	2.57E+06
1.31E+07	N/A	1.87E+07	1.76E+07
6.72E+06	N/A	8.15E+06	7.57E+06
3.82E+05	N/A	5.87E+05	5.16E+05
1.85E+06	N/A	2.66E+06	2.63E+06
6.76E+05	N/A	7.68E+05	7.80E+05
6.04E+05	N/A	2.28E+05	2.58E+05
1.11E+08	N/A	1.59E+08	1.45E+08
2.42E+06	N/A	3.11E+06	2.96E+06

0.0	0.0	0.0	0.0
0.0	0.0	0.0	0.0

435-B	Blank-3	QC-star-run-1	QC-star-run-2
Peak Area	Peak Area	Peak Area	Peak Area
4.47E+06	N/A	4.61E+06	4.58E+06
8.27E+06	N/A	6.85E+06	7.32E+06
7.98E+08	N/A	5.36E+08	5.45E+08
7.58E+05	N/A	1.21E+06	1.21E+06
2.82E+06	N/A	2.19E+06	2.50E+06
2.17E+05	N/A	4.09E+05	3.93E+05
4.69E+06	N/A	7.14E+06	7.31E+06
1.34E+06	N/A	1.73E+06	1.60E+06
1.53E+08	N/A	2.44E+08	2.38E+08
2.73E+05	N/A	3.32E+05	3.07E+05
6.38E+05	N/A	6.78E+05	6.68E+05
6.19E+05	N/A	6.10E+05	5.44E+05
2.57E+07	N/A	2.15E+07	2.37E+07
1.34E+07	N/A	1.78E+07	1.85E+07
3.94E+05	N/A	4.68E+05	5.25E+05
3.24E+06	N/A	6.17E+06	6.11E+06
2.07E+07	N/A	2.17E+07	2.19E+07
1.59E+05	N/A	2.17E+05	1.78E+05
1.39E+05	N/A	1.08E+05	1.49E+05
5.66E+06	N/A	5.09E+06	4.78E+06
3.21E+08	N/A	4.29E+08	4.06E+08
3.91E+06	N/A	2.46E+06	1.88E+06
5.66E+06	N/A	3.88E+06	2.96E+06
2.35E+07	N/A	1.54E+07	1.56E+07
2.31E+05	N/A	1.63E+05	1.70E+05
1.38E+06	N/A	1.54E+06	1.65E+06
3.45E+06	N/A	4.74E+06	4.75E+06
8.07E+05	N/A	1.02E+06	1.03E+06
6.31E+04	N/A	5.90E+04	6.82E+04
2.99E+06	N/A	3.97E+06	4.15E+06
7.60E+06	N/A	1.03E+07	1.03E+07
1.10E+06	N/A	1.01E+06	1.02E+06
5.41E+08	3.83E+07	5.46E+08	6.09E+08
5.36E+06	9.49E+04	3.54E+06	3.61E+06
2.44E+08	1.26E+07	1.95E+08	2.06E+08
4.26E+06	N/A	4.60E+06	4.52E+06
3.52E+06	N/A	1.81E+06	1.83E+06
3.83E+06	N/A	4.21E+06	4.35E+06
1.42E+05	N/A	8.87E+04	5.60E+04

2.41E+04	N/A	6.54E+04	7.99E+04
1.06E+06	N/A	8.40E+05	7.41E+05
1.99E+05	N/A	2.12E+05	2.01E+05
3.11E+05	N/A	3.10E+05	2.11E+05
1.58E+06	N/A	1.02E+07	1.04E+07
9.68E+05	N/A	1.37E+06	1.14E+06
6.22E+06	N/A	2.55E+06	2.44E+06
1.95E+05	N/A	1.50E+05	1.58E+05
6.24E+06	N/A	2.68E+06	2.65E+06
3.19E+06	N/A	2.48E+06	2.37E+06
1.18E+05	N/A	1.53E+05	1.63E+05
1.80E+05	N/A	7.91E+04	9.03E+04
3.21E+06	N/A	1.42E+06	1.33E+06
4.48E+04	N/A	1.26E+04	1.78E+04
1.30E+03	N/A	N/A	9.71E+02
2.70E+05	N/A	6.85E+05	6.34E+05
2.15E+05	N/A	1.06E+05	1.07E+05
6.60E+05	N/A	2.39E+05	2.35E+05
3.93E+05	N/A	2.15E+05	2.03E+05
1.91E+06	N/A	1.64E+06	1.52E+06
2.30E+06	N/A	3.10E+06	3.05E+06
9.30E+04	N/A	7.97E+04	9.01E+04
2.11E+05	N/A	2.37E+05	2.12E+05

QC-end-run-1	QC-end-run-2
Peak Area	Peak Area
1.36E+08	1.32E+08
6.20E+06	6.55E+06
8.14E+07	8.02E+07
3.75E+08	3.99E+08
4.70E+05	5.09E+05
1.13E+08	1.18E+08
5.66E+07	5.53E+07
1.57E+08	1.70E+08
1.08E+09	1.12E+09
1.16E+09	1.22E+09
1.71E+07	1.82E+07
1.82E+08	1.76E+08
1.13E+09	1.12E+09
7.89E+06	7.69E+06
3.33E+05	3.35E+05
5.76E+07	5.50E+07
3.04E+05	3.29E+05
2.40E+07	2.39E+07
5.28E+08	5.38E+08
3.15E+07	3.25E+07
6.03E+06	6.50E+06
4.65E+06	4.52E+06
9.90E+06	1.06E+07
1.82E+07	1.86E+07
3.69E+08	3.89E+08
9.15E+05	9.85E+05
4.30E+07	4.36E+07
1.05E+08	1.08E+08
6.69E+08	6.63E+08
3.46E+07	3.67E+07
1.91E+08	1.94E+08
6.69E+05	7.05E+05
1.22E+08	1.13E+08
2.50E+08	2.41E+08
6.69E+08	6.76E+08
7.40E+04	8.21E+04
2.90E+07	3.08E+07
3.79E+07	3.81E+07
1.72E+06	1.61E+06
1.47E+06	1.54E+06
7.95E+06	8.45E+06
2.90E+06	3.06E+06

2.64E+07	2.46E+07
4.91E+08	4.59E+08
4.00E+08	4.20E+08
8.46E+07	8.12E+07
1.16E+06	1.31E+06
2.34E+06	2.25E+06
2.97E+05	4.29E+05
6.27E+06	6.27E+06
7.49E+07	7.84E+07
1.70E+07	1.80E+07
4.86E+06	4.83E+06
6.88E+06	6.99E+06
9.81E+06	9.43E+06
6.60E+05	5.80E+05
5.87E+05	6.01E+05
8.25E+06	8.29E+06
3.35E+08	3.34E+08
5.13E+06	4.88E+06
1.45E+07	1.36E+07
1.51E+06	1.44E+06
4.41E+06	4.16E+06
6.97E+06	7.38E+06
2.36E+05	1.71E+05
1.94E+05	1.63E+05
2.20E+06	1.80E+06
2.78E+05	2.14E+05
1.09E+07	1.17E+07
1.46E+05	1.52E+05
3.15E+05	3.16E+05
3.99E+06	4.06E+06
1.48E+04	9.82E+03
7.87E+05	7.57E+05
5.83E+05	6.45E+05
4.81E+06	4.71E+06
2.44E+06	2.52E+06
1.77E+07	1.83E+07
6.99E+06	7.51E+06
5.17E+05	5.30E+05
2.55E+06	2.54E+06
7.56E+05	7.03E+05
2.47E+05	2.40E+05
1.38E+08	1.46E+08
2.77E+06	3.04E+06

0.0	0.0
0.0	0.0

QC-end-run-1	QC-end-run-2	
Peak Area	Peak Area	
5.13E+06	5.08E+06	
7.26E+06	7.29E+06	
5.73E+08	5.62E+08	
1.26E+06	1.12E+06	
2.43E+06	2.47E+06	
4.40E+05	3.96E+05	
7.32E+06	7.33E+06	
1.97E+06	1.65E+06	
2.35E+08	2.41E+08	
3.54E+05	3.39E+05	
7.87E+05	7.78E+05	
6.43E+05	5.92E+05	
2.26E+07	2.36E+07	
1.63E+07	1.81E+07	
5.42E+05	4.83E+05	
6.59E+06	6.39E+06	
2.21E+07	2.33E+07	
2.30E+05	2.04E+05	
1.55E+05	1.43E+05	
5.05E+06	5.10E+06	
4.20E+08	4.17E+08	
2.21E+06	2.72E+06	
3.27E+06	3.79E+06	Retention time of highlighted metabolites n[illegible]
1.64E+07	1.63E+07	
1.84E+05	1.22E+05	
1.42E+06	1.52E+06	
4.60E+06	4.93E+06	
1.08E+06	1.08E+06	
6.48E+04	7.49E+04	
4.00E+06	4.25E+06	
1.14E+07	1.09E+07	
1.15E+06	1.10E+06	
5.81E+08	6.59E+08	
3.79E+06	4.09E+06	
2.19E+08	2.11E+08	
4.66E+06	4.71E+06	
1.70E+06	1.76E+06	
4.64E+06	4.65E+06	
8.98E+04	1.39E+05	

6.56E+04	7.54E+04
7.85E+05	7.27E+05
2.26E+05	2.15E+05
2.13E+05	2.61E+05
9.95E+06	1.09E+07
1.29E+06	1.35E+06
2.54E+06	2.31E+06
1.72E+05	1.66E+05
2.98E+06	2.92E+06
2.51E+06	2.39E+06
1.75E+05	2.03E+05
6.68E+04	9.30E+04
1.45E+06	1.42E+06
1.24E+04	9.12E+03
N/A	N/A
6.22E+05	6.32E+05
1.07E+05	1.01E+05
2.25E+05	2.37E+05
2.03E+05	2.13E+05
1.65E+06	1.58E+06
2.94E+06	3.24E+06
7.16E+04	9.46E+04
2.52E+05	2.26E+05

eed to be confirmed with pure standard. Please provide us with 5mg of their pure standard.

Sample Name	Polarity
Urea(61.1/44.2/RT(min):5.12)	Positive
ethanolamine(62.1/44.2/RT(min):12.53)	Positive
alanine(90.1/44.2/RT(min):7.79)	Positive
choline(104/60/RT(min):12.15	Positive
4-aminobutyrate(104.01/69/RT(min):8.27)	Positive
dimethylglycine(104.02/58/RT(min):5.92)	Positive
serine(106/60/RT(min):8.48)	Positive
creatinine(114/44.2/RT(min):4.53)	Positive
proline(116.1/70.1/RT(min):6.38)	Positive
betaine(118.02/58/RT(min):5.33)	Positive
valine(118.1/55.2/RT(min):6.08)	Positive
threonine(120/74/RT(min):7.42)	Positive
creatine(132.003/90/RT(min):7.78)	Positive
nicotinamide(123.1/80/RT(min):3.55)	Positive
thymine(127.1/110/RT(min):3.37)	Positive
DL-Pipecolic acid(130/84/RT(min):6.05)	Positive
N-Acetylputrescine(131.001/114/RT(min):11.70)	Positive
hydroxyproline(132.004/68.2/RT(min):7.66)	Positive
leucine(132.1/86/RT(min):5.07)	Positive
ornithine(133/70/RT(min):12.81)	Positive
asparagine(133.1/74/RT(min):8.18)	Positive
aspartate(134/74/RT(min):8.58)	Positive
methylnicotinamide(137.001/94/RT(min):14.07)	Positive
lysine(147/67/RT(min):14.08)	Positive
glutamine(147.1/84.1/RT(min):8.05)	Positive
O-acetyl-L-serine(148/106/RT(min):4.87)	Positive
glutamate(148.1/84.1/RT(min):8.21)	Positive
methionine(150.1/133/RT(min):5.46)	Positive
carnitine(162.1/103/RT(min):6.65)	Positive
Methionine sulfoxide(166/74/RT(min):7.00)	Positive
phenylalanine(166.1/103/RT(min):4.63)	Positive
pyridoxine(170/134/RT(min):3.65)	Positive
1-Methyl-Histidine(170.1/124/RT(min):7.52)	Positive
arginine(175.02/60/RT(min):14.59)	Positive
citrulline(176/159/RT(min):8.66)	Positive
N-carbamoyl-L-aspartate(177.05/74/RT(min):10.28)	Positive
tyrosine(182.1/77/RT(min):6.64)	Positive
Phosphorylcholine(184.001/125/RT(min):8.38)	Positive
N6-Acetyl-L-lysine(189.001/84.2/RT(min):7.97)	Positive
Acetyllysine(189.002/84/RT(min):7.97)	Positive
N-acetyl-glutamine(189.1/130/RT(min):4.96)	Positive
N-acetyl-glutamate (190.1/84.1/RT(min):7.89)	Positive

Ng_Ng-dimethyl-L-arginine(203/70/RT(min):12.15)	Positive
Acetylcarnitine DL(204/85/RT(min):5.17)	Positive
tryptophan(205/146/RT(min):5.74)	Positive
Kynurenine(209/146/RT(min):5.17)	Positive
N-acetyl-glucosamine(222/138/RT(min):5.74)	Positive
cystathionine(223/134/RT(min):9.64)	Positive
5-methoxytryptophan(235/176/RT(min):5.21)	Positive
Cystine(241.002/74/RT(min):9.08)	Positive
cytidine(244.1/112/RT(min):5.95)	Positive
thiamine(265/122/RT(min):12.39)	Positive
adenosine(268.15/136.1/RT(min):4.20)	Positive
L-arginino-succinate(291/70/RT(min):9.96)	Positive
glutathione (308.1/162/RT(min):7.8)	Positive
UMP(325/97/RT(min):8.91)	Positive
dAMP(332.1/136/RT(min):6.78)	Positive
thiamine-phosphate(345.2/122/RT(min):7.62)	Positive
AMP(348.15/136/RT(min):7.87)	Positive
IMP(349/137/RT(min):9.31)	Positive
GMP(364/152/RT(min):10.18)	Positive
riboflavin(377/243/RT(min):3.94)	Positive
S-adenosyl-L-homoCysteine_pos(385.1/136/RT(min):6.97)	Positive
S-adenosyl-L-methionine(399.1/250/RT(min):9.1)	Positive
folate(442/295/RT(min):10.47)	Positive
7_8-dihydrofolate(444.2/178/RT(min):9.86)	Positive
glutathione disulfide_pos(613/231/RT(min):10.30)	Positive
FAD(786/348/RT(min):6.25)	Positive
Adenylosuccinate-1(464/252/RT(min): 11.58)	Positive
Isoleucine(132.1/86/RT(min):5.33)	Positive
glycolate(75/45.2/RT(min):2.90)	Negative
pyruvate(87/43/RT(min):3.55)	Negative
lactate(89/43.2/RT(min):4.09)	Negative
glycerate(105/75/RT(min):5.81)	Negative
fumarate(115/71/RT(min):9.91)	Negative
Maleic acid(115.03/71.03/RT(min):4.45)	Negative
succinate(117/73/RT(min):9.10)	Negative
nicotinate(122/78/RT(min):3.60)	Negative
taurine(124/80/RT(min):7.72)	Negative
Pyroglutamic acid(128/82.1/RT(min):4.60)	Negative
N-Acetyl-L-alanine(130/88/RT(min):3.62)	Negative
Hydroxyisocaproic acid(131.006/85.1/RT(min):2.30)	Negative
malate(133/115/RT(min):9.78)	Negative
a-ketoglutarate(145/101/RT(min):9.42)	Negative
3-methylphenylacetic acid(149.002/105/RT(min):2.34*)	Negative
orotate(155/111/RT(min):4.71)	Negative

allantoin(157.05/114/RT(min):7.19)	Negative
phenylpyruvate(163/91/RT(min):2.38)	Negative
Phenyllactic acid(165.006/103.1/RT(min):2.30)	Negative
quinolinate(166/122/RT(min):9.13)	Negative
Uric acid(167.001/124/RT(min):6.71)	Negative
dihydroxy-acetone-phosphate(169/79/RT(min):9.03)	Negative
D-glyceraldehdye-3-phosphate(169.05/97/RT(min):9.03)	Negative
aconitate(173.05/85/RT(min):11.22)	Negative
allantoate(175/132/RT(min):8.35)	Negative
N-carbamoyl-L-aspartate_neg(175.03/132/RT(min):10.28)	Negative
myo-inositol(179/161/RT(min):9.34)	Negative
hydroxyphenylpyruvate(179.05/107/RT(min):3.59)	Negative
homocysteic acid(182/80/RT(min):9.63)	Negative
4-Pyridoxic acid(182.003/138/RT(min):2.26)	Negative
Indoleacrylic acid(186/142.03/RT(min):3.60)	Negative
Kynurenic acid(188/144/RT(min):2.32)	Negative
citrate-isocitrate(191/111/RT(min):12)	Negative
isocitrate (191.02/117/RT(min):12.12)	Negative
citrate (191.05/87/RT(min):12.12)	Negative
Xanthurenic acid(204.001/160/RT(min):5.65)	Negative
D-glucarate(209/85/RT(min):10.20)	Negative
pantothenate(218/146/RT(min):3.59)	Negative
thymidine(241/125/RT(min):3.46)	Negative
uridine(243/200/RT(min):4.70)	Negative
glucose-6-phosphate(259.02/199/RT(min):10.38)	Negative
inosine(267/135/RT(min):5.41)	Negative
6-phospho-D-gluconate(275/97/RT(min):11.53)	Negative
xanthosine(283/151/RT(min):6.41)	Negative
N-acetyl-glucosamine-1-phosphate(300/79/RT(min):8.6)	Negative
glutathione_neg(306/143/RT(min):7.9)	Negative
cyclic-AMP(328/134.05/RT(min):4.45)	Negative
sucrose(341/179/RT(min):*8.09)	Negative
S-adenosyl-L-homocysteine_neg(383.1/134/RT(min):6.97)	Negative
Deoxycholic acid(391.202/345.2/RT(min):2.11)	Negative
UDP_neg(403/159/RT(min):10.38)	Negative
ADP_neg(426.1/159/RT(min):9.38)	Negative
GDP_neg(442/159/RT(min):11.48)	Negative
dATP_neg(490/159/RT(min):9.95)	Negative
Taurodeoxycholic acid(498.2/124/RT(min):2.0)	Negative
ATP_neg(506.1/159/RT(min):10.72)	Negative
UDP-D-glucose(565/323/RT(min):10.05)	Negative
UDP-N-acetyl-glucosamine(606/385/RT(min):9.04)	Negative
glutathione disulfide_neg(611/306/RT(min):10.3*)	Negative
2-hydroxygluterate(147.1/128.7/RT(min):9.18)	Negative

401-A	402-A	403-A	433-B
Peak Area	Peak Area	Peak Area	Peak Area
1.52E+08	1.60E+08	1.51E+08	9.85E+07
8.08E+06	8.36E+06	1.34E+07	3.40E+06
8.83E+07	9.79E+07	9.54E+07	7.46E+07
4.16E+08	4.41E+08	6.50E+08	2.32E+08
4.31E+05	2.48E+05	1.62E+06	1.70E+05
1.19E+08	1.36E+08	1.23E+08	1.05E+08
5.13E+07	6.11E+07	7.60E+07	4.60E+07
1.61E+08	1.67E+08	1.77E+08	1.56E+08
1.08E+09	1.12E+09	1.19E+09	1.03E+09
1.15E+09	1.23E+09	1.32E+09	1.16E+09
1.69E+07	1.95E+07	1.63E+07	1.47E+07
1.41E+08	1.60E+08	1.64E+08	2.00E+08
1.06E+09	1.25E+09	1.24E+09	1.08E+09
5.84E+06	8.68E+06	1.29E+07	2.64E+06
2.54E+05	3.27E+05	3.54E+05	1.86E+05
5.51E+07	5.76E+07	4.78E+07	3.80E+07
2.56E+05	3.20E+05	3.01E+05	2.14E+05
1.51E+07	2.26E+07	2.51E+07	3.11E+07
5.69E+08	5.72E+08	5.86E+08	3.57E+08
4.22E+07	3.55E+07	2.94E+07	2.80E+07
9.85E+06	9.02E+06	1.00E+07	5.21E+06
4.49E+06	3.88E+06	1.11E+07	2.46E+06
7.25E+06	1.21E+07	4.79E+06	6.41E+06
1.91E+07	1.81E+07	2.09E+07	1.47E+07
5.44E+08	5.52E+08	6.33E+08	2.68E+08
8.80E+05	1.02E+06	1.21E+06	6.85E+05
6.61E+07	6.35E+07	9.97E+07	2.65E+07
1.17E+08	1.19E+08	1.19E+08	8.84E+07
6.46E+08	8.46E+08	7.48E+08	5.64E+08
3.53E+07	3.40E+07	4.42E+07	2.57E+07
1.98E+08	2.10E+08	2.24E+08	1.57E+08
6.95E+05	1.19E+06	3.65E+05	6.35E+05
8.37E+07	1.00E+08	1.03E+08	1.23E+08
2.13E+08	2.33E+08	2.78E+08	2.08E+08
5.58E+08	5.85E+08	5.95E+08	6.46E+08
3.89E+04	4.87E+04	5.40E+04	8.44E+04
2.71E+07	3.22E+07	3.21E+07	2.51E+07
1.11E+07	1.32E+07	1.45E+07	5.47E+07
2.69E+06	3.07E+06	2.63E+06	1.03E+06
2.09E+06	2.44E+06	2.20E+06	9.11E+05
7.62E+06	9.59E+06	8.74E+06	3.89E+06
3.71E+06	4.15E+06	5.37E+06	2.50E+06

2.34E+07	2.74E+07	3.15E+07	1.98E+07
4.00E+08	5.24E+08	4.37E+08	2.96E+08
4.19E+08	4.26E+08	4.38E+08	3.04E+08
5.14E+06	5.56E+06	5.78E+06	7.21E+08
1.53E+06	1.42E+06	2.14E+06	8.48E+05
1.35E+06	2.35E+06	3.76E+06	1.79E+06
2.15E+05	2.91E+05	3.42E+05	2.31E+05
5.71E+06	6.02E+06	6.80E+06	3.53E+06
5.97E+07	8.39E+07	1.07E+08	7.08E+07
1.93E+07	2.18E+07	2.72E+07	8.51E+06
1.30E+06	2.56E+06	6.03E+05	6.36E+05
3.03E+06	4.38E+06	7.06E+06	5.64E+06
2.89E+06	3.81E+06	4.50E+06	4.70E+06
N/A	N/A	N/A	7.02E+05
N/A	N/A	N/A	5.54E+05
5.91E+06	9.77E+06	6.61E+06	6.17E+06
3.63E+05	3.39E+05	5.23E+05	2.59E+08
N/A	N/A	N/A	6.37E+06
1.83E+04	2.51E+04	1.06E+05	1.79E+07
8.60E+05	1.15E+06	2.05E+06	1.15E+06
3.39E+06	4.18E+06	3.27E+06	3.49E+06
1.15E+07	1.37E+07	2.43E+07	1.95E+06
7.61E+04	2.78E+05	7.49E+04	2.56E+05
1.40E+05	1.92E+05	2.29E+05	2.07E+05
2.15E+05	4.18E+05	2.27E+06	2.95E+06
1.63E+05	1.16E+05	3.27E+05	1.47E+05
N/A	N/A	N/A	1.97E+05
1.84E+08	1.75E+08	1.53E+08	1.34E+08
5.16E+06	4.81E+06	5.14E+06	4.70E+06
8.90E+06	9.82E+06	5.48E+06	7.59E+06
4.21E+08	4.98E+08	3.74E+08	7.15E+08
1.47E+06	2.19E+06	2.37E+06	4.65E+05
5.02E+06	4.09E+06	3.91E+06	1.32E+06
3.25E+05	2.60E+05	2.21E+05	1.85E+05
4.30E+06	4.77E+06	5.19E+06	4.16E+06
6.75E+05	3.98E+06	1.61E+06	8.69E+05
2.85E+08	2.90E+08	3.99E+08	1.53E+08
2.80E+05	4.22E+05	4.61E+05	2.76E+05
7.19E+05	7.84E+05	9.50E+05	5.95E+05
5.03E+05	7.04E+05	6.36E+05	4.93E+05
4.67E+07	3.71E+07	3.60E+07	1.18E+07
2.14E+07	1.86E+07	1.90E+07	1.37E+07
6.65E+05	5.17E+05	6.26E+05	3.42E+05
7.72E+06	8.28E+06	9.71E+06	3.26E+06

1.89E+07	3.27E+07	3.16E+07	1.18E+07
2.71E+05	2.47E+05	1.52E+05	2.51E+05
1.40E+05	2.46E+05	2.05E+05	7.92E+04
4.18E+06	4.35E+06	6.16E+06	4.69E+06
4.34E+08	5.10E+08	6.53E+08	3.34E+08
N/A	N/A	N/A	3.33E+06
N/A	N/A	N/A	5.09E+06
4.50E+06	6.78E+06	5.83E+06	2.27E+07
3.36E+05	4.59E+05	2.86E+05	1.41E+05
1.18E+06	1.78E+06	1.68E+06	1.21E+06
8.47E+06	6.91E+06	1.50E+07	3.17E+06
1.07E+06	1.65E+06	7.45E+05	1.20E+06
5.61E+04	1.02E+05	1.42E+05	5.64E+04
3.35E+06	5.57E+06	5.84E+06	3.38E+06
9.25E+06	8.94E+06	9.73E+06	5.99E+06
7.22E+05	8.58E+05	8.75E+05	9.72E+05
4.13E+08	6.17E+08	5.33E+08	6.57E+08
9.35E+05	1.58E+06	1.21E+06	5.34E+06
1.40E+08	1.93E+08	1.75E+08	2.28E+08
4.27E+06	4.73E+06	4.81E+06	3.71E+06
2.82E+05	7.88E+05	5.57E+05	2.21E+06
4.35E+06	6.07E+06	6.01E+06	3.65E+06
1.20E+05	7.93E+04	1.97E+05	2.39E+05
1.03E+05	1.26E+05	1.79E+05	1.75E+04
2.03E+05	3.22E+05	4.55E+05	7.65E+05
1.19E+05	2.32E+05	3.15E+05	8.08E+04
2.29E+05	1.97E+05	1.97E+05	2.78E+05
1.85E+07	2.19E+07	2.28E+07	1.19E+06
7.99E+05	1.23E+06	2.26E+06	7.79E+05
6.17E+05	9.33E+05	1.17E+06	1.74E+06
N/A	N/A	N/A	3.31E+05
5.55E+05	2.35E+06	8.98E+06	8.98E+05
1.99E+06	2.30E+06	1.77E+06	2.03E+06
1.87E+05	2.37E+05	1.87E+05	1.28E+05
N/A	N/A	N/A	1.11E+05
N/A	N/A	N/A	1.87E+06
N/A	N/A	N/A	2.33E+04
6.47E+02	N/A	N/A	N/A
5.58E+05	5.75E+05	1.14E+06	9.32E+05
N/A	N/A	N/A	2.27E+05
N/A	N/A	N/A	6.01E+05
N/A	N/A	N/A	3.83E+05
2.42E+05	4.46E+05	2.35E+06	2.49E+06
3.09E+06	2.44E+06	3.42E+06	1.52E+06

434-B	435-B	Blank-3
Peak Area	Peak Area	Peak Area
1.03E+08	1.07E+08	N/A
2.62E+06	2.67E+06	N/A
7.34E+07	7.40E+07	N/A
1.68E+08	2.08E+08	N/A
1.97E+05	3.66E+05	N/A
1.07E+08	9.06E+07	N/A
4.63E+07	5.37E+07	N/A
1.51E+08	1.55E+08	N/A
1.00E+09	1.12E+09	N/A
1.35E+09	1.22E+09	N/A
1.41E+07	1.47E+07	N/A
1.67E+08	1.83E+08	N/A
1.15E+09	1.01E+09	N/A
4.85E+06	5.79E+06	N/A
2.20E+05	2.19E+05	N/A
5.37E+07	4.39E+07	N/A
2.54E+05	2.78E+05	N/A
2.11E+07	2.76E+07	N/A
3.47E+08	3.97E+08	N/A
2.45E+07	3.03E+07	3.87E+05
4.06E+06	5.37E+06	N/A
2.53E+06	3.02E+06	N/A
9.47E+06	1.46E+07	N/A
1.39E+07	1.80E+07	N/A
2.94E+08	3.07E+08	N/A
7.84E+05	6.85E+05	N/A
2.48E+07	3.17E+07	N/A
8.11E+07	9.45E+07	N/A
5.71E+08	5.73E+08	N/A
3.43E+07	3.66E+07	N/A
1.54E+08	1.60E+08	N/A
3.58E+05	7.68E+05	N/A
1.03E+08	1.05E+08	N/A
2.01E+08	2.26E+08	9.10E+05
6.97E+08	6.48E+08	N/A
8.13E+04	8.28E+04	N/A
2.34E+07	2.57E+07	N/A
5.47E+07	5.58E+07	N/A
1.27E+06	1.53E+06	N/A
1.08E+06	1.79E+06	N/A
6.18E+06	7.19E+06	N/A
2.97E+06	2.28E+06	N/A

2.07E+07	1.96E+07	N/A
4.88E+08	3.93E+08	N/A
3.36E+08	3.55E+08	N/A
6.68E+08	6.58E+08	N/A
5.73E+05	7.85E+05	N/A
1.53E+06	1.78E+06	N/A
3.36E+05	4.28E+05	N/A
5.89E+06	5.75E+06	N/A
5.66E+07	4.24E+07	N/A
9.18E+06	1.14E+07	N/A
5.85E+06	1.02E+07	N/A
5.93E+06	6.61E+06	N/A
1.08E+07	2.16E+07	N/A
1.19E+06	9.48E+05	N/A
7.04E+05	1.27E+06	N/A
7.23E+06	7.66E+06	N/A
5.13E+08	4.71E+08	N/A
1.47E+07	4.39E+06	N/A
2.38E+07	2.61E+07	N/A
1.34E+06	1.14E+06	N/A
5.48E+06	6.26E+06	N/A
2.77E+06	2.89E+06	N/A
1.19E+05	1.44E+05	N/A
1.16E+05	8.62E+04	N/A
2.74E+06	2.47E+06	N/A
1.67E+05	1.45E+05	N/A
2.49E+05	6.04E+05	N/A
1.24E+08	1.11E+08	N/A
4.84E+06	4.47E+06	N/A
7.95E+06	8.27E+06	N/A
7.13E+08	7.98E+08	N/A
6.10E+05	7.58E+05	N/A
1.26E+06	2.82E+06	N/A
1.93E+05	2.17E+05	N/A
3.55E+06	4.69E+06	N/A
8.48E+05	1.34E+06	N/A
1.72E+08	1.53E+08	N/A
3.62E+05	2.73E+05	N/A
5.49E+05	6.38E+05	N/A
4.82E+05	6.19E+05	N/A
1.22E+07	2.57E+07	N/A
1.13E+07	1.34E+07	N/A
4.45E+05	3.94E+05	N/A
5.23E+06	3.24E+06	N/A

1.52E+07	2.07E+07	N/A	
1.66E+05	1.59E+05	N/A	
8.48E+04	1.39E+05	N/A	
4.21E+06	5.66E+06	N/A	
3.75E+08	3.21E+08	N/A	
4.71E+06	3.91E+06	N/A	
7.43E+06	5.66E+06	N/A	Retention time of highli
2.71E+07	2.35E+07	N/A	
1.86E+05	2.31E+05	N/A	
1.30E+06	1.38E+06	N/A	
3.01E+06	3.45E+06	N/A	
5.71E+05	8.07E+05	N/A	
5.88E+04	6.31E+04	N/A	
2.44E+06	2.99E+06	N/A	
1.59E+07	7.60E+06	N/A	
2.21E+06	1.10E+06	N/A	
6.87E+08	5.41E+08	3.83E+07	
6.96E+06	5.36E+06	9.49E+04	
2.24E+08	2.44E+08	1.26E+07	
3.99E+06	4.26E+06	N/A	
2.30E+06	3.52E+06	N/A	
3.37E+06	3.83E+06	N/A	
1.54E+05	1.42E+05	N/A	
2.27E+04	2.41E+04	N/A	
1.04E+06	1.06E+06	N/A	
2.09E+05	1.99E+05	N/A	
3.48E+05	3.11E+05	N/A	
1.61E+06	1.58E+06	N/A	
8.50E+05	9.68E+05	N/A	
2.95E+06	6.22E+06	N/A	
2.32E+05	1.95E+05	N/A	
5.88E+05	6.24E+06	N/A	
3.34E+06	3.19E+06	N/A	
1.03E+05	1.18E+05	N/A	
2.02E+05	1.80E+05	N/A	
2.68E+06	3.21E+06	N/A	
2.11E+04	4.48E+04	N/A	
9.72E+02	1.30E+03	N/A	
2.86E+05	2.70E+05	N/A	
1.84E+05	2.15E+05	N/A	
2.26E+05	6.60E+05	N/A	
6.98E+05	3.93E+05	N/A	
1.53E+06	1.91E+06	N/A	
1.74E+06	2.30E+06	N/A	

Sample Name	Polarity
13C-Proline1(122.00/75.00/RT(min):6.38)	Positive
13C-Alanine-1(93.94/47.00/RT(min):7.79)	Positive
13C-Serine-1(110.05/63.00/RT(min):8.48)	Positive
13C-Valine-1(123.93/77.10/RT(min):6.08)	Positive
13C-Glycine-1(78.91/32.00/RT(min):8.53)	Positive
13C-Cystine-1(248.89/155.90/RT(min):9.08)	Positive
13C-Glutamic acid-1(154.05/89.00/RT(min):8.22)	Positive
13C-Leucine-1(138.99/92.00/RT(min):5.07)	Positive
13C-Methionine-1(155.90/138.00/RT(min):5.46)	Positive
13C-Phenylalanine-1(176.01/128.90/RT(min):4.60)	Positive
13C-Tyrosine(192.01/174.00/RT(min):6.64)	Positive
13C tyrosine(192.00/83.00/RT(min):6.64)	Positive
13 C phenylalanine(176.00/111.00/RT(min):4.60)	Positive
13C lysine(155.00/90.10/RT(min):14.08)	Positive
13C-IsoLeucine-1(138.99/92.00/RT(min):5.33)	Positive

Sample Name	Polarity
13C-Serine(107.9/77.0/RT(min):8.49)	Negative
13C-Methionine(153.9/48.1/RT(min):5.46)	Negative

401-A	402-A	403-A	433-B
Peak Area	Peak Area	Peak Area	Peak Area
1.14E+07	1.08E+07	1.00E+07	1.18E+07
1.91E+05	1.56E+05	1.65E+05	1.47E+05
3.74E+05	3.36E+05	2.86E+05	3.02E+05
4.07E+06	4.02E+06	3.61E+06	4.01E+06
1.58E+04	1.58E+04	2.10E+04	1.23E+04
7.95E+05	8.65E+05	7.12E+05	8.01E+05
9.70E+05	8.83E+05	7.46E+05	5.82E+05
5.56E+06	4.84E+06	5.04E+06	3.46E+06
3.03E+06	2.52E+06	2.26E+06	2.24E+06
1.96E+07	1.79E+07	1.60E+07	1.40E+07
9.88E+06	8.93E+06	8.27E+06	6.06E+06
7.30E+05	6.46E+05	6.55E+05	3.68E+05
2.89E+06	2.52E+06	2.17E+06	1.97E+06
7.13E+05	7.35E+05	6.78E+05	6.71E+05
3.82E+06	3.08E+06	2.81E+06	2.78E+06

401-A	402-A	403-A	433-B
Peak Area	Peak Area	Peak Area	Peak Area
1.30E+05	1.10E+05	9.20E+04	6.58E+04
2.60E+05	2.48E+05	2.32E+05	2.03E+05

434-B	435-B	QC-star-run-1	QC-star-run-2
Peak Area	Peak Area	Peak Area	Peak Area
1.23E+07	1.00E+07	1.26E+07	1.17E+07
1.42E+05	1.66E+05	1.86E+05	1.64E+05
3.05E+05	3.05E+05	3.65E+05	3.44E+05
3.87E+06	3.22E+06	4.39E+06	4.31E+06
1.58E+04	1.16E+04	1.82E+04	1.61E+04
6.72E+05	6.34E+05	7.84E+05	8.40E+05
4.41E+05	5.36E+05	7.61E+05	6.25E+05
3.69E+06	3.52E+06	4.90E+06	5.28E+06
2.07E+06	2.05E+06	2.89E+06	2.57E+06
1.46E+07	1.31E+07	1.87E+07	1.76E+07
6.24E+06	6.72E+06	8.15E+06	7.57E+06
4.21E+05	3.82E+05	5.87E+05	5.16E+05
1.99E+06	1.85E+06	2.66E+06	2.63E+06
5.89E+05	6.76E+05	7.68E+05	7.80E+05
2.76E+06	2.42E+06	3.11E+06	2.96E+06

434-B	435-B	QC-star-run-1	QC-star-run-2
Peak Area	Peak Area	Peak Area	Peak Area
8.52E+04	9.30E+04	7.97E+04	9.01E+04
2.01E+05	2.11E+05	2.37E+05	2.12E+05

QC-end-run-1	QC-end-run-2	%CV of ISTD
Peak Area	Peak Area	
1.09E+07	1.17E+07	7.7
1.46E+05	1.52E+05	10.2
3.15E+05	3.16E+05	8.8
3.99E+06	4.06E+06	8.5
1.48E+04	9.82E+03	21.6
7.87E+05	7.57E+05	9.5
5.83E+05	6.45E+05	24.0
4.81E+06	4.71E+06	16.4
2.44E+06	2.52E+06	13.1
1.77E+07	1.83E+07	13.2
6.99E+06	7.51E+06	15.8
5.17E+05	5.30E+05	22.7
2.55E+06	2.54E+06	14.8
7.56E+05	7.03E+05	8.1
2.77E+06	3.04E+06	12.3

QC-end-run-1	QC-end-run-2	%CV of ISTD
Peak Area	Peak Area	
7.16E+04	9.46E+04	20.1
2.52E+05	2.26E+05	9.3

Sample Name	Polarity
Urea(61.1/44.2/RT(min):5.12)	Positive
ethanolamine(62.1/44.2/RT(min):12.53)	Positive
alanine(90.1/44.2/RT(min):7.79)	Positive
choline(104/60/RT(min):12.15	Positive
4-aminobutyrate(104.01/69/RT(min):8.27)	Positive
dimethylglycine(104.02/58/RT(min):5.92)	Positive
serine(106/60/RT(min):8.48)	Positive
creatinine(114/44.2/RT(min):4.53)	Positive
proline(116.1/70.1/RT(min):6.38)	Positive
betaine(118.02/58/RT(min):5.33)	Positive
valine(118.1/55.2/RT(min):6.08)	Positive
threonine(120/74/RT(min):7.42)	Positive
creatine(132.003/90/RT(min):7.78)	Positive
nicotinamide(123.1/80/RT(min):3.55)	Positive
thymine(127.1/110/RT(min):3.37)	Positive
DL-Pipecolic acid(130/84/RT(min):6.05)	Positive
N-Acetylputrescine(131.001/114/RT(min):11.70)	Positive
hydroxyproline(132.004/68.2/RT(min):7.66)	Positive
leucine(132.1/86/RT(min):5.07)	Positive
ornithine(133/70/RT(min):12.81)	Positive
asparagine(133.1/74/RT(min):8.18)	Positive
aspartate(134/74/RT(min):8.58)	Positive
methylnicotinamide(137.001/94/RT(min):14.07)	Positive
lysine(147/67/RT(min):14.08)	Positive
glutamine(147.1/84.1/RT(min):8.05)	Positive
O-acetyl-L-serine(148/106/RT(min):4.87)	Positive
glutamate(148.1/84.1/RT(min):8.21)	Positive
methionine(150.1/133/RT(min):5.46)	Positive
carnitine(162.1/103/RT(min):6.65)	Positive
Methionine sulfoxide(166/74/RT(min):7.00)	Positive
phenylalanine(166.1/103/RT(min):4.63)	Positive
pyridoxine(170/134/RT(min):3.65)	Positive
1-Methyl-Histidine(170.1/124/RT(min):7.52)	Positive
arginine(175.02/60/RT(min):14.59)	Positive
citrulline(176/159/RT(min):8.66)	Positive
N-carbamoyl-L-aspartate(177.05/74/RT(min):10.28)	Positive
tyrosine(182.1/77/RT(min):6.64)	Positive
Phosphorylcholine(184.001/125/RT(min):8.38)	Positive
N6-Acetyl-L-lysine(189.001/84.2/RT(min):7.97)	Positive
Acetyllysine(189.002/84/RT(min):7.97)	Positive
N-acetyl-glutamine(189.1/130/RT(min):4.96)	Positive
N-acetyl-glutamate (190.1/84.1/RT(min):7.89)	Positive

Ng_Ng-dimethyl-L-arginine(203/70/RT(min):12.15)	Positive
Acetylcarnitine DL(204/85/RT(min):5.17)	Positive
tryptophan(205/146/RT(min):5.74)	Positive
Kynurenine(209/146/RT(min):5.17)	Positive
N-acetyl-glucosamine(222/138/RT(min):5.74)	Positive
cystathionine(223/134/RT(min):9.64)	Positive
5-methoxytryptophan(235/176/RT(min):5.21)	Positive
Cystine(241.002/74/RT(min):9.08)	Positive
cytidine(244.1/112/RT(min):5.95)	Positive
thiamine(265/122/RT(min):12.39)	Positive
adenosine(268.15/136.1/RT(min):4.20)	Positive
L-arginino-succinate(291/70/RT(min):9.96)	Positive
glutathione (308.1/162/RT(min):7.8)	Positive
UMP(325/97/RT(min):8.91)	Positive
dAMP(332.1/136/RT(min):6.78)	Positive
thiamine-phosphate(345.2/122/RT(min):7.62)	Positive
AMP(348.15/136/RT(min):7.87)	Positive
IMP(349/137/RT(min):9.31)	Positive
GMP(364/152/RT(min):10.18)	Positive
riboflavin(377/243/RT(min):3.94)	Positive
S-adenosyl-L-homoCysteine_pos(385.1/136/RT(min):6.97)	Positive
S-adenosyl-L-methionine(399.1/250/RT(min):9.1)	Positive
folate(442/295/RT(min):10.47)	Positive
7_8-dihydrofolate(444.2/178/RT(min):9.86)	Positive
glutathione disulfide_pos(613/231/RT(min):10.30)	Positive
FAD(786/348/RT(min):6.25)	Positive
13C-Proline1(122.00/75.00/RT(min):6.38)	Positive
13C-Alanine-1(93.94/47.00/RT(min):7.79)	Positive
13C-Serine-1(110.05/63.00/RT(min):8.48)	Positive
13C-Valine-1(123.93/77.10/RT(min):6.08)	Positive
13C-Glycine-1(78.91/32.00/RT(min):8.53)	Positive
13C-Cystine-1(248.89/155.90/RT(min):9.08)	Positive
13C-Glutamic acid-1(154.05/89.00/RT(min):8.22)	Positive
13C-Leucine-1(138.99/92.00/RT(min):5.07)	Positive
13C-Methionine-1(155.90/138.00/RT(min):5.46)	Positive
13C-Phenylalanine-1(176.01/128.90/RT(min):4.60)	Positive
13C-Tyrosine(192.01/174.00/RT(min):6.64)	Positive
13C tyrosine(192.00/83.00/RT(min):6.64)	Positive
13 C phenylalanine(176.00/111.00/RT(min):4.60)	Positive
13C lysine(155.00/90.10/RT(min):14.08)	Positive
Adenylosuccinate-1(464/252/RT(min): 11.58)	Positive
Isoleucine(132.1/86/RT(min):5.33)	Positive
13C-IsoLeucine-1(138.99/92.00/RT(min):5.33)	Positive

Sample Name	Polarity
glycolate(75/45.2/RT(min):2.90)	Negative
pyruvate(87/43/RT(min):3.55)	Negative
lactate(89/43.2/RT(min):4.09)	Negative
glycerate(105/75/RT(min):5.81)	Negative
fumarate(115/71/RT(min):9.91)	Negative
Maleic acid(115.03/71.03/RT(min):4.45)	Negative
succinate(117/73/RT(min):9.10)	Negative
nicotinate(122/78/RT(min):3.60)	Negative
taurine(124/80/RT(min):7.72)	Negative
Pyroglutamic acid(128/82.1/RT(min):4.60)	Negative
N-Acetyl-L-alanine(130/88/RT(min):3.62)	Negative
Hydroxyisocaproic acid(131.006/85.1/RT(min):2.30)	Negative
malate(133/115/RT(min):9.78)	Negative
a-ketoglutarate(145/101/RT(min):9.42)	Negative
3-methylphenylacetic acid(149.002/105/RT(min):2.34*)	Negative
orotate(155/111/RT(min):4.71)	Negative
allantoin(157.05/114/RT(min):7.19)	Negative
phenylpyruvate(163/91/RT(min):2.38)	Negative
Phenyllactic acid(165.006/103.1/RT(min):2.30)	Negative
quinolinate(166/122/RT(min):9.13)	Negative
Uric acid(167.001/124/RT(min):6.71)	Negative
dihydroxy-acetone-phosphate(169/79/RT(min):9.03)	Negative
D-glyceraldehdye-3-phosphate(169.05/97/RT(min):9.03)	Negative
aconitate(173.05/85/RT(min):11.22)	Negative
allantoate(175/132/RT(min):8.35)	Negative
N-carbamoyl-L-aspartate_neg(175.03/132/RT(min):10.28)	Negative
myo-inositol(179/161/RT(min):9.34)	Negative
hydroxyphenylpyruvate(179.05/107/RT(min):3.59)	Negative
homocysteic acid(182/80/RT(min):9.63)	Negative
4-Pyridoxic acid(182.003/138/RT(min):2.26)	Negative
Indoleacrylic acid(186/142.03/RT(min):3.60)	Negative
Kynurenic acid(188/144/RT(min):2.32)	Negative
citrate-isocitrate(191/111/RT(min):12)	Negative
isocitrate (191.02/117/RT(min):12.12)	Negative
citrate (191.05/87/RT(min):12.12)	Negative
Xanthurenic acid(204.001/160/RT(min):5.65)	Negative
D-glucarate(209/85/RT(min):10.20)	Negative
pantothenate(218/146/RT(min):3.59)	Negative
thymidine(241/125/RT(min):3.46)	Negative

uridine(243/200/RT(min):4.70)	Negative
glucose-6-phosphate(259.02/199/RT(min):10.38)	Negative
inosine(267/135/RT(min):5.41)	Negative
6-phospho-D-gluconate(275/97/RT(min):11.53)	Negative
xanthosine(283/151/RT(min):6.41)	Negative
N-acetyl-glucosamine-1-phosphate(300/79/RT(min):8.6)	Negative
glutathione_neg(306/143/RT(min):7.9)	Negative
cyclic-AMP(328/134.05/RT(min):4.45)	Negative
sucrose(341/179/RT(min):*8.09)	Negative
S-adenosyl-L-homocysteine_neg(383.1/134/RT(min):6.97)	Negative
Deoxycholic acid(391.202/345.2/RT(min):2.11)	Negative
UDP_neg(403/159/RT(min):10.38)	Negative
ADP_neg(426.1/159/RT(min):9.38)	Negative
GDP_neg(442/159/RT(min):11.48)	Negative
Taurodeoxycholic acid(498.2/124/RT(min):2.0)	Negative
ATP_neg(506.1/159/RT(min):10.72)	Negative
UDP-D-glucose(565/323/RT(min):10.05)	Negative
UDP-N-acetyl-glucosamine(606/385/RT(min):9.04)	Negative
glutathione disulfide_neg(611/306/RT(min):10.3*)	Negative
2-hydroxygluterate(147.1/128.7/RT(min):9.18)	Negative
13C-Serine(107.9/77.0/RT(min):8.49)	Negative
13C-Methionine(153.9/48.1/RT(min):5.46)	Negative

QC-star-run-1	QC-star-run-2	QC-end-run-1	QC-end-run-2
Peak Area	Peak Area	Peak Area	Peak Area
1.61E+08	1.57E+08	1.36E+08	1.32E+08
7.55E+06	6.88E+06	6.20E+06	6.55E+06
9.20E+07	9.25E+07	8.14E+07	8.02E+07
4.33E+08	4.06E+08	3.75E+08	3.99E+08
5.41E+05	5.58E+05	4.70E+05	5.09E+05
1.29E+08	1.23E+08	1.13E+08	1.18E+08
6.58E+07	6.20E+07	5.66E+07	5.53E+07
1.86E+08	1.70E+08	1.57E+08	1.70E+08
1.18E+09	1.13E+09	1.08E+09	1.12E+09
1.24E+09	1.20E+09	1.16E+09	1.22E+09
1.86E+07	1.79E+07	1.71E+07	1.82E+07
1.86E+08	1.88E+08	1.82E+08	1.76E+08
1.15E+09	1.15E+09	1.13E+09	1.12E+09
8.40E+06	7.82E+06	7.89E+06	7.69E+06
3.56E+05	3.05E+05	3.33E+05	3.35E+05
6.06E+07	5.66E+07	5.76E+07	5.50E+07
3.66E+05	3.28E+05	3.04E+05	3.29E+05
2.82E+07	2.66E+07	2.40E+07	2.39E+07
5.33E+08	5.36E+08	5.28E+08	5.38E+08
3.50E+07	3.49E+07	3.15E+07	3.25E+07
7.25E+06	7.24E+06	6.03E+06	6.50E+06
5.33E+06	4.93E+06	4.65E+06	4.52E+06
1.15E+07	1.07E+07	9.90E+06	1.06E+07
2.03E+07	1.90E+07	1.82E+07	1.86E+07
3.98E+08	4.08E+08	3.69E+08	3.89E+08
1.00E+06	1.03E+06	9.15E+05	9.85E+05
4.69E+07	4.55E+07	4.30E+07	4.36E+07
1.15E+08	1.15E+08	1.05E+08	1.08E+08
6.97E+08	7.35E+08	6.69E+08	6.63E+08
3.72E+07	3.82E+07	3.46E+07	3.67E+07
2.14E+08	2.03E+08	1.91E+08	1.94E+08
8.10E+05	7.90E+05	6.69E+05	7.05E+05
1.16E+08	1.22E+08	1.22E+08	1.13E+08
2.63E+08	2.79E+08	2.50E+08	2.41E+08
6.99E+08	6.77E+08	6.69E+08	6.76E+08
6.98E+04	8.78E+04	7.40E+04	8.21E+04
3.17E+07	2.91E+07	2.90E+07	3.08E+07
4.36E+07	4.05E+07	3.79E+07	3.81E+07
1.80E+06	1.83E+06	1.72E+06	1.61E+06
1.51E+06	1.53E+06	1.47E+06	1.54E+06
8.86E+06	8.41E+06	7.95E+06	8.45E+06
3.23E+06	3.16E+06	2.90E+06	3.06E+06

2.92E+07	2.50E+07	2.64E+07	2.46E+07
4.66E+08	4.56E+08	4.91E+08	4.59E+08
4.37E+08	4.13E+08	4.00E+08	4.20E+08
8.82E+07	8.08E+07	8.46E+07	8.12E+07
1.30E+06	1.40E+06	1.16E+06	1.31E+06
2.23E+06	2.30E+06	2.34E+06	2.25E+06
3.50E+05	2.89E+05	2.97E+05	4.29E+05
6.81E+06	6.12E+06	6.27E+06	6.27E+06
7.89E+07	7.39E+07	7.49E+07	7.84E+07
1.85E+07	1.74E+07	1.70E+07	1.80E+07
5.28E+06	5.05E+06	4.86E+06	4.83E+06
7.54E+06	7.86E+06	6.88E+06	6.99E+06
1.13E+07	1.12E+07	9.81E+06	9.43E+06
6.39E+05	6.87E+05	6.60E+05	5.80E+05
5.98E+05	6.10E+05	5.87E+05	6.01E+05
9.37E+06	9.55E+06	8.25E+06	8.29E+06
3.70E+08	3.36E+08	3.35E+08	3.34E+08
5.30E+06	5.47E+06	5.13E+06	4.88E+06
1.46E+07	1.37E+07	1.45E+07	1.36E+07
1.68E+06	1.54E+06	1.51E+06	1.44E+06
4.46E+06	4.84E+06	4.41E+06	4.16E+06
7.69E+06	7.34E+06	6.97E+06	7.38E+06
2.52E+05	1.83E+05	2.36E+05	1.71E+05
2.02E+05	2.15E+05	1.94E+05	1.63E+05
2.18E+06	2.10E+06	2.20E+06	1.80E+06
2.39E+05	1.61E+05	2.78E+05	2.14E+05
1.26E+07	1.17E+07	1.09E+07	1.17E+07
1.86E+05	1.64E+05	1.46E+05	1.52E+05
3.65E+05	3.44E+05	3.15E+05	3.16E+05
4.39E+06	4.31E+06	3.99E+06	4.06E+06
1.82E+04	1.61E+04	1.48E+04	9.82E+03
7.84E+05	8.40E+05	7.87E+05	7.57E+05
7.61E+05	6.25E+05	5.83E+05	6.45E+05
4.90E+06	5.28E+06	4.81E+06	4.71E+06
2.89E+06	2.57E+06	2.44E+06	2.52E+06
1.87E+07	1.76E+07	1.77E+07	1.83E+07
8.15E+06	7.57E+06	6.99E+06	7.51E+06
5.87E+05	5.16E+05	5.17E+05	5.30E+05
2.66E+06	2.63E+06	2.55E+06	2.54E+06
7.68E+05	7.80E+05	7.56E+05	7.03E+05
2.28E+05	2.58E+05	2.47E+05	2.40E+05
1.59E+08	1.45E+08	1.38E+08	1.46E+08
3.11E+06	2.96E+06	2.77E+06	3.04E+06

QC-star-run-1	QC-star-run-2	QC-end-run-1	QC-end-run-2
Peak Area	Peak Area	Peak Area	Peak Area
4.61E+06	4.58E+06	5.13E+06	5.08E+06
6.85E+06	7.32E+06	7.26E+06	7.29E+06
5.36E+08	5.45E+08	5.73E+08	5.62E+08
1.21E+06	1.21E+06	1.26E+06	1.12E+06
2.19E+06	2.50E+06	2.43E+06	2.47E+06
4.09E+05	3.93E+05	4.40E+05	3.96E+05
7.14E+06	7.31E+06	7.32E+06	7.33E+06
1.73E+06	1.60E+06	1.97E+06	1.65E+06
2.44E+08	2.38E+08	2.35E+08	2.41E+08
3.32E+05	3.07E+05	3.54E+05	3.39E+05
6.78E+05	6.68E+05	7.87E+05	7.78E+05
6.10E+05	5.44E+05	6.43E+05	5.92E+05
2.15E+07	2.37E+07	2.26E+07	2.36E+07
1.78E+07	1.85E+07	1.63E+07	1.81E+07
4.68E+05	5.25E+05	5.42E+05	4.83E+05
6.17E+06	6.11E+06	6.59E+06	6.39E+06
2.17E+07	2.19E+07	2.21E+07	2.33E+07
2.17E+05	1.78E+05	2.30E+05	2.04E+05
1.08E+05	1.49E+05	1.55E+05	1.43E+05
5.09E+06	4.78E+06	5.05E+06	5.10E+06
4.29E+08	4.06E+08	4.20E+08	4.17E+08
2.46E+06	1.88E+06	2.21E+06	2.72E+06
3.88E+06	2.96E+06	3.27E+06	3.79E+06
1.54E+07	1.56E+07	1.64E+07	1.63E+07
1.63E+05	1.70E+05	1.84E+05	1.22E+05
1.54E+06	1.65E+06	1.42E+06	1.52E+06
4.74E+06	4.75E+06	4.60E+06	4.93E+06
1.02E+06	1.03E+06	1.08E+06	1.08E+06
5.90E+04	6.82E+04	6.48E+04	7.49E+04
3.97E+06	4.15E+06	4.00E+06	4.25E+06
1.03E+07	1.03E+07	1.14E+07	1.09E+07
1.01E+06	1.02E+06	1.15E+06	1.10E+06
5.46E+08	6.09E+08	5.81E+08	6.59E+08
3.54E+06	3.61E+06	3.79E+06	4.09E+06
1.95E+08	2.06E+08	2.19E+08	2.11E+08
4.60E+06	4.52E+06	4.66E+06	4.71E+06
1.81E+06	1.83E+06	1.70E+06	1.76E+06
4.21E+06	4.35E+06	4.64E+06	4.65E+06
8.87E+04	5.60E+04	8.98E+04	1.39E+05

6.54E+04	7.99E+04	6.56E+04	7.54E+04
8.40E+05	7.41E+05	7.85E+05	7.27E+05
2.12E+05	2.01E+05	2.26E+05	2.15E+05
3.10E+05	2.11E+05	2.13E+05	2.61E+05
1.02E+07	1.04E+07	9.95E+06	1.09E+07
1.37E+06	1.14E+06	1.29E+06	1.35E+06
2.55E+06	2.44E+06	2.54E+06	2.31E+06
1.50E+05	1.58E+05	1.72E+05	1.66E+05
2.68E+06	2.65E+06	2.98E+06	2.92E+06
2.48E+06	2.37E+06	2.51E+06	2.39E+06
1.53E+05	1.63E+05	1.75E+05	2.03E+05
7.91E+04	9.03E+04	6.68E+04	9.30E+04
1.42E+06	1.33E+06	1.45E+06	1.42E+06
1.26E+04	1.78E+04	1.24E+04	9.12E+03
6.85E+05	6.34E+05	6.22E+05	6.32E+05
1.06E+05	1.07E+05	1.07E+05	1.01E+05
2.39E+05	2.35E+05	2.25E+05	2.37E+05
2.15E+05	2.03E+05	2.03E+05	2.13E+05
1.64E+06	1.52E+06	1.65E+06	1.58E+06
3.10E+06	3.05E+06	2.94E+06	3.24E+06
7.97E+04	9.01E+04	7.16E+04	9.46E+04
2.37E+05	2.12E+05	2.52E+05	2.26E+05

%CV of QC
9.9
8.4
7.7
6.0
7.4
5.6
8.1
6.9
3.5
2.6
3.6
3.0
1.2
4.0
6.3
4.1
7.8
8.2
0.8
5.1
8.8
7.4
6.1
4.6
4.3
5.0
4.0
4.6
4.7
4.2
5.1
9.1
3.8
6.5
1.9
10.3
4.5
6.7
5.6
1.9
4.4
4.6

7.9
3.4
3.7
4.1
7.7
2.1
18.9
4.8
3.3
3.7
4.2
6.3
9.2
7.1
1.6
7.8
5.1
4.8
3.7
6.3
6.3
4.0
18.9
11.5
9.0
22.0
6.1
10.9
7.2
4.6
24.3
4.4
11.7
5.0
7.6
2.8
6.3
6.2
2.3
4.5
5.2
6.0
4.9

%CV of QC
6.1
3.1
3.0
5.0
5.9
5.2
1.2
9.3
1.7
5.9
8.7
7.0
4.5
5.3
6.9
3.5
3.2
10.8
15.0
3.0
2.2
15.5
12.5
3.2
16.6
6.2
2.8
2.9
10.0
3.1
4.8
6.4
8.0
6.5
4.9
1.8
3.2
4.9
36.8

10.1
6.6
4.9
18.9
3.9
8.0
4.4
5.9
5.9
2.8
12.4
14.6
3.9
27.7
4.4
2.7
2.6
3.2
3.6
4.1
12.3
7.3
